# Synthesis of α-Santonin Derivatives Linked to *N*-, *S*-, and *O*-Heterocycles via 1,2,3-Triazole-Linker: Investigation of Antimicrobial Effects

**DOI:** 10.3390/antibiotics15060611

**Published:** 2026-06-16

**Authors:** Mária Fanni Boncz, Kitti Tari, András Szekeres, Adriána Kovács, István Zupkó, Tam Minh Le, Zsolt Szakonyi

**Affiliations:** 1 Institute of Pharmaceutical Chemistry, University of Szeged, Eötvös utca 6, H-6720 Szeged, Hungary; bonczmaria@gmail.com (M.F.B.); leminhtam1411@gmail.com (T.M.L.); 2 Department of Biotechnology and Microbiology, University of Szeged, Közép fasor 52, H-6726 Szeged, Hungary; kittitari2000@gmail.com (K.T.); andras.j.szekeres@gmail.com (A.S.); 3 Institute of Pharmacodynamics and Biopharmacy, University of Szeged, Eötvös utca 6, H-6720 Szeged, Hungary; kovacs.adriana.judit@szte.hu (A.K.); zupko.istvan@szte.hu (I.Z.); 4 HUN-REN-SZTE Stereochemistry Research Group, Hungarian Research Network, University of Szeged, Eötvös utca 6, H-6720 Szeged, Hungary

**Keywords:** sesquiterpene, α-santonin, Michael addition, click reaction, antibacterial, antifungal

## Abstract

**Background/Objectives**: Resistant pathogenic bacteria and fungi are a growing problem worldwide; therefore, the discovery of new active ingredients is an important challenge for which the functionalization of natural terpenes with biologically active heterocycles can provide a basis. To reach this goal, a series of 1,4-disubstituted-1,2,3-triazole conjugates was designed and synthesized starting from commercially available α-santonin. **Methods**: The key azido derivative intermediate was prepared according to literature procedures via Michael addition between dehydrosantonin and the TMSN_3_/AcOH/Et_3_N system at its highly reactive *α*-methylene-*γ*-lactone motif. Subsequently, the obtained azide was applied to regioselective Huisgen 1,3-dipolar cycloaddition reaction with a wide range of terminal alkynes bearing *N*-, *S*- and *O*-heterocycles. These include pyridine, pyrimidine, purine, quinoline, indol, or coumarin to afford the sesquiterpene–heterocycle chimaeras. All triazole conjugates were screened for in vitro antiproliferative activity by MTT assay against HeLa, MDA-MB231, SiHa, MCF-7 and A2780 human cancer cell lines compared with fibroblast cells (NIH/3T3) to check their cytotoxicity and antimicrobial effects on two Gram-positive (*B. subtilis*, *S. aureus*) pathogenic bacteria, two Gram-negative (*E. coli* and *P. aeruginosa*) pathogenic bacteria, and two yeasts (*C. krusei* and *C. albicans*). **Results**: The results indicated that most of the examined compounds expressed weak activity against human cell lines, while some of them showed moderate activity against *S. aureus* (up to 99% inhibition at 100 µg/mL conc.), *C. krusei* (up to 51% inhibition at 10 µg/mL conc.) and *C. albicans* (up to 52% inhibition at 10 µg/mL conc.). **Conclusions**: Further structural modification of the best, selective antibacterial and antifungal compounds may open the possibility to the development of effective natural sesquiterpene-based selective antimicrobial agents.

## 1. Introduction

Antimicrobial resistance (AMR) and cancer today rank among the biggest global health problems in terms of both prevalence and treatment costs. These threats urgently necessitate the development of new, affordable and easily implementable therapeutic strategies and pharmacological agents. According to a WHO report, AMR directly caused about 1.3 million deaths and contributed to another 5 million cases in 2019. These huge numbers clearly demonstrate that infections are becoming increasingly difficult to treat, especially due to emerging superbugs and resistant fungi, thereby undermining the safety of essential medical interventions such as surgeries [1,2,3].

Sesquiterpene lactones (SLs) constitute a large and structurally diverse group of secondary metabolites produced mainly by plants [4,5,6]. These diverse compounds exhibit a wide range of biological activities, including antitumor, antimicrobial, anti-inflammatory, anti-ulcer and antiviral activities [7]. Concerning the structure–activity relationships (SAR), the observed biological effects of SLs are mainly attributed to the α-methylene-γ-lactone group, which acts as an alkylating agent on cellular proteins via Michael addition, particularly targeting their thiol groups [8]. However, the SLs typically have poor water solubility, and the *α*-methylene-*γ*-lactone moiety can exhibit non-selective binding as a Michael acceptor with undesired targets [9]. Therefore, a semi-synthetic strategy has been widely applied in which the reactive α,β-unsaturated enone is masked; additionally, it can enhance aqueous solubility, improve the pharmacokinetic profile by modifying polarity, and maintain or even augment the biological activity of the parent molecule [10]. In addition, structural modification has enabled an in-depth understanding of their chemical properties and the establishment of structure–activity relationships.

Semi-synthesis is a class of chemical synthesis that transforms a parent bioactive natural compound into a more bioactive or a new compound by incorporating heteroatoms (N, O and/or S), and it has become a modern approach to drug development [11,12,13,14,15]. Among the functional groups in the semi-synthetic precursors useful for such transformation, *α*,*β*-unsaturated carbonyl compounds with the Michael acceptor property are important. They allow both the easy extension of the reaction and the variability of the newly introduced functional groups [16].

α-Santonin, a sesquiterpene lactone, has been reported to be an anticancer, antifungal, antipyretic and anti-inflammatory agent. Interestingly, many α-santonin derivatives presented stronger and better bioactivities, such as anticancer, herbicidal immunosuppressant, phytotoxic and anti-angiogenic properties [17,18,19,20].

To combine two chemically different motifs, the Huisgen 1,3-dipolar cycloaddition reaction (click reaction) between alkynes and azides is an excellent process to build up 1,4-disubstituted 1,2,3-triazoles, which often bear remarkable antiproliferative or antimicrobial effects [21,22,23,24].

Based on our former results on terpene lactones and in a continuation of interest in the synthesis of nitrogen-containing heterocycles with various biological activities [25,26,27], a series of novel and potentially biologically active saturated and unsaturated heterocyclic agents is planned to be prepared from (–)-α-santonin (**1**) [20,28] by the various semi-synthetic modifications employing Huisgen 1,3-dipolar cycloaddition. To the best of our knowledge, triazole-functionalized santonin derivatives are known only with rearranged A-ring or by ring opening of the lactone ring system possessing inhibition effects on T- and B-cell proliferation [18,19], while in this paper, the antimicrobial activity of sesqiterpene-based heterocyclic compounds was in focus. The planned C-3 hetero modification seemed interesting based on the antiproliferative activities of similar amine adducts obtained by Aza-Michael addition [29].

## 2. Results

### 2.1. Synthesis of Key Intermediate Santonin-Based Azide ***3***

**Scheme 1:**
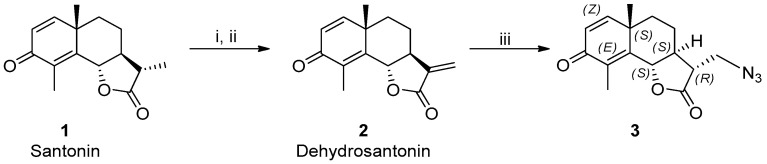
Stereoselective synthesis of azide key intermediate. (i) Diphenyl diselenide (Ph_2_Se_2_, 1.2 eq.), lithium diisopropyl amide (LDA, 2.4 eq., 2 M in THF), hexamethylphosphoramide (HMPA, 1.2 eq.), THF, −78 °C, Ar atm., 3 h, 30% (ii) H_2_O_2_ (6.5 eq., 30%), AcOH (3 eq.), THF, 2 h, 0 °C, 85% (iii) (**1**) TMSN_3_ (5 eq.), AcOH (5 eq.), CH_2_Cl_2_, 25 °C, 30 min; (**2**) TEA (5 eq.), CH_2_Cl_2_, 40 °C, 18 h, 60% (two steps).

Azido derivative **3** was obtained from (–)-α-santonin by a three-step sequence as shown in Scheme 1. Initially, the treatment of (–)-α-santonin (**1**) with lithium diisopropyl amide (LDA) followed by the capture of the organolithium with Ph_2_Se_2_ [20,30] and subsequent elimination of the resulting PhSe-product with hydrogen peroxide in the presence of acetic acid gave dehydrosantonin (**2**) [31]. Dehydrosantonin **2** was subjected to Michael addition using trimethylsilyl azide at its highly reactive *α*-methylene-*γ*-lactone motif to give corresponding azide **3**, which is a single diastereoisomer, as determined by NMR analysis of the crude product.

**Scheme 2:**
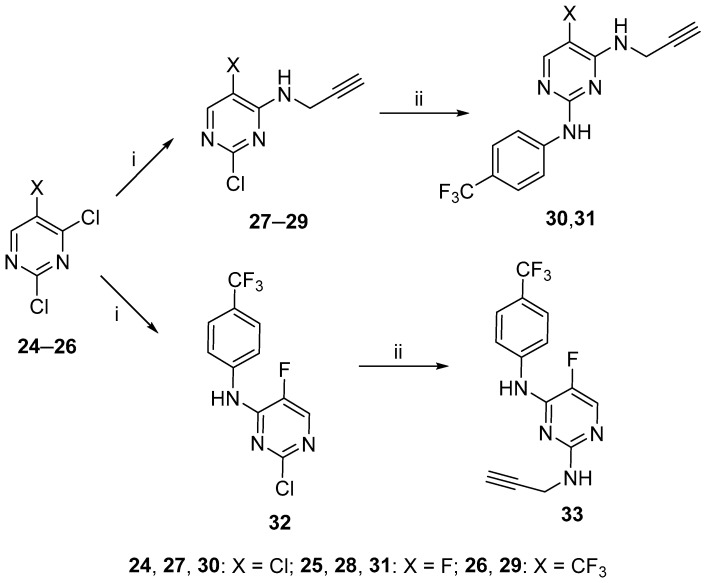
Synthesis of 2,4-diaminopyrimidine building blocks **27**–**33**. (i) Propargylamine or 4-trifluoromethylaniline (1 eq.), TEA (5 eq.), CH_3_CN, 100 °C, 5 h, 54–81%; (ii) 4-Trifluoromethylaniline (1 eq.); EtOH, MW, 200 W, 150 °C, 1.5 h for **30** and **31** or propargylamine (1 eq.), *i*-PrOH, MW, 200 W, 150 °C, 4 h for **33**, 27–82%.

**Figure 1:**
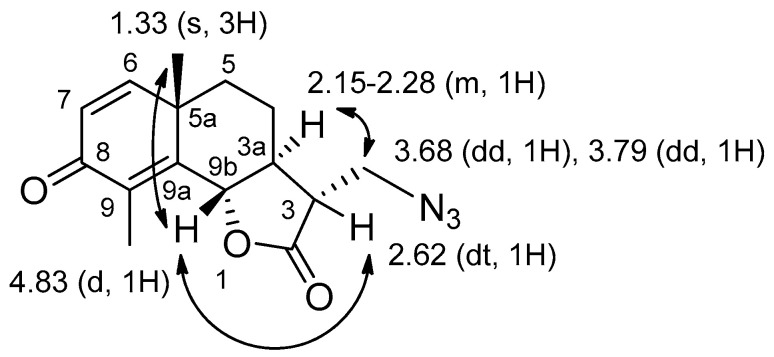
Relative configuration of azido derivative **3** based on NOESY measurement.

**Figure 2:**
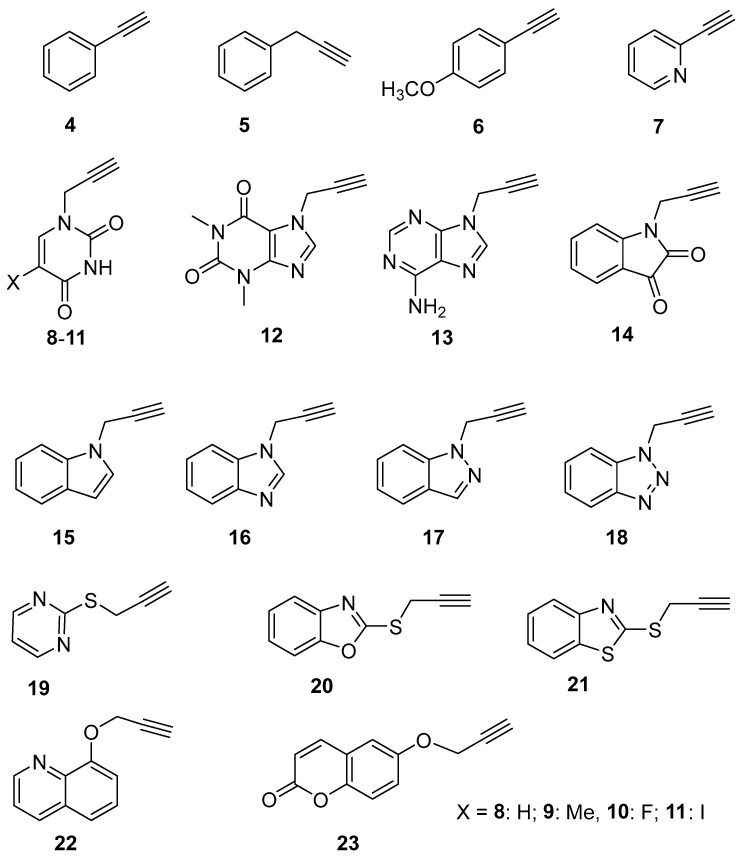
Alkynes, **8**–**23** prepared via *N*-, *S*- and *O*-alkylation by propargyl bromide in the presence of Cs_2_CO_3_ in DMF at 40 °C.

**Scheme 3:**
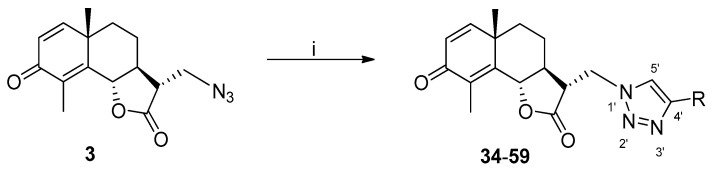
Regioselective Huisgen 1,3-dipolar cycloaddition with diverse heterocyclic alkynes. (i) method A: CuI (1.2 eq.), dry THF, alkyne **8**–**23** and **27**–**33** (1.5 eq.), 70 °C, 24 h; 16–82%; method B: Cu(OAc)_2_. H_2_O (0.1 eq.), sodium ascorbate (0.1 eq.), CH_2_Cl_2_/H_2_O 2:1, alkyne **8**–**23** and **27**–**33** (1.1 eq.), 40 °C, 24 h, 40–71%.

The configuration of the new stereocenter at position C-3 was confirmed by three clear NOE signals recorded between H-3 and H-9b, CH_3_-5a and H-9b and along with H-3a and CH_2_-N_3_ (Figure 1). Since the Huisgen 1,3-dipolar cycloaddition reaction (Figure 2, Scheme 2 and Table 1) does not affect the absolute configuration, the relative configuration of the chiral centers of **34**–**59** (Scheme 3, Table 1) is known to be the same as that of azide **3**. The excellent stereoselectivity of the azide addition can be explained by the steric hindrance of Me-5a and H-9b (Figure 1).

### 2.2. Synthesis of Key-Intermediate Alkynes

The alkynes, used in the synthesis of the target compounds linked with the 1,2,3-triazole linker, were partly commercially available compounds (phenylacetylene **4**, benzylacetylene **5**, 4-ethynylanisole **6** and 2-ethynylpyridine **7**) and partly obtained by the reaction of heterocycles containing the appropriate NH, SH and OH functions with propargyl bromide (compounds **8**–**23**) according to the literature methods [32,33].

The preparation of 2,4-diaminopyrimidine-based alkynes **30** and **31** was also carried out by coupling 2,4,5-trichloro-, 5-fluoro-2,4-dichloro- and 5-trifuoromethyl- 2,4-dichloropyrimidine with propargylamine, resulting in **27**–**29**, which was followed by the reaction of intermediates **27** and **28** with 4-trifluoromethylaniline according to the literature procedure (Scheme 2) [34]. The synthesis of regioisomeric 4-aniline-substituted pyrimidines **32** and **33** was accomplished via an alternative pathway. In the first step, 2-chloro-5-fluoro-6-(4-(trifluoromethyl)phenylamino)pyrimidine **32** was prepared and transferred to **33** in good yields (Scheme 2) [7].

### 2.3. Coupling of the Sesquiterpene Moiety with Alkyne-Functionalized Heterocycles via Click Reaction

To achieve our goal, the synthesis of santonin–heterocycle chimaeras coupling with 1,2,3-triazolo linker, azide **3** was reacted with diverse propargyl-substituted heterocycles (see Section 2.2). The reason is that the 1,2,3-triazole motif can not only serve as a pharmacophore bearing diverse biological properties, it can also act as a linker to connect different pharmacophores. There are even diverse synthetic methodologies for constructing triazoles and, consequently, the CuAAC reaction (copper-catalyzed azide–alkyne cycloaddition, the Huisgen 1,3-dipolar cycloaddition) has gained significant attention, and it was applied extensively to synthesize a broad range of 1,2,3-triazoles [35,36].

Two similar methods were applied to compare them: in method A, CuI was utilized as a catalyst in dry THF solution [32], while in method B, the Cu(OAc)_2_/sodium ascorbate system in CH_2_Cl_2_–H_2_O was used [37]. All reactions proceeded well within 24 h, affording the target products in moderate to good yields. The azide–alkyne cycloaddition reaction using copper(I)-containing catalysts (the CuAAc reaction) proceeds with the predominant formation of 1,4-regioisomers (Scheme 3, Table 1). The regioselectivity of the click reaction was determined by HMBC NMR measurements, whereas clear long-range correlations were observed between the bridge CH_2_ and CH-5’ as well as between C_q_-4’ and the first carbon of R substituent at position 4 (see Figure 1 and Supporting Information). The coupling constants of H-9b and H-3a, and the NOESY experiments, clearly proved that the click reactions did not change the configuration of the stereocenters. Due to biologically undesirable by-products such as dehydroascorbic acid, method B was only used in cases of low yields after applying method A. The reason for some low yields (Table 1) was that contaminating by-products appeared during the reactions, which required purification of the products by multiple recrystallizations or repeated chromatography.

Although method B provided significantly better yields in Entries 1, 17, 23, 24 and 25, its general application was hindered by the fact that in many cases, the solubility of the starting material was inadequate in the mixture used therein.

### 2.4. In Vitro Antiproliferative Studies of 1,4-Disubstituted-1,2,3-triazole–santonin Conjugates

The in vitro antiproliferative potential of the synthesized azide **3** and santonin 1,2,3-triazolo-coupled heterocycles **35**–**59** against a panel of different human cancer cell lines, including cervical (SiHa and HeLa), breast (MCF-7 and MDA-MB-231), and ovarian (A2780) cancers, as well as non-cancerous fibroblast cells, was assayed by the MTT method [38]. Selected results are presented in Table 2, while data for all examined products are presented in Table S1 and Figure S1 in the Supplementary Materials. Analysis of the observed activities showed that most compounds exhibit little or no cytotoxicity. The best and selective antiproliferative effect was observed with azide **3** in SiHa, MCF-7, MDA-MB-231, and A2780 cells without any toxic effect on fibroblast cells (NIH/3T3). Compound **3** exhibited antiproliferative activity with a relatively high IC_50_ value against HeLa cells, but lower values were observed for the other cancer cell lines. This is not a surprising result, since it is well known that in cancer therapy, azido derivatives exhibit anticancer activity through multiple, interconnected mechanisms primarily involving DNA synthesis interference, oxidative stress generation, enzyme inhibition, and apoptosis activation [39,40,41].

Interestingly, compound **34** showed moderate antiproliferative activity against all cancer cell lines but weak activity against fibroblast cells. Between purine- and pyrimidine-based compounds, only uracil **38** (against A2780) and iodouracil **41** (against SiHa) showed weak activity. From the pyrimidine series, 2-chloro-5-trifluoromethyl derivative **56** expressed moderate activity against MCF-7 and A2780 cancer cell lines.

### 2.5. In Vitro Studies of Antibacterial Effects of 1,4-Disubstituted-1,2,3-triazole–santonin Conjugates

Since sesquiterpenes possess remarkable antibacterial and/or antifungal activity [42], and pyrimidine-, purine-, benzimidazole- or even benzothiazole-coupled derivatives also exerted remarkable antimicrobial activities against several bacteria and yeasts [34,43,44,45], the antimicrobial activities of the analogues were tested against two Gram-positive and two Gram-negative bacteria as well as two yeasts (Table 3 and Table S2).

Some of the examined compounds exhibit moderate to good inhibitory activities against *Staphylococcus aureus*, *Candida albicans* and *Candida krusei*. The obtained results clearly show that a heteroatom (even N, O or S) next to the bridge methylene function is essential for the antimicrobial effect—most notably for the antifungal activity. Only compound **52** bearing a quinoline ring system possesses moderate inhibition against both *S. aureus* and *C. albicans* at 100 μg/mL concentration with MIC values of 4 and 16 μg/mL, respectively. Interestingly, five compounds (**37**, **50**, **53**, **54**, and **59**) bearing diverse heterocyclic ring systems exhibited moderate but selective antifungal activity against *C. albicans* at 10 μg/mL, achieving up to 51.8% growth inhibition. Among these, compounds **54** and **59** displayed MIC values of 64 and 2 μg/mL, respectively. In addition, three derivatives (**38**, **43**, and **44**) showed selective antifungal activity against *C. krusei*, resulting in up to 61.1% growth inhibition, while compounds **43** and **44** exhibited MIC values of 8 μg/mL and 2 μg/mL, respectively. These preliminary results clearly showed that ethereal or thioethereal linking is not the reason for obtaining the rich remarkable antibacterial or antifungal activity from the presented library of santonine derivatives.

Furthermore, several examples of the antibacterial, antifungal or even antiviral activities of alicyclic or heterocyclic compounds bearing an azido function were indicated in the literature [33,44,45,46]. Hence, intermediate azide **3** was also applied to the antimicrobial assay. Interestingly, it exhibited moderate and selective antimicrobial activity against both *S. aureus* and *C. albicans* at a concentration of 100 μg/mL with an MIC value of 8 μg/mL against *S. aureus*.

None of these compounds shows cytotoxicity against human cancer or even fibroblast cells (NIH/3T3; see Table 2 and Table S1 in Supplementary Materials). This antibacterial and antifungal activity is in good agreement with our previous results, which were observed for azidodiol-type monoterpenes and diterpenes [27,47].

In the case of the indole (**45**) derivative, the benzimidazole (**46**) derivative, and the benzoxazole (**50**) and benzothiazole (**51**) derivatives, the poor solubility of the compounds did not allow for studies planned at higher concentrations (Supporting Information, Table S2). The same solubility problem also limited the study of the aminopyrimidine derivatives (**56**–**59**).

## 3. Discussion

A series of 1,4-disubstituted-1,2,3-triazole-sesquiterpene conjugates was designed and synthesized starting from commercially available α-santonin via stereoselective Michael addition followed by regioselective Cu(I)-catalyzed alkyne−azide [3 + 2] cycloaddition with a wide range of terminal alkynes bearing *N*-, *S*- and *O*-heterocycles. All triazole conjugates were screened for in vitro antiproliferative activity by MTT assay against human cancer cell lines compared with fibroblast cells (NIH/3T3) and antimicrobial effects on Gram-positive, Gram-negative pathogenic bacteria and yeasts. These preliminary results indicated that most of the examined compounds expressed weak activity against human cell lines, while some of them showed promising activity against *S. aureus* (up to 99% inhibition at 100 µg/mL conc.), *C. krusei* (up to 51% inhibition at 10 µg/mL conc.) and *C. albicans* (up to 52% inhibition at 10 µg/mL conc.).

Among the synthesized derivatives, compounds containing larger aromatic heterocycles generally displayed higher antimicrobial activity than many of the simpler heterocyclic analogues. In particular, the quinoline-containing derivative **52** exhibited the highest antimicrobial activity within the tested series and was one of the few compounds for which MIC values could be determined against both *S. aureus* and *C. albicans*. This observation suggests that the incorporation of fused aromatic heterocycles may be beneficial for antimicrobial activity within this scaffold. Several pyrimidine- and purine-containing derivatives exhibited species-dependent antimicrobial effects. For example, compounds **38**, **43**, and **44** showed inhibitory activity against *C. krusei*, whereas compounds **54** and **59** displayed activity primarily against *C. albicans*. These findings indicate that the heterocyclic moiety influences not only the level of activity but also the microbial selectivity of the resulting conjugates.

Further structural modification of the best, selective antibacterial and antifungal compounds may open the door to the development of effective natural sesquiterpene-based selective antimicrobial agents. The results are an indicative of the fact that compound **52** proved to be the best analog against *S. aureus* and *C. albicans*, which can be explained by the known chelating property of the 8-quinoline (8HQ) scaffold. The mechanism of antimicrobial action of the majority of 8HQ derivatives involves metalloproteins, which are metal ion-dependent proteins, emerging as the main targets in the development of 8HQ derivatives as antimicrobial agents [48]. However, further studies need to be carried out to improve the biological activities of these through revealing the exact mechanism of action, which will be taken up in the future in our research. The rearrangement of the α-santonin A and B ring into an aromatic ring system [17] may serve as an interesting parallel bioactive library in the next paper. The interesting antiproliferative activity of azido intermediate **3** can be explained by that general behavior of azido derivatives, which exhibits promising dual-action profiles targeting both resistant microbes and cancer cells. They primarily target bacterial survival, replication, and membrane integrity through several key pathways such as cell membrane disruption [49].

## 4. Materials and Methods

### 4.1. General Methods

Commercially available reagents were used as obtained from suppliers (Oqema Co., 1089 Budapest, Hungary, Orczy út 6.; Merck Ltd., Budapest, Hungary; and VWR International Ltd., Debrecen, Hungary), while solvents were dried according to standard procedures. Chromatographic separations and reaction monitoring were performed on Merck Kieselgel 60 (Merck Ltd., Budapest, Hungary). Optical rotations were measured in MeOH at 25 °C with a PerkinElmer 341 polarimeter (PerkinElmer Inc., Shelton, CT, USA). Melting points were determined with a Kofler apparatus (Nagema, Dresden, Germany). HRMS flow injection analysis was performed with a Thermo Scientific Q Exactive Plus hybrid quadrupole-Orbitrap (Thermo Fisher Scientific, Waltham, MA, USA) mass spectrometer coupled to a Waters Acquity I-Class UPLC™ (Waters, Manchester, UK). ^1^H-, ^13^C J-MOD- and ^19^F-NMR spectra were recorded on a Bruker Avance DRX 500 spectrometer (Bruker Biospin, Karlsruhe, Baden-Württemberg, Germany) [500 MHz (^1^H), 125 MHz (^13^C J-MOD) and 470 MHz (^19^F) δ = 0 (TMS)]. Chemical shifts are expressed in ppm (δ) relative to TMS as an internal reference. J values are given in Hz. All ^1^H-, ^13^C, J-MOD-, ^19^F-NMR, COSY, NOESY, 2D-HMBC and 2D-HSQC spectra are available in the Supporting Information files. In CDCl_3_, hexane residual (δ_H_ = 1.15), water (δ_H_ = 1.56) and CH_2_Cl_2_ (δ_H_ = 5.30, δ_C_ = 53.5) were preferred as impurities in the NMR spectra, while in the case of DMSO-d_6_, water (δ_H_ = 3.33) and residual CH_2_Cl_2_ (δ_H_ = 5.76, δ_C_ = 84.8) were obtained as impurities in the NMR spectra.

### 4.2. Starting Materials

α-Santonin is commercially available from Merck Co. with *ee*% > 99% ([α]_D_^25^ = −170 (c 1, EtOH)). All propargyl-functionalized heterocycles were prepared as prescribed in the literature, starting from nucleobases (uracil, 5-fluorouracil, 5-iodouracil, thymine, adenine) or other heterocycles (theophylline, indole, indazole, isatin, benzotriazole, benzimidazole, 8-hydroxyquinoline, 7-hydroxycoumarin, 2-mercaptobenzoxazole, 2-mercaptobenzothiazol, 2-mercaptopyrimidine) and propargyl bromide applying K_2_CO_3_ or Cs_2_CO_3_ as bases [32,33]. The synthesis of 2,4,5-disubstututed pyrimidines was accomplished according to our former results and literature methods [7,34]. All physical–chemical and spectroscopic data were similar to those described therein. In the Section 4, in addition to azide **3** and the newly prepared diaminopyrimidine compound **33**, the synthesis and characterization of compounds **34**–**36** are presented as examples of methods A and B, while a detailed description of compounds **37**–**57** is included in the Supporting Information.

**(3*R*,3a*S*,5a*S*,9b*S*)-3-(Azidomethyl)-5a,9-dimethyl-3a,4,5,5a-tetrahydronaphtho[1,2-*b*]furan-2,8(3*H*,9b*H*)-dione (3)**

To a stirred solution of trimethylsilylazide (0.269 mL, 5 eq.) in dry CH_2_Cl_2_ (5 mL), glacial AcOH (0.117 mL, 5 eq.) was added, and the mixture was stirred at 25 °C for 30 min before a solution of 11-*exo*-metylene-santonin (100 mg, 0.41 mmol) in dry CH_2_Cl_2_ (1.5 mL) was added. A catalytic amount of triethylamine (0.285 mL, 5 eq.) was then added dropwise to the reaction; then, the mixture was stirred at 40 °C for 18 h. When the reaction was complete (as indicated by TLC), the mixture was poured into water (15 mL) and then extracted with CH_2_Cl_2_ (3 × 10 mL). The combined organic layer was combined and washed with saturated NaHCO_3_ solution (3 × 10 mL) and brine (3 × 10 mL); then, it was dried over dry Na_2_SO_4_, filtered and evaporated to dryness. The resulting crude product was purified on silica gel column eluting with CHCl_3_/MeOH 19:1 mixture to afford the product as yellow crystals.

The reaction yield was 70.4 mg (60%); yellow crystals; m.p.: 111.3–115.5 °C; [α]_D_^20^ = −87 (c 0.160 MeOH); ^1^H-NMR (500 MHz, CDCl_3_) δ (ppm): 1.33 (s, 3H), 1.51–1.60 (m, 1H, overlapping with water), 1.74 (ddd, 1H, *J* = 3.6, 13.0, 26.3 Hz), 1.87–1.93 (m, 1H), 2.10–2.18 (m, 5H), 2.62 (dt, 1H, *J* = 5.8, 12.2 Hz), 3.68 (dd, 1H, *J* = 6.2, 12.8 Hz), 3.79 (dd, 1H, *J* = 4.0, 12.8 Hz), 4.83 (d, 1H, *J* = 11.2 Hz), 6.26 (d, 1H, *J* = 9.9 Hz), 6.69 (d, 1H, *J* = 9.9 Hz); ^13^C-NMR (125 MHz, CDCl_3_) δ (ppm): 10.9 (CH_3_), 23.3 (CH_2_), 25.2 (CH_3_), 37.8 (CH_2_), 41.2 (C_q_), 46.0 (CH), 49.0 (CH_2_), 49.5 (CH), 81.4 (CH), 126.0 (CH), 129.2 (C_q_), 150.0 (C_q_), 154.6 (CH), 174.0 (C_q_), 186.0 (C_q_); HRMS (ESI+): *m*/*z* calcd. for C_15_H_18_N_3_O_3_^+^ [M + H]^+^ 288.13427 found 288.13395.

**5-Fluoro-*N*^2^-(prop-2-yn-1-yl)-*N*^4^-(4-(trifluoromethyl)phenyl)pyrimidine-2,4-diamine (33)**

To the solution of pyrimidine 32 (50 mg, 0.17 mmol) in *i*-PrOH (4 mL), propargylamine (0.327 mL, 3 eq.) and triethylamine (0.120 mL, 5 eq.) were added. The mixture was heated in a microwave reactor at 150 °C, 200 W, 19 bar for 4 h. The formed precipitate was filtered off to afford the desired product in pure form without further purification. The reaction mixture was evaporated to dryness, and the resulting crude product was purified on a silica gel column eluting with n-hexane/EtOAc 3:1 mixture to afford the product as brown crystals.

The reaction yield was 27%; brown crystals; m.p.: 155.3–160.1 °C; ^1^H-NMR (500 MHz, (CD_3_)_2_SO) δ (ppm): 3.03 (s, 1H), 3.95–4.00 (m, 2H), 7.26 (t, 1H, *J* = 5.8 Hz), 7.63 (d, 2H, *J* = 8.6 Hz), 8.07 (d, 1H, *J* = 3.7 Hz), 8.14 (d, 2H, *J* = 8.5 Hz), 9.60 (s, 1H); ^13^C-NMR (125 MHz, (CD_3_)_2_SO) δ (ppm): 31.3 (CH_2_), 72.4 (CH), 82.9 (C_q_), 120.4 (2 × CH_2_), 122.7 (C_q_, *J* = 31.9 Hz), 125.0 (C_q_, *J* = 271.4 Hz), 126.0 (2 × CH, *J* = 3.7 Hz), 140.5 (C_q_, *J* = 245.8 Hz), 142.1 (CH), 143.6 (C_q_), 149.6 (C_q_, *J* = 10.8 Hz), 158.0 (C_q_); ^19^F-NMR (470 MHz, (CD_3_)_2_SO) δ (ppm): −166.6, −60.1; HRMS (ESI+): *m*/*z* calcd. for C_14_H_11_F_4_N_4_^+^ [M + H]^+^ 311.09198; found 311.09080.

### 4.3. General Procedures for the Preparation of 1,2,3-Triazol Derivatives

Method A: To a solution of compound **3** (50 mg, 0.17 mmol) in dry THF (2 mL), the appropriate terminal alkynes (0.26 mmol) followed by a catalytic amount of CuI (39 mg) under inert atmosphere was added. The resulting mixture was stirred at 70 °C for 24 h. The reaction mixture was poured into brine (20 mL) and was extracted with ethyl acetate (3 × 10 mL). The combined organic layer was dried over anhydrous Na_2_SO_4_, filtered, and concentrated under reduced pressure. The residue was purified with chromatography on silica gel with CHCl_3_/MeOH 19:1 to afford the products.

Method B: Alkyne (0.19 mmol), sodium ascorbate (3.5 mg) and a solution of Cu(OAc)_2_.H_2_O (3.5 mg) in H_2_O (5.0 mL) were successively added to a stirred solution of azide **3** (50 mg, 0.17 mmol) in CH_2_Cl_2_ (10 mL). The reaction mixture was stirred at 40 °C for 24 h (TLC control). After the completion of the reaction, H_2_O (20 mL) was added to the mixture, and the products were extracted with CH_2_Cl_2_ (3 × 20 mL). The combined organic layers were dried over MgSO_4_, and evaporated. The residue (compounds **34**, **50**, **56**, **58** and **59**) was loaded onto a silica gel column (eluent CHCl_3_/MeOH = 19:1).

**(3*R*,3a*S*,5a*S*,9b*S*)-5a,9-Dimethyl-3-((4-phenyl-1H-1,2,3-triazol-1-yl)methyl)-3a,4,5,5a-tetrahydronaphtho[1,2-*b*]furan-2,8(3*H*,9b*H*)-dione (34)**

The reaction was implemented using phenylacetylene (**4**) according to the general procedures A and B. Yield: 33.0 mg (49%, method A) and 48.0 mg (71%, method B); yellowish white crystals; m.p.: 218.7–219.5 °C; [α]_D_^20^ = −5 (c 0.148 MeOH); ^1^H-NMR (500 MHz, CDCl_3_) δ (ppm): 1.29 (s, 3H), 1.48 (dt, 1H, *J* = 4.3, 13.3 Hz), 1.67 (ddd, 1H, *J* = 3.4, 12.5, 25.4 Hz), 1.80–1.98 (m, 3H), 2.05 (s, 3H), 3.02 (dt, 1H, *J* = 5.0, 12.5 Hz), 4.75 (dd, 1H, *J* = 5.5, 14.5 Hz), 4.87 (dd, 1H, *J* = 4.5, 14.5 Hz), 4.88 (s, 1H), 6.23 (d, 1H, *J* = 9.9 Hz), 6.64 (d, 1H, *J* = 9.9 Hz), 7.34 (t, 1H, *J* = 7.3 Hz). 7.43 (t, 2H, *J* = 7.4 Hz), 7.82 (d, 2H, *J* = 7.8 Hz), 7.89 (s, 1H). ^13^C-NMR (125 MHz, CDCl_3_) δ (ppm): 10.9 (CH_3_), 22.8 (CH_2_), 25.2 (CH_3_), 37.4 (CH_2_), 41.1 (C_q_), 47.1 (CH), 47.4 (CH_2_), 49.4 (CH), 81.6 (CH), 120.6 (CH), 125.8 (2 × CH), 126.0 (CH), 128.5 (CH), 128.9 (2 × CH), 129.3 (C_q_), 130.1 (C_q_), 148.5 (C_q_), 149.5 (C_q_), 154.5 (CH), 174.0 (C_q_), 185.9 (C_q_). HRMS (ESI+): *m*/*z* calcd. for C_23_H_24_N_3_O_3_^+^ [M + H]^+^ 390.18122.; found 390.18062.

**(3*R*,3a*S*,5a*S*,9b*S*)-3-((4-benzyl-1*H*-1,2,3-triazol-1-yl)methyl)-5a,9-dimethyl-3a,4,5,5a-tetrahydronaphtho[1,2-*b*]furan-2,8(3*H*,9b*H*)-dione (35)**

The reaction was implemented using benzylacetylene (**5**) according to the general procedure A. Yield: 37.3 mg (53%); white crystals; m.p.: 80.2–80.6 °C; [α]_D_^20^ = −27 (c 0.152 MeOH); ^1^H-NMR (500 MHz, CDCl_3_) δ (ppm): 1.28 (s, 3H), 1.44 (dt, 1H, *J* = 3.5, 13.5 Hz), 1.61 (dd, 1H, *J* = 14.0, 24.4 Hz), 1.72–1.90 (m, 3H), 2.04 (s, 3H), 2.94 (dt, 1H, *J* = 4.8, 12.4 Hz), 4.07 (s, 2H), 4.62 (dd, 1H, *J* = 5.5, 12.5 Hz), 4.76–4.85 (m, 2H), 6.24 (d, 1H, *J* = 9.9 Hz), 6.65 (d, 1H, *J* = 10.0 Hz), 7.18–7.33 (m, 6H, overlapping with CDCl_3_). ^13^C-NMR (125 MHz, CDCl_3_) δ (ppm): 10.8 (CH_3_), 22.8 (CH_2_), 25.1 (CH_3_), 32.2 (CH_2_), 37.4 (CH_2_), 41.1 (C_q_), 47.0 (CH), 47.2 (CH_2_), 49.3 (CH), 81.5 (CH), 122.5 (CH), 126.0 (CH), 126.6 (CH), 128.5 (2 × CH), 128.7 (2 × CH), 129.2 (C_q_), 138.8 (C_q_), 148.5 (C_q_), 149.6 (C_q_), 154.6 (CH), 174.0 (C_q_), 185.9 (C_q_). HRMS (ESI+): *m*/*z* calcd. for C_24_H_26_N_3_O_3_^+^ [M + H]^+^ 404.19687; found 404.19625.

**(3*R*,3a*S*,5a*S*,9b*S*)-3-((4-(4-methoxyphenyl)-1*H*-1,2,3-triazol-1-yl)methyl)-5a,9-dimethyl-3a,4,5,5a-tetrahydronaphtho[1,2-*b*]furan-2,8(3*H*,9b*H*)-dione (36)**

The reaction was implemented applying 1-ethynyl-4-methoxybenzene (**6**) according to the general procedure method A. Yield: 46.5 mg (64%); white crystals; m.p.: 191.6–191.8 °C; [α]_D_^20^ = −4 (c 0.146 MeOH); ^1^H-NMR (500 MHz, CDCl_3_) δ (ppm): 1.29 (s, 3H), 1.48 (dt, 1H, *J* = 4.2, 13.5 Hz), 1.67 (ddd, 1H, *J* = 3.2, 12.5, 25.3 Hz), 1.78–1.99 (m, 3H), 2.05 (s, 3H), 3.01 (dt, 1H, *J* = 5.0, 12.3 Hz), 3.84 (s, 3H), 4.73 (dd, 1H, *J* = 5.5, 14.7 Hz), 4.83–4.91 (m, 2H), 6.23 (d, 1H, *J* = 9.9 Hz), 6.64 (d, 1H, *J* = 9.9 Hz), 6.96 (d, 2H, *J* = 8.1 Hz), 7.74 (d, 2H, *J* = 8.0 Hz), 7.80 (s, 1H). ^13^C-NMR (125 MHz, CDCl_3_) δ (ppm): 10.9 (CH_3_), 22.8 (CH_2_), 25.2 (CH_3_), 37.4 (CH_2_), 41.1 (C_q_), 47.1 (CH), 47.3 (CH_2_), 49.3 (CH), 55.4 (CH_3_), 81.6 (CH), 114.3 (2 × CH), 119.8 (CH), 122.8 (C_q_), 126.0 (CH), 127.1 (2 × CH), 129.3 (C_q_), 148.3 (C_q_), 149.5 (C_q_), 154.6 (CH), 159.9 (C_q_), 174.1 (C_q_), 185.9 (C_q_). HRMS (ESI+): *m*/*z* calcd. for C_24_H_26_N_3_O_4_^+^ [M + H]^+^ 420.19178; found 420.19111.

### 4.4. Determination of the Antiproliferative Properties

The growth-inhibitory effects of the isosteviol-based 1,3-aminoalcohols were determined by a standard MTT (3-(4,5-dimethylthiazol-2-yl)-2,5-diphenyltetrazolium bromide) assay on a panel containing five cell lines, including HeLa, SiHa (cervical cancer), MDA-MB-231 and MCF-7 (breast cancers) and A2780 ovarian cancer cells. NIH/3T3 fibroblasts were additionally used to obtain preliminary information on the cancer selectivity. All cell lines were purchased from the European Collection of Cell Cultures (Salisbury, UK) except for SiHa (American Type Culture Collection (ATCC), Manassas, VA, USA). The cells were maintained in a minimal essential medium supplemented with 10% fetal bovine serum, 1% non-essential amino acids and 1% penicillin–streptomycin at 37 °C in a humidified atmosphere containing 5% CO_2_. All media and supplements were obtained from Lonza Group Ltd. (Basel, Switzerland). Cancer cells were seeded into 96-well plates (5000 cells/well); after an overnight incubation, the test compound was added at two concentrations (10 µM and 30 µM) and incubated for another 72 h under cell-culture conditions. In the next step, 20 μL of 5 mg/mL MTT solution was added to each well and incubated for a further 4 h. The medium was removed, and the precipitated formazan crystals were dissolved in DMSO after 60 min of shaking at 37 °C. As a final step, the absorbance was measured at 545 nm by using a microplate reader. Untreated cells were included as controls. Two independent experiments were performed with five wells per condition in each. For the most effective compound (**3**), the assays were repeated using a dilution series (0.1–30 µM) to determine the IC_50_ values. Calculations were performed using GraphPad Prism 10.0 (GraphPad Software, Inc., San Diego, CA, USA).

### 4.5. Antimicrobial Analyses Method

Stock solutions of the synthesized compounds were prepared in MeOH and subsequently diluted with H_2_O to final concentrations of up to 100 µg/mL and 10 µg/mL, maintaining a final MeOH content of 10%. Complete dissolution was visually confirmed in all cases.

The resulting solutions were evaluated using a microdilution assay against a panel of bacterial and yeast strains, including *Bacillus subtilis* SZMC 0209 (Gram-positive), *Staphylococcus aureus* SZMC 14611 (Gram-positive), *Escherichia coli* SZMC 6271 (Gram-negative), *Pseudomonas aeruginosa* SZMC 0568 (Gram-negative), as well as *Candida albicans* SZMC 1533 and *Candida krusei* SZMC 1352. Fresh overnight cultures were grown at 37 °C in bacterial medium (10 g/L peptone, 5 g/L NaCl, 5 g/L yeast extract) or yeast medium (20 g/L peptone, 10 g/L yeast extract, 20 g/L glucose). Microbial suspensions were prepared and adjusted with sterile medium to a cell density previously optimized in antimicrobial assays.

For the assay, 100 µL of microbial suspension, 50 µL of sterile broth, and 50 µL of the test solution were added to each well of a 96-well microplate. Plates were incubated for 24 h at 37 °C.

The blank consisted of 150 µL broth and 50 µL of test solution and was used for background correction. The negative control contained 100 µL microbial suspension, 50 µL sterile broth, and 50 µL of 10% MeOH. Ampicillin and nystatin were used as positive controls for bacteria and fungi, respectively, at final concentrations of 100 µg/mL and 10 µg/mL.

The inhibitory effect was expressed as a percentage relative to the negative control after blank correction, according to the following equation: inhibitory effect (%) = 100 − ((OD_test_ − OD_blank_)/(OD_negative_ − OD_blank_) × 100), where OD_test_, OD_negative_ and OD_blank_ represent optical density values of the test samples, negative control and blank, respectively.

The MIC was also determined for certain compounds, which were based on the broth microdilution method described above and in the M07-A10 CLSI guideline [50]. The compounds were prepared in two-fold dilutions in 10% MeOH covering the final concentration range of 1–64.00 µg/mL. The MIC was observed as the lowest concentration level of the compound that completely inhibits the growth of the organism in microdilution wells. All experiments were repeated three times.

## Figures and Tables

**Table 1:** “Click” reaction mediated synthesis of santonin conjugates **34**–**59**.

Entry	Alkyne	Product	Yield (%) ^a^
1	**4**	**34**	49/71 ^b^
2	**5**	**35**	53
3	**6**	**36**	64
4	**7**	**37**	68
5	**8**	**38**	67
6	**9**	**39**	75
7	**10**	**40**	44
8	**11**	**41**	56
9	**12**	**42**	40
10	**13**	**43**	50
11	**14**	**44**	40
12	**15**	**45**	63
13	**16**	**46**	49
14	**17**	**47**	73
15	**18**	**48**	70
16	**19**	**49**	59
17	**20**	**50**	22/47 ^b^
18	**21**	**51**	68
19	**22**	**52**	47
20	**23**	**53**	82
21	**27**	**54**	74
22	**28**	**55**	69
23	**29**	**56**	41/48 ^b^
24	**30**	**57**	41
25	**31**	**58**	16/48 ^b^
26	**33**	**59**	21/40 ^b^

^a^ Isolated yield after purification. ^b^ Method B, see Scheme 3.

**Table 2:** Selected data of the most active derivatives **3**, **38**, **41** and **56**.

Comp.	Conc. (μM)	Growth Inhibition (%) ± SEM ^1^					
		HeLa	SiHa	MCF-7	MDA-MB-231	A2780	NIH/3T3
**3**	10	23.95 ± 2.95	51.53 ± 2.09	40.14 ± 2.99	44.44 ± 2.30	13.61 ± 3.01	– ^2^
	30	53.74 ± 2.03	76.31 ± 0.89	88.08 ± 0.95	89.60 ± 0.82	84.28 ± 0.30	40.02 ± 3.18
Calculated IC_50_ (μM) ^3^		25.61	10.21	11.89	11.19	17.64	>30
**34**	10	–	–	–	17.18 ± 2.96	–	–
	30	55.50 ± 1.37	42.69 ± 0.68	56.82 ± 0.79	50.62 ± 1.27	66.54 ± 2.59	26.80 ± 3.15
**38**	10	–	–	–	–	–	–
	30	–	–	21.24 ± 3.06	–	45.64 ± 1.58	–
**41**	10	11.43 ± 2.12	27.08 ± 2.68	24.83 ± 2.39	–	–	13.13 ± 2.07
	30	23.17 ± 1.35	41.48 ± 2.54	33.45 ± 3.03	10.48 ± 2.91	20.21 ± 3.37	15.32 ± 2.82
**56**	10	15.64 ± 1.95	–	–	–	17.44 ± 2.46	–
	30	37.31 ± 1.64	26.91 ± 1.11	49.80 ± 2.50	20.20 ± 1.96	70.17 ± 1.45	–

^1^: Mean ± SEM values from two determinations with five parallel wells in each. ^2^: Inhibition values less than 10% are regarded as negligible and are not given numerically. ^3^: Calculated from viability results of experiments applying a broad range of concentrations (0.1–30 μM).

**Table 3:** Antimicrobial activities of selected compounds according to Figure 2.

Comp. ^1,2^	Conc. (µg/mL)	Growth Inhibition (%) ^2^ ± SEM ^3^					
		*B. subtilis* SZMC 0209	*S. aureus* SZMC 14611	*E. coli* SZMC 6271	*P. aeruginosa* SZMC 0568	*C. albicans* SZMC 1533	*C. krusei* SZMC 1352
**Amp**	100	99.7 ± 1.7	100 ± 5.4	100.2 ± 4.1	100 ± 6.7	-	-
	10	88.8 ± 9.8	99.6 ± 8.5	72.4 ± 15.8	27.5 ± 6.5	-	-
**Nyst**	100	-	-	-	-	98.9 ± 0.9	99.3 ± 1
	10	-	-	-	-	99.4 ± 1.5	99 ± 1.9
**3**	100	3.2 ± 13.2	99.2 ± 14.3	8.3 ± 3.2	0	45.4 ± 16.8	10.9 ± 8.6
	10	15.3 ± 5.1	19.6 ± 6.5	1 ± 1.1	0	5.1 ± 15.7	2.5 ± 16.6
**37**	100	7.3 ± 15.9	13.9 ± 8.9	0	0	45.9 ± 14.2	8.5 ± 9.6
	10	8.6 ± 9.6	16.3 ± 6.8	2.8 ± 3.1	0	49.1 ± 13.7	8.6 ± 7.9
**38**	100	2.9 ± 4.1	12.6 ± 6.8	0	0	20.5 ± 14.4	45.9 ± 27.9
	10	18.5 ± 4.2	8.8 ± 3.7	0	0	0	0
**43**	100	14.5 ± 9.5	32.2 ± 5.7	0	0	40.9 ± 7.7	61.3 ± 15.1
	10	23.6 ± 5.9	13.8 ± 5.7	0	0	0	22.5 ± 6.6
**44**	100	30.6 ± 12.4	2.2 ± 9.5	12 ± 5.1	0	1.8 ± 11.9	0
	10	28.1 ± 6.9	1.4 ± 9.3	11.7 ± 3.8	0	0	51.4 ± 17.3
**50**	100	-	-	-	-	-	-
	10	6.8 ± 7.4	9.4 ± 7.1	19 ± 2.1	14.6 ± 2.3	46.8 ± 14.5	0
**52**	100	25.3 ± 12.9	98.9 ± 17.9	25.4 ± 4.7	5.5 ± 7.5	73.5 ± 21.2	19.6 ± 4.7
	10	35.2 ± 9.9	7.9 ± 4.7	15.7 ± 2.2	9.5 ± 6.2	3.3 ± 12.9	0
**53**	100	2.2 ± 17.5	8.9 ± 10.5	14.9 ± 6.4	18.1 ± 4.6	26.7 ± 16.7	6.4 ± 22.9
	10	13.2 ± 5.3	5.1 ± 7.5	20.5 ± 4.3	22.2 ± 3.1	45.7 ± 19.6	0
**54**	100	7.9 ± 12.8	7.1 ± 5.9	0	0	51.8 ± 10.6	2.1 ± 8.7
	10	16.0 ± 6.3	13.4 ± 3.6	8.3 ± 3	0	10.4 ± 14.2	5.1 ± 5.5
**59**	100	-	-	-	-	-	-
	10	2.6 ± 4.9	12.4 ± 8	6.3 ± 0.9	0	50.1 ± 20.6	17.1 ± 10.4

^1^: Amp: ampicillin, Nys: nystatin; numerical values for all prepared compounds are given in Table S2 in the Supplementary Materials. ^2^: Solutions, which were not tested due to solubility problems, are marked with a hyphen (-). ^3^: Mean ± SEM values from two determinations with 3 parallel wells in each.

## Data Availability

The original contributions presented in this paper are included in the body of the paper and the Supplementary Materials. Further inquiries can be directed to the corresponding author.

## Supplementary Materials

The following supporting information can be downloaded at https://www.mdpi.com/article/10.3390/antibiotics15060611/s1, Figure S1: Determination of IC50 values of azide 3 on HeLa, MDA-MB231, SiHa, MCF-7 and A2780 human cancer cell and NIH/3T3 fibroblast cell tissues, Figures S2–S100: 1H, 13C, 19F NMR, COSY, NOESY, HSQC, HMBC and HRMS spectra of new compounds; Table S1: The antiproliferative effect and IC50 values of the prepared new compounds; Table S2: Investigation of antibacterial and antifungal activity.

## Abbreviations

The following abbreviations are used in this manuscript:

AMR	Antimicrobial resistance
HMPA	Hexamethylphosphoramide
LDA	Lithium diisopropyl amide
MW	Microwave
TEA	Triethylamine
