# Supplementary material for: Synthesis of α-Santonin Derivatives Linked to N-, S-, and O-Heterocycles via 1,2,3-Triazole-Linker: Investigation of Antimicrobial Effects
Source: Antibiotics (Basel). 2026 Jun 16;15(6):611. doi: 10.3390/antibiotics15060611 (PMC13295796; doi:10.3390/antibiotics15060611)

Supporting information

for

Synthesis of α-santonin derivatives linked to *N*-, *S*-and *O*-heterocycles *via* 1,2,3-triazole-linker: investigation of antimicrobial effects

Mária Fanni Boncz [1], Kitti Tari [2], András Szekeres [2], Adriána Kovács [3], István Zupkó [3], Tam Minh Le [1,4] and Zsolt Szakonyi [1]*

[1]Institute of Pharmaceutical Chemistry, University of Szeged, Eötvös utca 6, H-6720 Szeged, Hungary; MFB: bonczmaria@gmail.com; ZS: szakonyi.zsolt@szte.hu
[2]Department of Biotechnology and Microbiology, University of Szeged, Közép fasor 52. H-6726 Szeged Hungary; AS: andras.j.szekeres@gmail.com; KT: kittitari2000@gmail.comInstitute of
[3]Pharmacodynamics and Biopharmacy, University of Szeged, H-6720 Szeged, Eötvös utca 6, Hungary; IZ: zupko.istvan@szte.hu; AK: kovacs.adriana.judit@szte.hu;
[4] HUN-REN-SZTE Stereochemistry Research Group, Hungarian Research Network, University of Szeged, Eötvös u. 6, H-6720 Szeged, Hungary; TML: leminhtam1411@gmail.com
*Correspondence: szakonyi.zsolt@szte.hu; Tel.: +36-62-546809

Contents

Experimental part S4-S18

Investigation of antiproliferative activity, Table S1 S19–S21

Determination of IC_{50} values of azide **3**, Figure S1 S22

Investigation of antibacterial and antifungal activity, Table S2 S23–S26

^{1}H, ^{13}C, ^{19}F NMR, COSY, NOESY, HSQC, HMBC spectra of new compounds S27–S126

Compound **3** S28-S33

Compound **33** S34-S38

Compound **34** S39-S40

Compound **35** S41-S42

Compound **36** S43-S44

Compound **37** S45-S46

Compound **38** S47-S50

Compound **39** S51-S55

Compound **40** S56-S60

Compound **41** S61-S62

Compound **42** S63-S66

Compound **43** S67-S70

Compound **44** S71-S72

Compound **45** S73-S76

Compound **46** S77-S80

Compound **47** S81-S82

Compound **48** S83-S84

Compound **49** S85-S86

Compound **50** S87-S90

Compound **51** S91-S94

Compound **52** S95-S98

Compound **53** S99-S100

Compound **54** S101-S102

Compound **55** S103-S106

Compound **56** S107-S111

Compound **57**	S112-S116
Compound **58**	S117-S120
Compound **59**	S121-S126

Experimental part

(3*R*,3a*S*,5a*S*,9b*S*)-3-(Azidomethyl)-5a,9-dimethyl-3a,4,5,5a-tetrahydronaphtho[1,2-b]furan-2,8(3*H*,9b*H*)-dione (3)

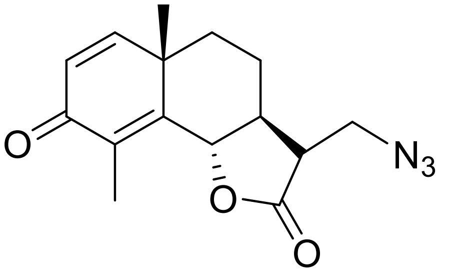

The reaction yield was 70.4 mg (60%); yellow crystals; m.p.: 111.3–115.5 °C; $[\alpha]_D^{20}$ = –87 (c 0.160 MeOH); ^{1}H-NMR (500 MHz, $CDCl_3$) δ (ppm): 1.33 (s, 3H), 1.51-1.60 (m, 1H, overlapping with water), 1.74 (ddd, 1H, *J* = 3.6, 13.0, 26.3 Hz), 1.87-1.93 (m, 1H), 2.10-2.18 (m, 5H), 2.62 (dt, 1H, *J* = 5.8, 12.2 Hz), 3.68 (dd, 1H, *J* = 6.2, 12.8 Hz), 3.79 (dd, 1H, *J* = 4.0, 12.8 Hz), 4.83 (d, 1H, *J* = 11.2 Hz), 6.26 (d, 1H, *J* = 9.9 Hz), 6.69 (d, 1H, *J* = 9.9 Hz); ^{13}C-NMR (125 MHz, $CDCl_3$) δ (ppm): 10.9 (CH_3), 23.3 (CH_2), 25.2 (CH_3), 37.8 (CH_2), 41.2 (C_q), 46.0 (CH), 49.0 (CH_2), 49.5 (CH), 81.4 (CH), 126.0 (CH), 129.2 (C_q), 150.0 (C_q), 154.6 (CH), 174.0 (C_q), 186.0 (C_q); HRMS (ESI+): m/z calcd. for $C_{15}H_{18}N_3O_3^+$ [M + H]$^+$ 288.13427 found 288.13395.

5-Fluoro-*N*^{2}-(prop-2-yn-1-yl)-*N*^{4}-(4-(trifluoromethyl)phenyl)pyrimidine-2,4-diamine (33)

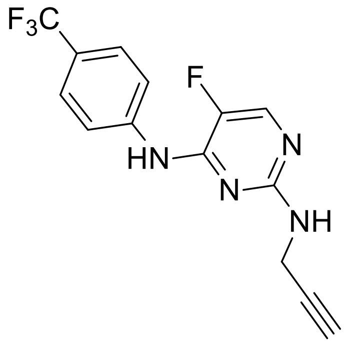

The reaction yield was 27%; brown crystals; m.p.: 155.3–160.1 °C; ^{1}H-NMR (500 MHz, $(CD_3)_2SO$) δ (ppm): 3.03 (s, 1H), 3.95-4.00 (m, 2H), 7.26 (t, 1H, *J* = 5.8 Hz), 7.63 (d, 2H, *J* = 8.6 Hz), 8.07 (d, 1H, *J* = 3.7 Hz), 8.14 (d, 2H, *J* = 8.5 Hz), 9.60 (s, 1H); ^{13}C-NMR (125 MHz, $(CD_3)_2SO$) δ (ppm): 31.3 (CH_2), 72.4 (CH), 82.9 (C_q), 120.4 (2 × CH_2), 122.7 (C_q, *J* = 31.9 Hz), 125.0 (C_q, *J* = 271.4 Hz), 126.0 (2 × CH, *J* = 3.7 Hz), 140.5 (C_q, *J* = 245.8 Hz), 142.1 (CH), 143.6 (C_q), 149.6 (C_q, *J* = 10.8 Hz), 158.0 (C_q); ^{19}F-NMR (470 MHz, $(CD_3)_2SO$) δ (ppm): –166.6, –60.1 ; HRMS (ESI+): m/z calcd. for $C_{14}H_{11}F_4N_4^+$ [M + H]$^+$ 311.09198; found 311.09080.

(3*R*, 3a*S*,5a*S*,9b*S*)-5a,9-Dimethyl-3-((4-phenyl-1*H*-1,2,3-triazol-1-yl)methyl)-3a,4,5,5a-tetrahydronaphtho[1,2-*b*]furan-2,8(3*H*,9b*H*)-dione (34)

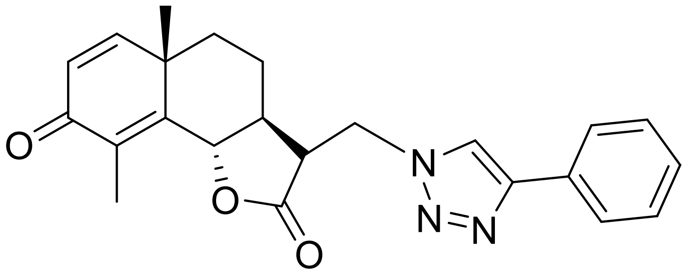

The reaction was implemented using phenylacetylene (**4**) according to the general procedures A and B. Yield: 33.0 mg (49%, Method A) and 48.0 mg (71%, Method B); yellowish white crystals; m.p.: 218.7–219.5 °C; $[\alpha]_D^{20}$ = –5 (c 0.148 MeOH); ^{1}H-NMR (500 MHz, $CDCl_3$) δ (ppm): 1.29 (s, 3H), 1.48 (dt, 1H, *J* = 4.3, 13.3 Hz), 1.67 (ddd, 1H, *J* = 3.4, 12.5, 25.4 Hz), 1.80-1.98 (m, 3H), 2.05 (s, 3H), 3.02 (dt, 1H, *J* = 5.0, 12.5 Hz), 4.75 (dd, 1H, *J* = 5.5, 14.5 Hz), 4.87 (dd, 1H, *J* = 4.5, 14.5 Hz), 4.88 (s, 1H), 6.23 (d, 1H, *J* = 9.9 Hz), 6.64 (d, 1H, *J* = 9.9 Hz), 7.34 (t, 1H, *J* = 7.3 Hz). 7.43 (t, 2H, *J* = 7.4 Hz), 7.82 (d, 2H, *J* = 7.8 Hz), 7.89 (s, 1H). ^{13}C-NMR (125 MHz, $CDCl_3$) δ (ppm): 10.9 (CH_3), 22.8 (CH_2), 25.2 (CH_3), 37.4 (CH_2), 41.1 (C_q), 47.1 (CH), 47.4 (CH_2), 49.4 (CH), 81.6 (CH), 120.6 (CH), 125.8 (2 × CH), 126.0 (CH), 128.5 (CH), 128.9 (2 × CH), 129.3 (C_q), 130.1 (C_q), 148.5 (C_q), 149.5 (C_q), 154.5 (CH), 174.0 (C_q), 185.9 (C_q). HRMS (ESI+): m/z calcd. for $C_{23}H_{24}N_3O_3^+$ $[M + H]^+$ 390.18122.; found 390.18062.

(3*R*,3a*S*,5a*S*,9b*S*)-3-((4-benzyl-1*H*-1,2,3-triazol-1-yl)methyl)-5a,9-dimethyl-3a,4,5,5a-tetrahydronaphtho[1,2-*b*]furan-2,8(3*H*,9b*H*)-dione (35)

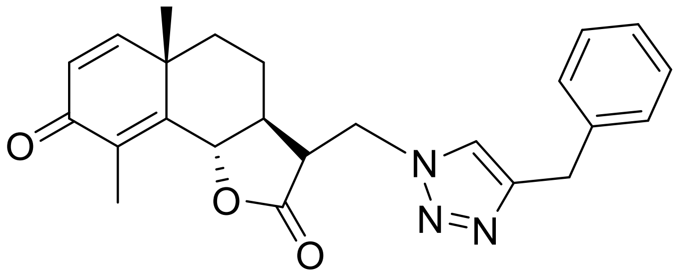

The reaction was implemented using benzylacetylene (**5**) according to the general procedure A. Yield: 37.3 mg (53%); white crystals; m.p.: 80.2–80.6 °C; $[\alpha]_D^{20}$ = –27 (c 0.152 MeOH); ^{1}H-NMR (500 MHz, $CDCl_3$) δ (ppm): 1.28 (s, 3H), 1.44 (dt, 1H, *J* = 3.5, 13.5 Hz), 1.61 (dd, 1H, *J* = 14.0, 24.4 Hz), 1.72-1.90 (m, 3H), 2.04 (s, 3H), 2.94 (dt, 1H, *J* = 4.8, 12.4 Hz), 4.07 (s, 2H), 4.62 (dd, 1H, *J* = 5.5, 12.5 Hz), 4.76-4.85 (m, 2H), 6.24 (d, 1H, *J* = 9.9 Hz), 6.65 (d, 1H, *J* = 10.0 Hz), 7.18-7.33 (m, 6H, overlapping with $CDCl_3$). ^{13}C-NMR (125 MHz, $CDCl_3$) δ (ppm): 10.8 (CH_3), 22.8 (CH_2), 25.1 (CH_3), 32.2 (CH_2), 37.4 (CH_2), 41.1 (C_q), 47.0 (CH), 47.2 (CH_2), 49.3 (CH), 81.5 (CH), 122.5 (CH), 126.0 (CH), 126.6 (CH), 128.5 (2 × CH), 128.7 (2 × CH), 129.2 (C_q), 138.8 (C_q), 148.5 (C_q), 149.6 (C_q), 154.6 (CH), 174.0 (C_q), 185.9 (C_q). HRMS (ESI+): m/z calcd. for $C_{24}H_{26}N_3O_3^+$ $[M + H]^+$ 404.19687; found 404.19625.

(3*R*,3a*S*,5a*S*,9b*S*)-3-((4-(4-methoxyphenyl)-1*H*-1,2,3-triazol-1-yl)methyl)-5a,9-dimethyl-3a,4,5,5a-tetrahydronaphtho[1,2-*b*]furan-2,8(3*H*,9b*H*)-dione (36)

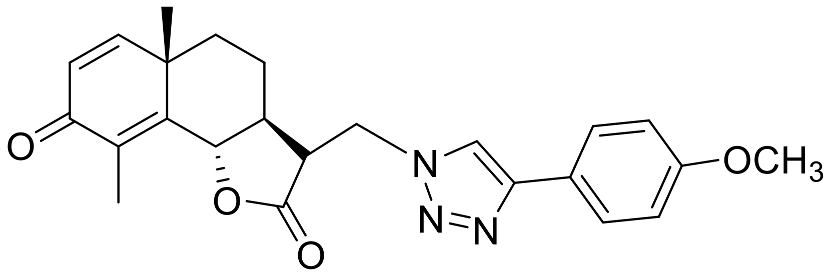

The reaction was implemented applying 1-ethynyl-4-methoxybenzene (**6**) according to the general procedure method A. Yield: 46.5 mg (64%); white crystals; m.p.: 191.6–191.8 °C; $[\alpha]_D^{20}$ = –4 (c 0.146 MeOH); ^{1}H-NMR (500 MHz, $CDCl_3$) δ (ppm): 1.29 (s, 3H), 1.48 (dt, 1H, *J* = 4.2, 13.5 Hz), 1.67 (ddd, 1H, *J* = 3.2, 12.5, 25.3 Hz), 1.78-1.99 (m, 3H), 2.05 (s, 3H), 3.01 (dt, 1H, *J* = 5.0, 12.3 Hz), 3.84 (s, 3H), 4.73 (dd, 1H, *J* = 5.5, 14.7 Hz), 4.83-4.91 (m, 2H), 6.23 (d, 1H, *J* = 9.9 Hz), 6.64 (d, 1H, *J* = 9.9 Hz), 6.96 (d, 2H, *J* = 8.1 Hz), 7.74 (d, 2H, *J* = 8.0 Hz), 7.80 (s, 1H). ^{13}C-NMR (125 MHz, $CDCl_3$) δ (ppm): 10.9 (CH_3), 22.8 (CH_2), 25.2 (CH_3), 37.4 (CH_2), 41.1 (C_q), 47.1 (CH), 47.3 (CH_2), 49.3 (CH), 55.4 (CH_3), 81.6 (CH), 114.3 (2 × CH), 119.8 (CH), 122.8 (C_q), 126.0 (CH), 127.1 (2 × CH), 129.3 (C_q), 148.3 (C_q), 149.5 (C_q), 154.6 (CH), 159.9 (C_q), 174.1 (C_q), 185.9 (C_q). HRMS (ESI+): m/z calcd. for $C_{24}H_{26}N_3O_4^+$ $[M + H]^+$ 420.19178; found 420.19111.

(3*R*,3a*S*,5a*S*,9b*S*)-5a,9-dimethyl-3-((4-(pyridin-2-yl)-1*H*-1,2,3-triazol-1-yl)methyl)-3a,4,5,5a-tetrahydronaphtho[1,2-*b*]furan-2,8(3*H*,9b*H*)-dione (37)

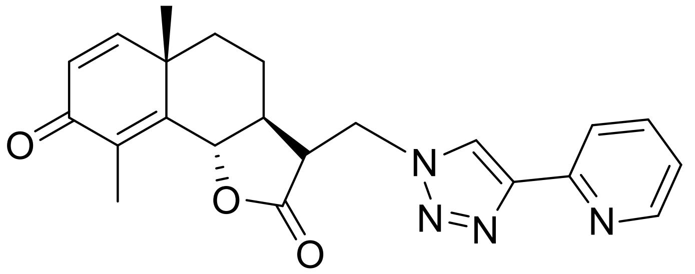

The reaction was implemented using 2-ethynylpyridine (**7**) according to the general procedure A. Yield: 46.0 mg (68%); pale brown crystals**;** m.p.: 189.5–191.0 °C; $[\alpha]_D^{20}$ = –16 (c 0.153 MeOH);^{1}H-NMR (500 MHz, $CDCl_3$) δ (ppm): 1.28 (s, 3H), 1.47 (dt, 1H, *J* = 4.6, 13.5 Hz), 1.63 (ddd, 1H, *J* = 3.8, 12.7, 23.8 Hz), 1.76-1.85 (m, 2H), 1.98 (ddd, 1H, *J* = 3.0, 11.8, 23.9 Hz) 2.07 (s, 3H), 3.08 (dt, 1H, *J* = 5.3, 12.4 Hz), 4.73 (dd, 1H, *J* = 6.6, 14.5 Hz), 4.86 (d, 1H, *J* = 11.4 Hz), 4.94 (dd, 1H, *J* = 4.4, 14.5 Hz), 6.23 (d, 1H, *J* = 9.9 Hz), 6.64 (d, 1H, *J* = 9.9 Hz), 7.25 (t, 1H, *J* = 6.2 Hz, overlapping with $CDCl_3$), 7.78 (t, 1H, *J* = 7.7 Hz), 8.13 (d, 1H, *J* = 7.9 Hz), 8.24 (s, 1H), 8.59 (d, 1H, *J* = 4.6 Hz). ^{13}C-NMR (125 MHz, $CDCl_3$) δ (ppm): 10.9 (CH_3), 22.9 (CH_2), 25.2 (CH_3), 37.4 (CH_2), 41.1 (C_q), 47.0 (CH), 48.0 (CH_2), 49.9 (CH), 81.5 (CH), 120.3 (CH), 123.0 (CH), 123.1 (CH), 126.0 (CH), 129.3 (C_q), 136.9 (CH), 149.1 (C_q), 149.5 (C_q), 149.6 (CH), 149.8 (C_q), 154.6 (CH), 173.6 (C_q), 185.9 (C_q). HRMS (ESI+): m/z calcd. for $C_{22}H_{23}N_4O_3^+$ $[M + H]^+$ 391.17647; found 391.17592.

1-((1-(((3*R*,3a*S*,5a*S*,9b*S*)-5a,9-dimethyl-2,8-dioxo-2,3,3a,4,5,5a,8,9b-octahydronaphtho[1,2-*b*]furan-3-yl)methyl)-1*H*-1,2,3-triazol-4-yl)methyl)pyrimidine-2,4(1*H*,3*H*)-dione (38)

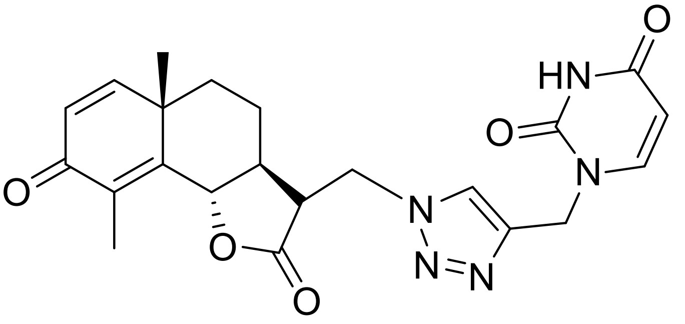

The reaction was implemented using 1-propargyluracil (**8**) according to the general procedure A. Yield: 51.4 mg (67%); white crystals; m.p.: 165.7–166.9 °C; $[\alpha]_D^{20}$ = –25 (c 0.150 MeOH); ^{1}H-NMR (500 MHz, $CDCl_3$) δ (ppm): 1.29 (s, 3H), 1.45 (dt, 1H, *J* = 4.3, 13.3 Hz), 1.64 (ddd, 1H, *J* = 3.2, 12.9, 25.6 Hz), 1.76-1.84 (m, 2H), 1.90 (dd, 1H, *J* = 12.4, 23.8 Hz), 2.06 (s, 3H), 3.02 (dt, 1H, *J* = 5.2, 12.5 Hz), 4.66 (dd, 1H, *J* = 6.0, 14.5 Hz), 4.80-4.88 (m, 2H), 4.90 (d, 1H, *J* = 14.5 Hz), 5.03 (d, 1H, *J* = 14.5 Hz), 5.70 (d, 1H, *J* = 7.9 Hz), 6.24 (d, 1H, *J* = 9.9 Hz), 6.65 (d, 1H, *J* = 9.8 Hz), 7.46 (d, 1H, *J* = 8.0 Hz), 7.84 (s, 1H), 8.55 (br s, 1H). ^{13}C-NMR (125 MHz, $CDCl_3$) δ (ppm): 10.9 (CH_3), 22.8 (CH_2), 25.1 (CH_3), 37.4 (CH_2), 41.1 (C_q), 43.2 (CH_2), 46.8 (CH), 47.7 (CH_2), 49.7 (CH), 81.5 (CH), 102.7 (CH), 124.8 (CH), 126.0 (CH), 129.3 (C_q), 142.3 (C_q), 144.2 (CH), 149.5 (C_q), 150.6 (C_q), 154.5 (CH), 163.0 (C_q), 173.7 (C_q), 185.9 (C_q). HRMS (ESI+): m/z calcd. for $C_{22}H_{24}N_5O_5^+$ $[M + H]^+$ 438.17775; found 438.17559.

1-((1-(((3*R*,3a*S*,5a*S*,9b*S*)-5a,9-dimethyl-2,8-dioxo-2,3,3a,4,5,5a,8,9b-octahydronaphtho[1,2-*b*]furan-3-yl)methyl)-1*H*-1,2,3-triazol-4-yl)methyl)-5-methylpyrimidine-2,4(1*H*,3*H*)-dione (39)

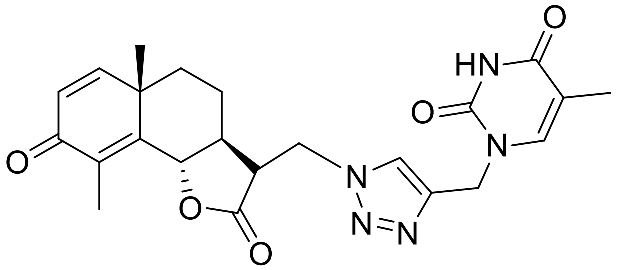

The reaction was implemented using 1-propargylthymine (**9**) according to the general procedure A. Yield: 59.0 mg (75%); white crystals; m.p.: 151.7–152.2 °C; $[\alpha]_D^{20}$ = –19 (c 0.149 MeOH); ^{1}H-NMR (500 MHz, CD_3OD) δ (ppm): 1.33 (s, 3H), 1.37 (dt, 1H, *J* = 4.3, 13.1 Hz), 1.43-1.50 (m, 1H), 1.68 (ddd, 1H, *J* = 3.3, 13.0, 25.8 Hz), 1.84-1.90 (m, 4H), 1.97 (ddd, 1H, *J* = 3.0, 12.1, 24.0 Hz), 2.05 (s, 3H), 3.36 (m, 2H, overlapping with water), 4.74 (dd, 1H, *J* = 6.8, 6.8 Hz), 4.87-4.89 (m, 1H, overlapping with CD_3OD), 5.00 (s, 2H), 5.10 (d, 1H, *J* = 11.4 Hz), 6.21 (d, 1H, *J* = 9.9 Hz), 6.90 (d, 1H, *J* = 9.8 Hz), 7.54 (s, 1H), 8.04 (s, 1H). ^{13}C-NMR (125 MHz, CD_3OD) δ (ppm): 9.5 (CH_3), 10.8 (CH_3), 21.9 (CH_2), 23.7 (CH_3), 37.4 (CH_2), 41.5 (C_q), 42.4 (CH_2), 45.9 (CH), 47.8 (CH_2, overlapping with CD_3OD), 49.9 (CH), 81.2 (CH), 110.2 (C_q), 124.6 (CH), 124.8 (CH), 127.6 (C_q), 141.2 (CH), 143.0 (C_q), 151.2 (C_q), 153.0 (C_q), 156.8 (CH), 165.4 (C_q), 174.5 (C_q), 186.9 (C_q). HRMS (ESI+): m/z calcd. for $C_{23}H_{26}N_5O_5^+$ $[M + H]^+$ 452.19285; found 452.19228.

1-((1-(((3*R*,3a*S*,5a*S*,9b*S*)-5a,9-dimethyl-2,8-dioxo-2,3,3a,4,5,5a,8,9b-octahydronaphtho[1,2-*b*]furan-3-yl)methyl)-1*H*-1,2,3-triazol-4-yl)methyl)-5-fluoropyrimidine-2,4(1*H*,3*H*)-dione (40)

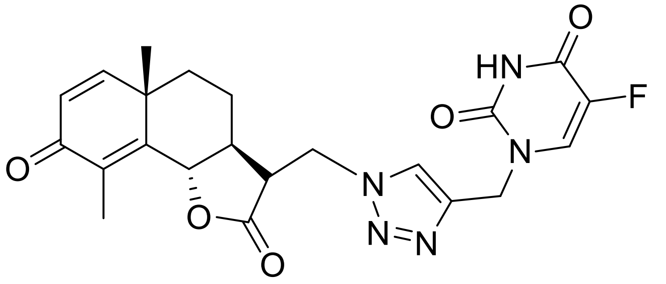

The reaction was implemented using 5-fluoro-1-propargyluracil (**10**) according to the general procedure A. Yield: 35.0 mg (44%); white crystals; m.p.: 153.6–154.7 °C, $[\alpha]_D^{20}$ = –17 (c 0.155 MeOH); ^{1}H-NMR (500 MHz, CD_3OD) δ (ppm): 1.21 (s, 3H), 1.27 (dt, 1H, *J* = 4.3, 13.6 Hz), 1.37 (d, 1H, *J* = 13.2 Hz), 1.58 (ddd, 1H, *J* = 3.3, 12.7, 25.6 Hz), 1.73-1.79 (m, 1H), 1.87 (ddd, 1H, *J* = 2.8, 1.6, 23.8 Hz), 1.94 (s, 3H), 3.18-3.26 (m, 2H, overlapping with water) 4.62 (dd, 1H, *J* = 7.0, 14.7 Hz), 4.68-4.80 (m, 1H, overlapping with MeOH), 4.87 (s, 2H), 4.99 (d, 1H, *J* = 11.5 Hz), 6.09 (d, 1H, *J* = 9.9 Hz), 6.78 (d, 1H, *J* = 9.8 Hz), 7.81 (d, 1H, *J* = 6.1 Hz), 7.95 (s, 1H). ^{13}C-NMR (125 MHz, CD_3OD) δ (ppm): 9.5 (CH_3), 21.9 (CH_2), 23.7 (CH_3), 37.3 (CH_2), 41.5 (C_q), 42.7 (CH_2), 45.9 (CH), 47.9 (CH_2), 50.0 (CH), 81.2 (CH), 124.6 (CH), 124.9 (CH), 127.6 (C_q), 129.3 (CH, *J* = 33.2 Hz), 140.3 (C_q , *J* = 233.7 Hz), 142.5 (C_q), 149.9 (C_q), 153.0 (C_q), 156.8 (CH), 158.4 (C_q), 174.5 (C_q), 186.9 (C_q). ^{19}F-NMR (470 MHz, $CDCl_3$) δ (ppm): –169.8. HRMS (ESI+): m/z calcd. for $C_{22}H_{23}FN_5O_5^+$ $[M + H]^+$ 456.16777; found 456.16715.

1-((1-(((3*R*,3a*S*,5a*S*,9b*S*)-5a,9-dimethyl-2,8-dioxo-2,3,3a,4,5,5a,8,9b-octahydronaphtho[1,2-*b*]furan-3-yl)methyl)-1*H*-1,2,3-triazol-4-yl)methyl)-5-iodopyrimidine-2,4(1*H*,3*H*)-dione (41)

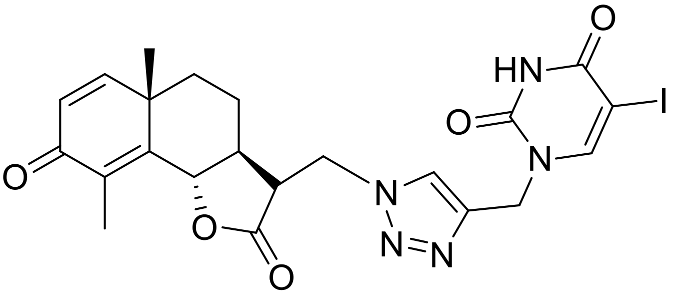

The reaction was implemented using 5-iodo-1-propargyluracil (**11**) according to the general procedure A. Yield: 55.0 mg (56%); white crystals; m.p.: 169.3–172.0 °C; $[\alpha]_D^{20}$ = + 4 (c 0.150 MeOH); ^{1}H-NMR (500 MHz, CD_3OD) δ (ppm): 1.30 (s, 3H), 1.47 (dt, 1H, *J* = 4.0, 14.1 Hz), 1.66 (dd, 1H, *J* = 12.1, 25.4 Hz), 1.83 (d, 2H, *J* = 11.6 Hz), 1.91 (dd, 1H, *J* = 11.5, 23.4 Hz), 2.07 (s, 3H), 3.03 (dt, 1H, *J* = 5.2, 12.5 Hz), 4.68 (dd, 1H, J = 5.9, 14.5 Hz), 4.79-4.95 (m, 3H), 4.99-5.08 (m, 1H), 6.25 (d, 1H, *J* = 9.9 Hz), 6.66 (d, 1H, *J* = 9.9 Hz), 7.90 (d, 2H, *J* = 24.1 Hz), 8.80 (br s, 1H). ^{13}C-NMR (125 MHz, $CDCl_3$) δ (ppm): 10.9 (CH_3), 22.8 (CH_2), 25.1 (CH_3), 37.4 (CH_2), 41.1 (C_q), 43.4 (CH_2), 46.8 (CH), 47.7 (CH_2), 49.7 (CH), 68.4 (C_q), 81.5 (CH), 125.0 (CH), 126.0 (CH), 129.3 (C_q), 141.9 (C_q), 148.6 (CH), 149.5 (C_q), 150.3 (C_q), 154.6 (CH), 160.1 (C_q), 173.7 (C_q), 185.9 (C_q). HRMS (ESI+): m/z calcd. for $C_{22}H_{23}IN_5O_5^+$ $[M + H]^+$ 564.07384; found 564.07274.

7-((1-(((3*R*,3a*S*,5a*S*,9b*S*)-5a,9-dimethyl-2,8-dioxo-2,3,3a,4,5,5a,8,9b-octahydronaphtho[1,2-*b*]furan-3-yl)methyl)-1*H*-1,2,3-triazol-4-yl)methyl)-1,3-dimethyl-1*H*-purine-2,6(3*H*,7*H*)-dione (42)

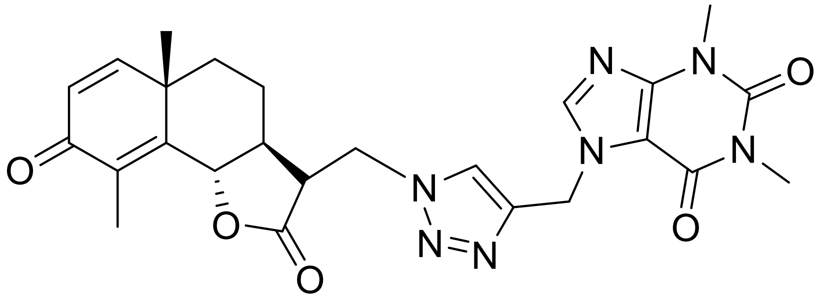

The reaction was implemented using propargyl theophylline (**12**) according to the general procedure A. Yield: 35.0 mg (40%); yellow crystals; m.p.: 150.1–151.2 °C; $[\alpha]_D^{20}$ = –25 (c 0.150 MeOH); ^{1}H-NMR (500 MHz, $CDCl_3$) δ (ppm): 1.29 (s, 3H), 1.44 (dt, 1H, *J* = 4.0, 15.9 Hz), 1.55-1.67 (m, 1H, overlapping with water), 1.74-1.82 (m, 2H), 1.90 (ddd, 1H, J = 2.8, 12.3, 24.1 Hz), 2.05 (s, 3H), 3.00 (dt, 1H, J = 5.3, 12.5 Hz), 3.40 (s, 3H), 3.57 (s, 3H), 4.64 (dd, 1H, *J* = 6.2, 14.5 Hz), 4.78-4.86 (m, 2H), 5.57 (q, 2H, *J* = 15.0 Hz), 6.25 (d, 1H, *J* = 9.9 Hz), 6.65 (d, 1H, *J* = 9.9 Hz), 7.78 (s, 1H), 7.94 (s, 1H). ^{13}C-NMR (125 MHz, $CDCl_3$) δ (ppm): 10.8 (CH_3), 22.8 (CH_2), 25.1 (CH_3), 28.0 (CH_3), 29.8 (CH_3), 37.4 (CH_2), 41.1 (C_q), 41.5 (CH_2), 46.9 (CH), 47.8 (CH_2), 49.7 (CH), 81.5 (CH), 106.5 (C_q), 124.8 (CH), 126.0 (CH), 129.3 (C_q), 141.3 (CH), 142.8 (C_q), 149.0 (C_q), 149.4 (C_q), 151.6 (C_q), 154.5 (CH), 155.4 (C_q), 173.5 (C_q), 185.9 (C_q). HRMS (ESI+): m/z calcd. for $C_{25}H_{28}N_7O_5^+$ $[M + H]^+$ 506.21464; found 506.21381.

(3*R*,3a*S*,5a*S*,9b*S*)-3-((4-((6-amino-9*H*-purin-9-yl)methyl)-1*H*-1,2,3-triazol-1-yl)methyl)-5a,9-dimethyl-3a,4,5,5a-tetrahydronaphtho[1,2-*b*]furan-2,8(3*H*,9b*H*)-dione (43)

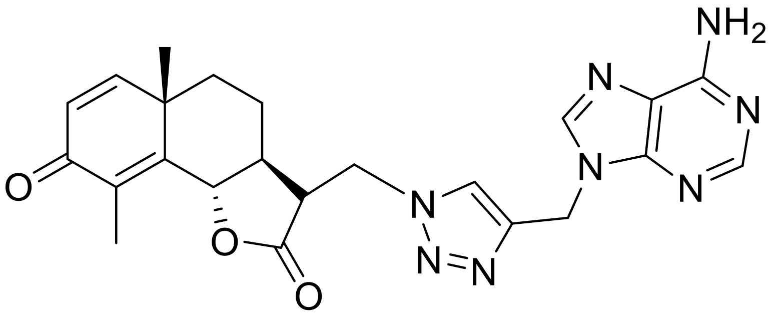

The reaction was implemented using 9-propargyl adenine (**13**) according to the general procedure A. Yield: 40.0 mg (50%); white crystals; m.p.: 162.7–164.1 °C; $[\alpha]_D^{20}$ = –19 (c 0.150 MeOH); ^{1}H-NMR (500 MHz, CD_3OD) δ (ppm): 1.25 (dt, 1H, *J* = 3.8, 13.6 Hz), 1.29 (s, 3H), 1.31-1.37 (m, 1H), 1.59 (ddd, 1H, *J* = 3.3, 13.3, 25.7 Hz), 1.77 (d, 1H, *J* = 13.6 Hz), 1.90 (ddd, 1H, *J* = 3.0, 12.1, 24.0 Hz), 2.00 (s, 3H), 3.26-3.31 (m, 1H), 4.72 (dd, 1H, *J* = 7.1, 14.5 Hz), 4.88 (d, 1H, *J* = 5.1 Hz), 5.06 (d, 1H, *J* = 11.4 Hz), 5.55 (s, 2H), 6.20 (d, 1H, *J* = 9.8 Hz), 6.87 (d, 1H, *J* = 9.9 Hz), 8.10 (s, 1H), 8.21 (d, 2H, *J* = 3.2 Hz). ^{13}C-NMR (125 MHz, $(CD_3)_2SO$) δ (ppm): 10.9 (CH_3), 22.0 (CH_2), 24.8 (CH_3), 37.6 (CH_2), 38.5 (CH_2), 41.6 (C_q), 45.8 (CH), 48.0 (CH_2), 50.0 (CH), 80.9 (CH), 119.1 (C_q), 124.9 (CH), 125.1 (CH), 126.9 (C_q), 141.0 (CH), 143.3 (C_q), 149.7 (C_q), 152.9 (C_q), 153.0 (CH), 156.4 (C_q), 156.7 (CH), 174.8 (C_q), 185.8 (C_q). HRMS (ESI+): m/z calcd. for $C_{23}H_{25}N_8O_3^+$ $[M + H]^+$ 461.20441; found 461.20364.

1-((1-(((3*R*,3a*S*,5a*S*,9b*S*)-5a,9-dimethyl-2,8-dioxo-2,3,3a,4,5,5a,8,9b-octahydronaphtho[1,2-*b*]furan-3-yl)methyl)-1*H*-1,2,3-triazol-4-yl)methyl)indoline-2,3-dione (44)

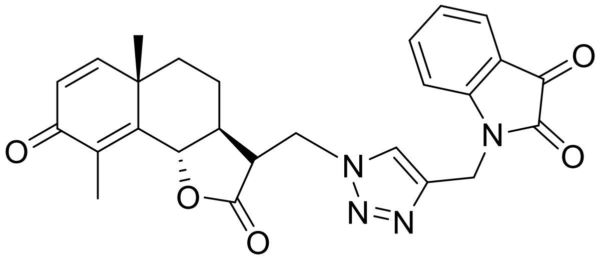

The reaction was implemented using **14** according to the general procedure A. Yield: 72.6 mg (40%); orange crystals; m.p.: 100.6–103.1 °C; $[\alpha]_D^{20}$ = –18 (c 0.154 MeOH); ^{1}H-NMR (500 MHz, $CDCl_3$) δ (ppm): 1.28 (s, 3H), 1.43 (dt, 1H, *J* = 4.3, 14.4 Hz), 1.61 (ddd, 1H, *J* = 3.0, 12.8 Hz, overlapping with water), 1.74-1.81 (m, 2H), 1.86 (dd, 1H, *J* = 11.7, 24.0 Hz), 2.02 (s, 3H), 2.99 (dt, 1H, *J* = 5.4, 12.5 Hz), 4.65 (dd, 1H, *J* = 6.0, 14.5 Hz), 4.76-4.86 (m, 2H), 5.02 (q, 2H, *J* = 15.7, 29.7 Hz), 6.24 (d, 1H, *J* = 9.9 Hz), 6.64 (d, 1H, *J* = 9.9 Hz), 7.11 (t, 1H, *J* = 7.5 Hz), 7.19 (d, 1H, *J* = 8.0), 7.55 (t, 1H, *J* = 7.8 Hz), 7.59 (d, 1H, *J* = 7.4 Hz), 7.76 (s, 1H). ^{13}C-NMR (125 MHz, $CDCl_3$) δ (ppm): 10.9 (CH_3), 22.8 (CH_2), 25.1 (CH_3), 35.4 (CH_2), 37.4 (CH_2), 41.1 (C_q), 46.9 (CH), 47.7 (CH_2), 49.6 (CH), 81.4 (CH), 111.2 (CH), 117.6 (C_q), 124.1 (CH), 124.1 (CH), 125.4 (CH), 126.0 (CH), 129.2 (C_q), 138.5 (CH), 142.3 (C_q), 149.5 (C_q), 150.1 (C_q), 154.5 (CH), 157.9 (C_q), 173.6 (C_q), 182.9 (C_q), 185.9 (C_q) HRMS (ESI+): m/z calcd. for $C_{26}H_{25}N_4O_5^+$ $[M + H]^+$ 473.18249; found 473.18210.

(3*R*,3a*S*,5a*S*,9b*S*)-3-((4-((1*H*-indol-1-yl)methyl)-1*H*-1,2,3-triazol-1-yl)methyl)-5a,9-dimethyl-3a,4,5,5a-tetrahydronaphtho[1,2-*b*]furan-2,8(3*H*,9b*H*)-dione (45)

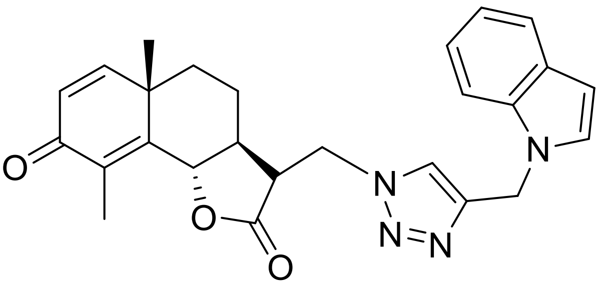

The reaction was implemented using **15** according to the general procedure A. Yield: 48.6 mg (63%); yellowish white crystals; m.p.: 101.5–101.7 °C; $[\alpha]_D^{20}$ = –30 (c 0.154 MeOH); ^{1}H-NMR (500 MHz, $CDCl_3$) δ (ppm): 1.26 (s, 3H), 1.36 (dt, 1H, *J* = 4.2, 13.3 Hz), 1.51 (ddd, 1H, *J* = 3.3, 12.7, 25.3 Hz, overlapping with water), 1.64-1.82 (m, 3H), 2.03 (s, 3H), 2.96 (dt, 1H, *J* = 5.3, 12.5 Hz), 4.54 (dd, 1H, *J* = 6.0, 14.5 Hz), 4.74 (dd, 1H, *J* = 4.6, 14.6 Hz), 4.78 (d, 1H, *J* = 11.3 Hz), 5.43 (s, 2H), 6.24 (d, 1H, *J* = 9.9 Hz), 6.52 (d, 1H, *J* = 2.9 Hz), 6.64 (d, 1H, *J* = 9.9 Hz), 7.10 (t, 1H, *J* = 7.5 Hz), 7.15-7.19 (m, 2H), 7.31-7.35 (m, 2H), 7.62 (d, 1H, *J* = 7.9 Hz).^{13}C-NMR (125 MHz, $CDCl_3$) δ (ppm): 10.9 (CH_3), 22.7 (CH_2), 25.1 (CH_3), 37.3 (CH_2), 41.1 (C_q), 42.0 (CH_2), 46.9 (CH), 47.5 (CH_2), 49.5 (CH), 81.5 (CH), 102.3 (CH), 109.4 (CH), 119.8 (CH), 121.2 (CH), 121.9 (CH), 122.8 (CH), 126.0 (CH), 127.7 (CH), 128.9 (C_q), 129.2 (C_q), 135.8 (C_q), 145.4 (C_q), 149.4 (C_q), 154.5 (CH), 173.7 (C_q), 185.9 (C_q). HRMS (ESI+): m/z calcd. for $C_{26}H_{27}N_4O_3^+$ $[M + H]^+$ 443.20831; found 443.20750.

(3*R*,3a*S*,5a*S*,9b*S*)-3-((4-((1*H*-benzo[d]imidazol-1-yl)methyl)-1*H*-1,2,3-triazol-1-yl)methyl)-5a,9-dimethyl-3a,4,5,5a-tetrahydronaphtho[1,2-*b*]furan-2,8(3*H*,9b*H*)-dione (46)

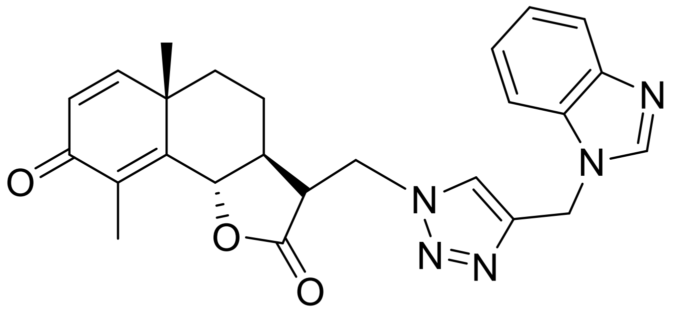

The reaction was implemented using **16** according to the general procedure A. Yield: 37.7 mg (49%); white crystals; m.p.: 122.8–125.1 °C; $[\alpha]_D^{20}$ = –2 (c 0.150 MeOH); ^{1}H-NMR (500 MHz, $CDCl_3$) δ (ppm): 1.27 (s, 3H), 1.39 (dt, 1H, *J* = 4.5, 14.1 Hz), 1.58 (dd, 1H, overlapping with water), 1.73-1.85 (m, 3H), 2.02 (s, 3H), 2.92 (dt, 1H, *J* = 4.9, 12.4 Hz), 4.62 (dd, 1H, *J* = 5.4, 14.5 Hz), 4.73-4.84 (m, 2H), 5.48 (s, 2H), 6.24 (d, 1H, *J* = 9.9 Hz), 6.64 (d, 1H, *J* = 9.9 Hz), 7.28 (d, 2H, overlapping with $CDCl_3$), 7.41 (d, 1H, *J* = 7.6 Hz), 7.53 (s, 1H), 7.80 (d, 1H, *J* = 6.8 Hz), 8.01 (s, 1H). ^{13}C-NMR (125 MHz, $CDCl_3$) δ (ppm): 10.9 (CH_3), 22.7 (CH_2), 25.1 (CH_3), 37.3 (CH_2), 40.4 (CH_2), 41.1 (C_q), 46.9 (CH), 47.4 (CH_2), 49.3 (CH), 81.5 (CH), 109.7 (CH), 120.6 (CH), 122.5 (CH), 123.3 (CH), 123.4 (CH), 126.0 (CH), 129.3 (C_q), 133.5 (C_q), 142.8 (CH), 143.5 (C_q), 144.0 (C_q), 149.3 (C_q), 154.5 (CH), 173.7 (C_q), 185.9 (C_q); HRMS (ESI+): m/z calcd. for $C_{25}H_{26}N_5O_3^+$ $[M + H]^+$ 444.20357; found 444.20370.

(3*R*,3a*S*,5a*S*,9b*S*)-3-((4-((1*H*-indazol-1-yl)methyl)-1*H*-1,2,3-triazol-1-yl)methyl)-5a,9-dimethyl-3a,4,5,5a-tetrahydronaphtho[1,2-*b*]furan-2,8(3*H*,9b*H*)-dione (47)

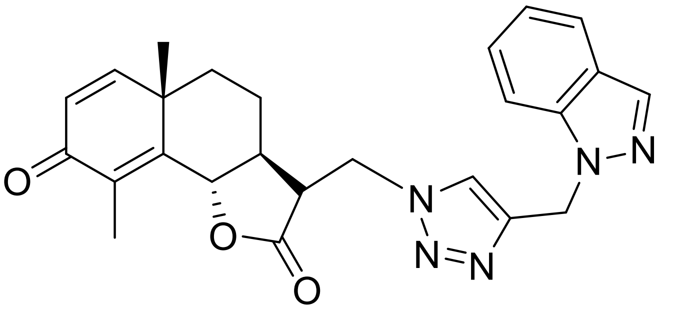

The reaction was implemented using **17** according to the general procedure A. Yield: 56.3 mg (73%); brown crystals; m.p.: 82.9–83.0 °C; $[\alpha]_D^{20}$ = –22 (c 0.155 MeOH); ^{1}H-NMR (500 MHz, $CDCl_3$) δ (ppm): 1.25 (s, 3H), 1.35 (dt, 1H, *J* = 2.9, 13.2 Hz), 1.51 (dd, 1H, *J* = 11.8, 24.6 Hz), 1.63-1.82 (m, 3H), 2.01 (s, 3H), 2.93 (dt, 1H, *J* = 5.8, 12.3 Hz), 4.56 (dd, 1H, *J* = 6.1, 14.5 Hz), 4.74-4.81 (m, 2H), 5.70 (s, 2H), 6.23 (d, 1H, *J* = 9.9 Hz), 6.62 (d, 1H, *J* = 9.9 Hz), 7.14 (t, 1H, *J* = 7.5 Hz), 7.36 (t, 1H, *J* = 7.6 Hz), 7.48-7.53 (m, 2H), 7.72 (d, 1H, *J* = 8.1 Hz), 8.00 (s, 1H). ^{13}C-NMR (125 MHz, $CDCl_3$) δ (ppm): 10.8 (CH_3), 22.7 (CH_2), 25.1 (CH_3), 37.3 (CH_2), 41.1 (C_q), 44.6 (CH_2), 46.9 (CH), 47.6 (CH_2), 49.5 (CH), 81.4 (CH), 109.3 (CH), 121.0 (CH), 121.2 (CH), 123.4 (CH), 124.4 (C_q), 126.0 (CH), 126.6 (CH), 129.2 (C_q), 133.8 (CH), 139.4 (C_q), 144.6 (C_q), 149.4 (C_q), 154.5 (CH), 173.7 (C_q), 185.9 (C_q). HRMS (ESI+): m/z calcd. for $C_{25}H_{26}N_5O_3^+$ $[M + H]^+$ 444.20357; found 444.20322.

(3*R*,3a*S*,5a*S*,9b*S*)-3-((4-((1*H*-benzo[*d*][1,2,3]triazol-1-yl)methyl)-1*H*-1,2,3-triazol-1-yl)methyl)-5a,9-dimethyl-3a,4,5,5a-tetrahydronaphtho[1,2-*b*]furan-2,8(3*H*,9b*H*)-dione (48)

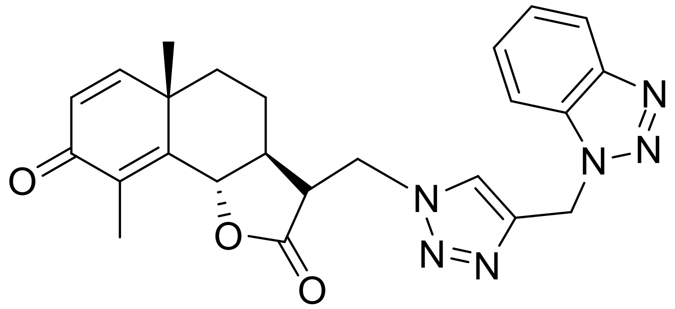

The reaction was implemented using **18** according to the general procedure A. Yield: 54.0 mg (70%); yellowish white crystals; m.p.: 102.4–103.8 °C; $[\alpha]_D^{20}$ = –16 (c 0.150 MeOH); ^{1}H-NMR (500 MHz, $CDCl_3$) δ (ppm): 1.26 (s, 3H), 1.39 (dt, 1H, *J* = 3.4, 13.5 Hz), 1.56 (dd, 1H, *J* = 12.5 Hz, overlapping with water), 1.69-1.77 (m, 2H), 1.81 (dd, 1H, *J* = 11.8, 23.7 Hz), 2.00 (s, 3H), 2.96 (dt, 1H, *J* = 5.2, 12.4 Hz), 4.62 (dd, 1H, *J* = 5.9, 14.5 Hz), 4.75-4.84 (m, 2H), 5.96 (s, 2H), 6.24 (d, 1H, *J* = 9.9 Hz), 6.64 (d, 1H, *J* = 9.9 Hz), 7.36 (t, 1H, *J* = 7.4 Hz), 7.46 (t, 1H, *J* = 7.4 Hz), 7.66 (d, 1H, *J* = 8.3 Hz), 7.68 (s, 1H), 8.04 (d, 1H, *J* = 8.3 Hz). ^{13}C-NMR (125 MHz, $CDCl_3$) δ (ppm): 10.8 (CH_3), 22.7 (CH_2), 25.1 (CH_3), 37.3 (CH_2), 41.1 (C_q), 43.6 (CH_2), 46.9 (CH), 47.6 (CH_2), 49.5 (CH), 81.4 (CH), 109.8 (CH), 120.0 (CH), 124.1 (CH), 124.2 (CH), 126.0 (CH), 127.7 (CH), 129.3 (C_q), 132.6 (C_q), 142.7 (C_q), 146.2 (C_q), 149.4 (C_q), 154.5 (CH), 173.6 (C_q), 185.9 (C_q). HRMS (ESI+): m/z calcd. for $C_{24}H_{25}N_6O_3^+$ $[M + H]^+$ 445.19881; found 445.19827.

(3*R*,3a*S*,5a*S*,9b*S*)-5a,9-dimethyl-3-((4-((pyrimidin-2-ylthio)methyl)-1*H*-1,2,3-triazol-1-yl)methyl)-3a,4,5,5a-tetrahydronaphtho[1,2-*b*]furan-2,8(3*H*,9b*H*)-dione (49)

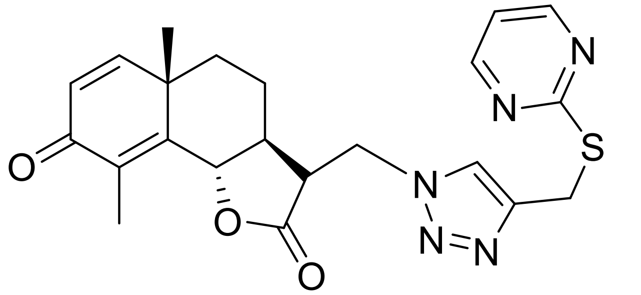

The reaction was implemented using **19** according to the general procedure A. Yield: 45.0 mg (59%); grey crystals; m.p.: 87.3-89.0 °C; $[\alpha]_D^{20}$ = –48 (c 0.154 MeOH); ^{1}H-NMR (500 MHz, $CDCl_3$) δ (ppm): 1.28 (s, 3H), 1.43 (dt, 1H, *J* = 3.2, 13.3 Hz), 1.55-1.66 (m, 1H), 1.76-1.86 (m, 3H), 2.01 (s, 3H), 2.95 (dt, 1H, *J* = 5.0, 12.3 Hz), 4.47 (dd, 2H, *J* = 14.6, 17.9 Hz), 4.62 (dd, 1H, *J* = 5.7, 14.5 Hz), 4.77-4.84 (m, 2H), 6.24 (d, 1H, *J* = 9.9 Hz), 6.64 (d, 1H, *J* = 9.9 Hz), 7.00 (t, 1H, *J* = 4.8 Hz), 7.67 (s, 1H), 8.54 (d, 2H, *J* = 4.8 Hz). ^{13}C-NMR (125 MHz, $CDCl_3$) δ (ppm): 10.8 (CH_3), 22.8 (CH_2), 25.1 (CH_3), 25.5 (CH_2), 37.4 (CH_2), 41.1 (C_q), 47.1 (CH), 47.3 (CH_2), 49.4 (CH), 81.5 (CH), 116.8 (CH), 123.6 (CH), 126.0 (CH), 129.2 (C_q), 145.9 (C_q), 149.5 (C_q), 154.6 (CH), 157.4 (2 × CH), 171.4 (C_q), 173.9 (C_q), 185.9 (C_q). HRMS (ESI+): m/z calcd. for $C_{22}H_{24}N_5O_3S^+$ $[M + H]^+$ 438.15999; found 438.16009.

(3*R*,3a*S*,5a*S*,9b*S*)-3-((4-((benzo[*d*]oxazol-2-ylthio)methyl)-1*H*-1,2,3-triazol-1-yl)methyl)-5a,9-dimethyl-3a,4,5,5a-tetrahydronaphtho[1,2-*b*]furan-2,8(3*H*,9b*H*)-dione (50)

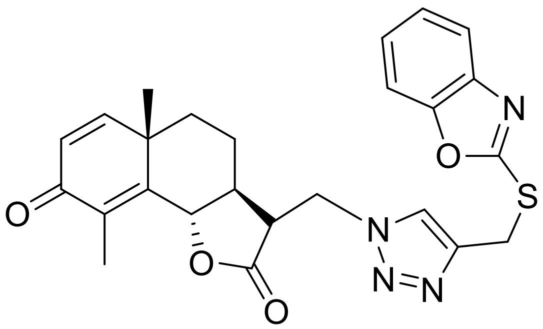

The reaction was implemented using **20** according to the general procedures A and B. Yield: 18.0 mg (22%, Method A) and 38.6 mg (47%, Method B); white crystals; m.p: 122.5–124.1 °C; $[\alpha]_D^{20}$ = –8 (c 0.117 MeOH); ^{1}H-NMR (500 MHz, $CDCl_3$) δ (ppm): 1.23 (s, 3H), 1.33 (dt, 1H, *J* = 4.6, 12.6 Hz), 1.50 (ddd, 1H, *J* = 3.7, 12.8, 25.8 Hz) 1.61-1.69 (m, 2H, overlapping with water), 1.84 (ddd, 1H, *J* = 3.4, 11.7, 24.0 Hz), 2.00 (s, 3H), 2.96 (dt, 1H, *J* = 5.5, 12.4 Hz), 4.57-4.63 (m, 3H), 4.76-4.84 (m, 2H), 6.21 (d, 1H, *J* = 9.9 Hz), 6.60 (d, 1H, *J* = 9.9 Hz), 7.23-7.33 (m, 2H, overlapping with $CDCl_3$), 7.44 (d, 1H, *J* = 7.9 Hz), 7.62 (d, 1H, *J* = 7.7 Hz), 7.84 (s, 1H). ^{13}C-NMR (125 MHz, $CDCl_3$) δ (ppm): 10.8 (CH_3), 22.7 (CH_2), 25.1 (CH_3), 26.6 (CH_2), 37.3 (CH_2), 41.1 (C_q), 47.0 (CH), 47.7 (CH_2), 49.7 (CH), 81.5 (CH), 110.0 (CH), 118.5 (CH), 124.1 (CH), 124.2 (CH), 124.5 (CH), 125.9 (CH), 129.2 (C_q), 141.8 (C_q), 144.4 (C_q), 149.5 (C_q), 152.1 (C_q), 154.5 (CH), 164.0 (C_q), 173.7 (C_q), 185.9 (C_q). HRMS (ESI+): m/z calcd. for $C_{25}H_{25}N_4O_4S^+$ $[M + H]^+$ 477.15965; found 477.15939.

(3*R*,3a*S*,5a*S*,9b*S*)-3-((4-((benzo[*d*]thiazol-2-ylthio)methyl)-1*H*-1,2,3-triazol-1-yl)methyl)-5a,9-dimethyl-3a,4,5,5a-tetrahydronaphtho[1,2-*b*]furan-2,8(3*H*,9b*H*)-dione (51)

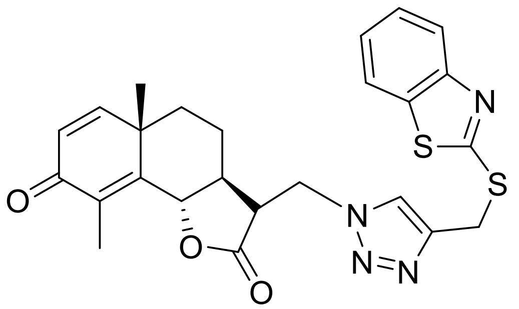

The reaction was implemented using **21** according to the general procedure A. Yield: 58.6 mg (68%); brown crystals; m.p.: 83.7–85.0 °C; $[\alpha]_D^{20}$ = –31 (c 0.138 MeOH); ^{1}H-NMR (500 MHz, $CDCl_3$) δ (ppm): 1.21 (s, 3H), 1.29 (dt, 1H, *J* = 4.2, 13.3 Hz), 1.45 (dd, 1H, *J* = 2.9, 12.8, 25.4 Hz,), 1.62 (d, 2H, *J* = 15.5 Hz, overlapping with water), 1.82 (ddd, 1H, *J* = 2.9, 12.0, 23.9 Hz), 2.00 (s, 3H), 2.94 (dt, 1H, *J* = 5.7, 12.5 Hz), 4.58 (dd, 1H, *J* = 6.6, 14.5 Hz), 4.66 (s, 2H), 4.76 (d, 1H, *J* = 11.4 Hz), 4.81 (dd, 1H, *J* = 4.4, 14.6 Hz), 6.21 (d, 1H, *J* = 9.9 Hz), 6.58 (d, 1H, *J* = 9.9 Hz), 7.31 (t, 1H, *J* = 7.4 Hz), 7.44 (t, 1H, *J* = 7.6 Hz), 7.74-7.78 (m, 2H), 7.90 (d, 1H, *J* = 8.1 Hz). ^{13}C-NMR (125 MHz, $CDCl_3$) δ (ppm): 10.8 (CH_3), 22.7 (CH_2), 25.0 (CH_3), 27.5 (CH_2), 37.3 (CH_2), 41.0 (C_q), 47.0 (CH), 47.8 (CH_2), 49.7 (CH), 81.5 (CH), 121.1 (CH), 121.6 (CH), 124.1 (CH), 124.5 (CH), 125.9 (CH), 126.2 (CH), 129.2 (C_q), 135.6 (C_q), 144.9 (C_q), 149.5 (C_q), 153.0 (C_q), 154.5 (CH), 165.4 (C_q), 173.7 (C_q), 185.9(C_q). HRMS (ESI+): m/z calcd. for $C_{25}H_{25}N_4O_3S_2^+$ $[M + H]^+$ 493.13681; found 493.13729.

(3*R*,3a*S*,5a*S*,9b*S*)-5a,9-dimethyl-3-((4-((quinolin-8-yloxy)methyl)-1*H*-1,2,3-triazol-1-yl)methyl)-3a,4,5,5a-tetrahydronaphtho[1,2-*b*]furan-2,8(3*H*,9b*H*)-dione (52)

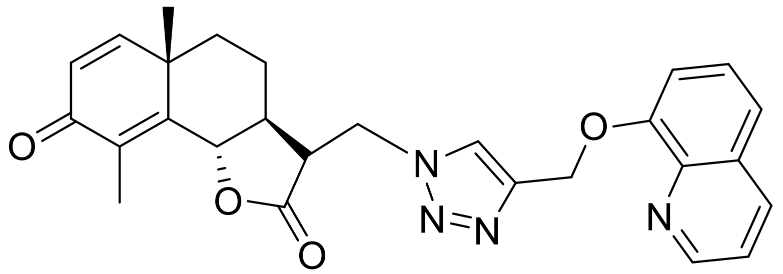

The reaction was implemented using **22** according to the general procedure A. Yield: 22.4 mg (47%); brown crystals; m.p.: 100.8–101.5 °C; $[\alpha]_D^{20}$ = –18 (c 0.115 MeOH); ^{1}H-NMR (500 MHz, $CDCl_3$) δ (ppm): 1.25 (s, 3H), 1.37 (dt, 1H, *J* = 4.1, 14.4 Hz), 1.48-1.60 (m, 1H, overlapping with water), 1.68-1.76 (m, 2H), 1.83 (dd, 1H, *J* = 12.7, 24.7 Hz), 2.00 (s, 3H), 2.97 (dt, 1H, *J* = 4.5, 12.6 Hz), 4.63 (dd, 1H, *J* = 6.2, 14.5 Hz), 4.78-4.87 (m, 2H), 5.53-5.61 (m, 2H), 6.24 (d, 1H, *J* = 9.8 Hz), 6.62 (d, 1H, *J* = 9.8 Hz), 7.21-7.29 (m, 1H, overlapping with $CDCl_3$), 7.37-7.47 (m, 3H), 7.85 (s, 1H), 8.14 (d, 1H, *J* = 8.2 Hz), 8.94 (s, 1H). ^{13}C-NMR (125 MHz, $CDCl_3$) δ (ppm): 10.8 (CH_3), 22.7 (CH_2), 25.1 (CH_3), 37.3 (CH_2), 41.1 (C_q), 46.9 (CH), 47.7 (CH_2), 49.6 (CH), 62.7 (CH_2), 81.5 (CH), 109.9 (CH), 120.5 (CH), 121.8 (CH), 124.3 (CH), 126.0 (CH), 126.5 (CH), 129.2 (C_q), 129.5 (C_q), 136.1 (CH), 140.3 (C_q), 144.9 (C_q), 149.5 (CH), 149.5 (C_q), 153.7 (C_q), 154.6 (CH), 173.7 (C_q), 186.0 (C_q). HRMS (ESI+): m/z calcd. for $C_{27}H_{27}N_4O_4^+$ $[M + H]^+$ 471.20268; found 471.20201.

(3*R*,3a*S*,5a*S*,9b*S*)-5a,9-dimethyl-3-((4-(((2-oxo-2*H*-chromen-6-yl)oxy)methyl)-1*H*-1,2,3-triazol-1-yl)methyl)-3a,4,5,5a-tetrahydronaphtho[1,2-*b*]furan-2,8(3*H*,9b*H*)-dione (53)

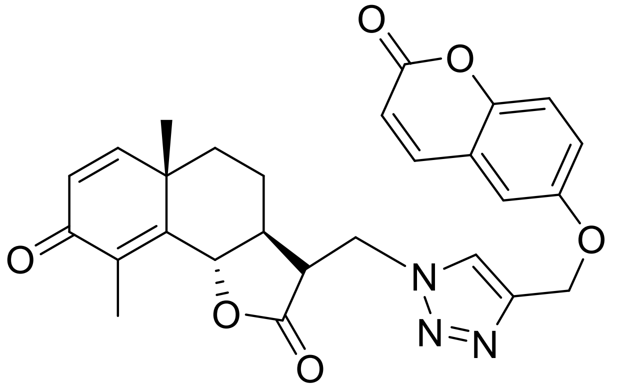

The reaction was implemented using **23** according to the general procedure A. Yield: 69.6 mg (82%); yellowish white crystals; m.p.: 98.5–101.3 °C; $[\alpha]_D^{20}$ = –5 (c 0.150 MeOH); ^{1}H-NMR (500 MHz, $CDCl_3$) δ (ppm): 1.29 (s, 3H), 1.45 (dt, 1H, *J* = 4.2, 13.4 Hz), 1.66 (dd, 1H, *J* = 3.3, 12.8, 25.5 Hz,), 1.76-1.86 (m, 2H), 1.92 (d, 1H, *J* = 12.8 Hz), 2.03 (s, 3H), 3.00 (dt, 1H, *J* = 4.9, 12.5 Hz), 4.73 (dd, 1H, *J* = 5.2, 14.5 Hz), 4.82-4.89 (m, 2H), 5.26 (s, 2H), 6.23 (d, 1H, *J* = 9.9 Hz), 6.27 (d, 1H, *J* = 9.4 Hz), 6.65 (d, 1H, *J* = 9.9 Hz), 6.89-6.93 (m, 2H), 7.38 (d, 1H, *J* = 9.2 Hz), 7.64 (d, 1H, *J* = 9.5 Hz), 7.79 (s, 1H). ^{13}C-NMR (125 MHz, $CDCl_3$) δ (ppm): 10.8 (CH_3), 22.8 (CH_2), 25.1 (CH_3), 37.4 (CH_2), 41.1 (C_q), 47.1 (CH), 47.3 (CH_2), 49.2 (CH), 62.2 (CH_2), 81.5 (CH), 102.0 (CH), 113.0 (CH), 113.1 (C_q), 113.6 (CH), 124.2 (CH), 126.0 (CH), 128.9 (CH), 129.2 (C_q), 143.3 (CH), 143.8 (C_q), 149.4 (C_q), 154.5 (CH), 155.7 (C_q), 161.0 (C_q), 161.2 (C_q), 173.9 (C_q), 185.9 (C_q). HRMS (ESI+): m/z calcd. for $C_{27}H_{26}N_3O_6^+$ $[M + H]^+$ 488.18216; found 488.18114.

(3*R*,3a*S*,5a*S*,9b*S*)-3-((4-(((2,5-dichloropyrimidin-4-yl)amino)methyl)-1*H*-1,2,3-triazol-1-yl)methyl)-5a,9-dimethyl-3a,4,5,5a-tetrahydronaphtho[1,2-*b*]furan-2,8(3*H*,9b*H*)-dione (54)

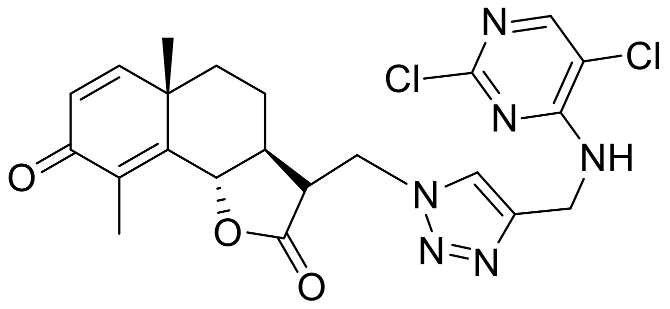

The reaction was implemented using **27** according to the general procedure A. Yield: 63.1 mg (74%); yellow crystals; m.p.: 108.8 –109.9 °C; $[\alpha]_D^{20}$ = –31 (c 0.137 MeOH); ^{1}H-NMR (500 MHz, $CDCl_3$) δ (ppm): 1.29 (s, 3H), 1.46 (dt, 1H, *J* = 3.9, 13.7 Hz), 1.63 (dd, 1H, *J* = 12.8, 25.6 Hz), 1.77-1.93 (m, 3H), 2.05 (s, 3H), 3.00 (dt, 1H, *J* = 5.0, 12.5 Hz), 4.66-4.87 (m, 5H), 6.16 (br s, 1H), 6.24 (d, 1H, *J* = 9.8 Hz), 6.65 (d, 1H, *J* = 9.9 Hz), 7.74 (s, 1H), 8.06 (s, 1H). ^{13}C-NMR (125 MHz, $CDCl_3$) δ (ppm): 10.9 (CH_3), 22.8 (CH_2), 25.1 (CH_3), 36.5 (CH_2) 37.4 (CH_2), 41.1 (C_q), 47.0 (CH), 47.6 (CH_2), 49.5 (CH), 81.5 (CH), 113.5 (C_q), 123.8 (CH), 126.0 (CH), 129.3 (C_q), 144.1 (C_q), 149.5 (C_q), 153.9 (CH), 154.5 (CH), 158.3 (C_q), 158.5 (C_q), 173.7 (C_q), 185.9 (C_q). HRMS (ESI+): m/z calcd. for $C_{22}H_{23}Cl_2N_6O_3^+$ $[M + H]^+$ 489.12032; found 489.11972.+-

(3*R*,3a*S*,5a*S*,9b*S*)-3-((4-(((2-chloro-5-fluoropyrimidin-4-yl)amino)methyl)-1*H*-1,2,3-triazol-1-yl)methyl)-5a,9-dimethyl-3a,4,5,5a-tetrahydronaphtho[1,2-*b*]furan-2,8(3*H*,9b*H*)-dione (55)

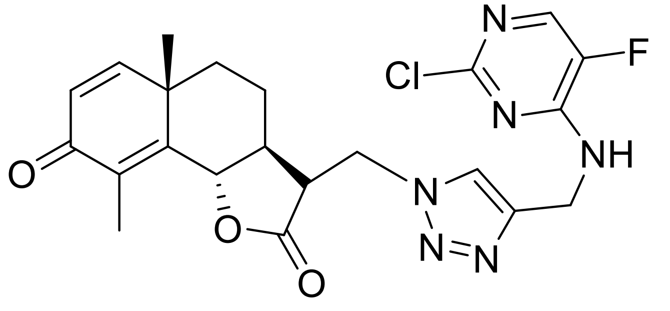

The reaction was implemented using **28** according to the general procedure A. Yield: 56.8 mg (69%); yellow crystals; m.p.: 98.5–98.9 °C; $[\alpha]_D^{20}$ = –30 (c 0.156 MeOH); ^{1}H-NMR (500 MHz, $CDCl_3$) δ (ppm): 1.29 (s, 3H), 1.46 (dt, 1H, *J* = 4.1, 13.5 Hz), 1.64 (dd, 1H, *J* = 13.0, 25.6 Hz), 1.77-1.94 (m, 3H), 2.05 (s, 3H), 3.00 (dt, 1H, *J* = 4.9, 12.7 Hz), 4.65-4.88 (m, 5H), 5.90 (br s, 1H), 6.24 (d, 1H, *J* = 10.0 Hz), 6.65 (d, 1H, *J* = 9.9 Hz), 7.74 (s, 1H), 7.92 (s, 1H). ^{13}C-NMR (125 MHz, $CDCl_3$) δ (ppm): 10.9 (CH_3), 22.8 (CH_2), 25.1 (CH_3), 36.0 (CH_2), 37.4 (CH_2), 41.1 (C_q), 47.0 (CH), 47.6 (CH_2), 49.5 (CH), 81.5 (CH), 123.8 (CH), 126.0 (CH), 129.3 (C_q), 140.1 (CH, *J* = 19.7 Hz), 144.1 (C_q), 145.4 (C_q, *J* = 258.0 Hz), 149.5 (C_q), 153.3 (C_q, *J* = 12.8 Hz), 154.4 (C_q), 154.6 (CH), 173.8 (C_q), 185.9 (C_q). ^{19}F-NMR (470 MHz, $CDCl_3$) δ (ppm): –159.2; HRMS (ESI+): m/z calcd. for $C_{22}H_{23}ClFN_6O_3^+$ $[M + H]^+$ 473.14987; found 473.14924.

(3*R*,3a*S*,5a*S*,9b*S*)-3-((4-(((2-chloro-5-(trifluoromethyl)pyrimidin-4-yl)amino)methyl)-1*H*-1,2,3-triazol-1-yl)methyl)-5a,9-dimethyl-3a,4,5,5a-tetrahydronaphtho[1,2-*b*]furan-2,8(3*H*,9b*H*)-dione (56)

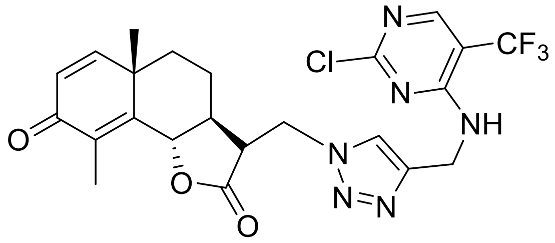

The reaction was implemented using **29** according to the general procedures A and B. Yield: 37.0 mg (41%, Method A) and 44.0 mg (48%, Method B); yellowish white crystals; m.p.: 96.7–97.2 °C; $[\alpha]_D^{20}$ = –40 (c 0.148 MeOH); ^{1}H-NMR (500 MHz, $CDCl_3$) δ (ppm): 1.29 (s, 3H), 1.45 (dt, 1H, *J* = 3.3, 13.3 Hz), 1.58-1.70 (m, 1H), 1.76-1.93 (m, 3H), 2.04 (s, 3H), 2.99 (br s, 1H), 4.67 (dd, 1H, *J* = 5.0, 14.5 Hz) 4.72-4.88 (m, 4H), 6.23 (d, 1H, *J* = 9.9 Hz), 6.39 (d, 1H, *J* = 71.6), 6.65 (d, 1H, *J* = 9.9 Hz), 7.70 (d, 1H, *J* = 11.5 Hz), 8.46 (d, 1H, *J* = 35.1 Hz). ^{13}C-NMR (125 MHz, $CDCl_3$) δ (ppm): 10.9 (CH_3), 22.8 (CH_2), 25.1 (CH_3), 37.0 (CH_2), 37.4 (CH_2), 41.1 (C_q), 47.0 (CH), 47.4 (CH_2), 49.4 (CH), 81.5 (CH), 123.1 (C_q), 123.4 (CH), 123.8 (C_q), 126.0 (CH), 129.3 (C_q), 149.5 (C_q), 150.2 (C_q), 151.5 (C_q) 154.6 (CH), 157.5 (CH), 173.9 (C_q), 185.9 (C_q) ^{19}F-NMR (470 MHz, $CDCl_3$) δ (ppm): –61.6; HRMS (ESI+): m/z calcd. for $C_{23}H_{23}ClF_3N_6O_3^+$ $[M + H]^+$ 523.14668; found 523.14595.

(3*R*,3a*S*,5a*S*,9b*S*)-3-((4-(((5-chloro-2-((4-(trifluoromethyl)phenyl)amino)pyrimidin-4-yl)amino)methyl)-1*H*-1,2,3-triazol-1-yl)methyl)-5a,9-dimethyl-3a,4,5,5a-tetrahydronaphtho[1,2-*b*]furan-2,8(3*H*,9b*H*)-dione (57)

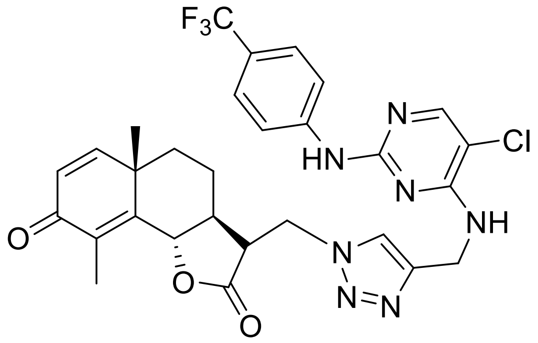

The reaction was implemented using **30** according to the general procedure A. Yield: 42.0 mg (41%); yellowish white crystals; m.p.: 142.7–143.1 °C; $[\alpha]_D^{20}$ = –10 (c 0.157 MeOH); ^{1}H-NMR (500 MHz, $CDCl_3$) δ (ppm): 1.25 (s, 3H), 1.35 (dt, 1H, *J* = 4.1, 13.4 Hz), 1.55-1.67 (m, 1H, overlapping with water), 1.72-1.81 (m, 2H), 1.88-1.94 (m, 1H), 1.97 (s, 3H), 2.91 (dt, 1H, *J* = 4.7, 12.5 Hz), 4.67 (dd, 1H, *J* = 5.0, 14.5 Hz), 4.72-4.85 (m, 4H), 5.90 (t, 1H, *J* = 4.8 Hz), 6.20 (d, 1H, *J*= 9.8 Hz), 6.59 (d, 1H, 9.9 Hz), 7.15 (s, 1H), 7.53 (d, 2H, *J* = 8.3), 7.64 (d, 2H, *J* = 8.3 Hz), 7.66 (s, 1H), 7.98 (s, 1H). ^{13}C-NMR (125 MHz, $CDCl_3$) δ (ppm): 10.8 (CH_3), 22.7 (CH_2), 25.0 (CH_3), 36.8 (CH_2), 37.3 (CH_2), 41.1 (C_q), 47.1 (CH_2), 47.1 (CH), 49.0 (CH), 81.5 (CH), 118.2 (2 × CH), 118.4 (C_q), 123.2 (CH), 123.7 (C_q), 126.0 (CH), 126.1 (2 × CH), 129.2 (C_q), 142.5 (C_q), 142.8 (C_q), 145.5 (C_q), 149.3 (C_q), 153.4 (CH), 154.5 (CH), 157.4 (C_q), 157.6 (C_q), 174.0 (C_q), 185.9 (C_q); ^{19}F-NMR (470 MHz, $CDCl_3$) δ (ppm): –61.7; HRMS (ESI+): m/z calcd. for $C_{29}H_{28}ClF_3N_7O_3^+$ $[M + H]^+$ 614.18888; found 614.18761.

(3*R*,3a*S*,5a*S*,9b*S*)-3-((4-(((5-fluoro-2-((4-(trifluoromethyl)phenyl)amino)pyrimidin-4-yl)amino)methyl)-1*H*-1,2,3-triazol-1-yl)methyl)-5a,9-dimethyl-3a,4,5,5a-tetrahydronaphtho[1,2-*b*]furan-2,8(3*H*,9b*H*)-dione (58)

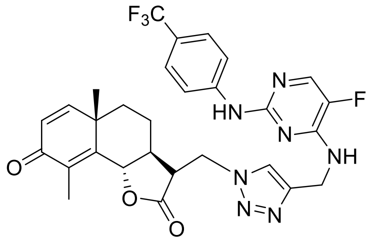

The reaction was implemented using **31** according to the general procedures A and B. Yield: 16.3 mg (16%, Method A) and 39.6 mg (48%, Method B); white crystals; m.p.: 145.2–146.4 °C; $[\alpha]_D^{20}$ = –19 (c 0.150 MeOH); ^{1}H-NMR (500 MHz, $CDCl_3$) δ (ppm): 1.26 (s, 3H), 1.37 (dt, 1H, *J* = 4.1 Hz, *J* = 13.4 Hz), 1.56-1.71 (m, 2H, overlapping with water), 1.72-1.82 (m, 2H), 1.88-1.95 (m, 1H), 1.97 (s, 3H), 2.92 (dt, 1H, *J* = 4.8, 12.5 Hz), 4.68 (dd, 1H, *J* = 4.8, 14.5 Hz), 4.73-4.83 (m, 4H), 5.63 (br s, 1H), 6.21 (d, 1H, *J* = 9.9 Hz), 6.60 (d, 1H, *J* = 9.9 Hz), 7.12 (s, 1H), 7.53 (d, 2H, *J* = 8.3 Hz), 7.63-7.68 (m, 3H), 7.85 (s, 1H). ^{13}C-NMR (125 MHz, $CDCl_3$) δ (ppm): 10.7 (CH_3), 22.7 (CH_2), 25.0 (CH_3), 36.3 (CH_2), 37.3 (CH_2), 41.1 (C_q), 47.1 (CH_2), 47.1 (CH), 49.0 (CH), 81.5 (CH), 117.7 (2 × CH), 121.2 (C_q), 123.1 (C_q), 123.3 (CH), 123.4 (C_q), 126.0 (CH), 126.1 (2 × CH), 129.2 (C_q), 139.3 (CH, *J* = 19.2 Hz), 143.2 (C_q), 145.4 (C_q), 149.3 (C_q), 152.3 (C_q, *J* = 12.2 Hz), 154.5 (CH), 155.0 (C_q), 174.0 (C_q), 185.9 (C_q). ^{19}F-NMR (470 MHz, $CDCl_3$) δ (ppm): –167.1, –61.6; HRMS (ESI+): m/z calcd. for $C_{29}H_{28}F_4N_7O_3^+$ [M + H]$^+$: 598.21843; found 598.21732.

(3*R*,3a*S*,5a*S*,9b*S*)-3-((4-(((5-fluoro-4-((4-(trifluoromethyl)phenyl)amino)pyrimidin-2-yl)amino)methyl)-1*H*-1,2,3-triazol-1-yl)methyl)-5a,9-dimethyl-3a,4,5,5a-tetrahydronaphtho[1,2-*b*]furan-2,8(3*H*,9b*H*)-dione (59)

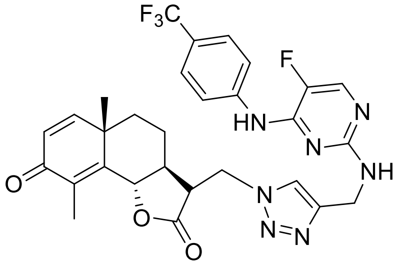

The reaction was implemented using **33** according to the general procedures A and B. Yield: 21.8 mg (21%, Method A) and 41.6 mg (40%, Method B); yellowish white crystals; m.p.: 145.2–146.4 °C; $[\alpha]_D^{20}$ = + 4.8 (c 0.166 MeOH); ^{1}H-NMR (500 MHz, $CDCl_3$) δ (ppm): 1.24 (s, 3H), 1.54-1.74 (m, 4H), 1.89 (d, 1H, *J* = 12.9 Hz), 1.96 (s, 3H), 2.90 (dt, 1H, *J* = 4,4 Hz, 12.2 Hz), 4.45-4.83 (m, 5H), 5.55 (br s, 1H), 6.17 (d, 1H, *J* = 9.9 Hz), 6.56 (d, 1H, *J* = 9.9 Hz), 6.90 (s, 1H), 7.56 (t, 3H, *J* = 9.8 Hz), 7.67 (d, 2H, *J* = 8.2 Hz) 7.93 (s, 1H). ^{13}C-NMR (125 MHz, $CDCl_3$) δ (ppm): 10.6 (CH_3), 22.7 (CH_2), 25.0 (CH_3), 29.7 (CH_2), 37.2 (CH_2), 41.1 (C_q), 46.9 (CH_2), 47.0 (CH), 48.8 (CH), 81.4 (CH), 119.3 (2 × CH), 120.2 (C_q), 122.6 (CH), 123.4 (C_q), 125.9 (CH), 126.2 (2 × CH), 129.0 (C_q), 141.1 (CH), 141.3 (C_q), 147.3 (C_q), 149.5 (C_q), 149.6 (C_q), 149.7 (C_q), 154.6 (CH), 157.6 (C_q), 174.0 (C_q), 185.9 (C_q); ^{19}F-NMR (470 MHz, $CDCl_3$) δ (ppm): –168.9, –61.8; HRMS (ESI+): m/z calcd. for $C_{29}H_{28}F_4N_7O_3^+$ [M + H]$^+$ 598.21843; found 598.21716.

Investigation of antiproliferative activity

Table S1 The antiproliferative effect and IC_{50} values of the prepared new compounds.

Comp.	Conc. (μM)	Growth inhibition (%) ± SEM HeLa	SiHa	MCF-7	MDA-MB-231	A2780	NIH/3T3
**3**	10	23.95 ± 2.95	51.53 ± 2.09	40.14 ± 2.99	44.44 ± 2.30	13.61 ± 3.01	–[2]
	30	53.74 ± 2.03	76.31 ± 0.89	88.08 ± 0.95	89.60 ± 0.82	84.28 ± 0.30	40.02 ± 3.18
Calculated IC_{50} (μM)[3]		25.61	10.21	11.89	11.19	17.64	>30
**34**	10	–	–	–	17.18 ± 2.96	–	–
	30	55.50 ± 1.37	42.69 ± 0.68	56.82 ± 0.79	50.62 ± 1.27	66.54 ± 2.59	26.80 ± 3.15
**35**	10	–	–	–	–	–	–
	30	14.73 ± 2.70	22.51 ± 1.58	10.66 ± 2.55	12.31 ± 1.88	–	–
**36**	10	–	–	–	–	–	–
	30	18.79 ± 2.13	17.99 ± 1.94	29.26 ± 1.70	–	–	–
**37**	10	–	–	–	–	–	–
	30	–	19.07 ± 1.57	12.03 ± 0.75	–	–	–
**38**	10	–	–	–	–	–	–
	30	–	–	21.24 ± 3.06	–	45.64 ± 1.58	–
**39**	10	13.21 ± 3.05	–	–	–	–	–
	30	20.44 ± 0.45	15.65 ± 2.90	–	–	–	–
**40**	10	–	–	–	–	–	–
	30	18.03 ± 0.93	19.10 ± 2.99	–	–	–	25.19 ± 2.40
**41**	10	11.43 ± 2.12	27.08 ± 2.68	24.83 ± 2.39	–	–	13.13 ± 2.07
	30	23.17 ± 1.35	41.48 ± 2.54	33.45 ± 3.03	10.48 ± 2.91	20.21 ± 3.37	15.32 ± 2.82
**42**	10	–	18.02 ± 0.62	–	–	–	14.40 ± 2.38
	30	20.44 ± 1.80	23.63 ± 1.85	–	–	–	17.82 ± 1.11
**43**	10	–	–	–	–	–	–
	30	21.38 ± 1.23	20.83 ± 0.55	–	–	–	–
**44**	10	–	13.08 ± 2.89	–	–	–	–
	30	17.79 ± 2.85	32.83 ± 1.90	13.15 ± 2.64	–	–	–
**45**	10	–	20.76 ± 3.00	–	–	–	–
	30	15.61 ± 0.94	30.38 ± 3.42	–	–	–	–
**46**	10	13.60 ± 3.33	–	–	–	–	–
	30	15.51 ± 2.09	–	–	–	–	–
**47**	10	–	–	–	–	–	–
	30	16.34 ± 3.04	39.48 ± 3.20	36.26 ± 3.31	–	–	–
**48**	10	–	–	–	–	–	–
	30	19.25 ± 0.76	–	–	–	–	–
**49**	10	–	–	–	–	–	–
	30	16.15 ± 1.85	12.83 ± 2.73	15.48 ± 2.90	–	–	–
**50**	10	–	–	–	–	–	–
	30	11.93 ± 0.81	–	–	–	–	–

**51**	10	–	–	–	–	–	–
	30	16.20 ± 2.29	15.10 ± 2.83	13.77 ± 2.41	–	–	–
**52**	10	14.88 ± 0.93	16.51 ± 1.26	–	–	–	–
	30	26.31 ± 0.71	26.44 ± 2.03	16.78 ± 2.74	–	33.33 ± 1.29	–
**53**	10	11.75 ± 2.73	–	–	–	–	–
	30	21.17 ± 2.08	33.78 ± 3.02	–	–	–	–
**54**	10	–	–	–	–	–	–
	30	28.21 ± 1.84	19.72 ± 1.69	–	16.35 ± 2.58	–	–
**55**	10	–	–	–	–	–	–
	30	12.29 ± 1.60	17.56 ± 2.03	10.54 ± 1.69	–	–	–
**56**	10	15.64 ± 1.95	–	–	–	17.44 ± 2.46	–
	30	37.31 ± 1.64	26.91 ± 1.11	49.80 ± 2.50	20.20 ± 1.96	70.17 ± 1.45	–
**57**	10	–	–	–	–	–	–
	30	–	45.48 ± 3.12	–	–	–	–
**58**	10	–	–	–	–	–	–
	30	–	–	–	–	25.71 ± 2.86	–
**59**	10	–	–	–	–	–	–
	30	–	–	–	–	19.86 ± 3.10	–

[1]: Mean ± SEM values from two determinations with five parallel wells in each.

[2]: Inhibition values less than 10% are regarded as negligible and are not given numerically.

[3]: Calculated from viability results of experiments applying a broad range of concentrations (0.1–30 μM).

Figure S1 Determination of IC_{50} values of azide **3** on HeLa, MDA-MB231, SiHa, MCF-7 and A2780 human cancer cell and NIH/3T3 fibroblast cell tissues

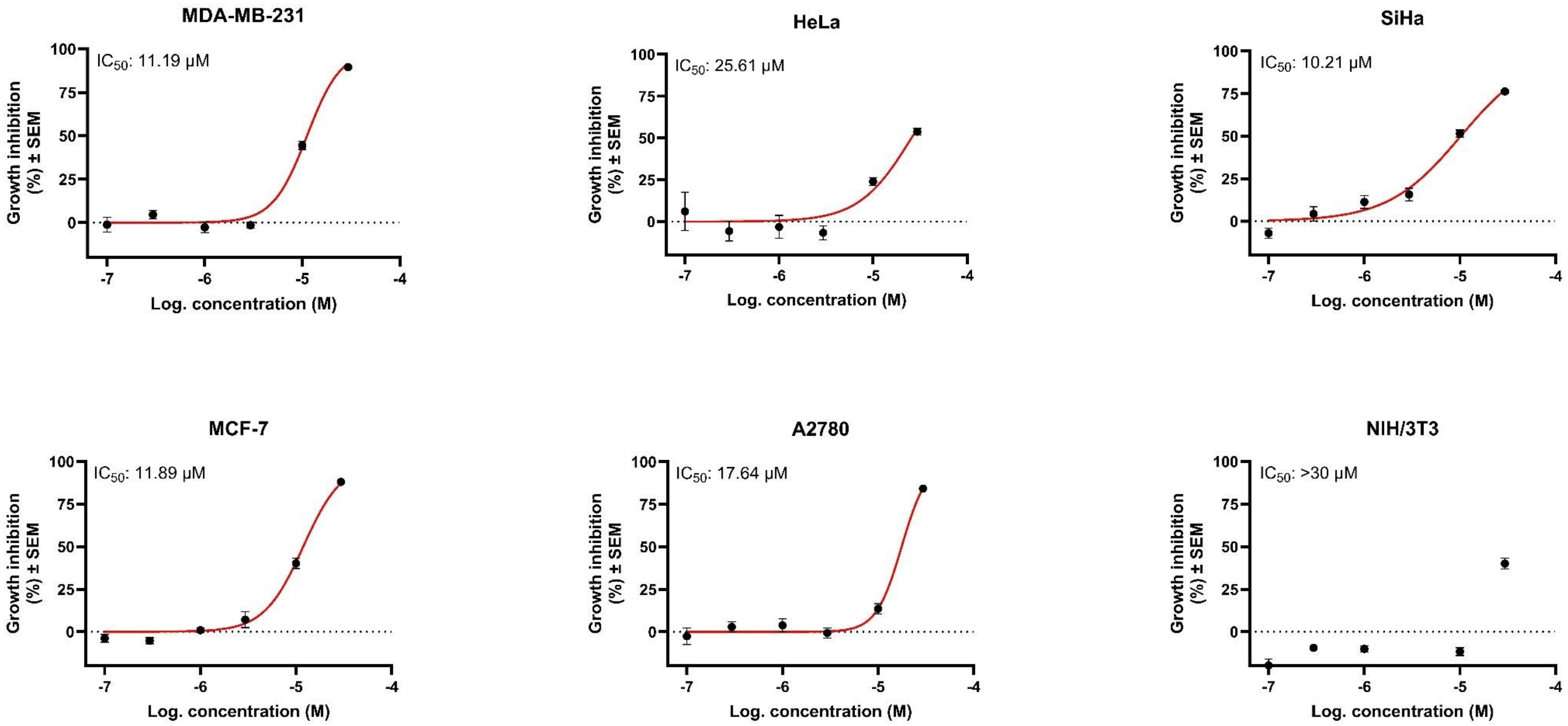

Investigation of antibacterial and antifungal activity

Table S2 Investigation of antibacterial and antifungal activity

Analogue	Conc. (μg/ml)	*B.subtilis* SZMC 0209	*S.aureus* SZMC14611	*E.coli* SZMC 6271	*P.aeruginosa SZMC* 0568	*C.albicans* SZMC1533	*C.krusei* SZMC1352
**Ampicillin**	100	99.7 ± 1.7	100 ± 5.4	100.2 ± 4.1	100 ± 6.7	-	-
	10	88.8 ± 9.8	99.6 ± 8.5	72.4 ± 15.8	27.5 ± 6.5	-	-
**Nystatin**	100	-	-	-	-	98.9 ± 0.9	99.3 ± 1
	10	-	-	-	-	99.4 ± 1.5	99 ± 1.9
**3**	100	3.2 ± 13.2	99.2 ± 14.3	8.3 ± 3.2	0	45.4 ± 16.8	10.9 ± 8.6
	10	15.3 ± 5.1	19.6 ± 6.5	1 ± 1.1	0	5.1 ± 15.7	2.5 ± 16.6
**34**	100	-	-	-	-	-	-
	10	0	16.1 ± 5.6	3.6 ± 3.1	0	23.2 ± 4.8	10.1 ± 17.1
**35**	100	0	7.5 ± 8	0	0	0	0
	10	6.4 ± 8.4	15.5 ± 6.9	2 ± 3.8	0	33.5 ± 18.4	0
**35**	100	-	-	-	-	-	-
	10	0	11.2 ± 3.7	10.3 ± 2.1	0	27.7 ± 9	29.6 ± 24.1
**35**	100	7.3 ± 15.9	13.9 ± 8.9	0	0	45.9 ± 14.2	8.5 ± 9.6
	10	8.6 ± 9.6	16.3 ± 6.8	2.8 ± 3.1	0	49.1 ± 13.7	8.6 ± 7.9
**38**	100	2.9 ± 4.1	12.6 ± 6.8	0	0	20.5 ± 14.4	45.9 ± 27.9
	10	18.5 ± 4.2	8.8 ± 3.7	0	0	0	0
**39**	100	3.9 ± 10.1	28.3 ± 11.5	0	0	31.4 ± 7.5	32 ± 13.4
	10	19.3 ± 4.1	0.6 ± 8.3	0	0	0	0.7 ± 6
**40**	100	20.8 ± 7.5	26.3 ± 12.8	0	0	35.8 ± 16.7	12 ± 10.3
	10	26.7 ± 8.6	11.6 ± 4	0	0	10.9 ± 2.7	7.9 ± 26
**41**	100	23.4 ± 12.8	20.9 ± 5.2	0	0	32.3 ± 4	40.7 ± 2.7

	10	33.1 ± 3	11.5 ± 5.8	12.5 ± 5.9	8 ± 4.7	0	0
**42**	100	14.6 ± 19.6	26.1 ± 11.7	0	0	34.9 ± 9.4	33.1 ± 6.5
	10	27.5 ± 3.4	3.6 ± 5.8	0.1 ± 6.6	3.9 ± 4.5	0	0
**43**	100	14.5 ± 9.5	32.2 ± 5.7	0	0	40.9 ± 7.7	61.3 ± 15.1
	10	23.6 ± 5.9	13.8 ± 5.7	0	0	0	22.5 ± 6.6
**44**	100	30.6 ± 12.4	2.2 ± 9.5	12 ± 5.1	0	1.8 ± 11.9	0
	10	28.1 ± 6.9	1.4 ± 9.3	11.7 ± 3.8	0	0	51.4 ± 17.3
**45**	100	-	-	-	-	-	-
	10	7.7 ± 9.2	13.2 ± 1.6	21.9 ± 3.2	20.6 ± 1.1	24.5 ± 17.7	19.8 ± 8.3
**46**	100	-	-	-	-	-	-
	10	10.7 ± 8.2	11.2 ± 1.5	11.9 ± 3.0	22.6 ± 1.6	23.5 ± 18.5	12.7 ± 7.3
**47**	100	0	20.1 ± 8.2	14.2 ± 3.3	18.6 ± 2.6	15.3 ± 6	29.7 ± 14.1
	10	5.6 ± 7	0	10.1 ± 4.2	21.6 ± 4.6	25.6 ± 13.6	1.4 ± 6.7
**48**	100	12.5 ± 5.9	12.9 ± 2.4	17.5 ± 5.3	21.9 ± 5.6	2.9 ± 13.2	0
	10	13.5 ± 9.1	6.9 ± 4.2	20.5 ± 2.5	28 ± 2.1	18.8 ± 3.7	0
**49**	100	14.1 ± 8.7	14.7 ± 5	14.2 ± 6	20.5 ± 8	4.9 ± 7.1	0
	10	8 ± 7.9	2.7 ± 6.1	16.8 ± 5.9	25.4 ± 3.8	0	0
**50**	100	-	-	-	-	-	-
	10	6.8 ± 7.4	9.4 ± 7.1	19 ± 2.1	14.6 ± 2.3	46.8 ± 14.5	0
**51**	100	-	-	-	-	-	-
	10	0	15.9 ± 5.3	31.3 ± 6.2	26.9 ± 7.5	4.2 ± 5	0
**52**	100	25.3 ± 12.9	98.9 ± 17.9	25.4 ± 4.7	5.5 ± 7.5	73.5 ± 21.2	19.6 ± 4.7
	10	35.2 ± 9.9	7.9 ± 4.7	15.7 ± 2.2	9.5 ± 6.2	3.3 ± 12.9	0
**53**	100	2.2 ± 17.5	8.9 ± 10.5	14.9 ± 6.4	18.1 ± 4.6	26.7 ± 16.7	6.4 ± 22.9
	10	13.2 ± 5.3	5.1 ± 7.5	20.5 ± 4.3	22.2 ± 3.1	45.7 ± 19.6	0
**54**	100	7.9 ± 12.8	7.1 ± 5.9	0	0	51.8 ± 10.6	2.1 ± 8.7
	10	16 ± 6.3	13.4 ± 3.6	8.3 ± 3	0	10.4 ± 14.2	5.1 ± 5.5

**55**	100	20.7 ± 4.3	8.6 ± 8.1	0	0	28.9 ± 7.3	36.6 ± 4.1
	10	11.9 ± 6.4	13.1 ± 3.6	5.2 ± 1.6	0	15.5 ± 9.6	5.2 ± 6.7
**56**	100	-	-	-	-	-	-
	10	19.2 ± 5.6	0	0	0	32.7 ± 14.8	0
**57**	100	-	-	-	-	-	-
	10	2 ± 7.1	4.8 ± 8	10.6 ± 1.6	0	31.7 ± 7.4	0
**58**	100	-	-	-	-	-	-
	10	13 ± 6.7	12 ± 4.9	11 ± 3	0	44.8 ± 13.3	0
**59**	100	-	-	-	-	-	-
	10	2.6 ± 4.9	12.4 ± 8	6.3 ± 0.9	0	50.1 ± 20.6	17.1 ± 10.4

NMR spectra of new compounds

(3*R*,3a*S*,5a*S*,9b*S*)-3-(azidomethyl)-5a,9-dimethyl-3a,5,5a,9b-tetrahydronaphtho[1,2-*b*]furan-2,8(3*H*,4*H*)-dione (**3**)

Figure S2 ^{1}H-NMR of compound **3**

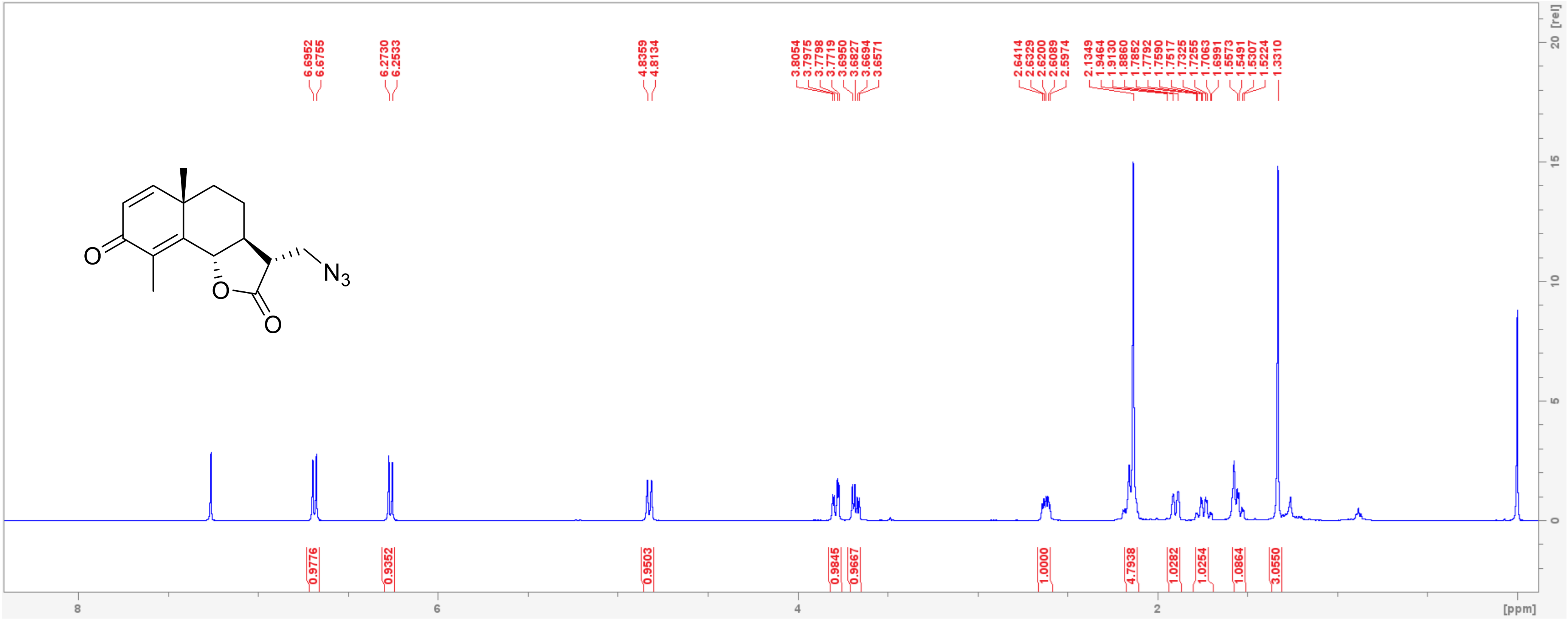

Figure S3 ^{13}C-NMR of compound **3**

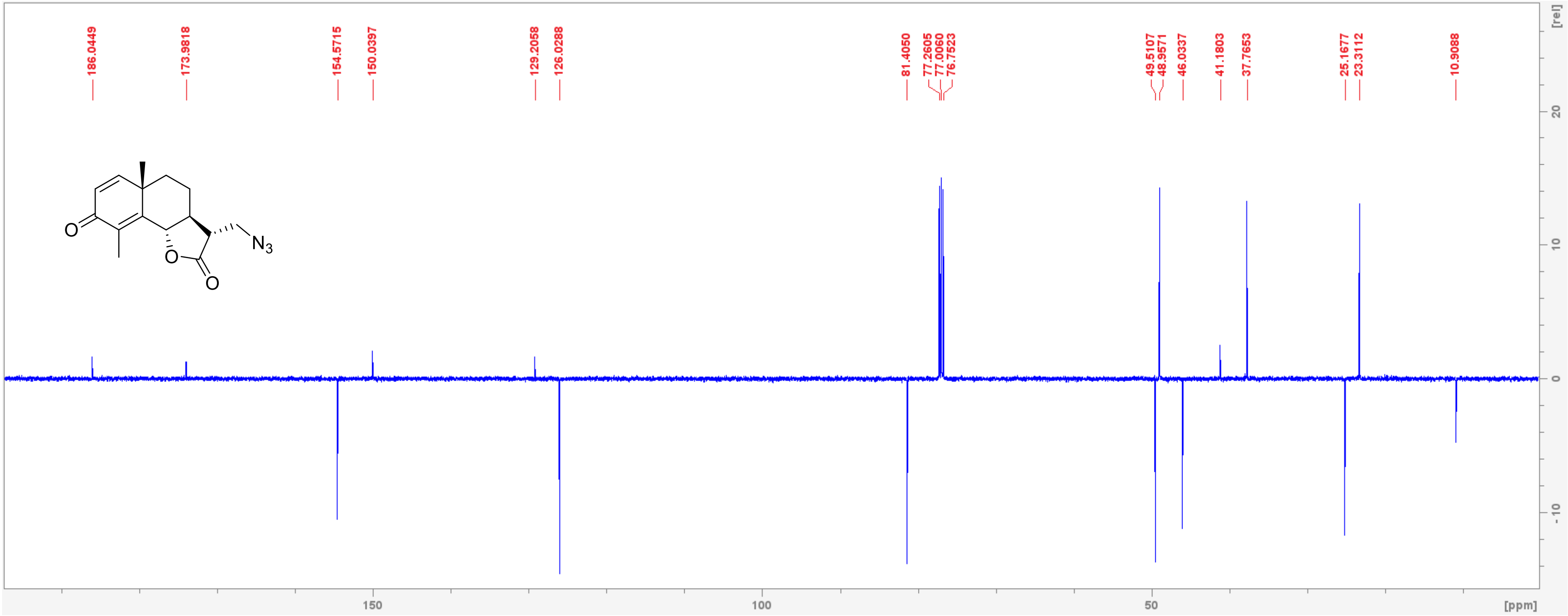

Figure S4 COSY of compound **3**

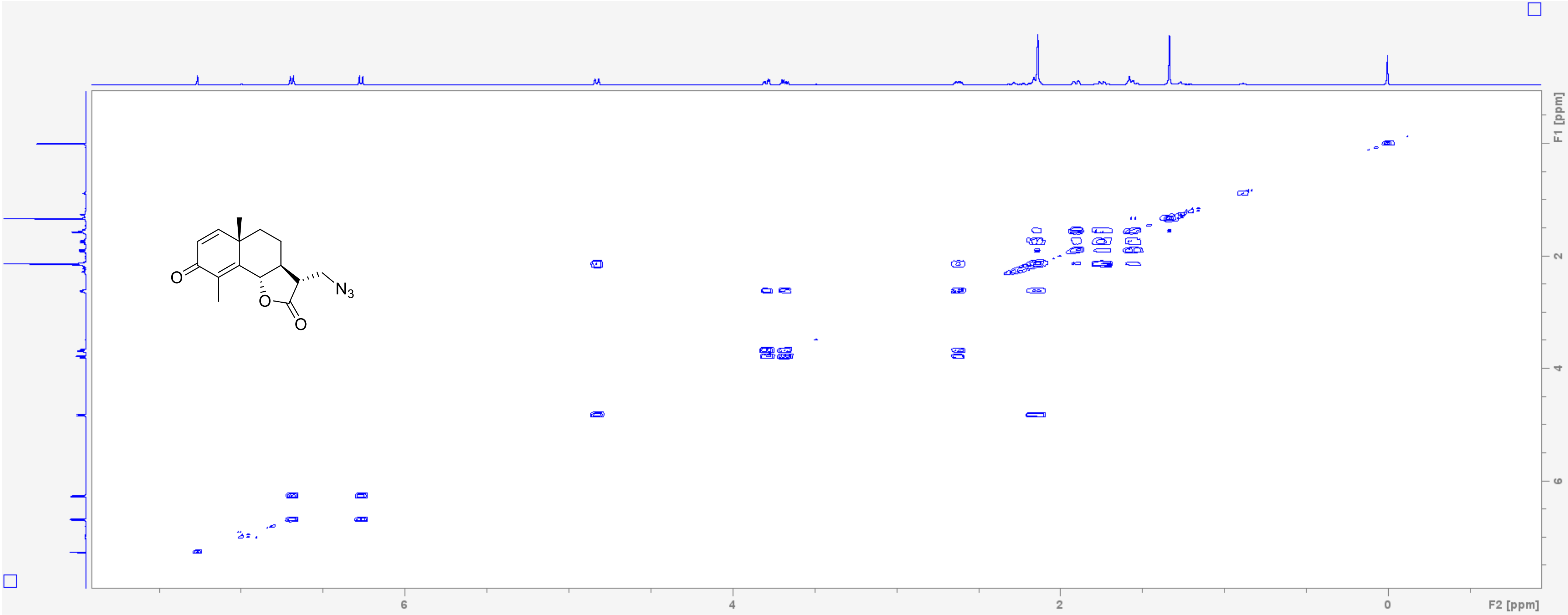

Figure S5 NOESY of compound **3**

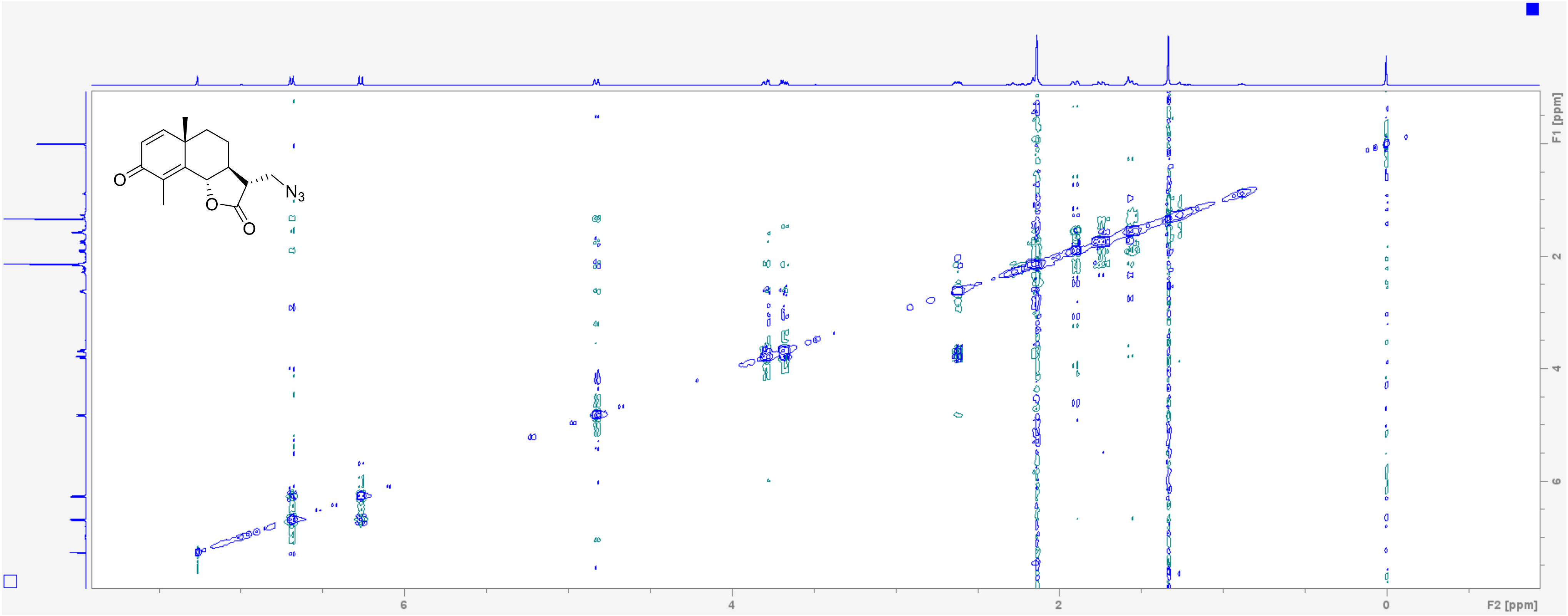

Figure S6 HSQC of compound **3**

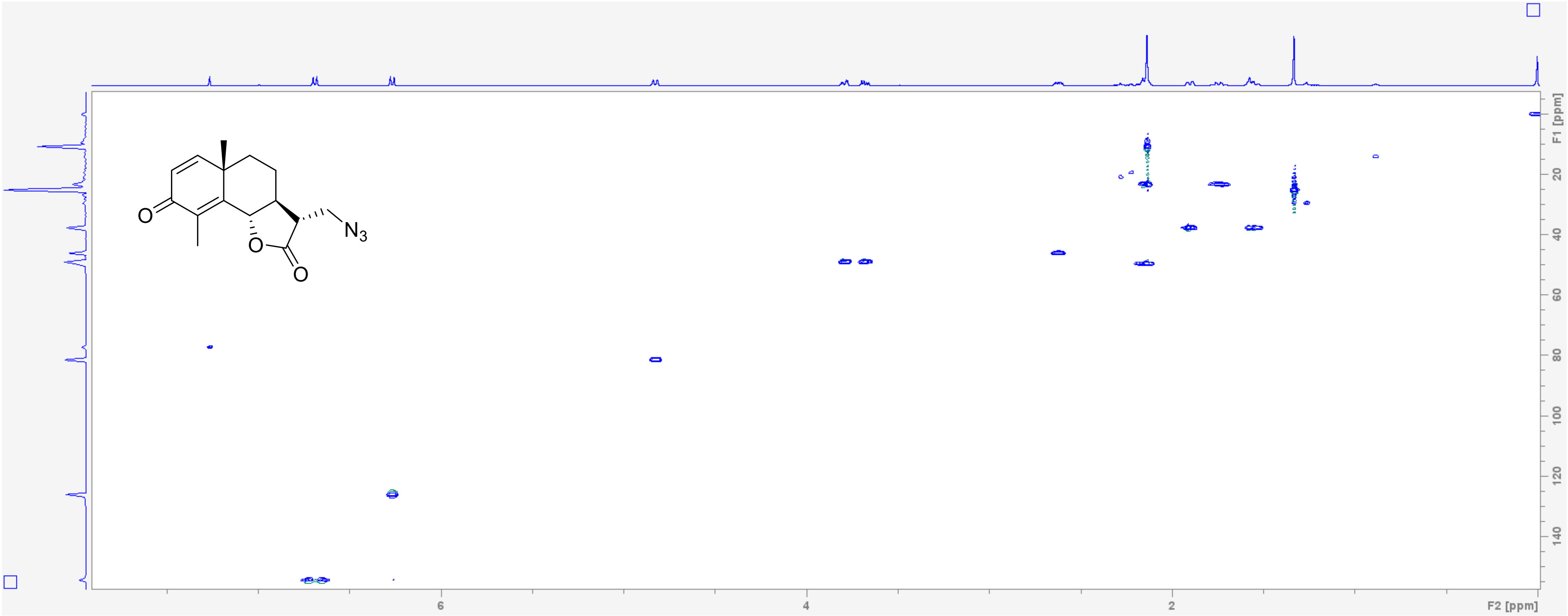

Figure S7 HMBC of compound **3**

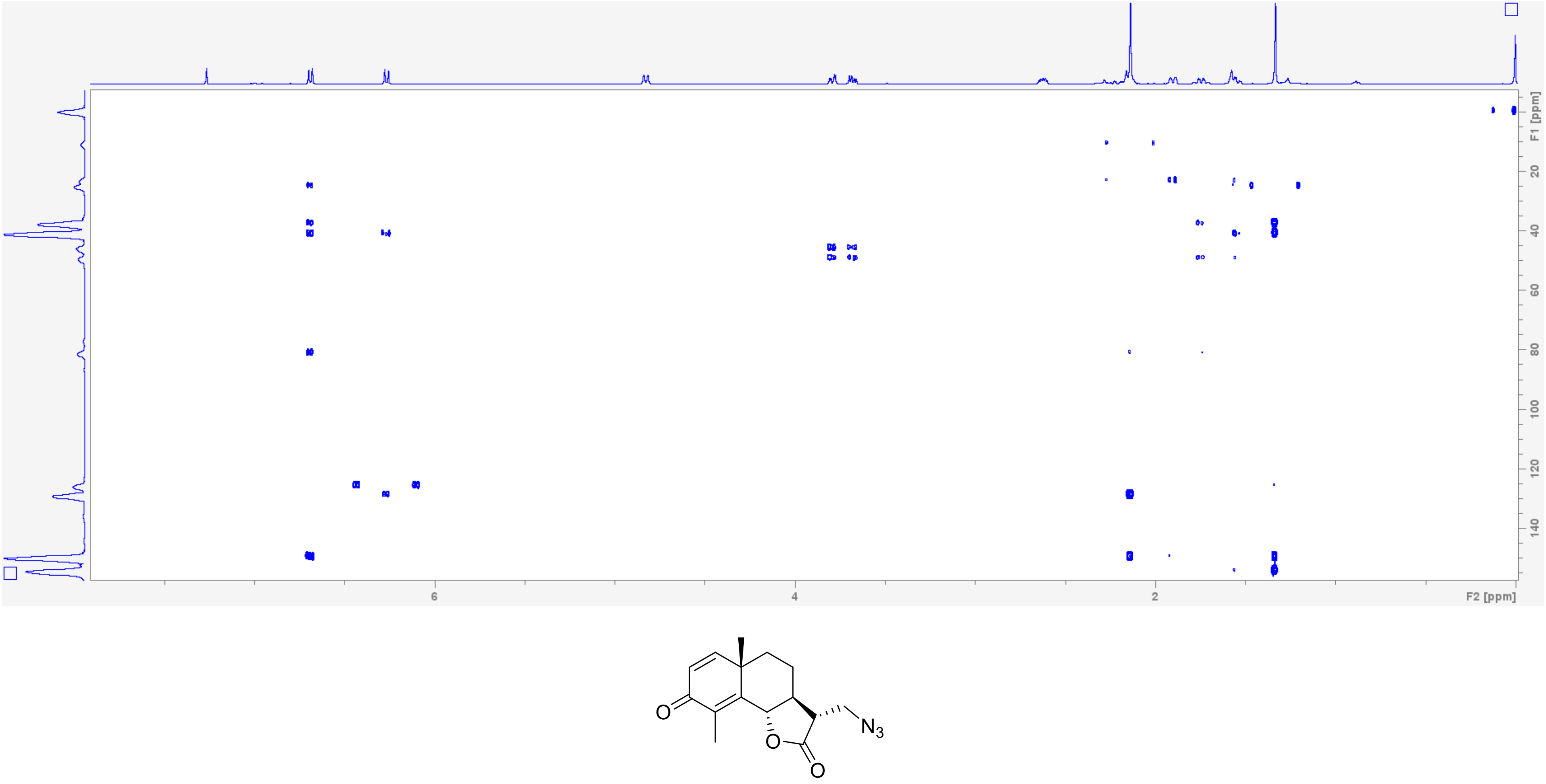

5-fluoro-N2-(prop-2-yn-1-yl)-N4-(4-(trifluoromethyl)phenyl)pyrimidine-2,4-diamine (**33**)

Figure S8 ^{1}H-NMR of compound **33**

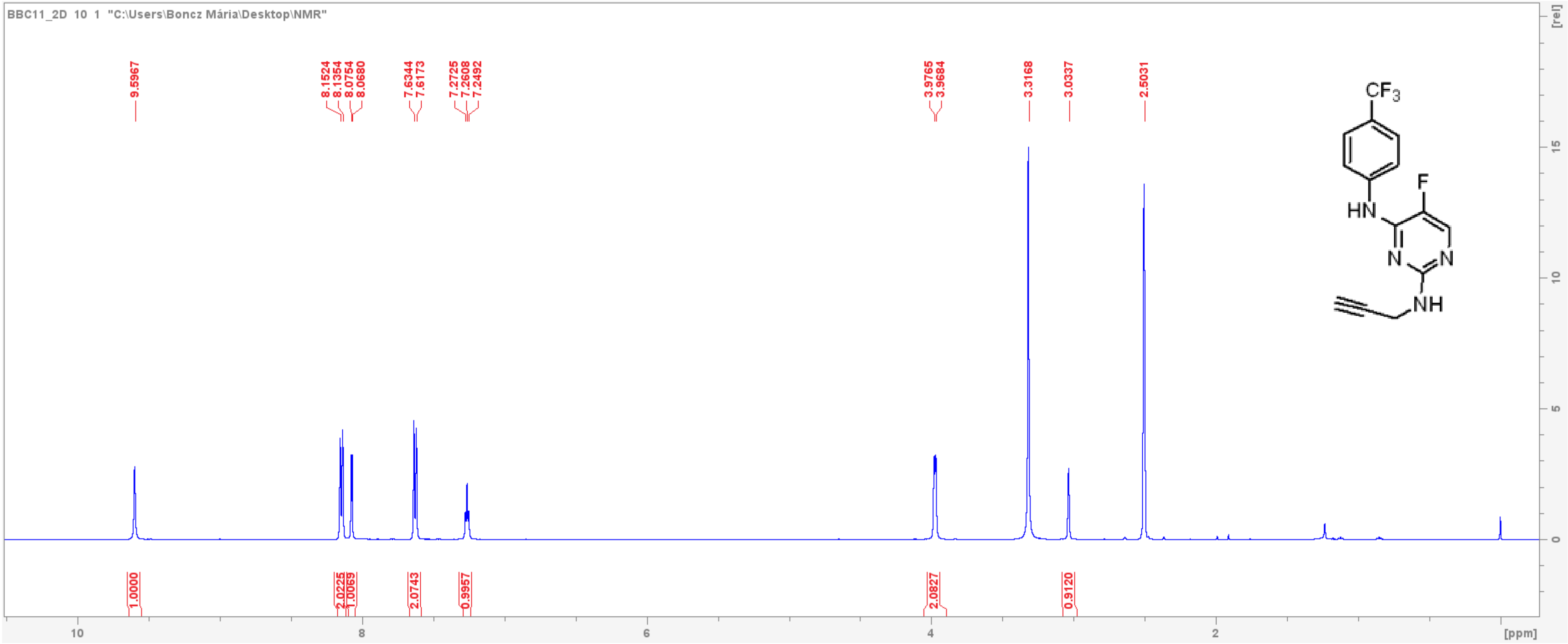

Figure S9 ^{13}C-NMR of compound **33**

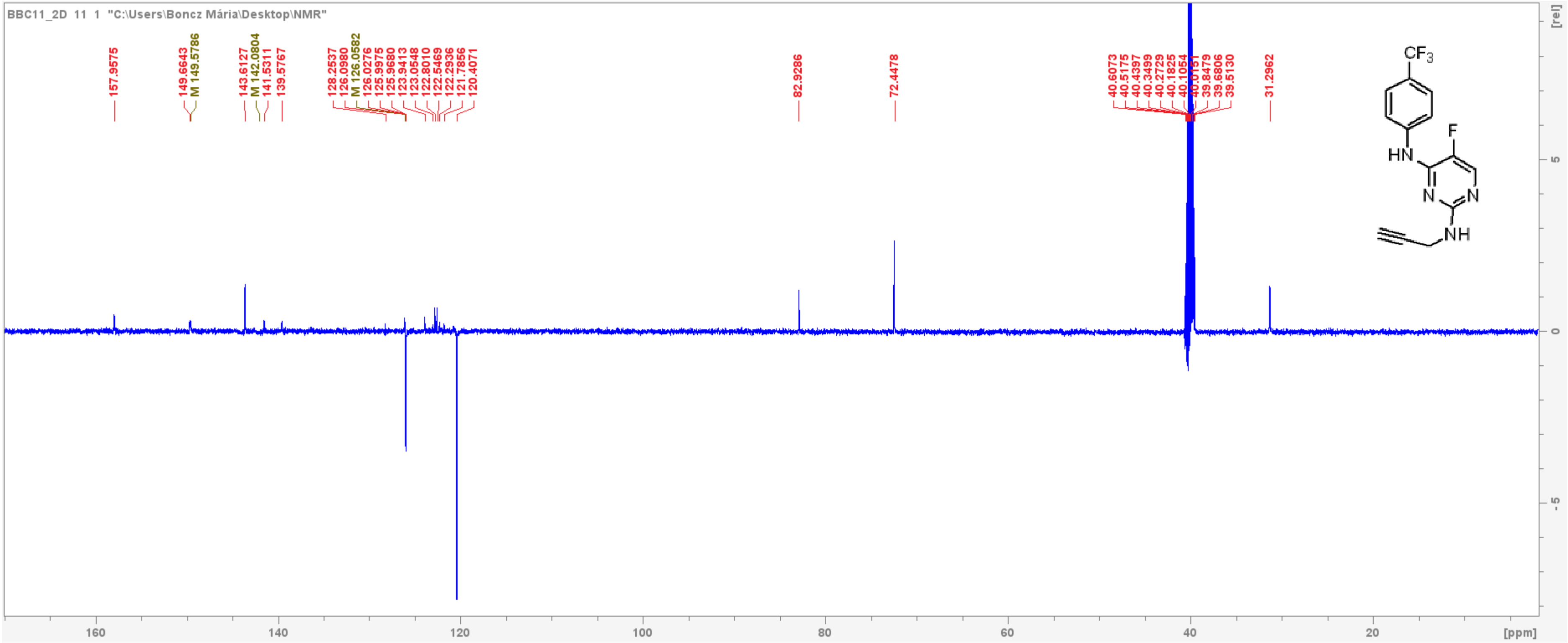

Figure S10 HSQC of compound **33**

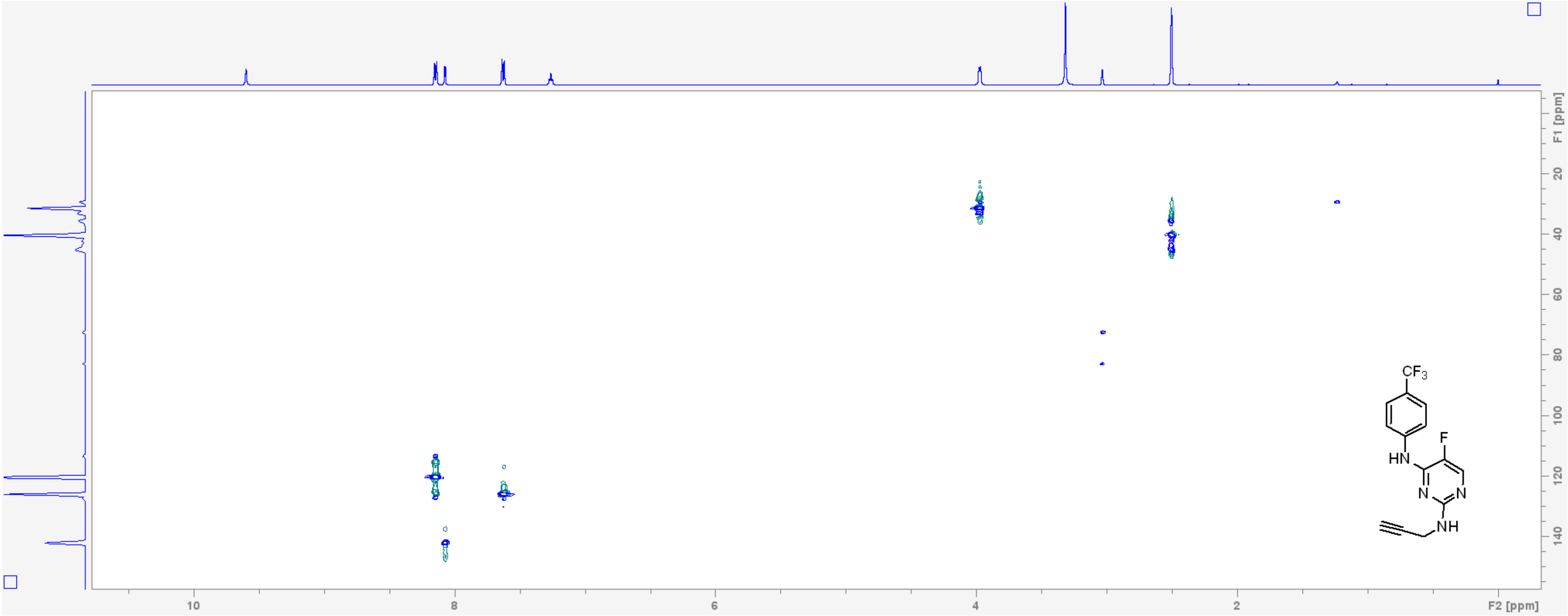

Figure S11 HMBC of compound **33**

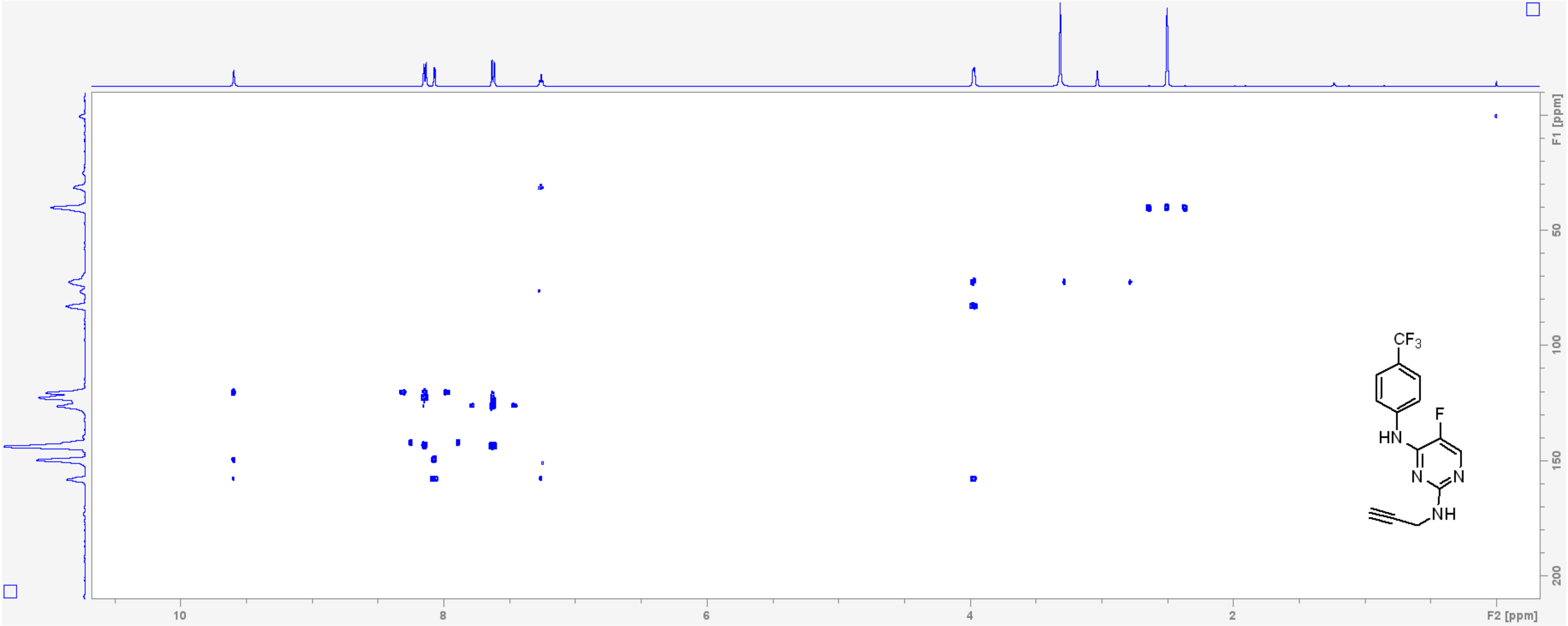

Figure S12 ^{19}F-NMR of compound **33**

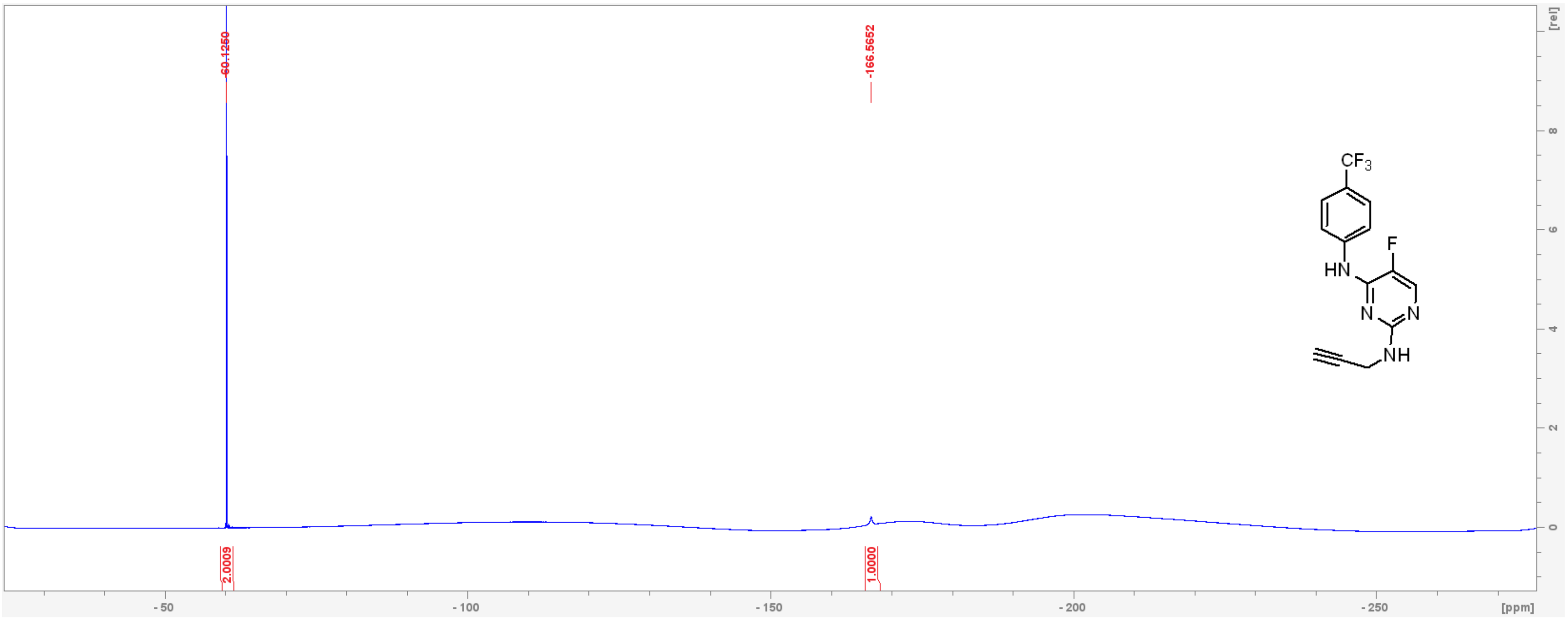

(5a*S*,9b*S*)-5a,9-dimethyl-3-((4-phenyl-1*H*-1,2,3-triazol-1-yl)methyl)-3a,5,5a,9b-tetrahydronaphtho[1,2-*b*]furan-2,8(3*H*,4*H*)-dione, (**34**)

Figure S13 ^{1}H-NMR of compound **34**

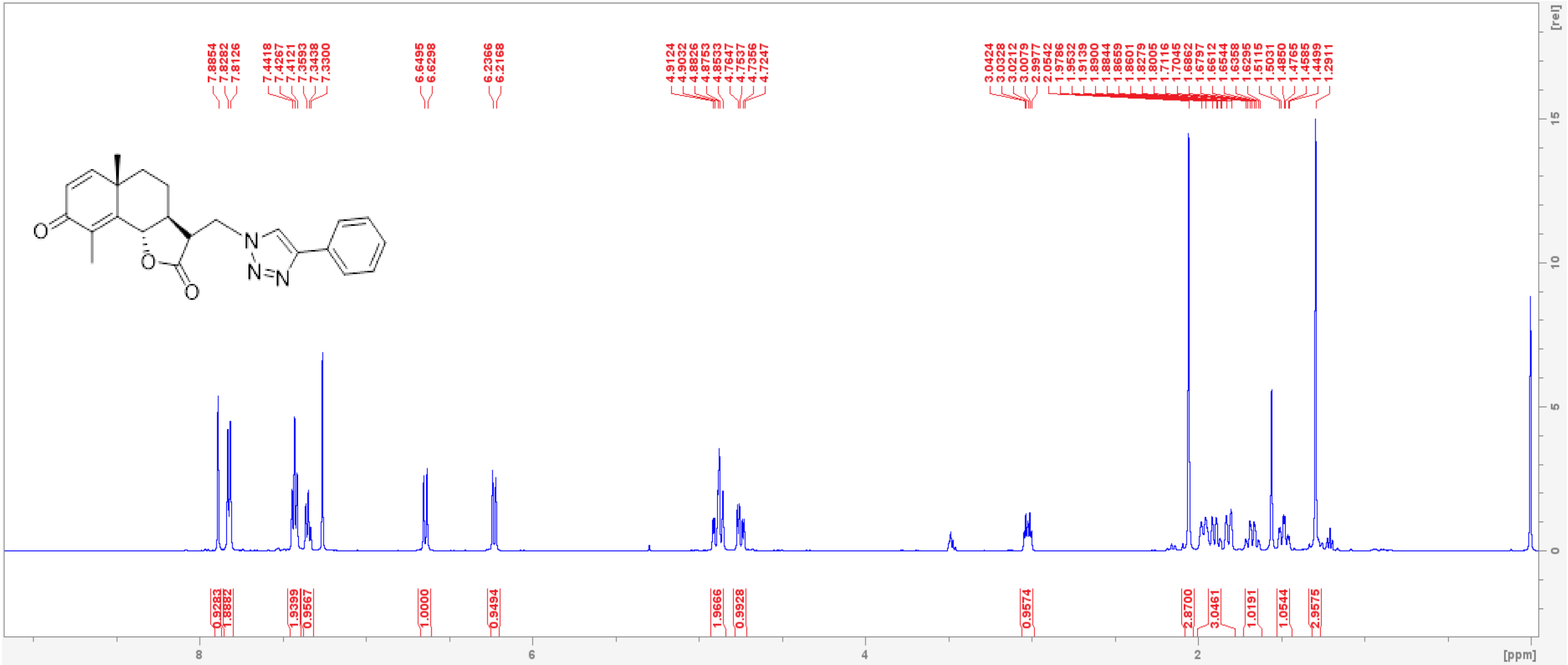

Figure S14 ^{13}C-NMR of compound **34**

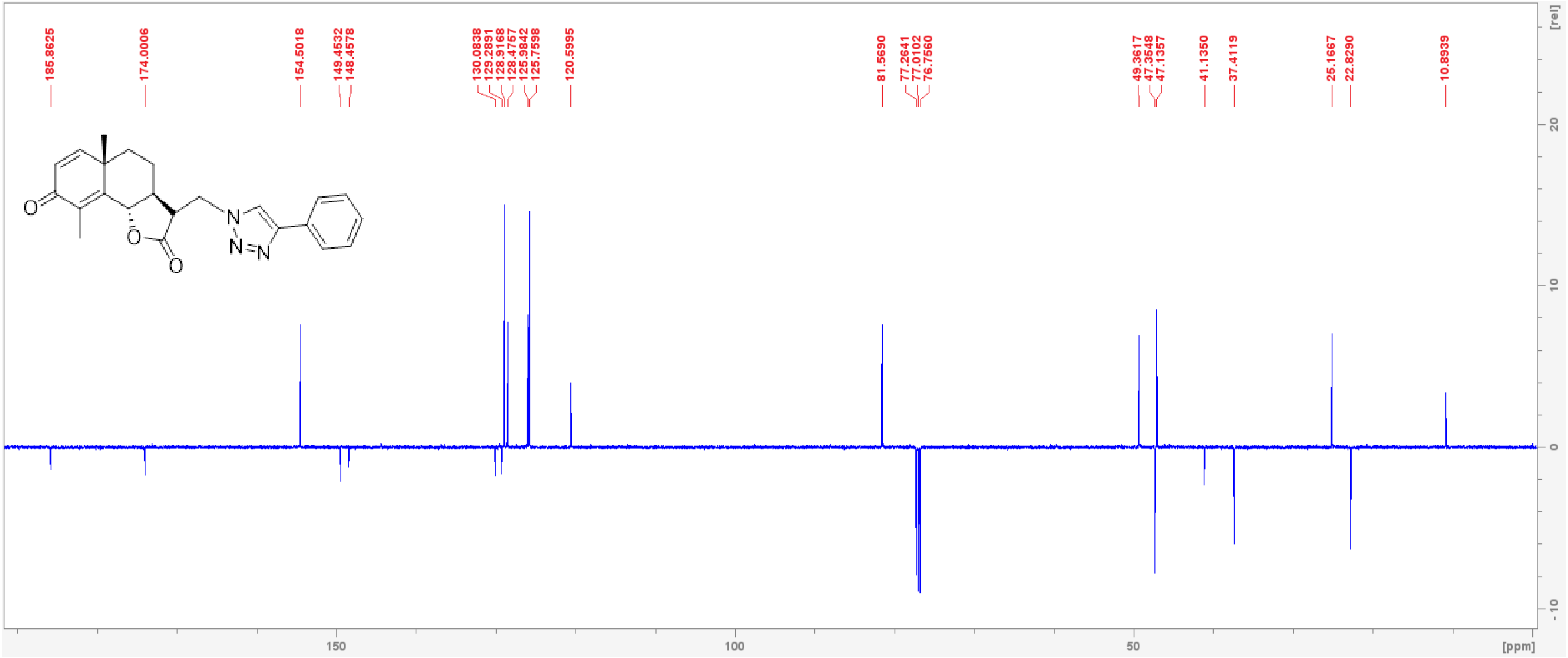

(5a*S*,9b*S*)-5a,9-dimethyl-3-((4-benzyl-1*H*-1,2,3-triazol-1-yl)methyl)-3a,5,5a,9b-tetrahydronaphtho[1,2-*b*]furan-2,8(3*H*,4*H*)-dione (**35**)

Figure S15 ^{1}H-NMR of compound **35**

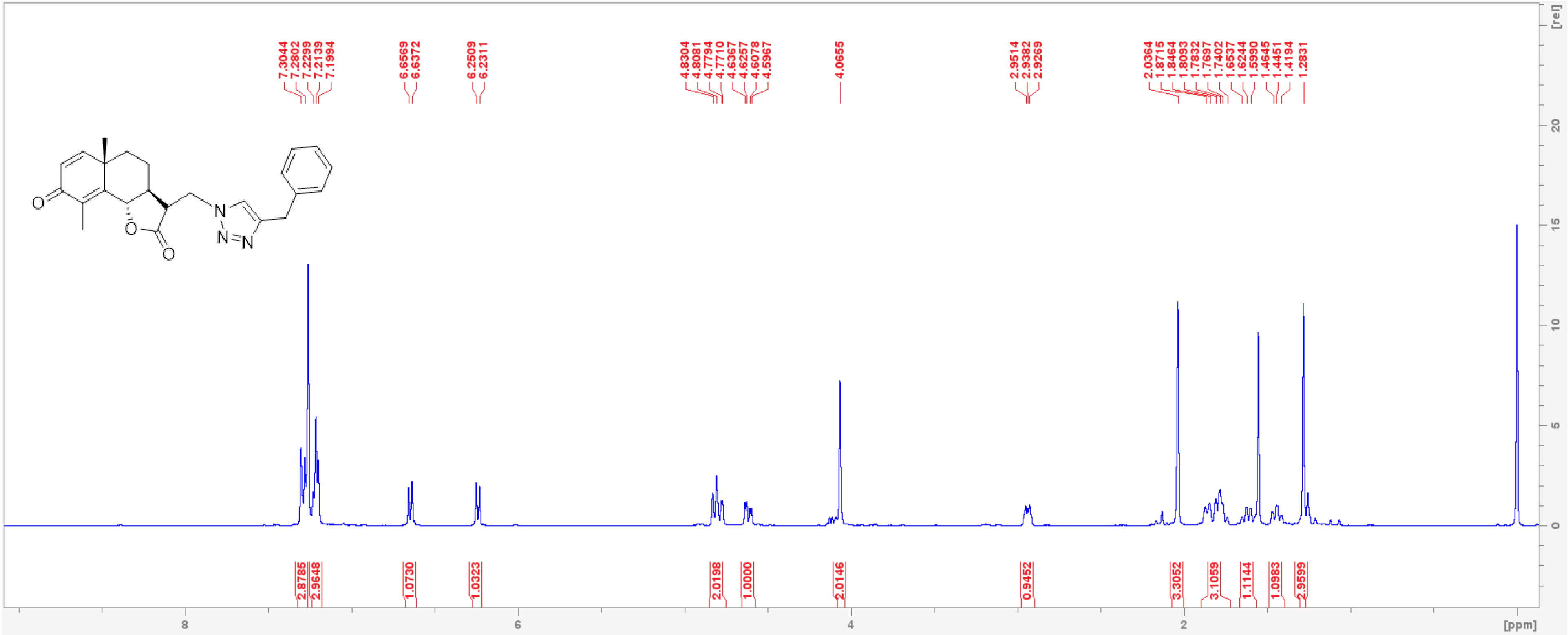

Figure S16 ^{13}C-NMR of compound **35**

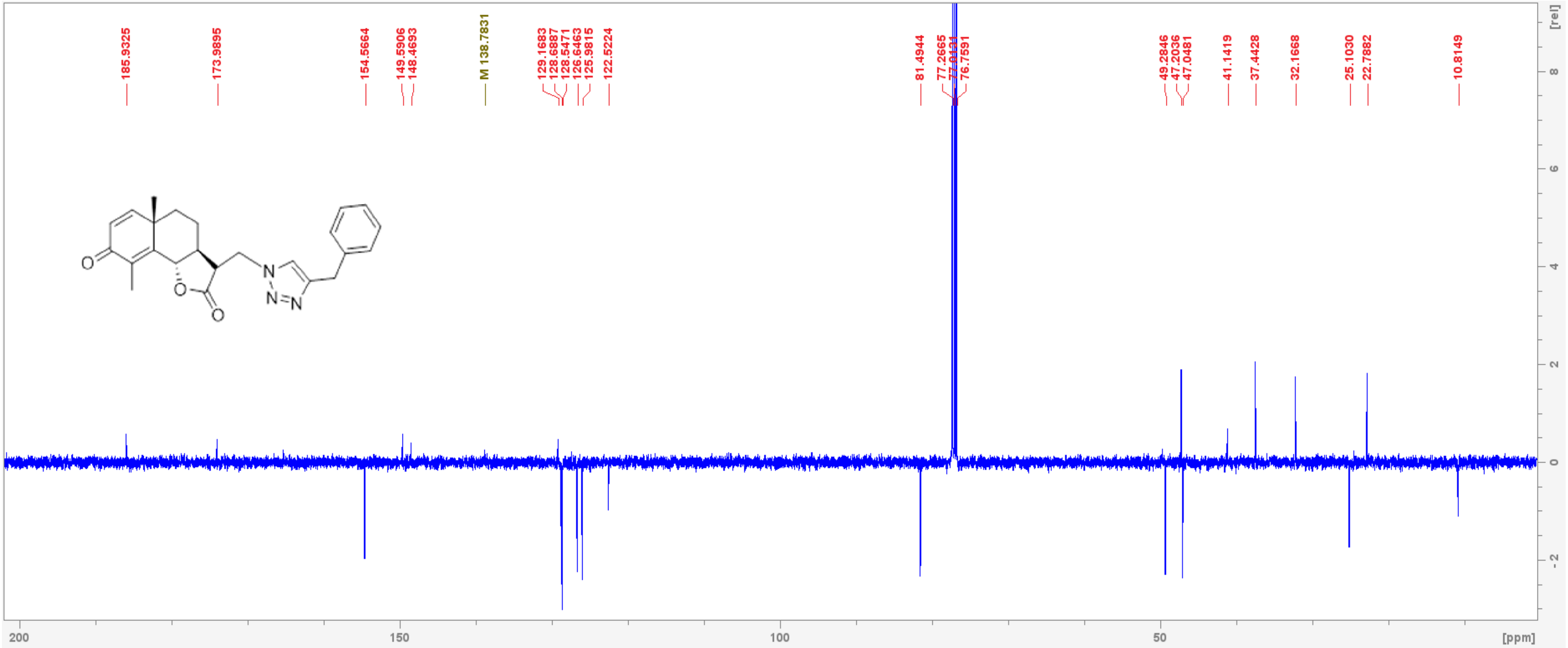

(5a*S*,9b*S*)-5a,9-dimethyl-3-((4-(4-methoxyphenyl)-1*H*-1,2,3-triazol-1-yl)methyl)-3a,5,5a,9b-tetrahydronaphtho[1,2-*b*]furan-2,8(3*H*,4*H*)-dione (**36**)

Figure S17 ^{1}H-NMR of compound **36**

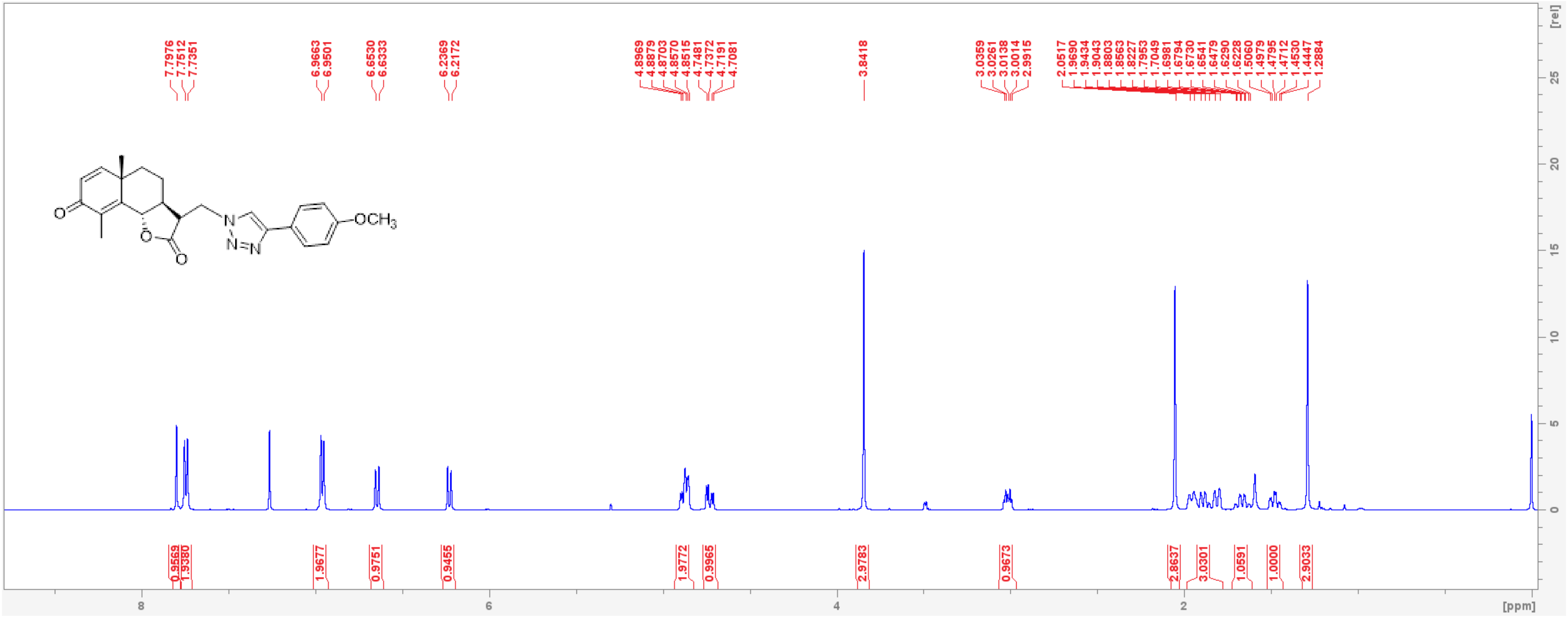

Figure S18 ^{13}C-NMR of compound **36**

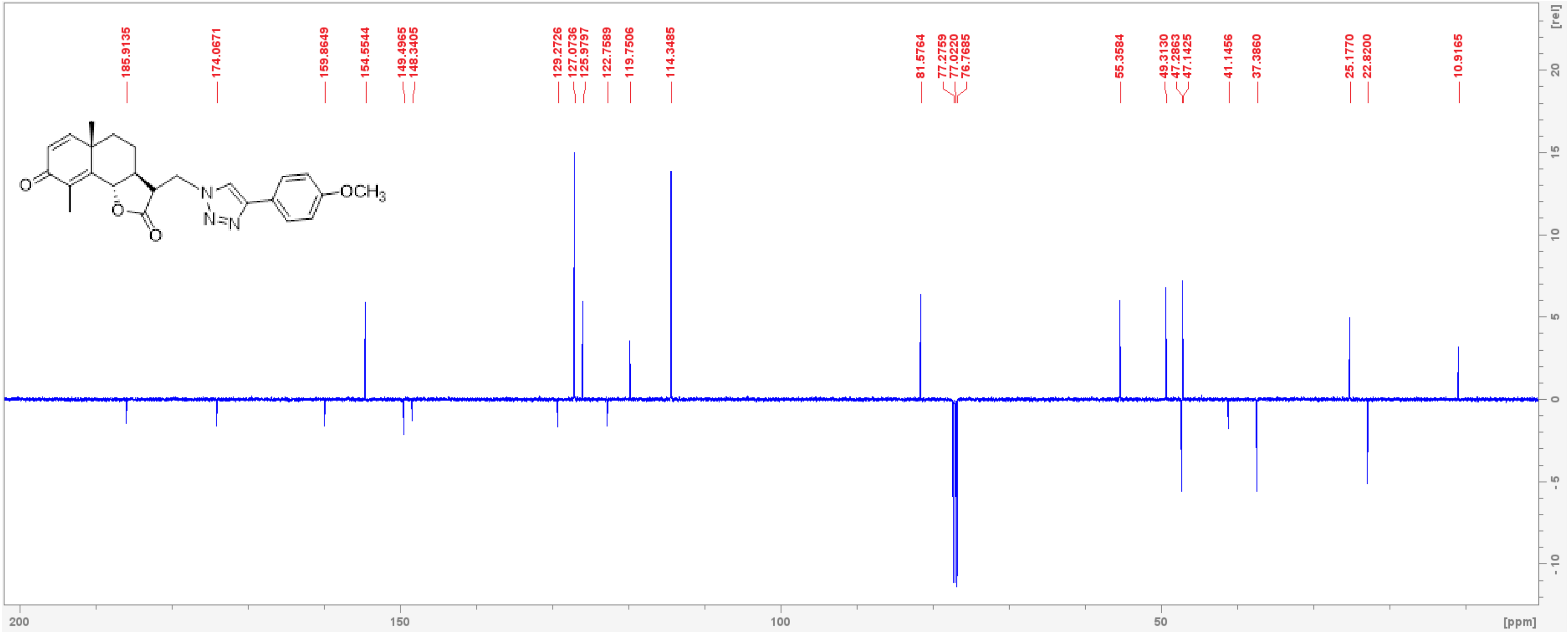

(5a*S*,9b*S*)-5a,9-dimethyl-3-((4-(pyridin-2-yl)-1*H*-1,2,3-triazol-1-yl)methyl)-3a,5,5a,9b-tetrahydronaphtho[1,2-*b*]furan-2,8(3*H*,4*H*)-dione (**37**)

Figure S19 ^{1}H-NMR of compound **37**

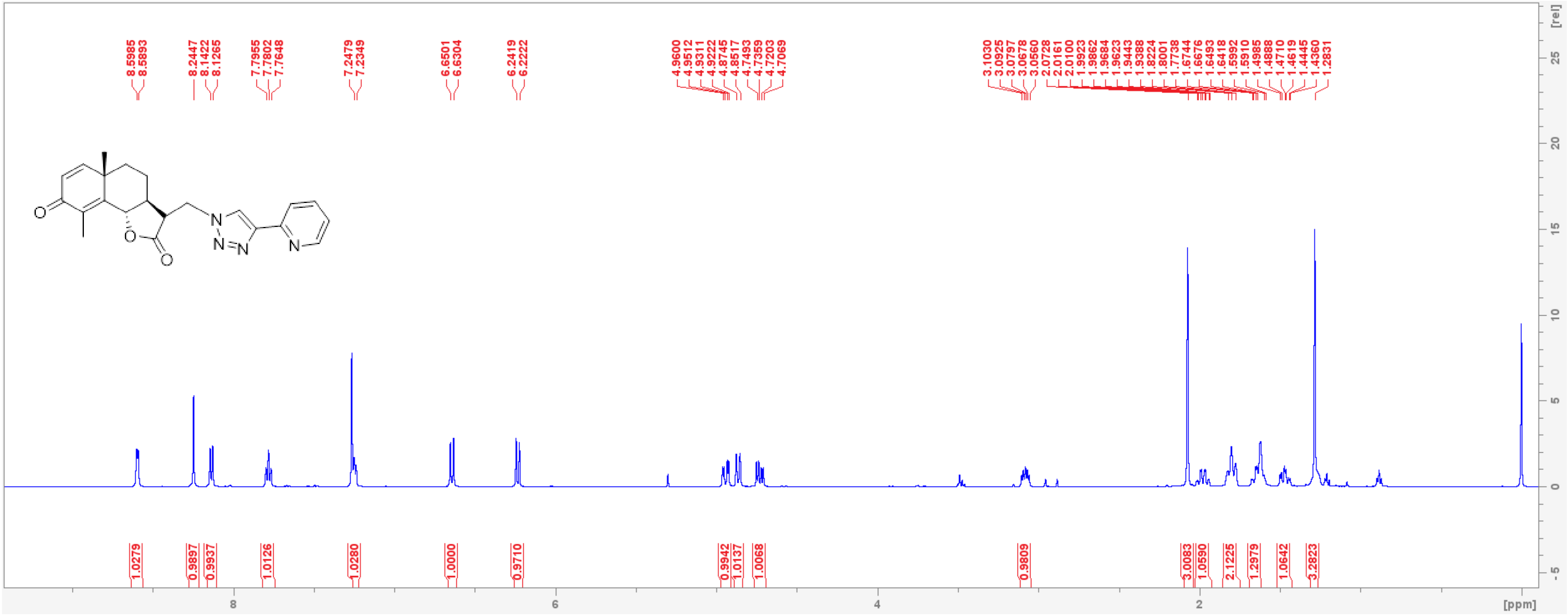

Figure S20 ^{13}C-NMR of compound **37**

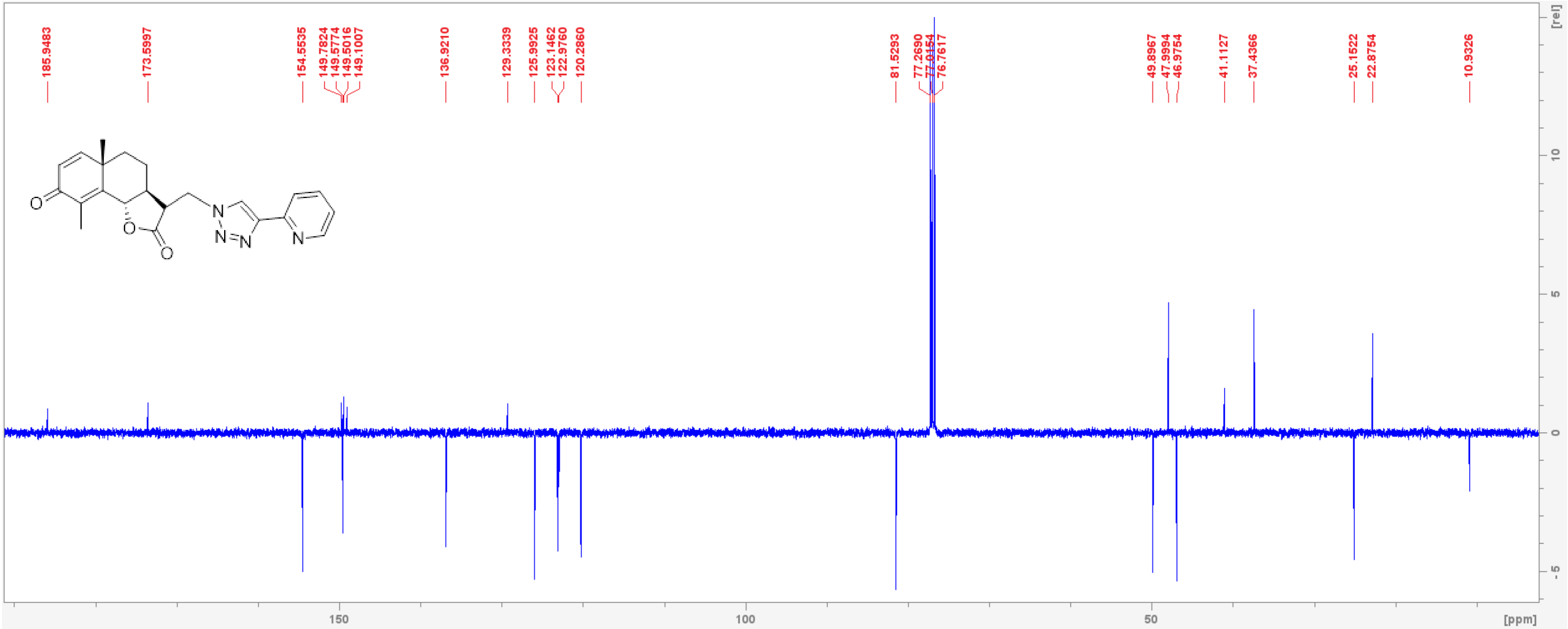

1-((1-(((5aS,9bS)-5a,9-dimethyl-2,8-dioxo-2,3,3a,4,5,5a,8,9b-octahydronaphtho[1,2-*b*]furan-3-yl)methyl)-1*H*-1,2,3-triazol-4-yl)methyl)pyrimidine-2,4(1*H*,3*H*)-dione (**38**)

Figure S21 ^{1}H-NMR of compound **38**

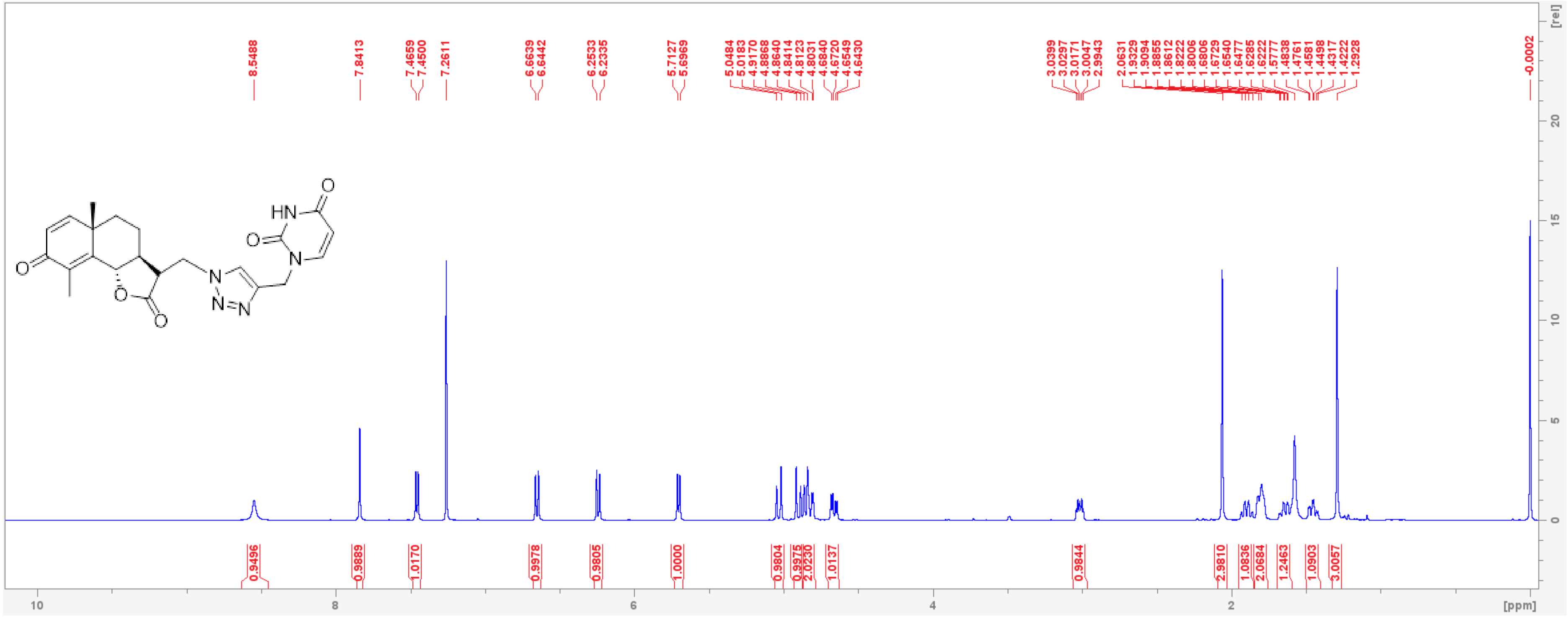

Figure S22 ^{13}C-NMR of compound **38**

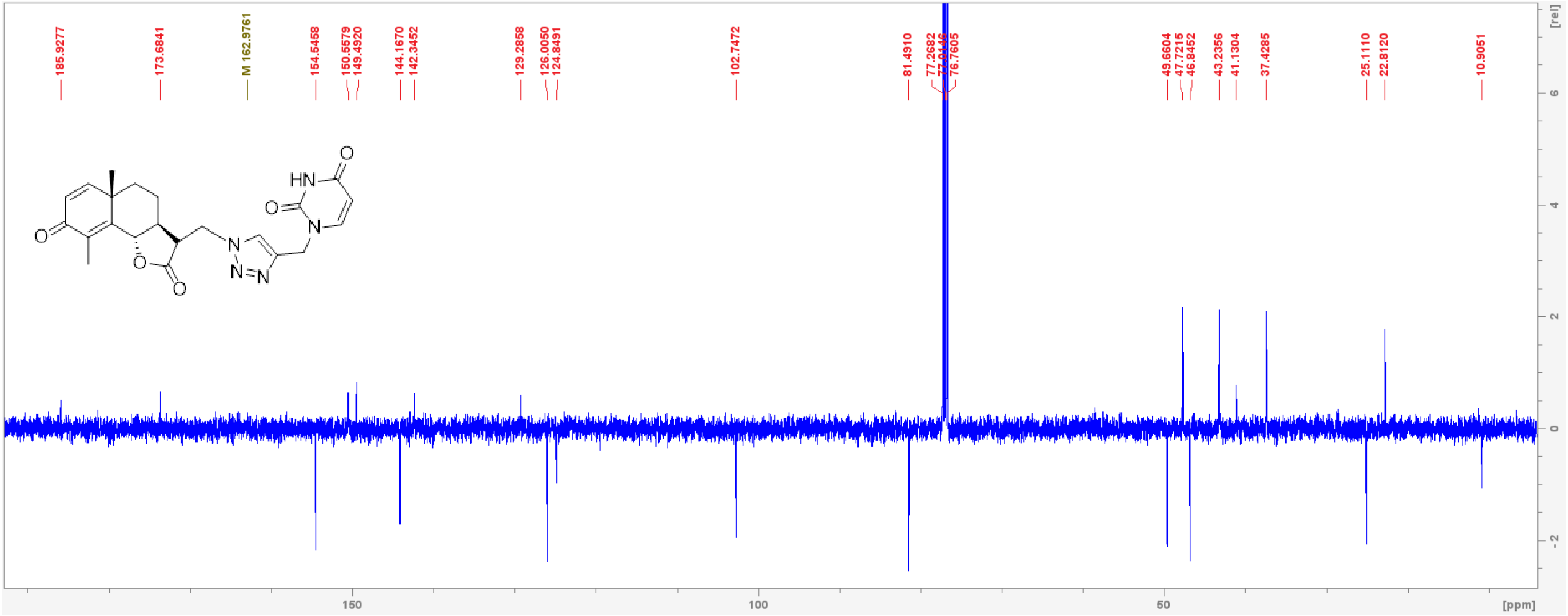

Figure S23 HSQC of compound **38**

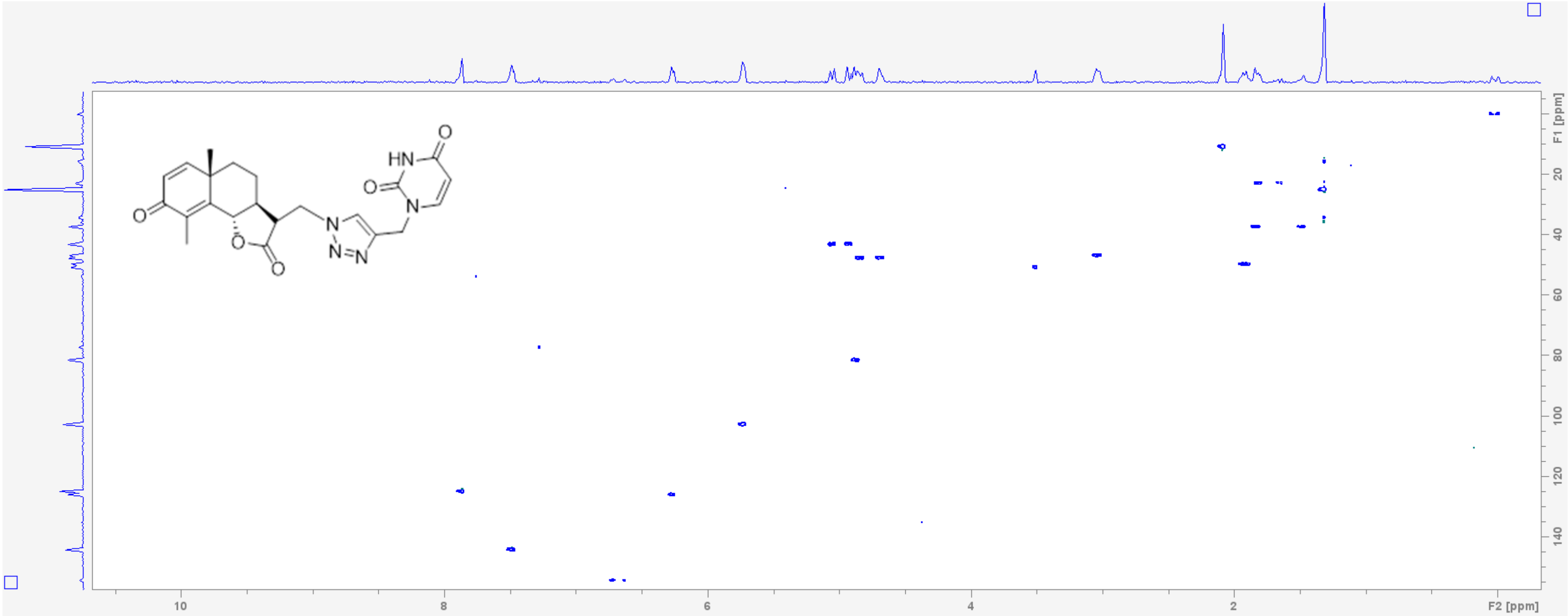

Figure S24 HMBC of compound **38**

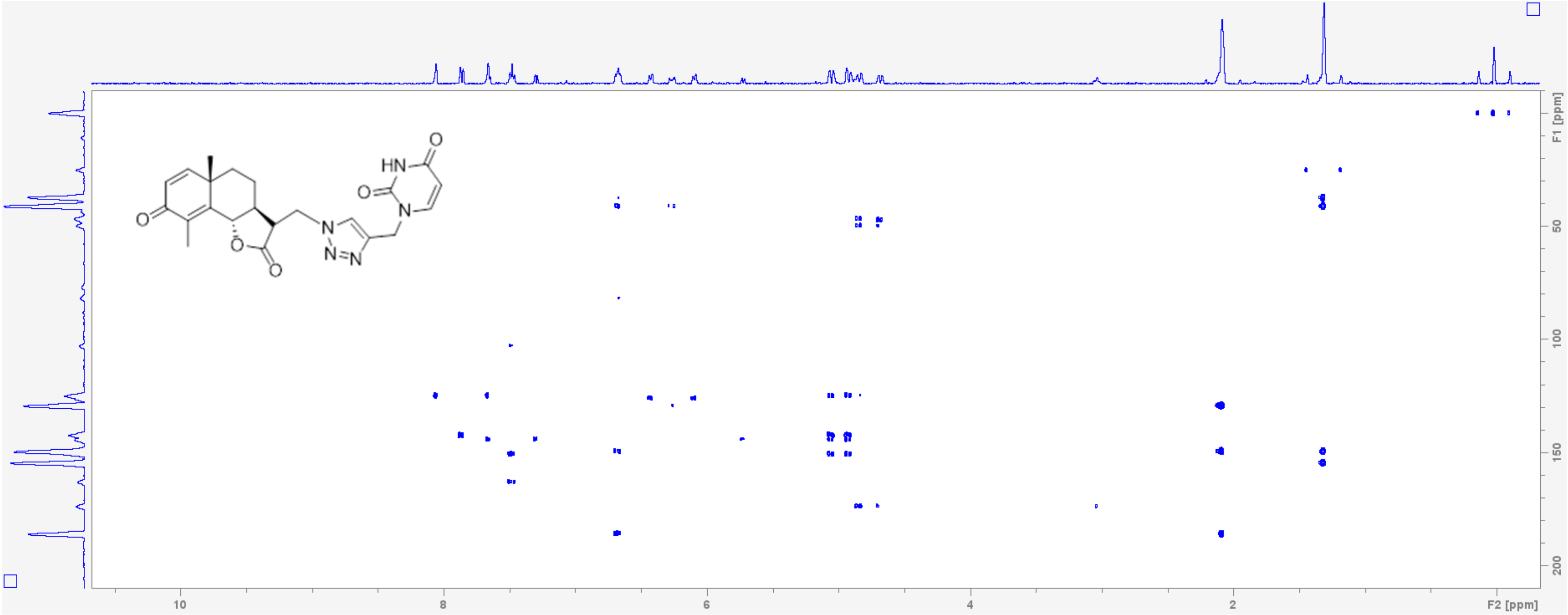

1-((1-(((5a*S*,9b*S*)-5a,9-dimethyl-2,8-dioxo-2,3,3a,4,5,5a,8,9b-octahydronaphtho[1,2-*b*]furan-3-yl)methyl)-1*H*-1,2,3-triazol-4-yl)methyl)-5-methylpyrimidine-2,4(1*H*,3*H*)-dione (**39**)

Figure S25 ^{1}H-NMR of compound **39**

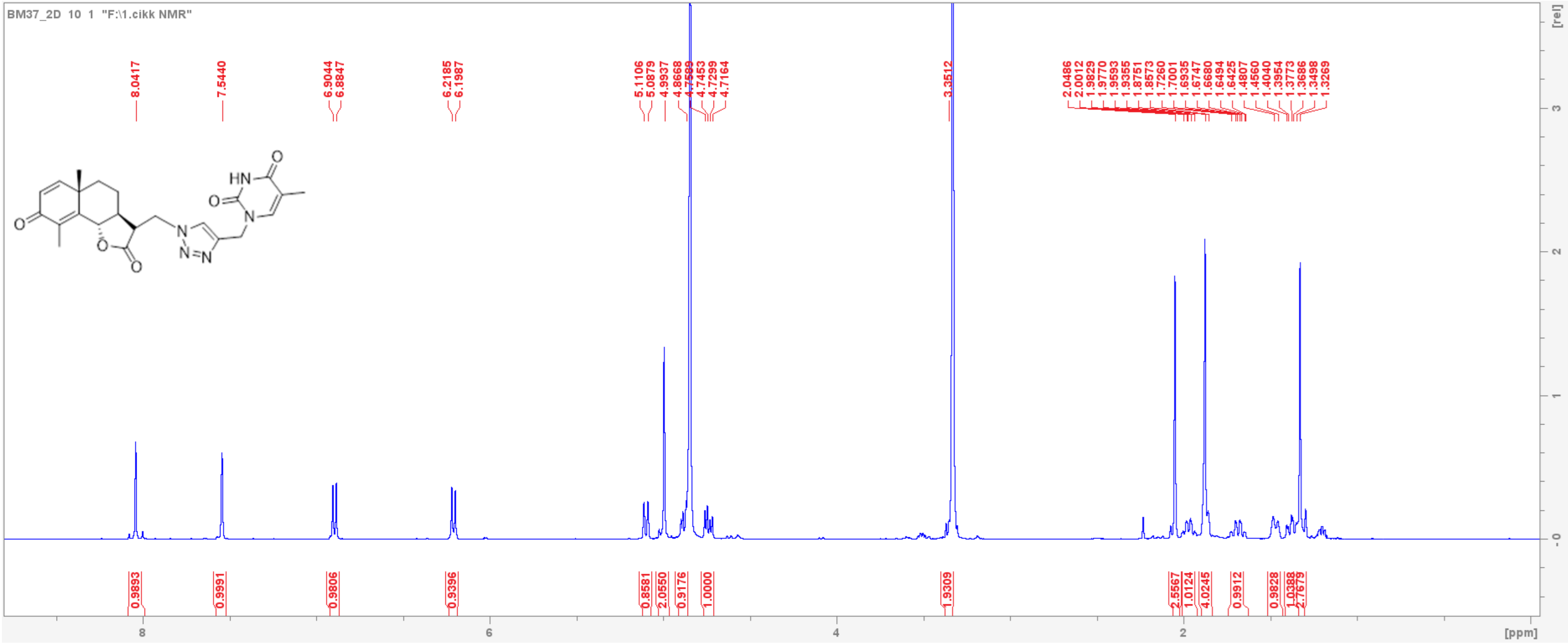

Figure S26 ^{13}C-NMR of compound **39**

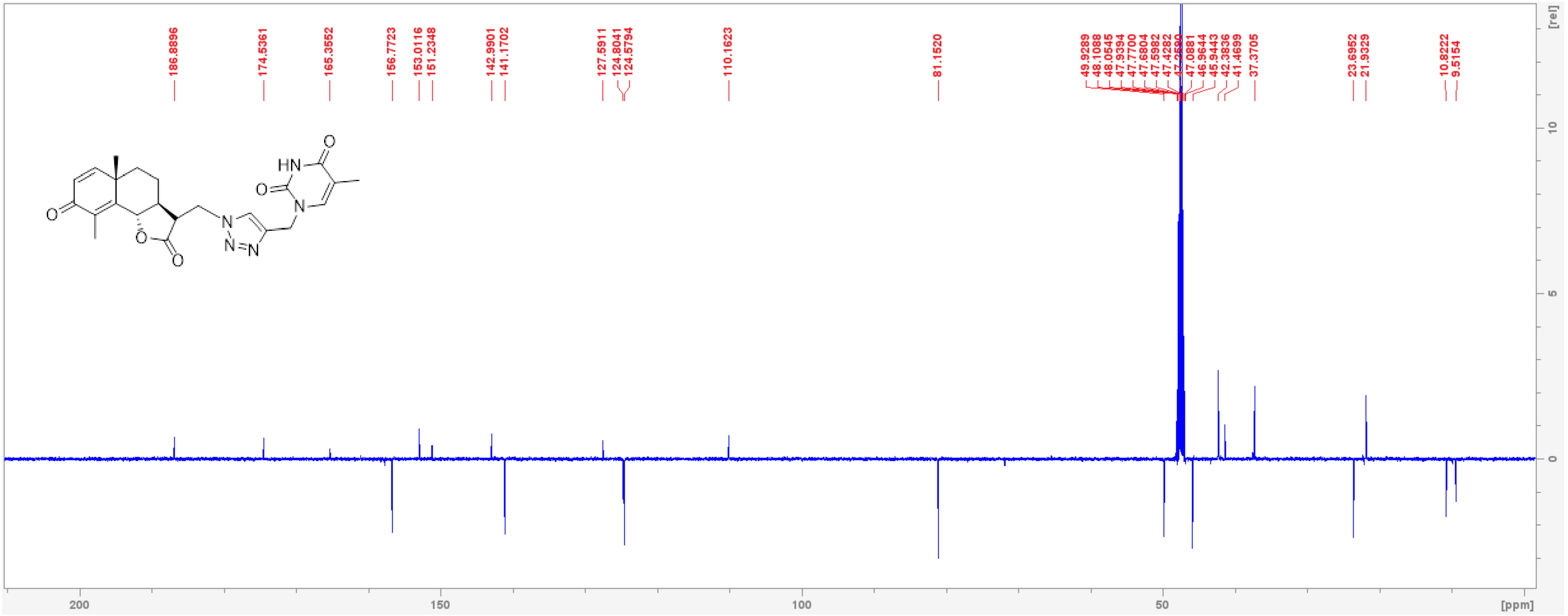

Figure S27 HSQC of compound **39**

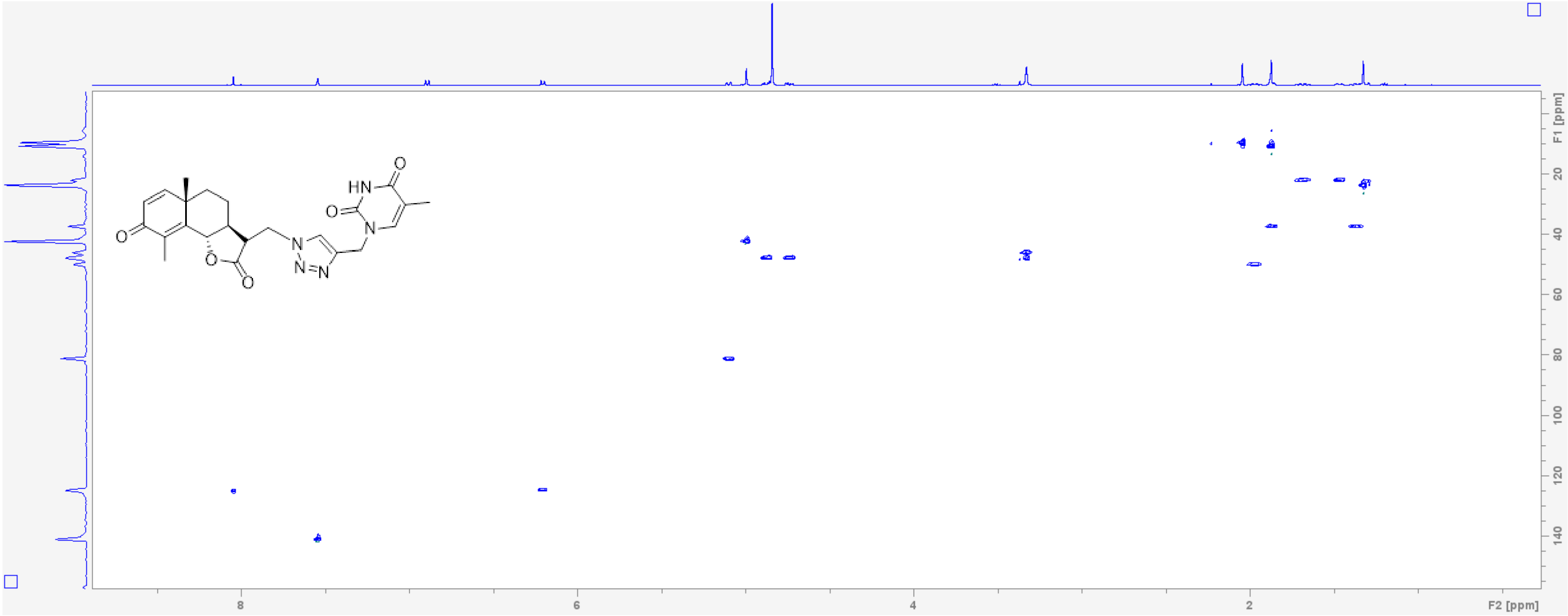

Figure S28 HSQC of compound **39**

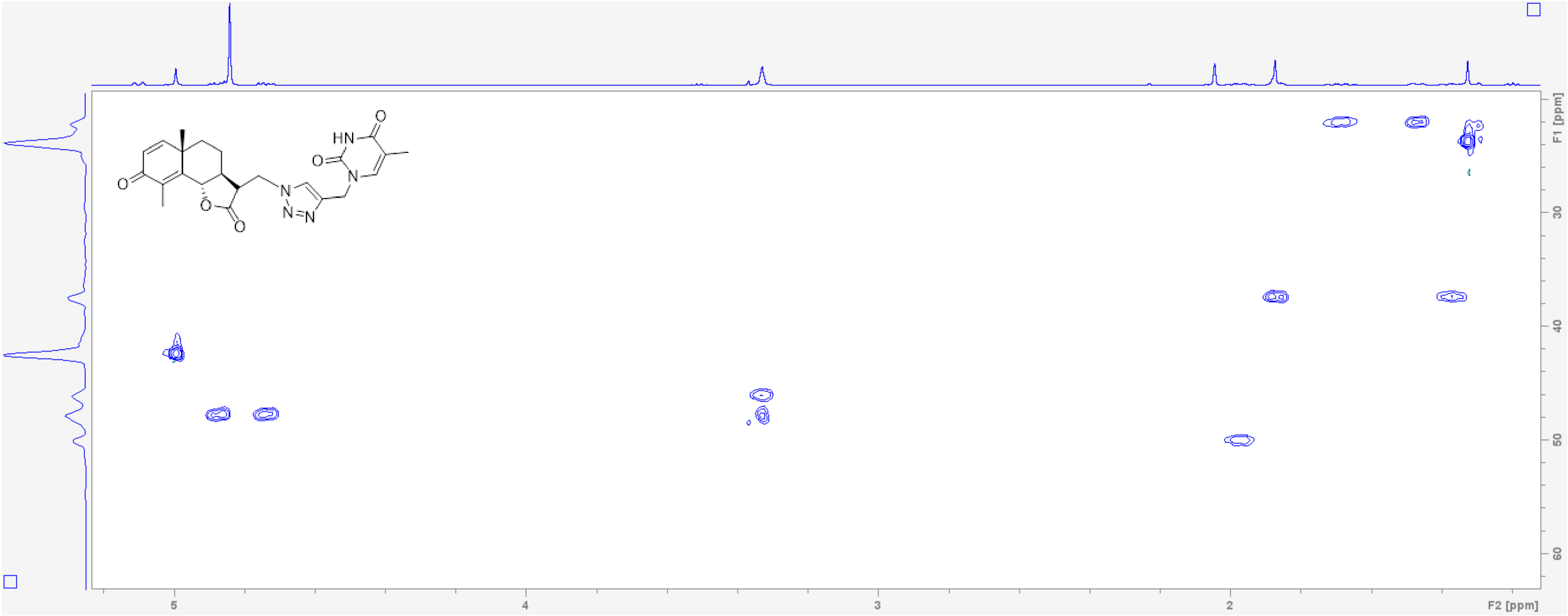

Figure S29 HMBC of compound **39**

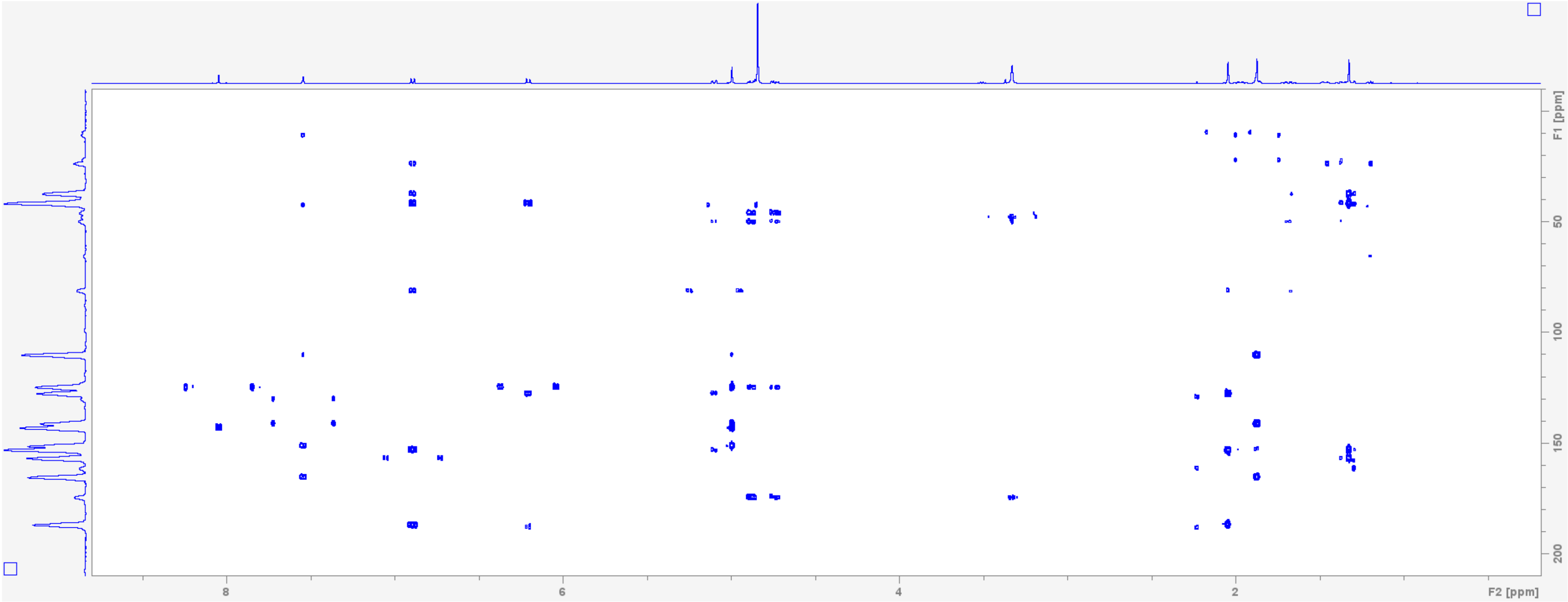

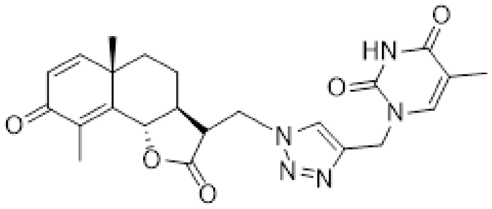

1-((1-(((5a*S*,9b*S*)-5a,9-dimethyl-2,8-dioxo-2,3,3a,4,5,5a,8,9b-octahydronaphtho[1,2-*b*]furan-3-yl)methyl)-1*H*-1,2,3-triazol-4-yl)methyl)-5-fluoropyrimidine-2,4(1*H*,3*H*)-dione (**40**)

Figure S30 ^{1}H-NMR of compound **40**

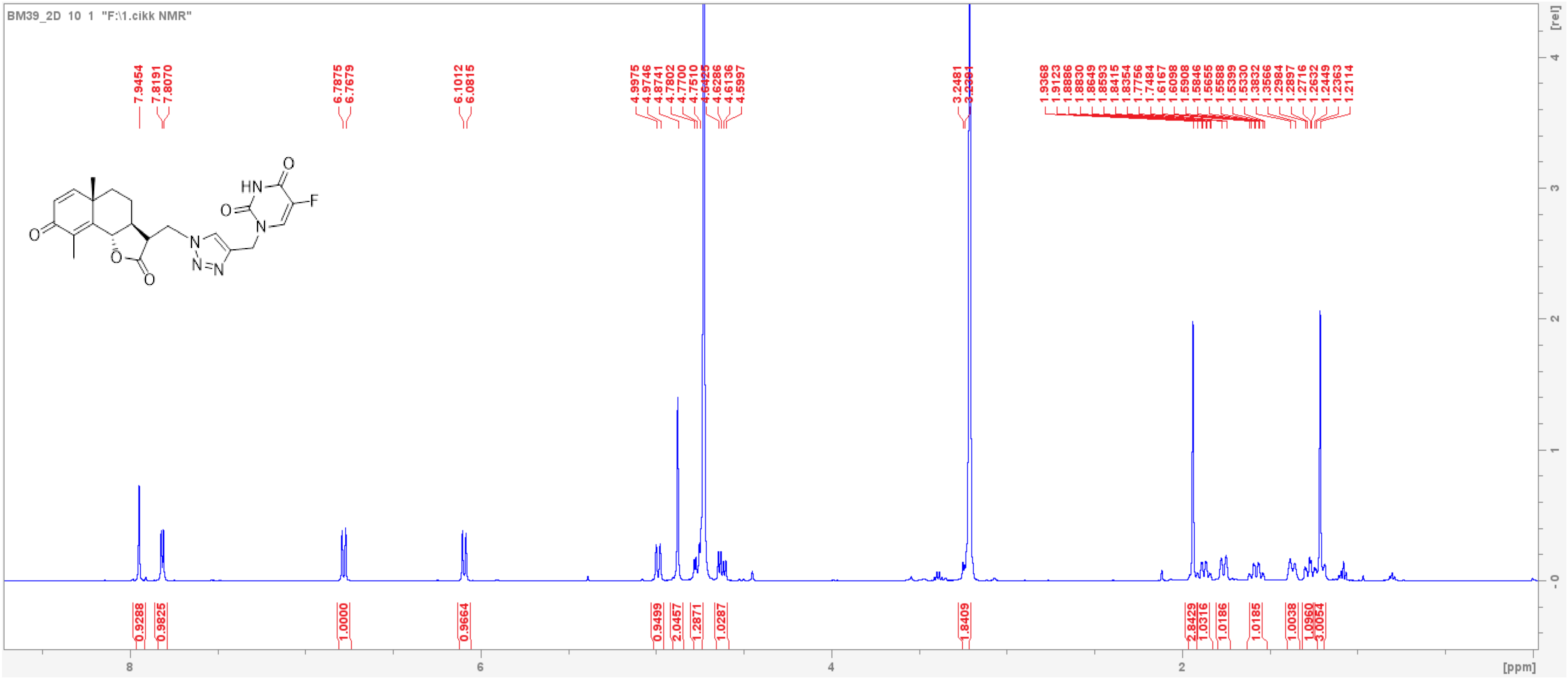

Figure S31 ^{13}C-NMR of compound **40**

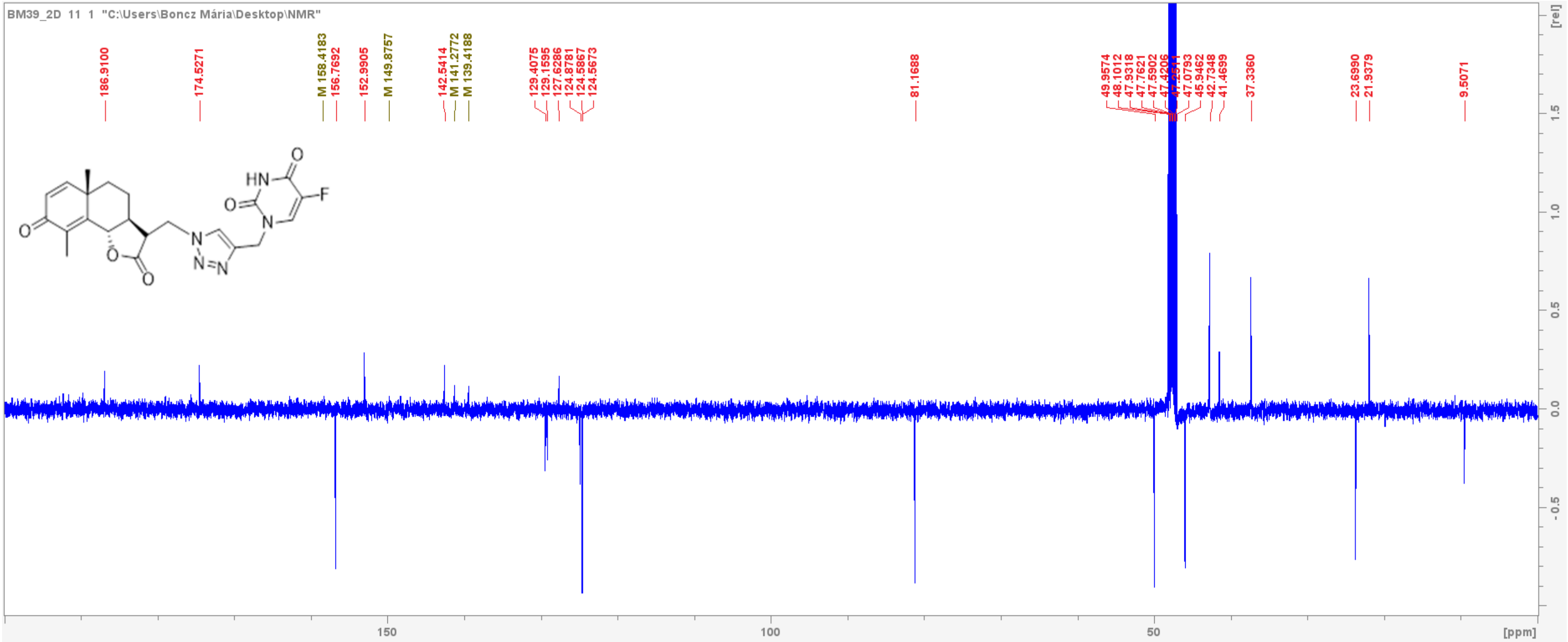

Figure S32 HSQC of compound **40**

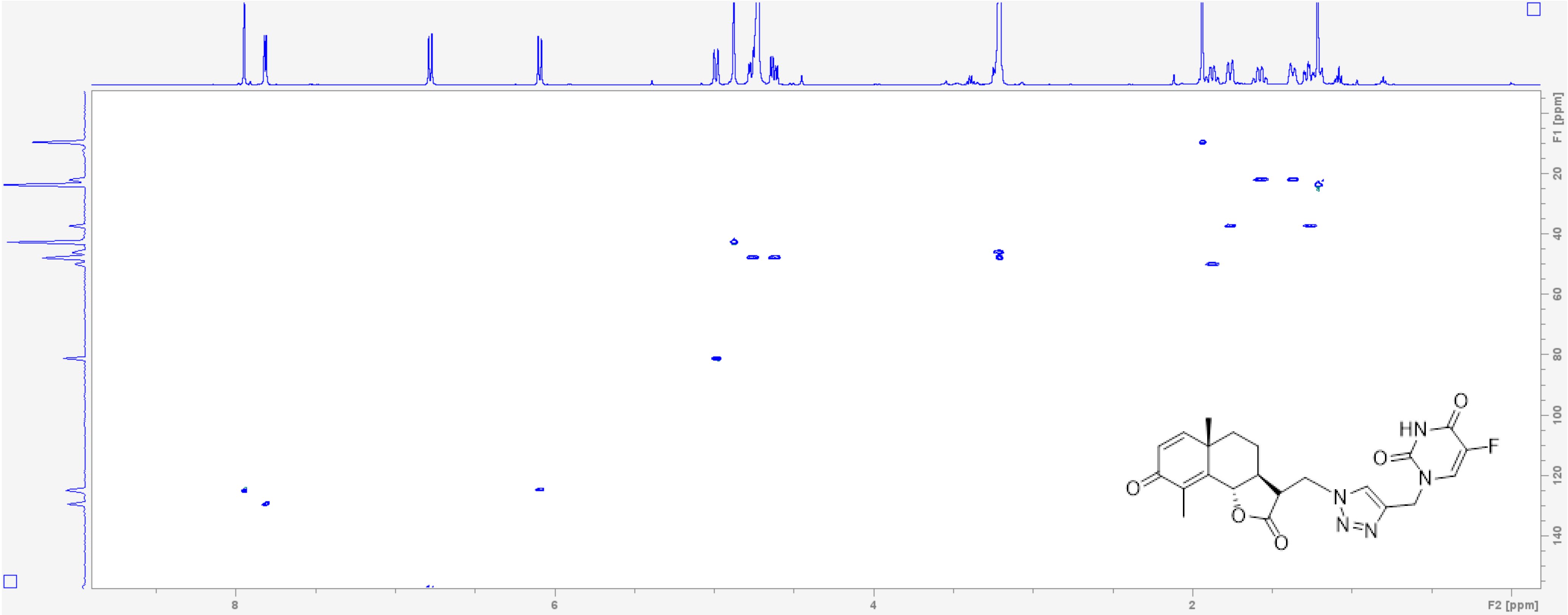

Figure S33 HMBC of compound **40**

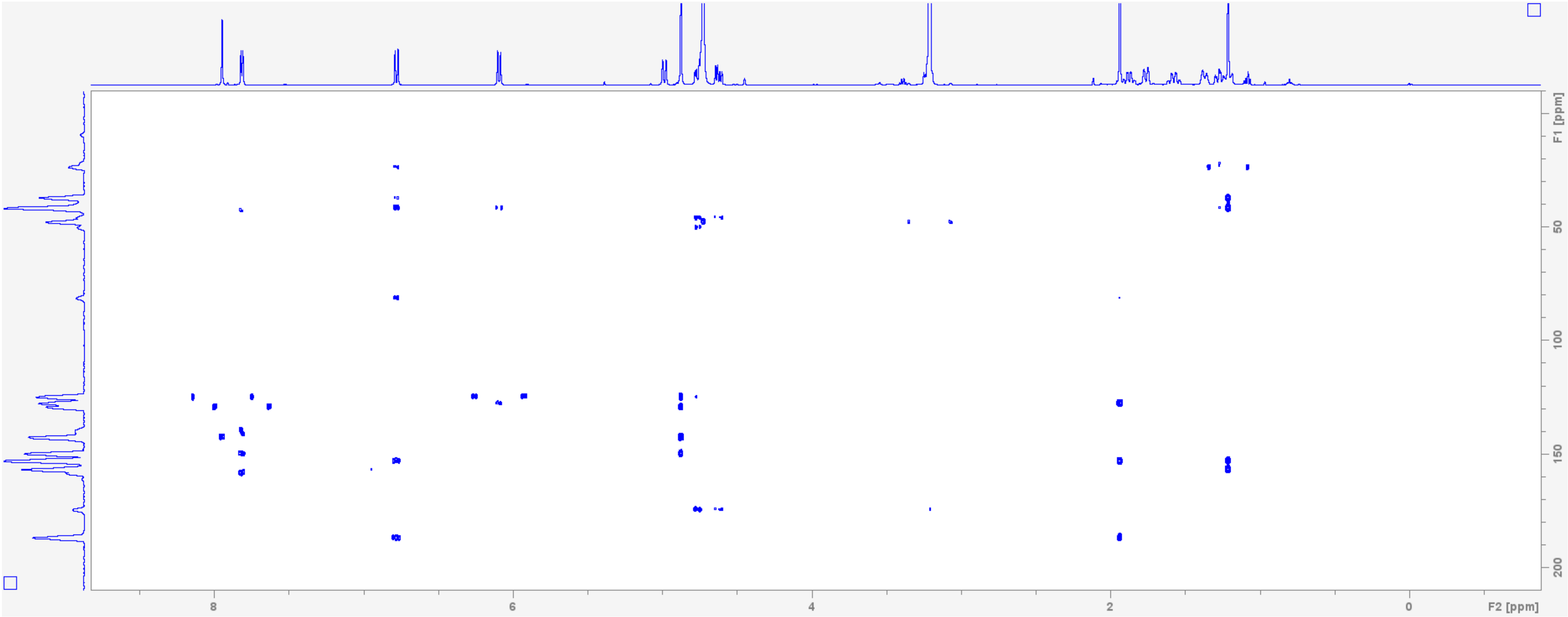

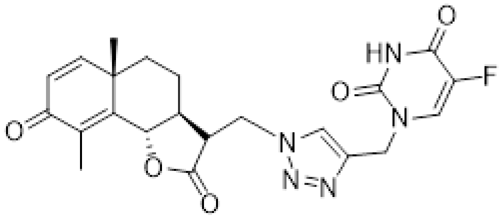

Figure S34 HMBC of compound **40**

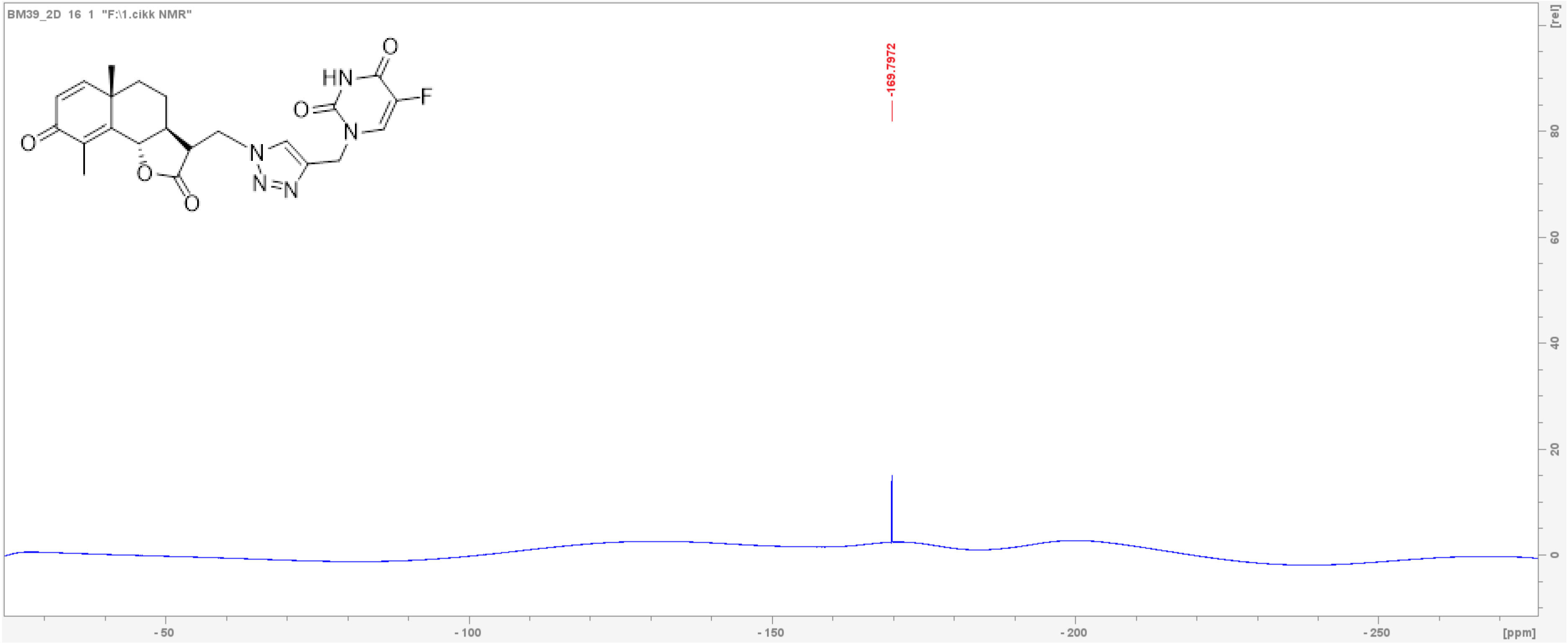

1-((1-(((5aS,9bS)-5a,9-dimethyl-2,8-dioxo-2,3,3a,4,5,5a,8,9b-octahydronaphtho[1,2-*b*]furan-3-yl)methyl)-1*H*-1,2,3-triazol-4-yl)methyl)-5-iodopyrimidine-2,4(1*H*,3*H*)-dione (**41**)

Figure S35 ^{1}H-NMR of compound **41**

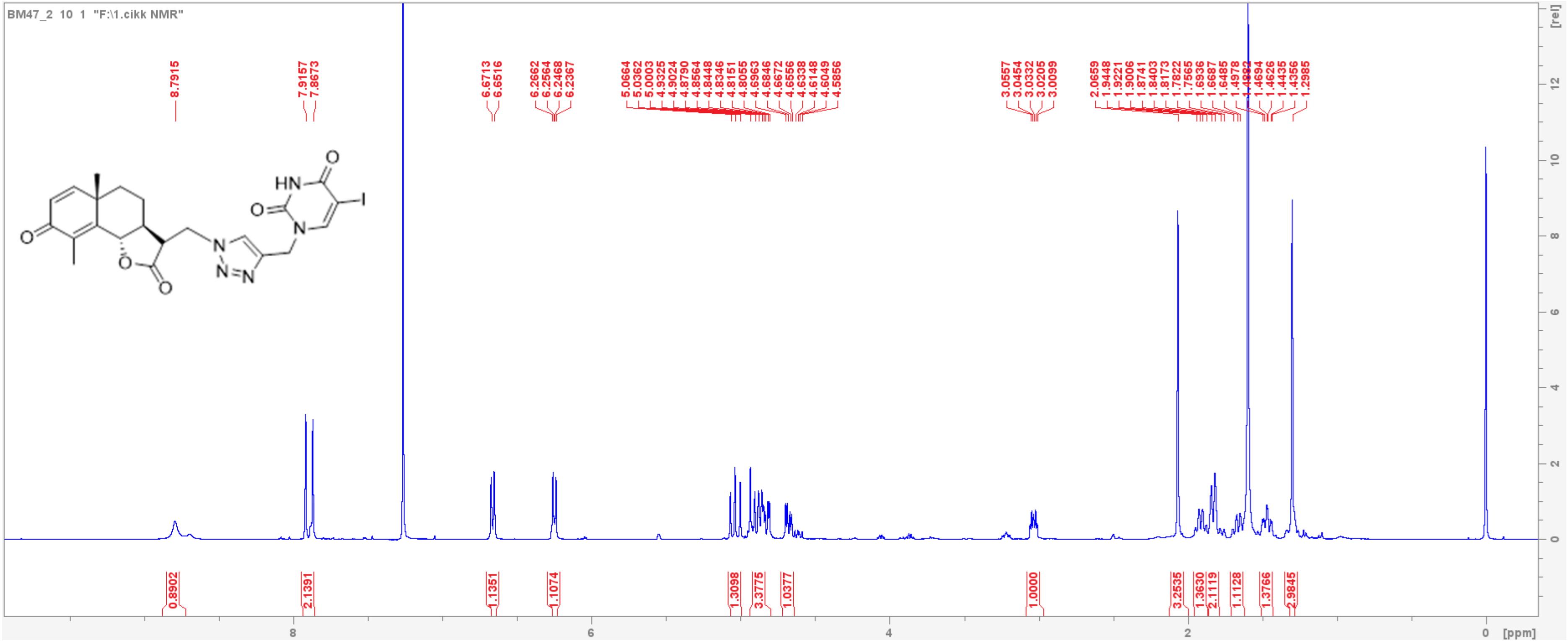

Figure S36 ^{13}C-NMR of compound **41**

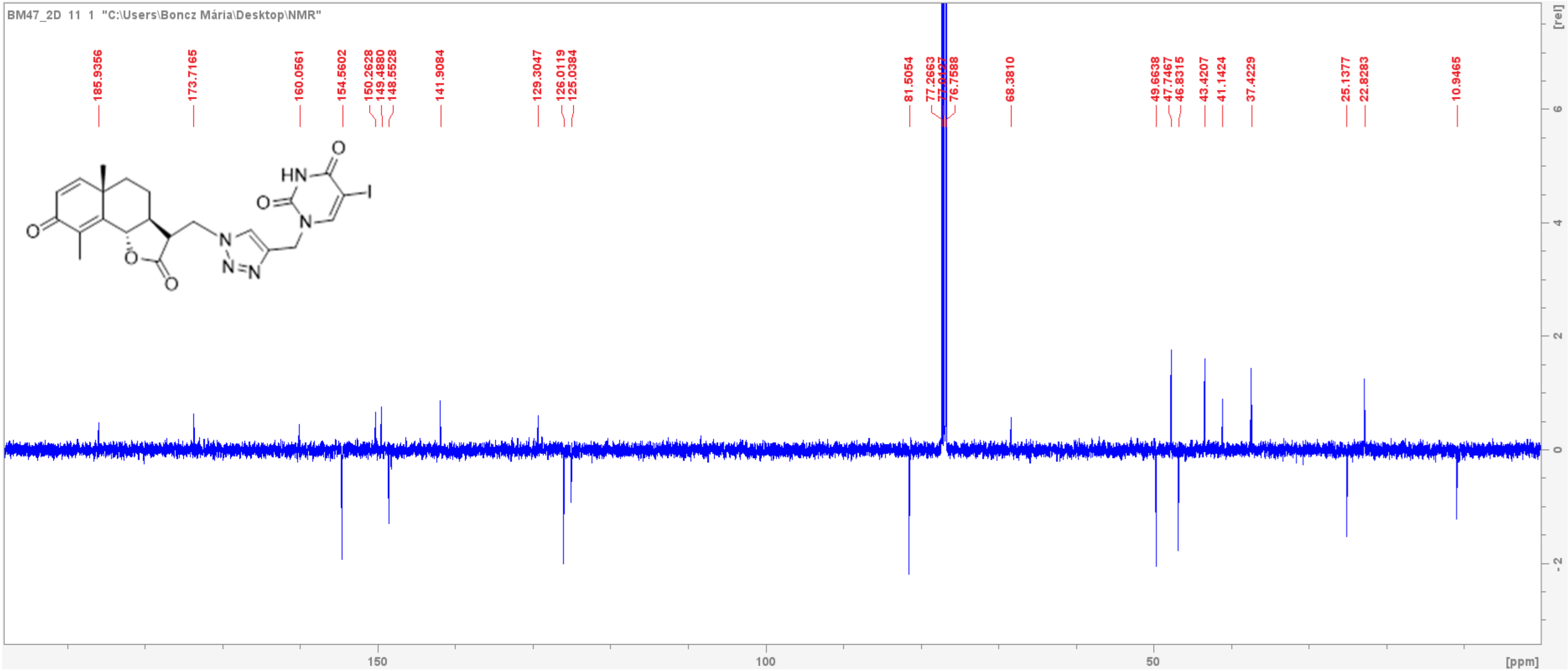

7-((1-(((5aS,9bS)-5a,9-dimethyl-2,8-dioxo-2,3,3a,4,5,5a,8,9b-octahydronaphtho[1,2-*b*]furan-3-yl)methyl)-1*H*-1,2,3-triazol-4-yl)methyl)-1,3-dimethyl-3,7-dihydro-1*H*-purine-2,6-dione (**42**)

Figure S37 ^{1}H of compound **42**

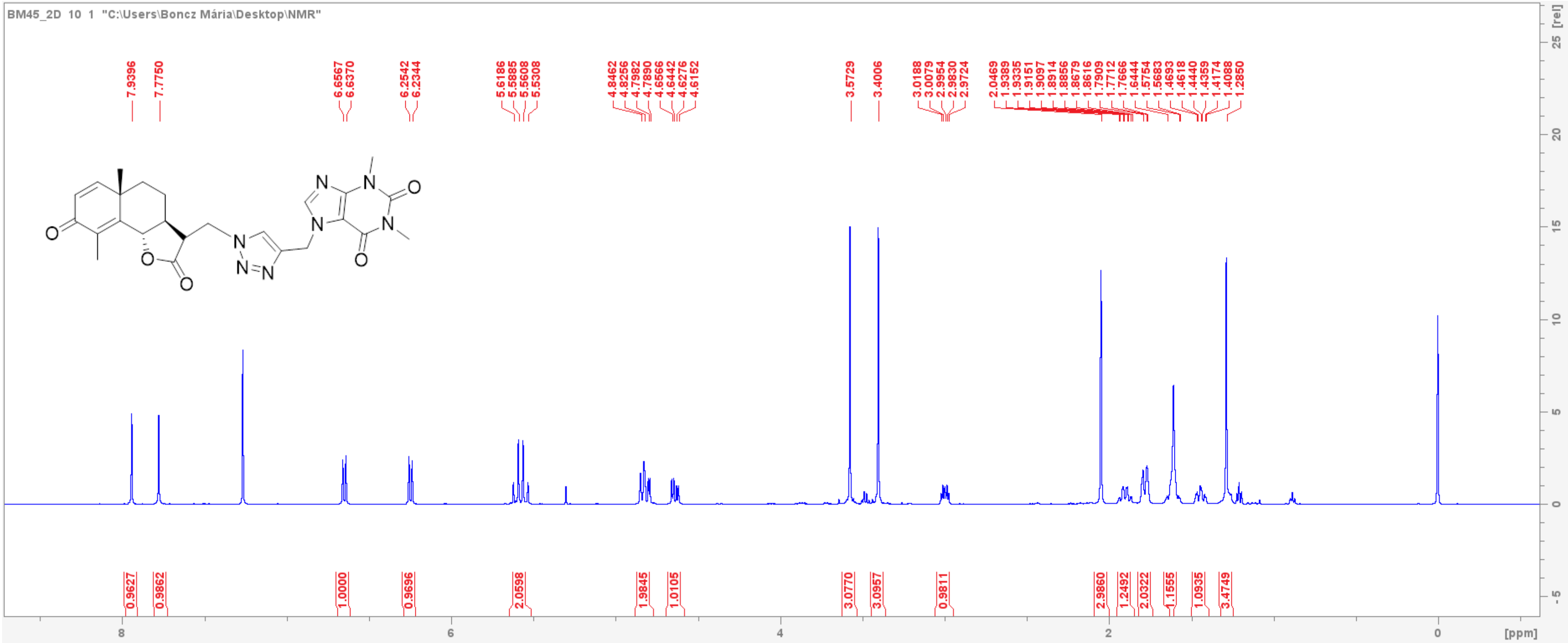

Figure S38 ^{13}C of compound **42**

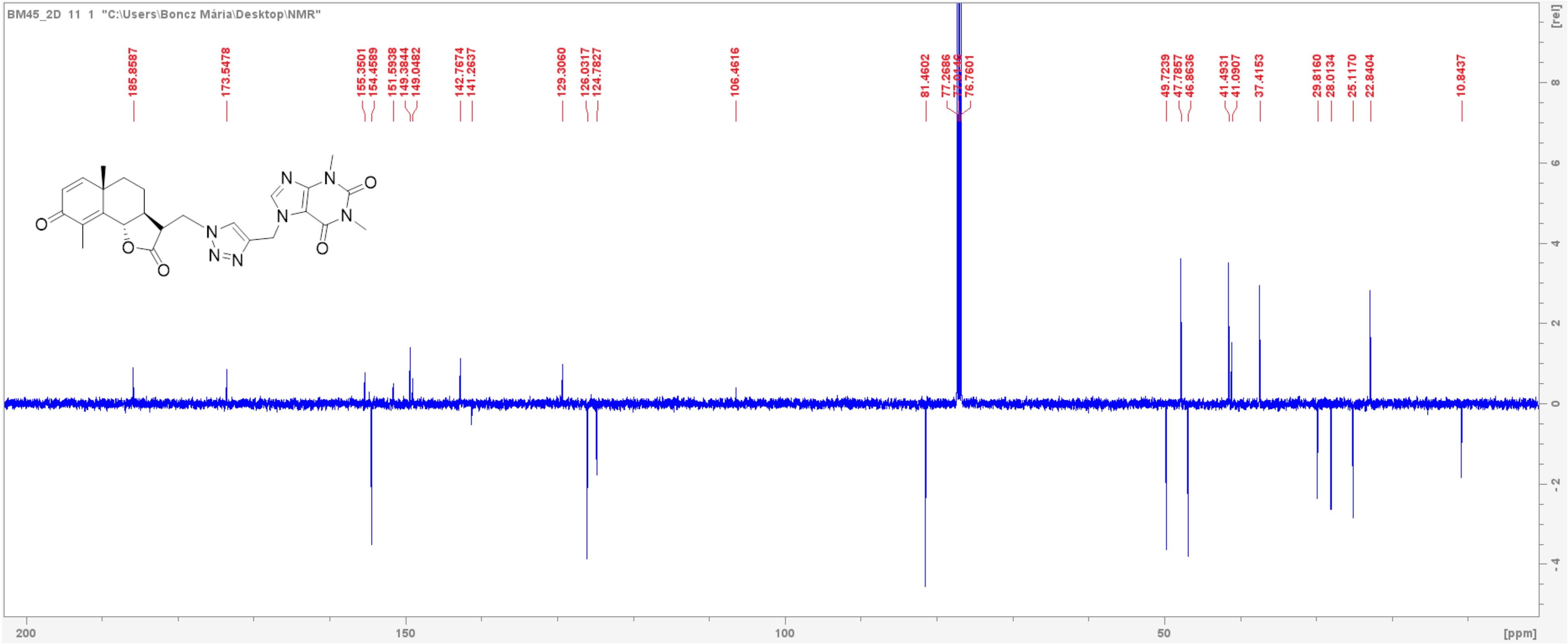

Figure S39 HSQC of compound **42**

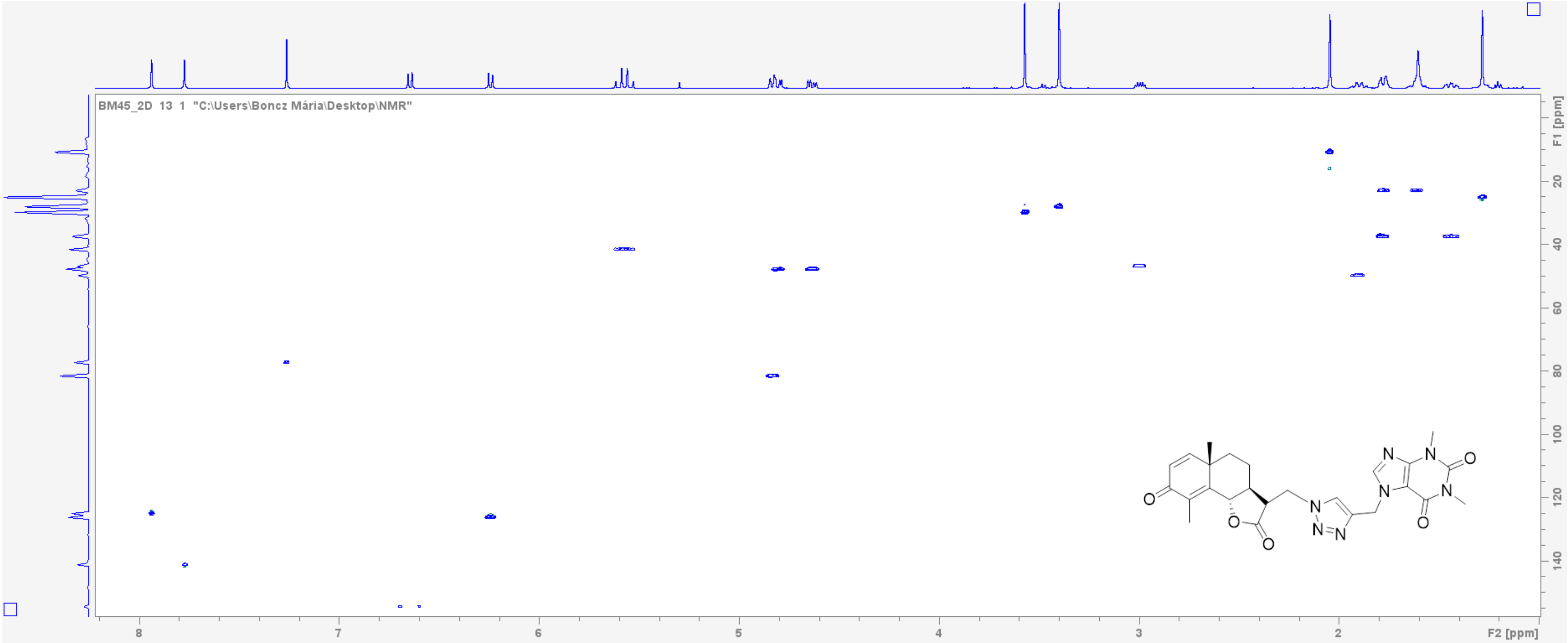

Figure S40 HMBC of compound **42**

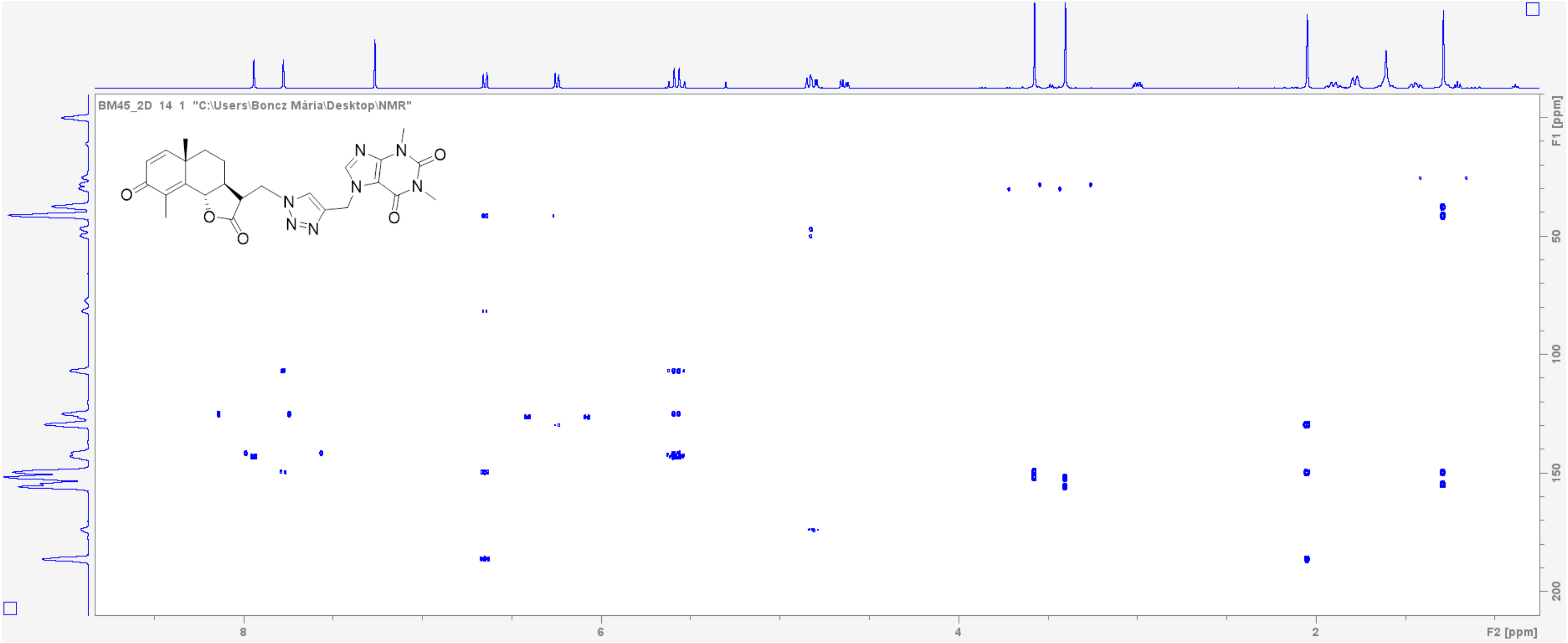

(5a*S*,9b*S*)-3-((4-((6-amino-9*H*-purin-9-yl)methyl)-1*H*-1,2,3-triazol-1-yl)methyl)-5a,9-dimethyl-3a,5,5a,9b-tetrahydronaphtho[1,2-*b*]furan-2,8(3*H*,4*H*)-dione, (**43**)

Figure S41 ^{1}H-NMR of compound **43**

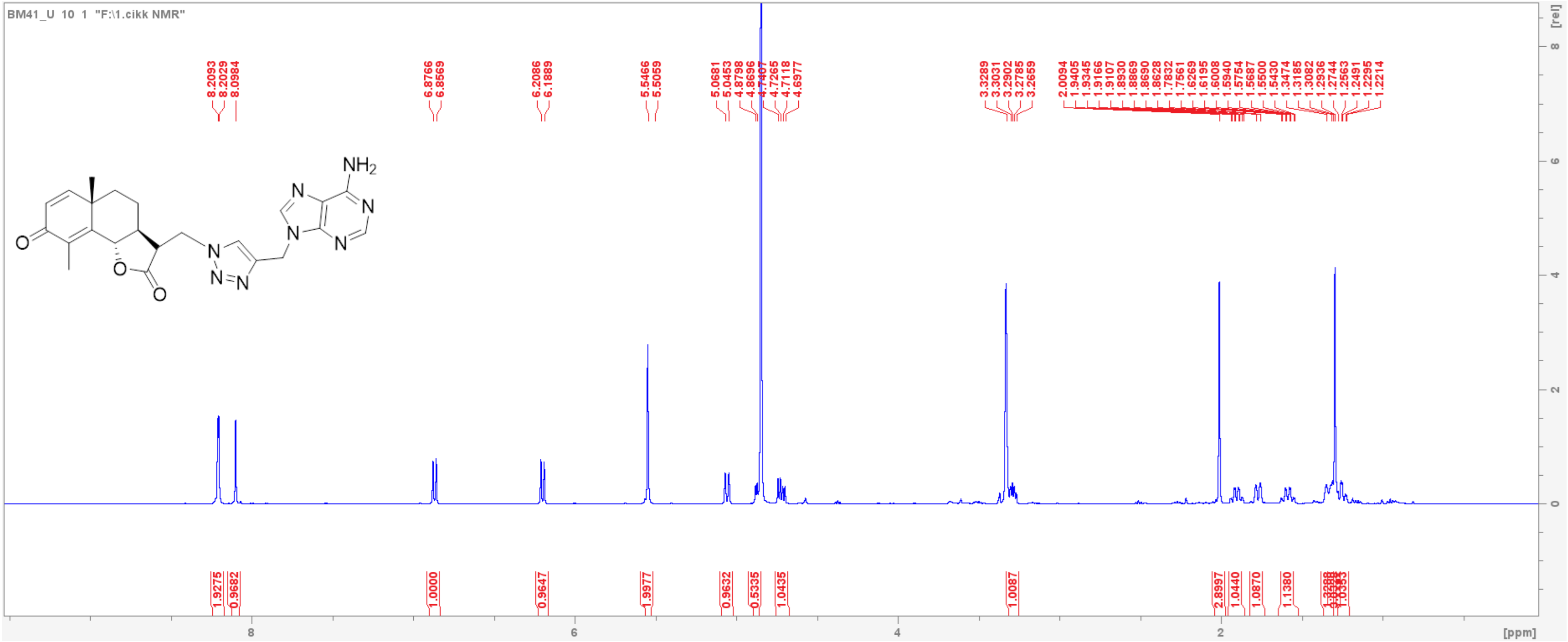

Figure S42 ^{13}C-NMR of compound **43**

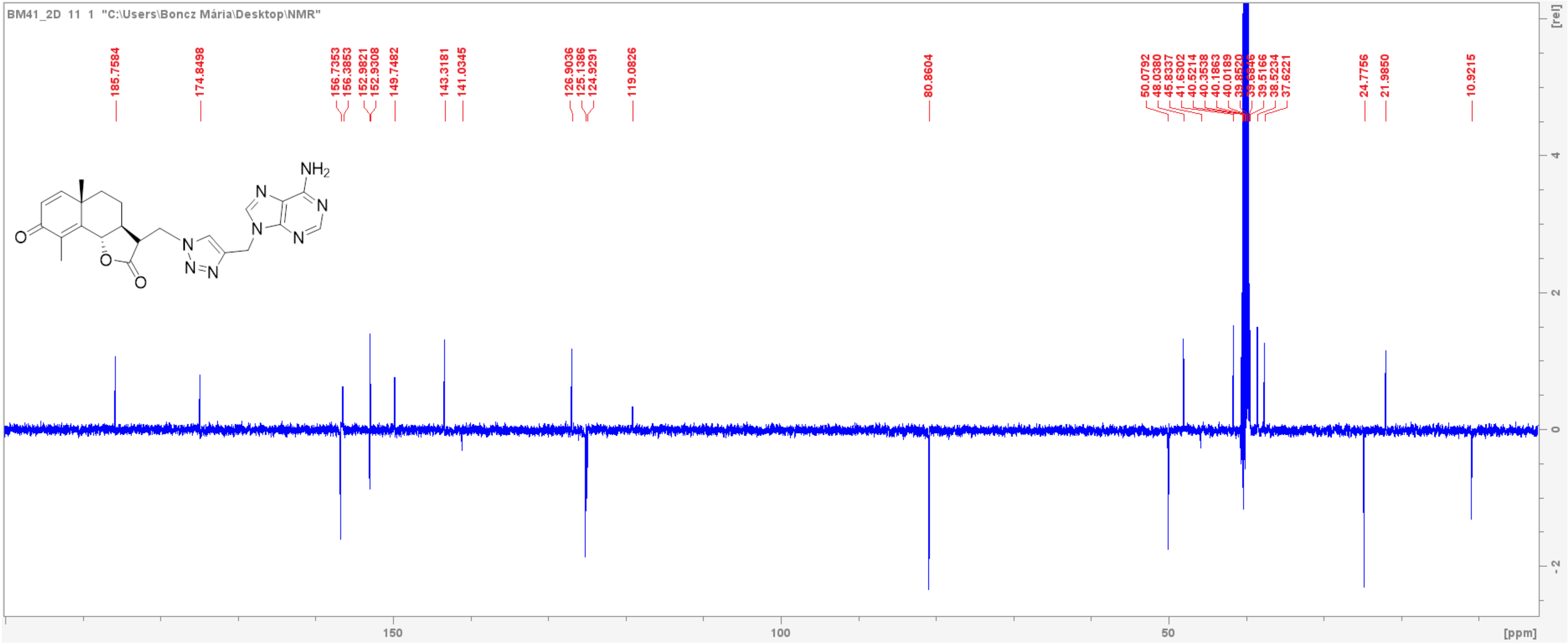

Figure S43 HSQC of compound **43**

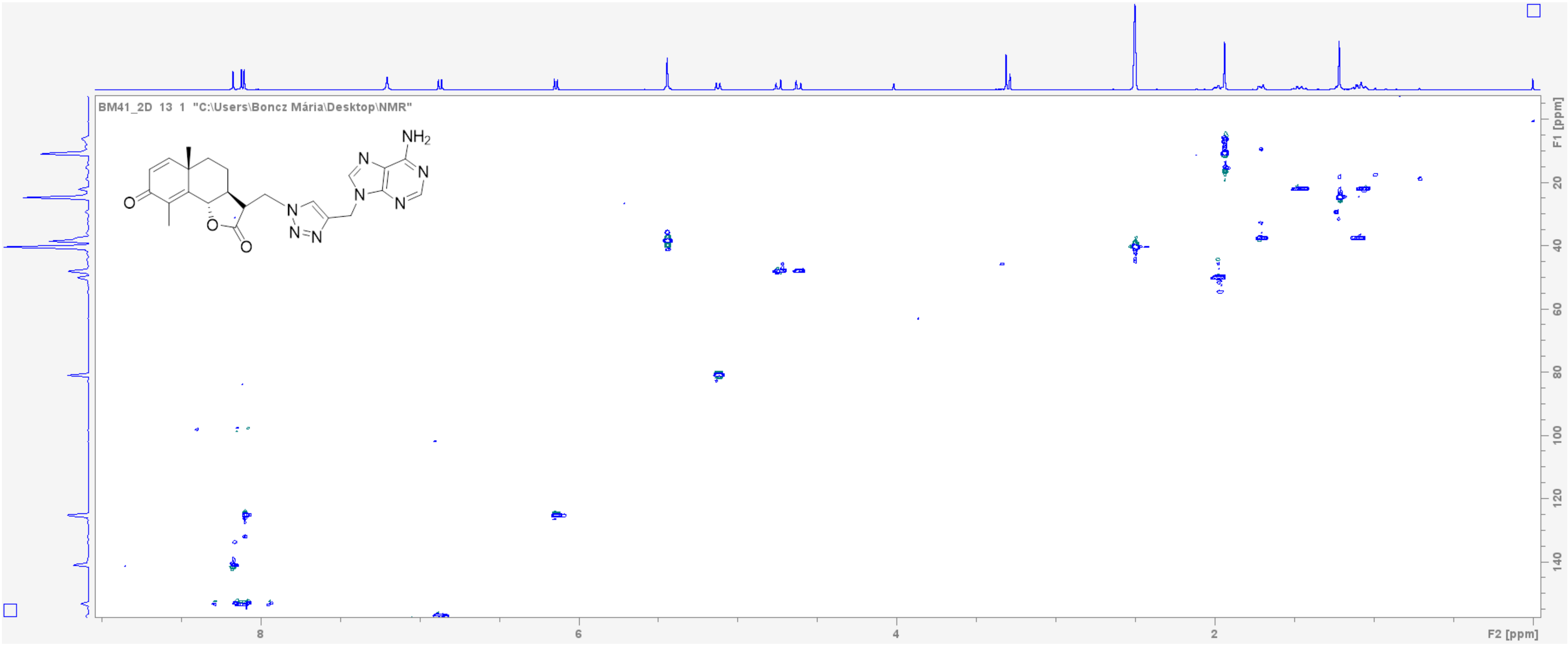

Figure S44 HMBC of compound **43**

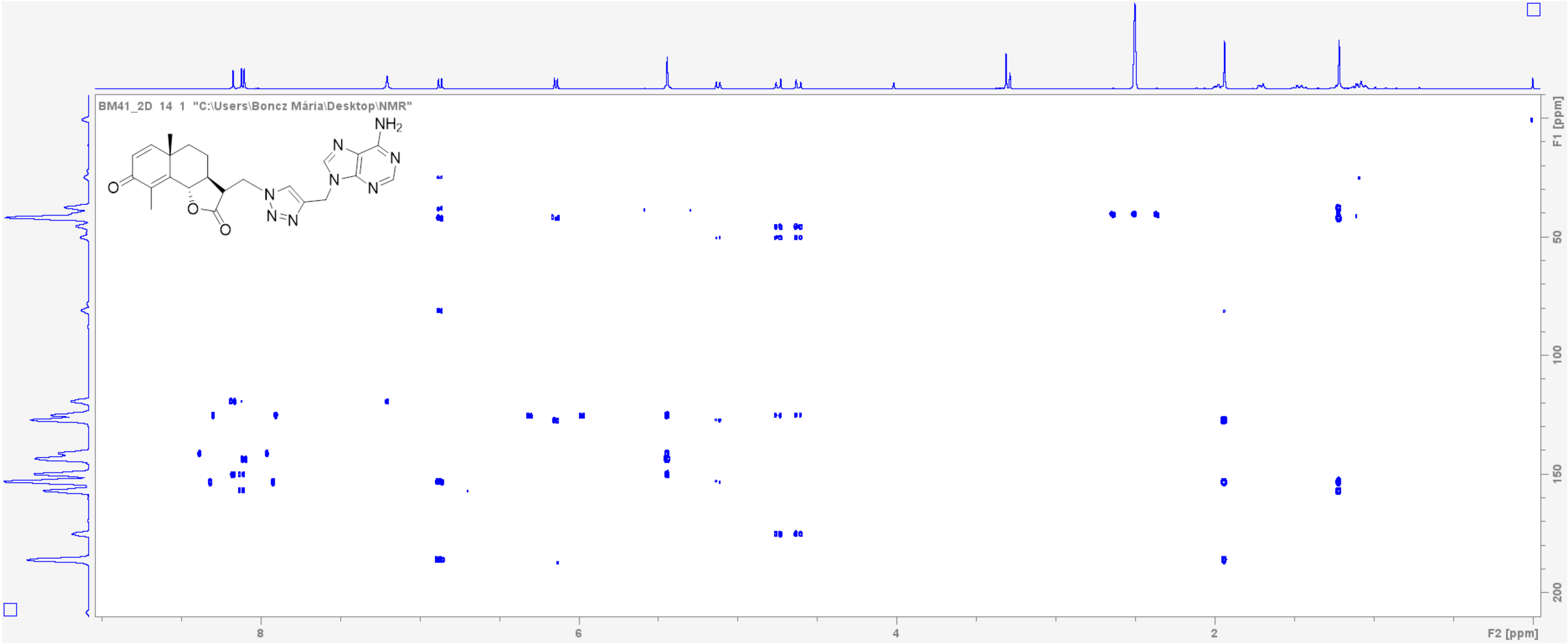

1-((1-(((5a*S*,9b*S*)-5a,9-dimethyl-2,8-dioxo-2,3,3a,4,5,5a,8,9b-octahydronaphtho[1,2-*b*]furan-3-yl)methyl)-1*H*-1,2,3-triazol-4-yl)methyl)indoline-2,3-dione (**44**)

Figure S45 ^{1}H-NMR of compound **44**

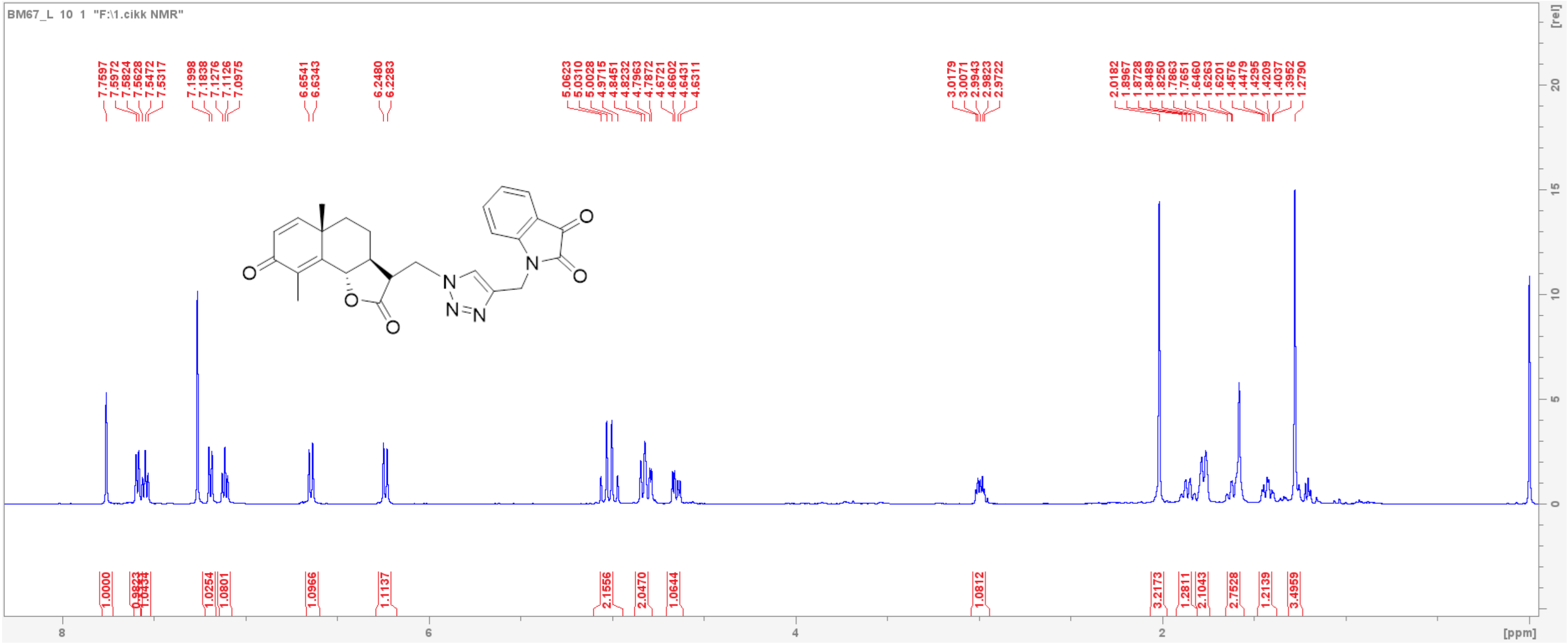

Figure S46 ^{13}C-NMR of compound **44**

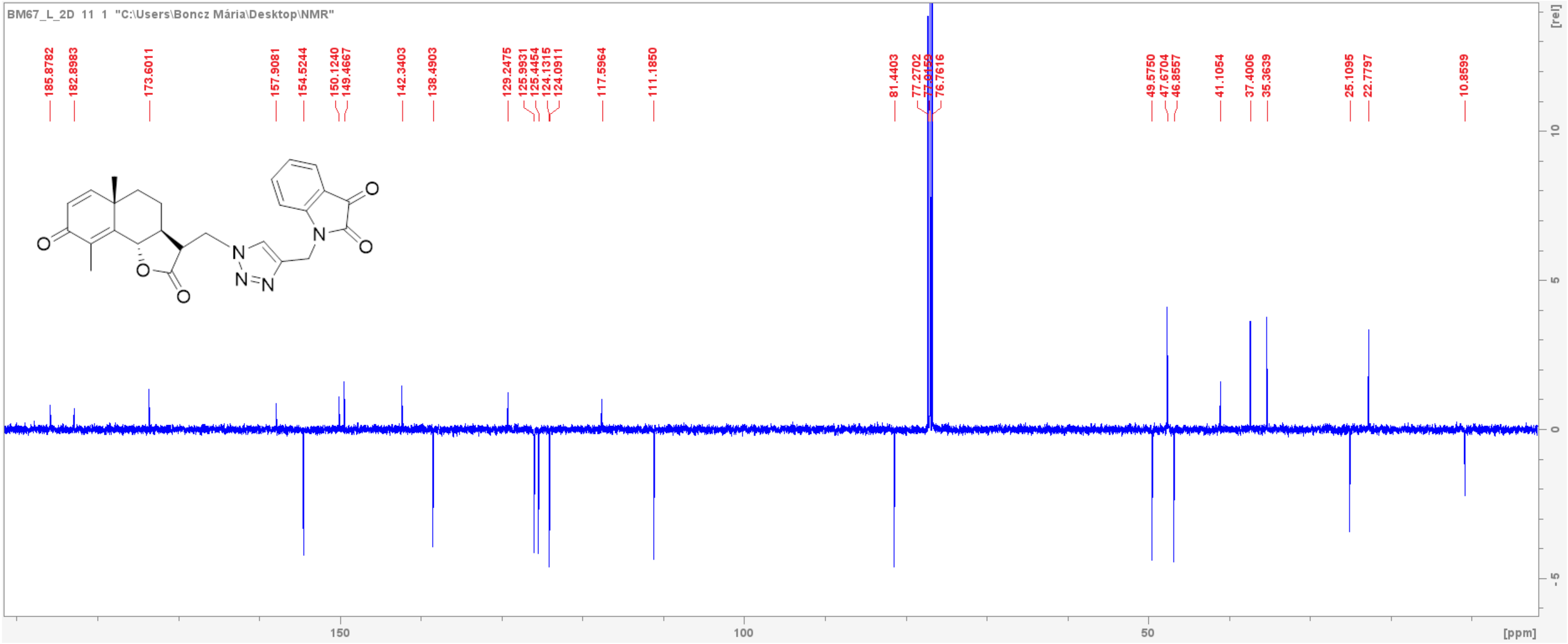

(5a*S*,9b*S*)-3-((4-((1*H*-indol-1-yl)methyl)-1*H*-1,2,3-triazol-1-yl)methyl)-5a,9-dimethyl-3a,5,5a,9b-tetrahydronaphtho[1,2-*b*]furan-2,8(3*H*,4*H*)-dione (**45**)

Figure S47 ^{1}H-NMR of compound **45**

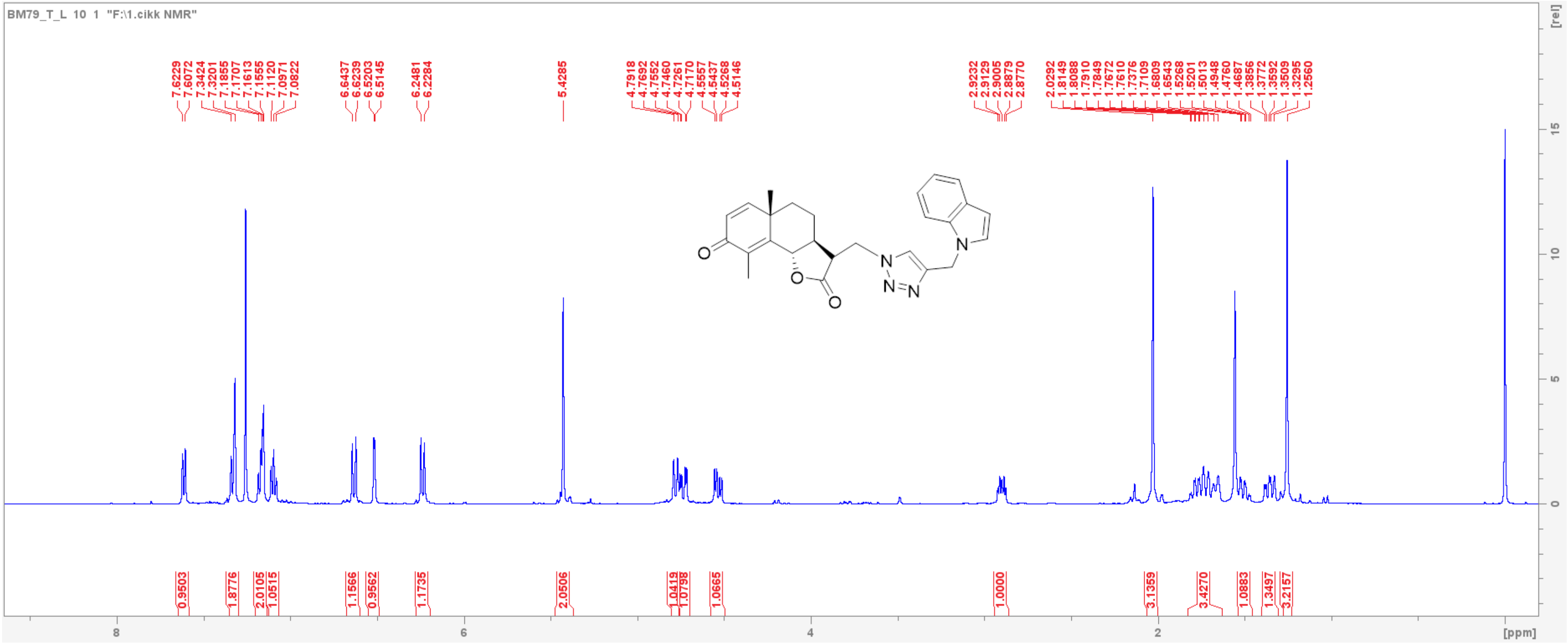

Figure S48 ^{13}C-NMR of compound **45**

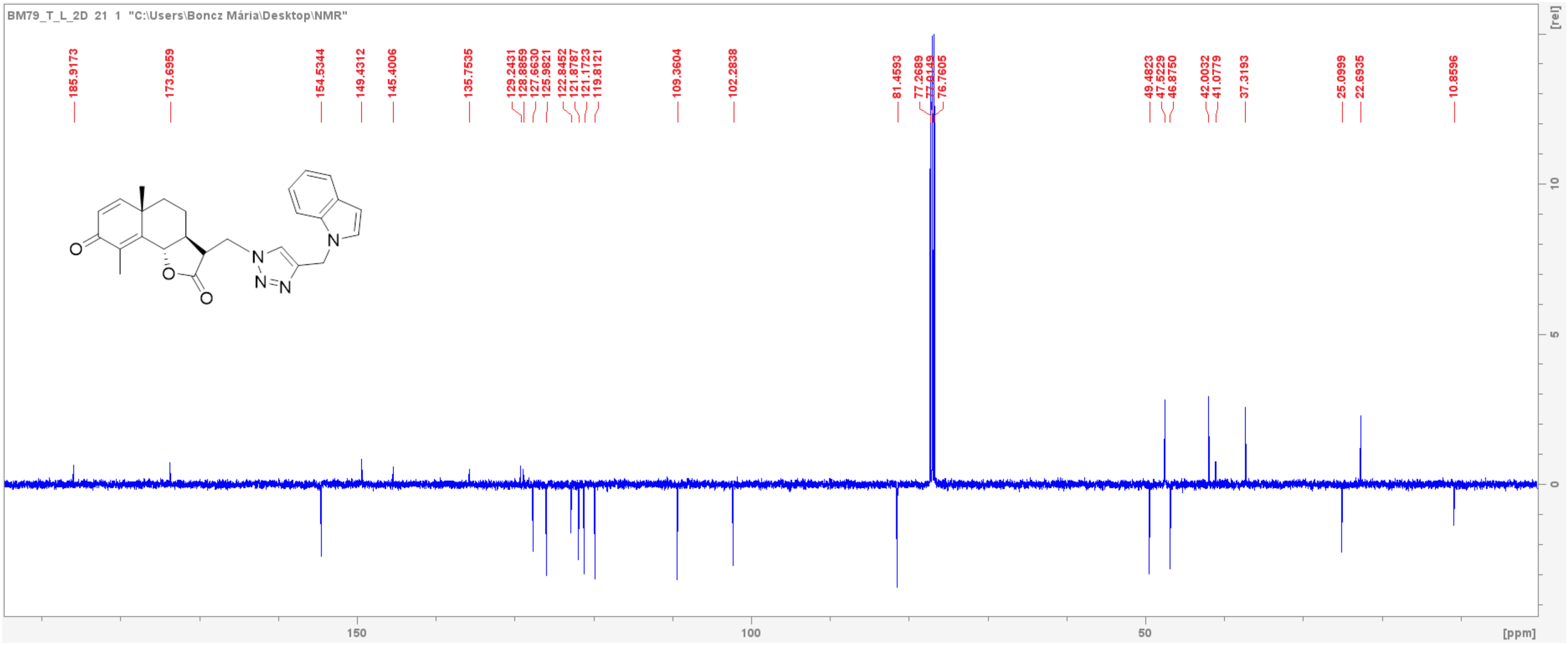

Figure S49 HSQC of compound **45**

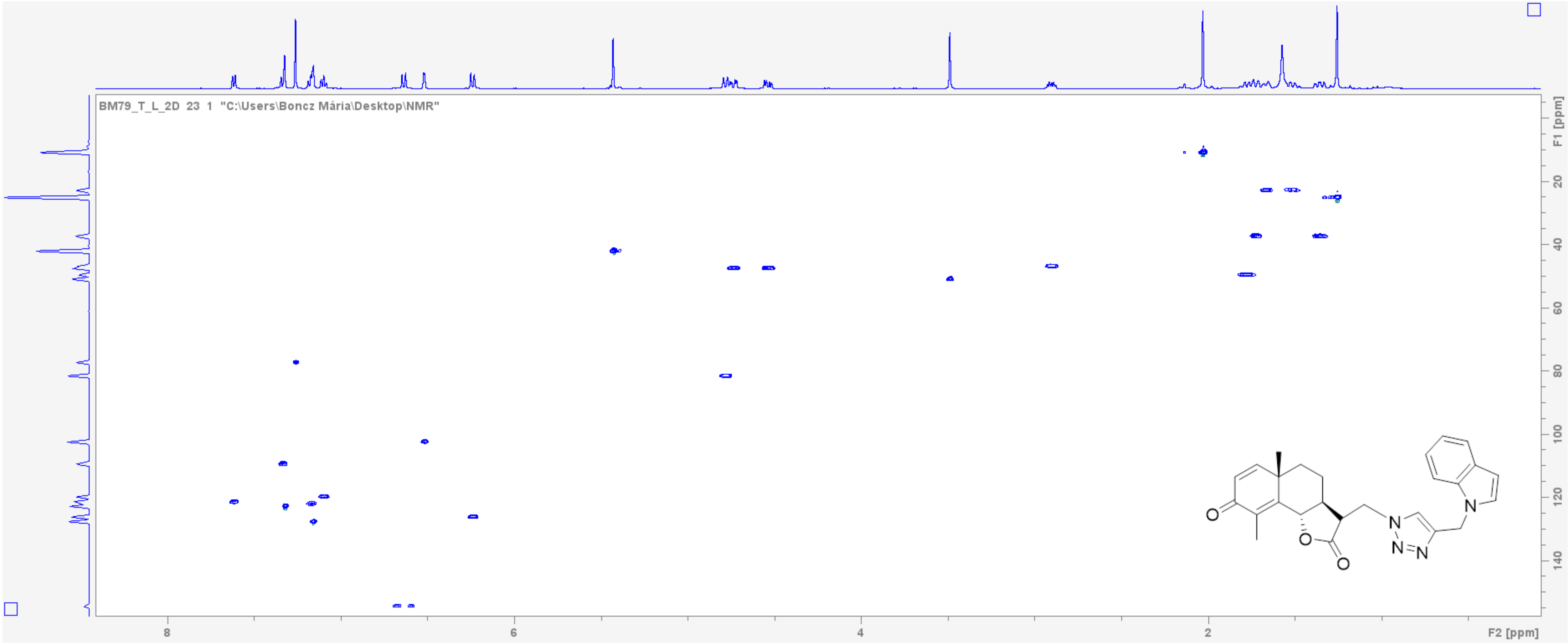

Figure S50 HMBC of compound **45**

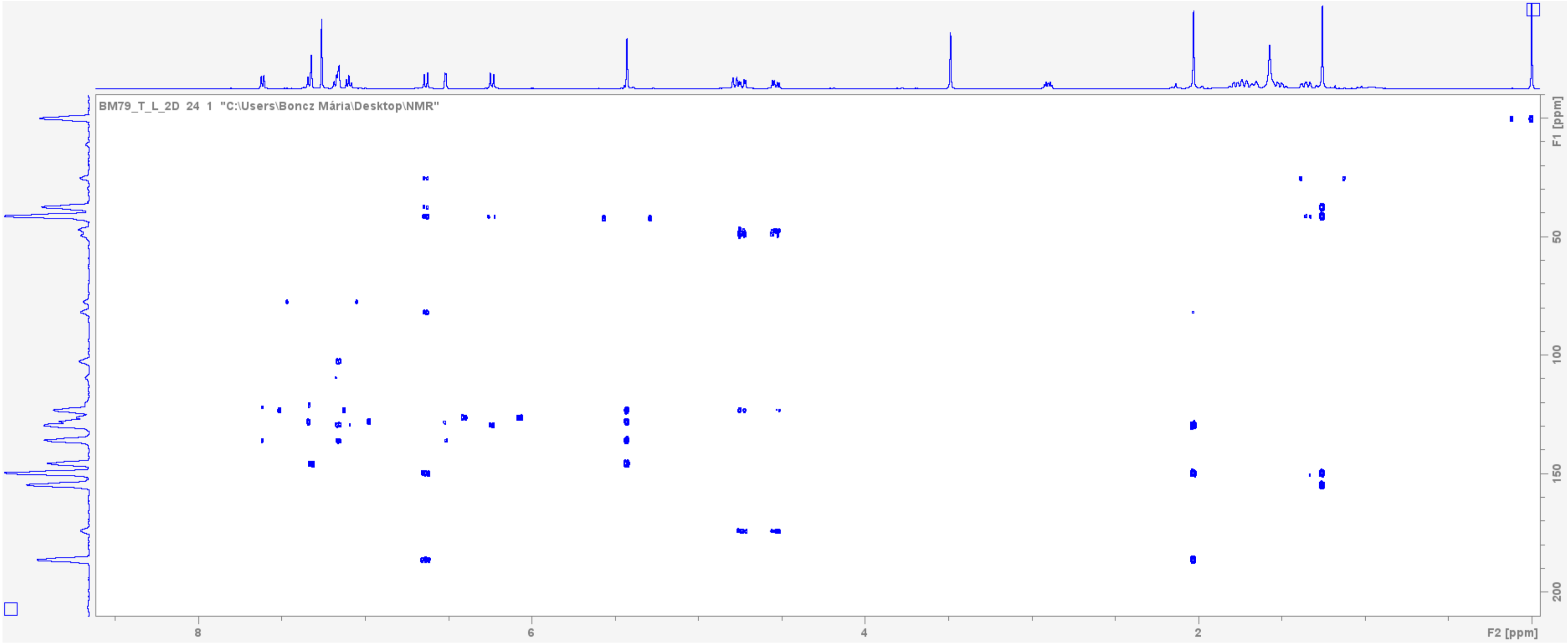

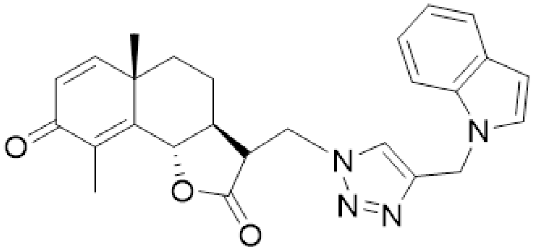

(5a*S*,9b*S*)-3-((4-((1*H*-benzo[*d*]imidazol-1-yl)methyl)-1*H*-1,2,3-triazol-1-yl)methyl)-5a,9-dimethyl-3a,5,5a,9b-tetrahydronaphtho[1,2-*b*]furan-2,8(3*H*,4*H*)-dione (**46**)

Figure S51 ^{1}H-NMR of compound **46**

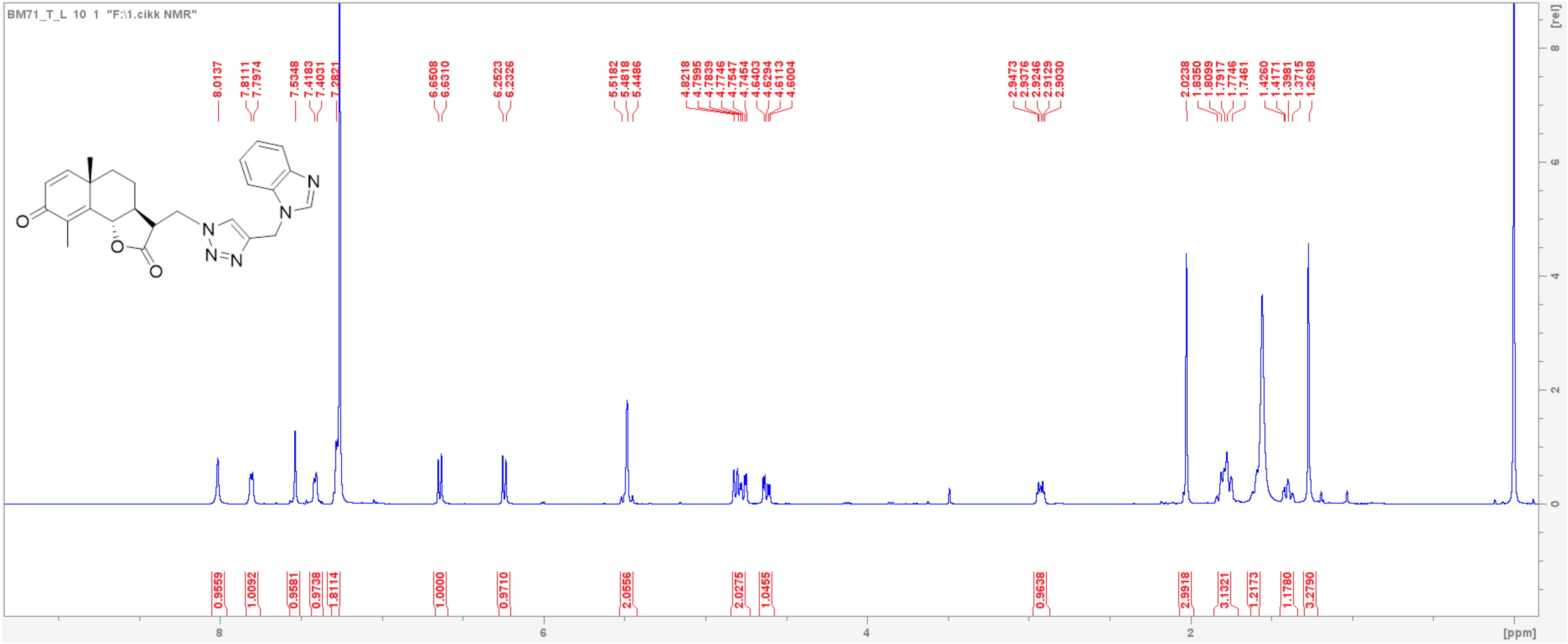

Figure S52 ^{13}C-NMR of compound **46**

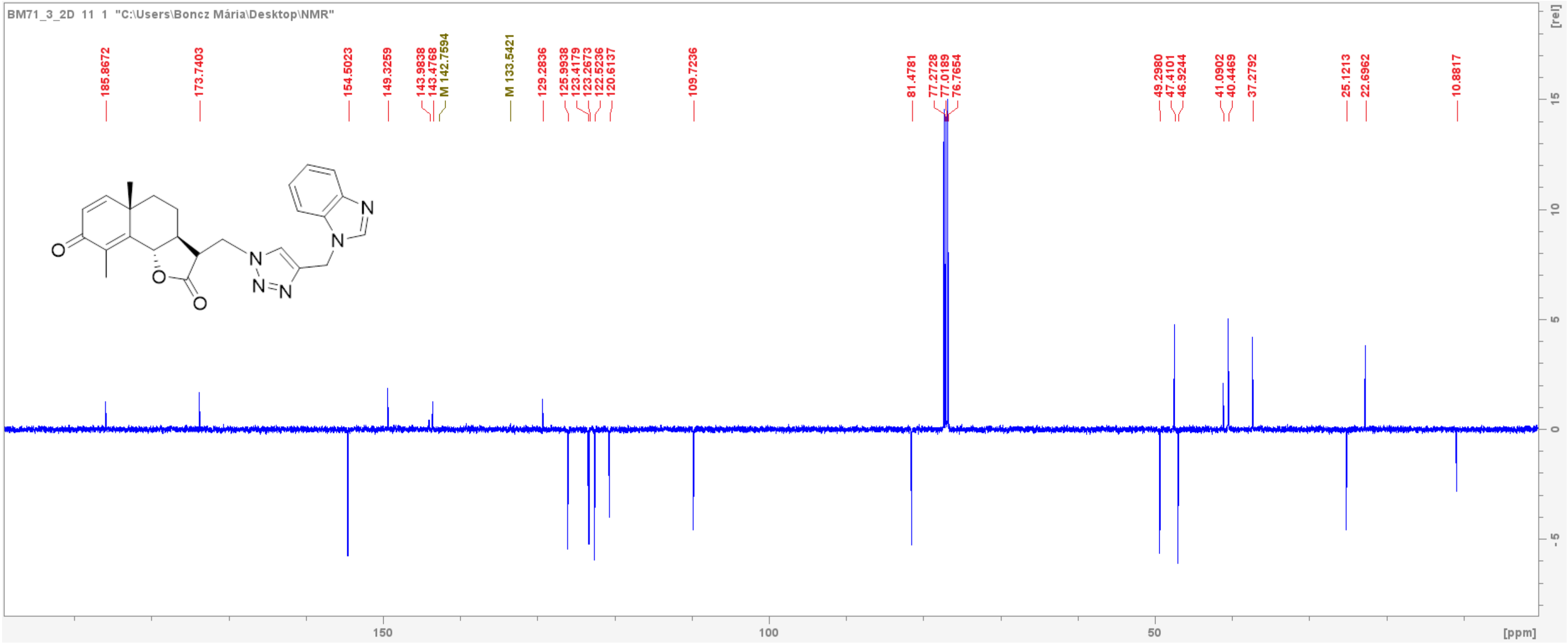

Figure S53 HSQC of compound **46**

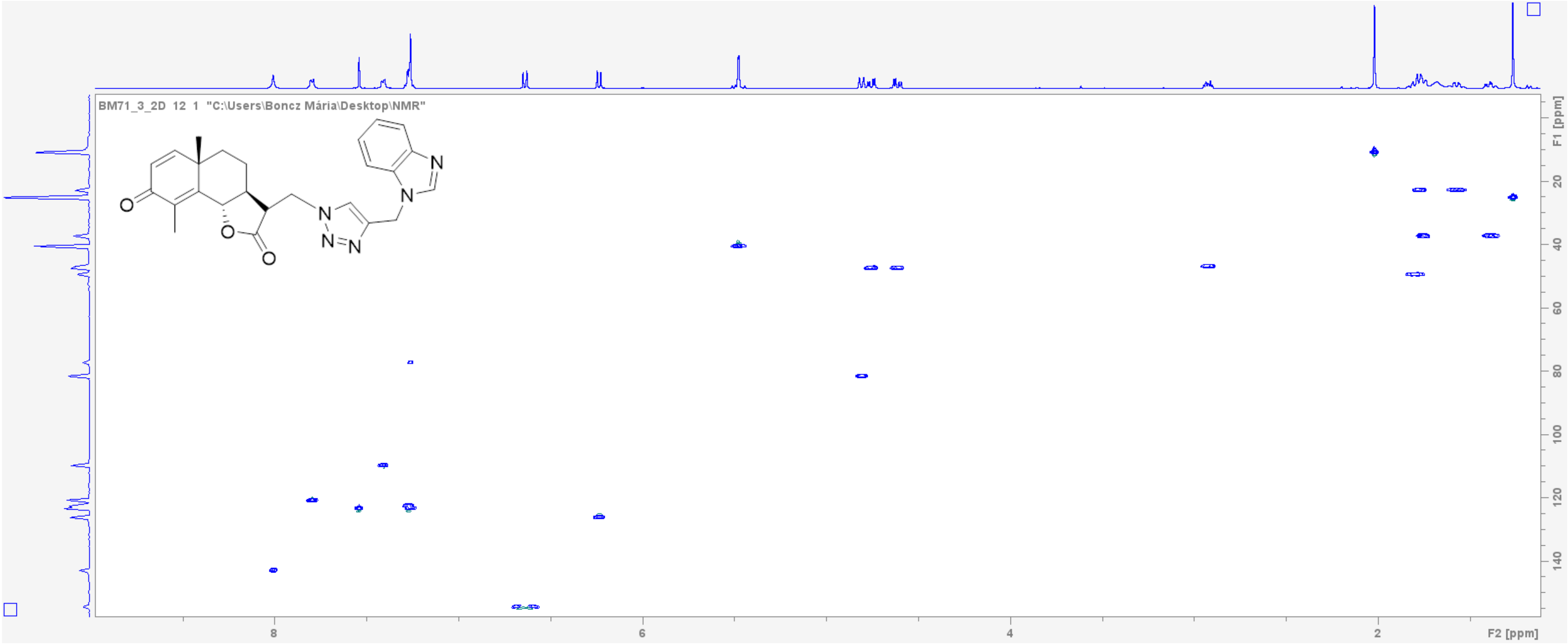

Figure S54 HMBC of compound **46**

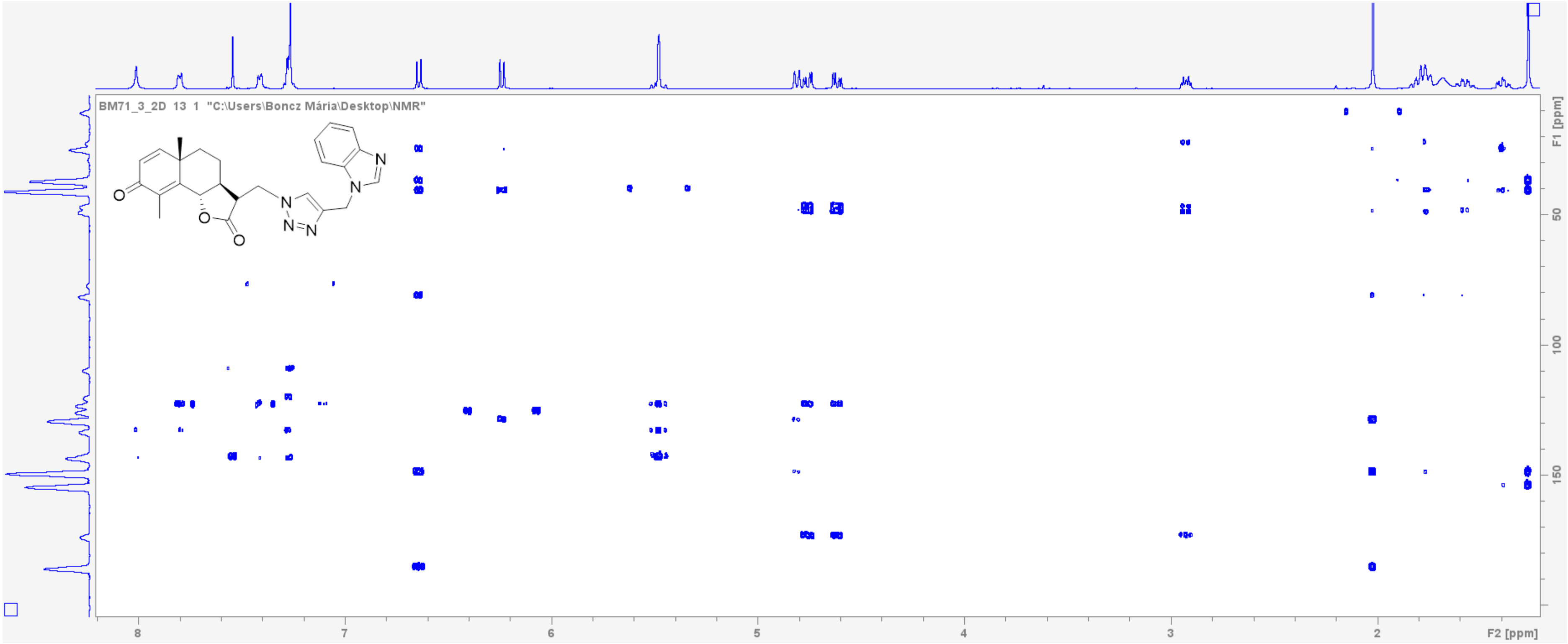

(5a*S*,9b*S*)-3-((4-((1*H*-indazol-1-yl)methyl)-1*H*-1,2,3-triazol-1-yl)methyl)-5a,9-dimethyl-3a,5,5a,9b-tetrahydronaphtho[1,2-*b*]furan-2,8(3*H*,4*H*)-dione (**47**)

Figure S55 ^{1}H-NMR of compound **47**

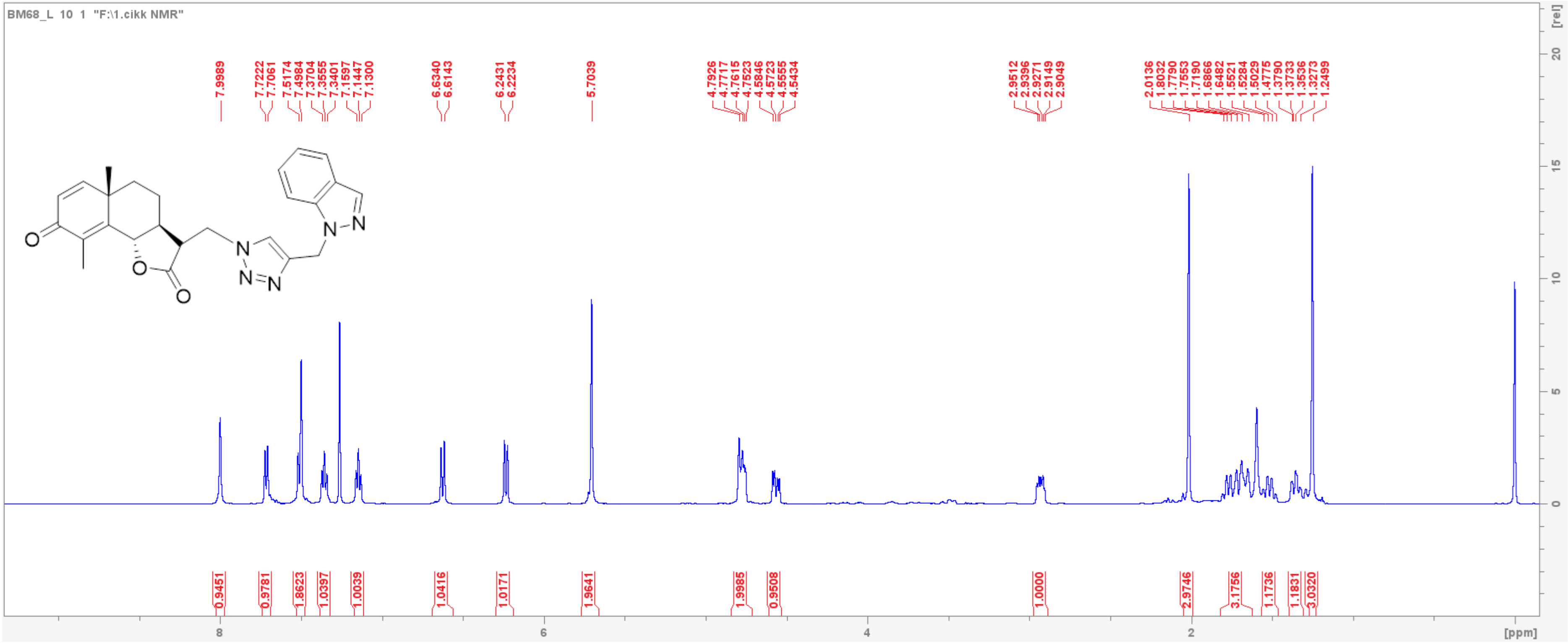

Figure S56 ^{13}C-NMR of compound **47**

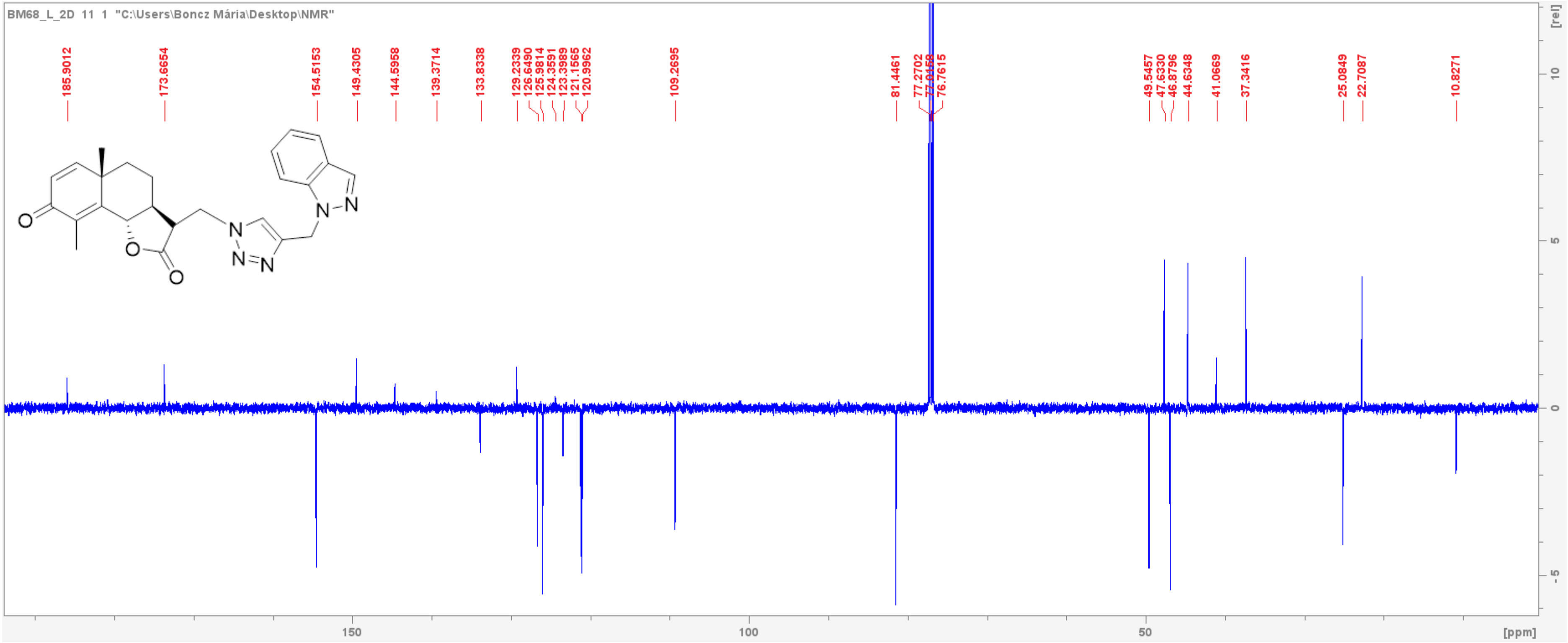

(5a*S*,9b*S*)-3-((4-((1*H*-benzo[*d*][1,2,3]triazol-1-yl)methyl)-1*H*-1,2,3-triazol-1-yl)methyl)-5a,9-dimethyl-3a,5,5a,9b-tetrahydronaphtho[1,2-*b*]furan-2,8(3*H*,4*H*)-dione (**48**)

Figure S57 ^{1}H-NMR of compound **48**

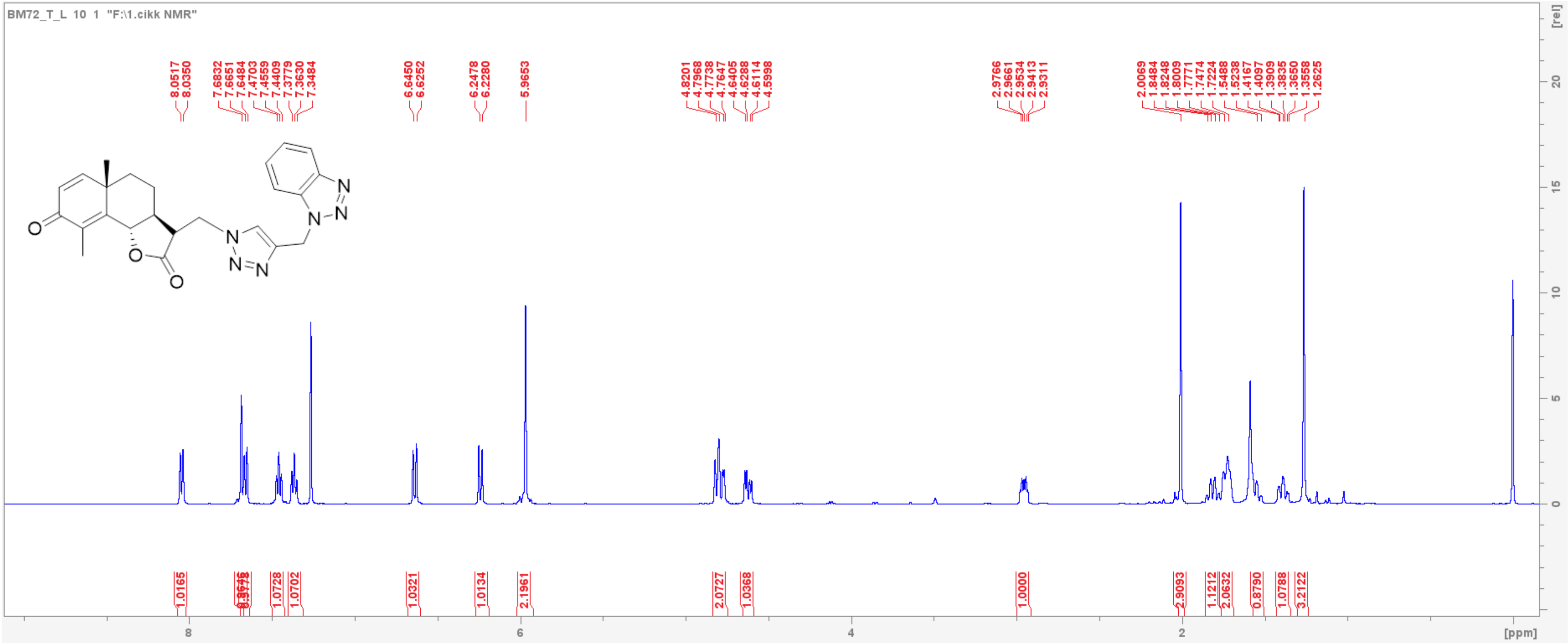

Figure S58 ^{13}C-NMR of compound **48**

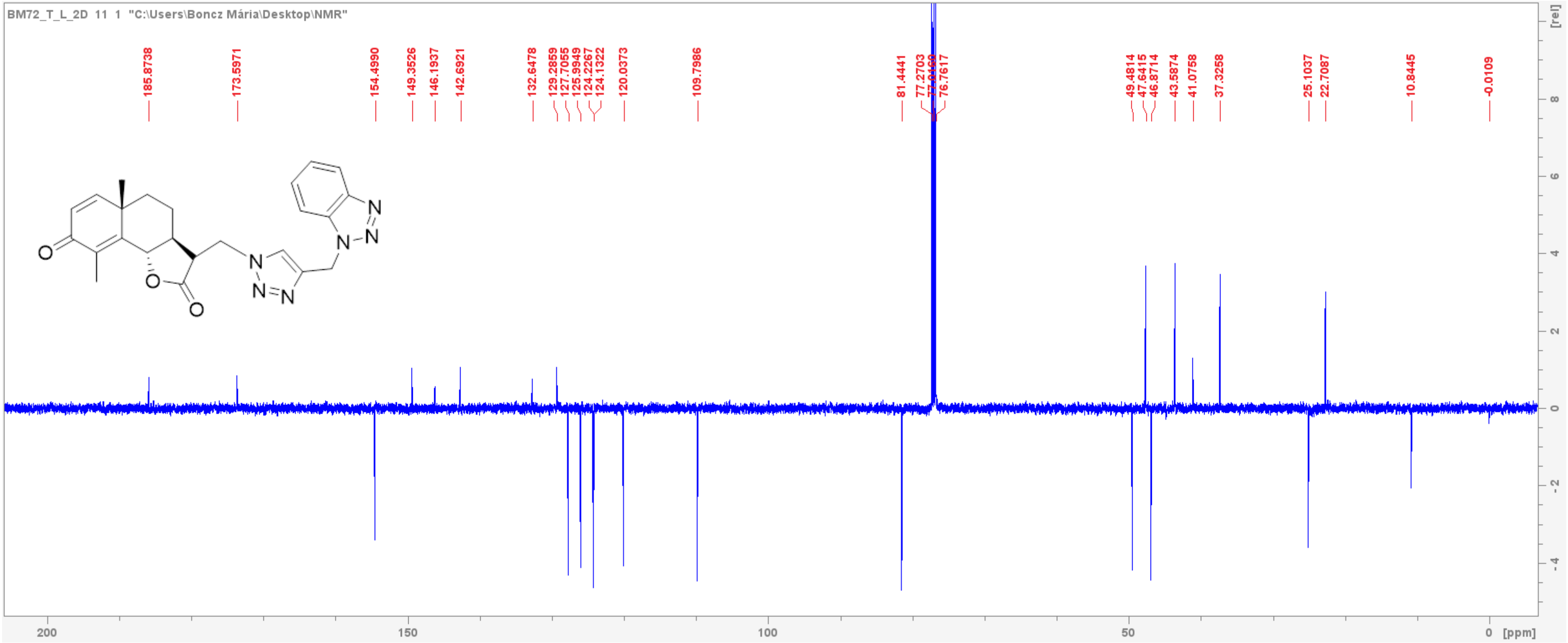

(5a*S*,9b*S*)-5a,9-dimethyl-3-((4-((pyrimidin-2-ylthio)methyl)-1*H*-1,2,3-triazol-1-yl)methyl)-3a,5,5a,9b-tetrahydronaphtho[1,2-*b*]furan-2,8(3*H*,4*H*)-dione (**49**)

Figure S59 ^{1}H-NMR of compound **49**

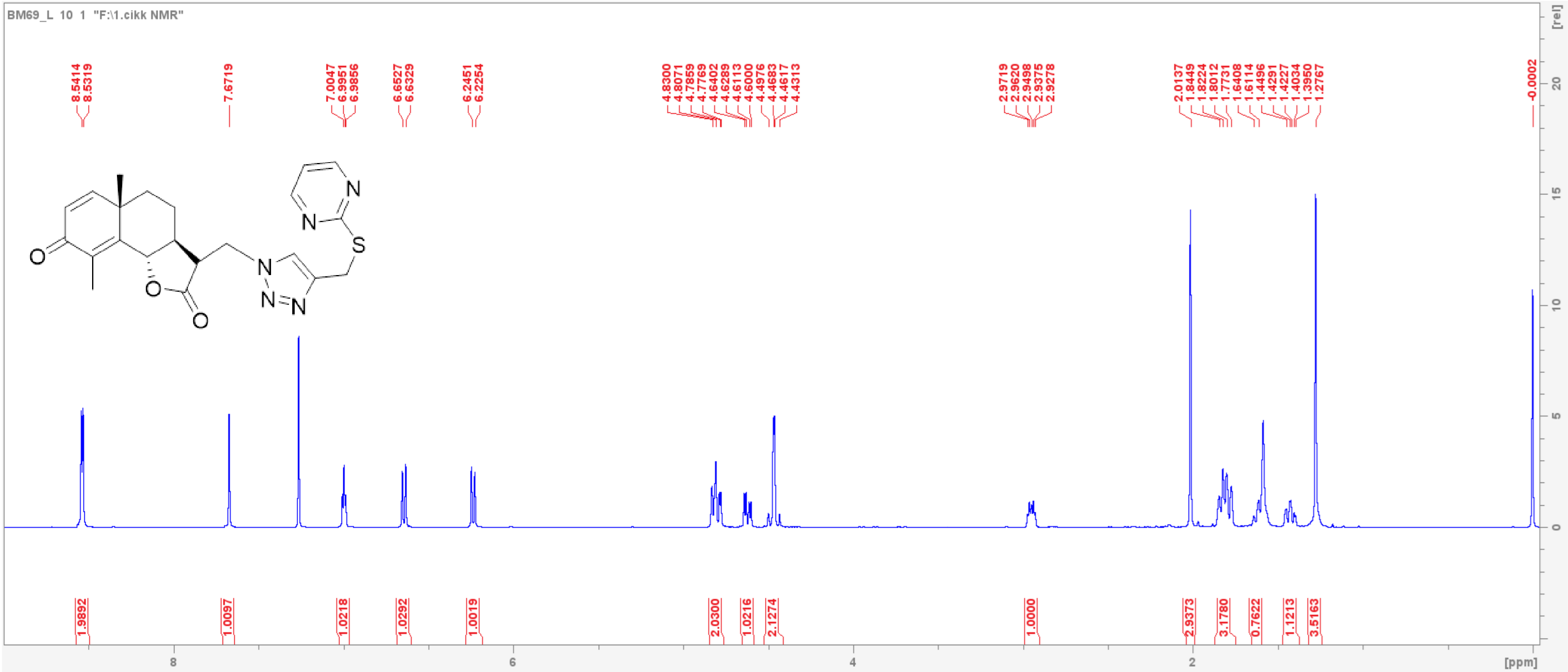

Figure S60 ^{13}C-NMR of compound **49**

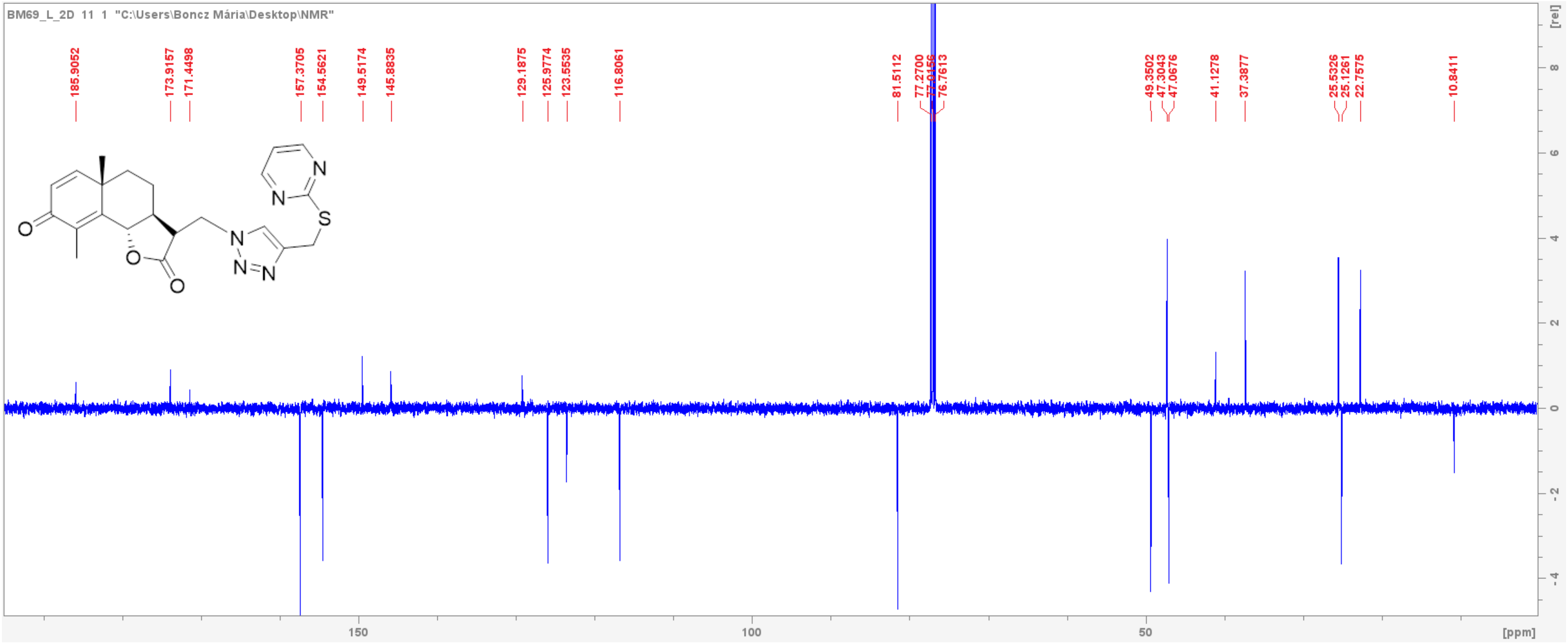

(5a*S*,9b*S*)-3-((4-((benzo[d]oxazol-2-ylthio)methyl)-1H-1,2,3-triazol-1-yl)methyl)-5a,9-dimethyl-3a,5,5a,9b-tetrahydronaphtho[1,2-*b*]furan-2,8(3*H*,4*H*)-dione (**50**)

Figure S61 ^{1}H-NMR of compound **50**

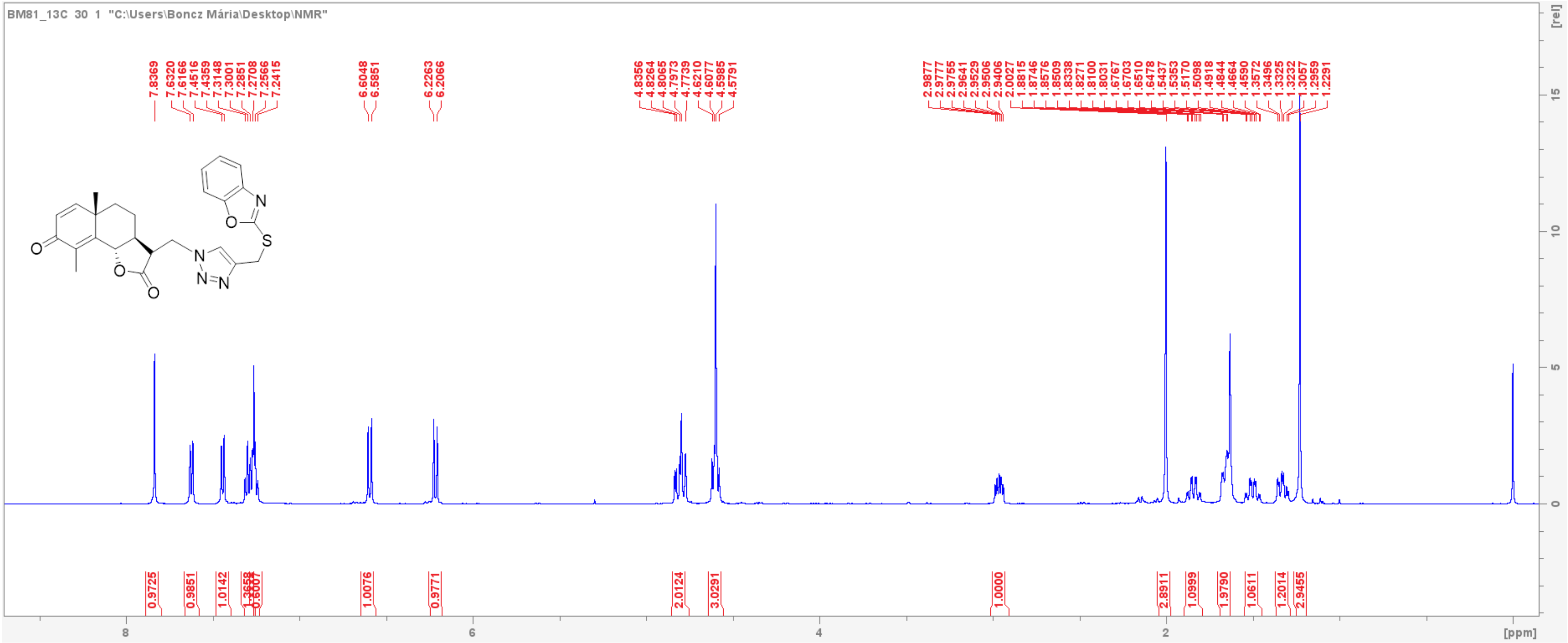

Figure S62 ^{13}C-NMR of compound **50**

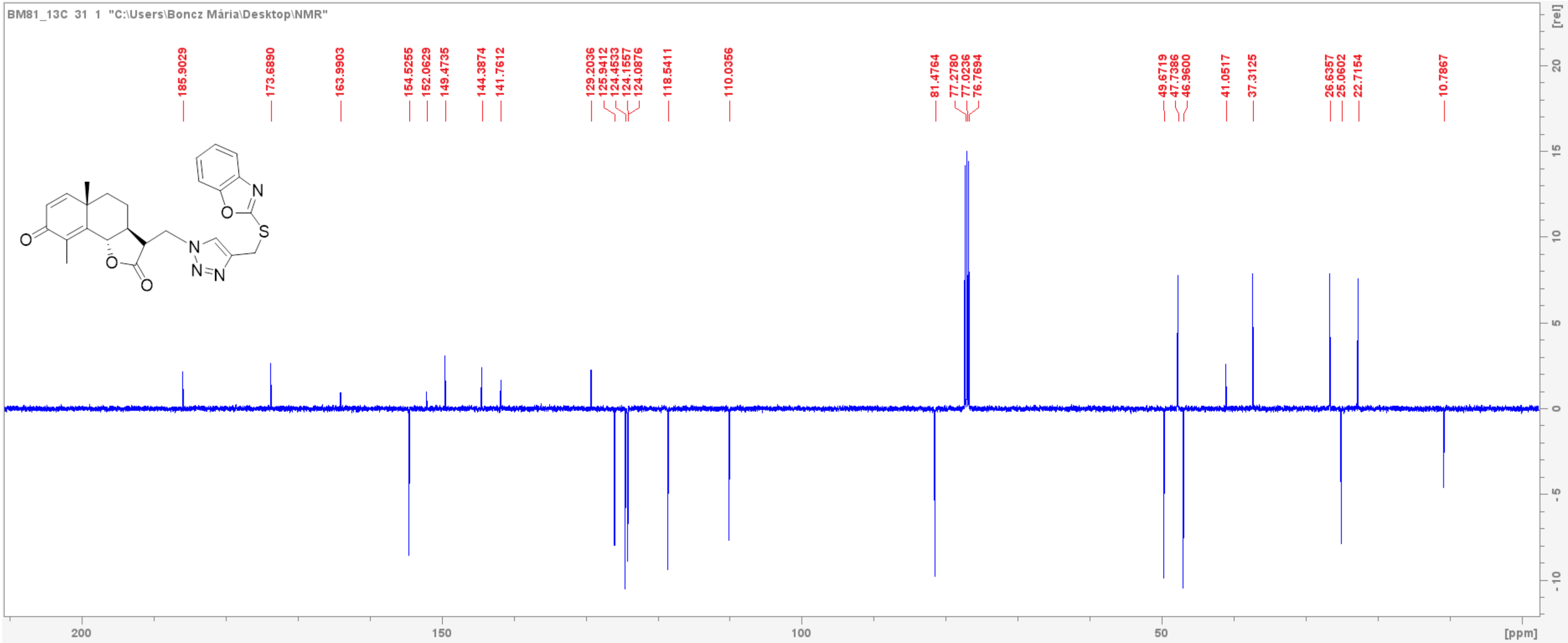

Figure S63 HSQC of compound **50**

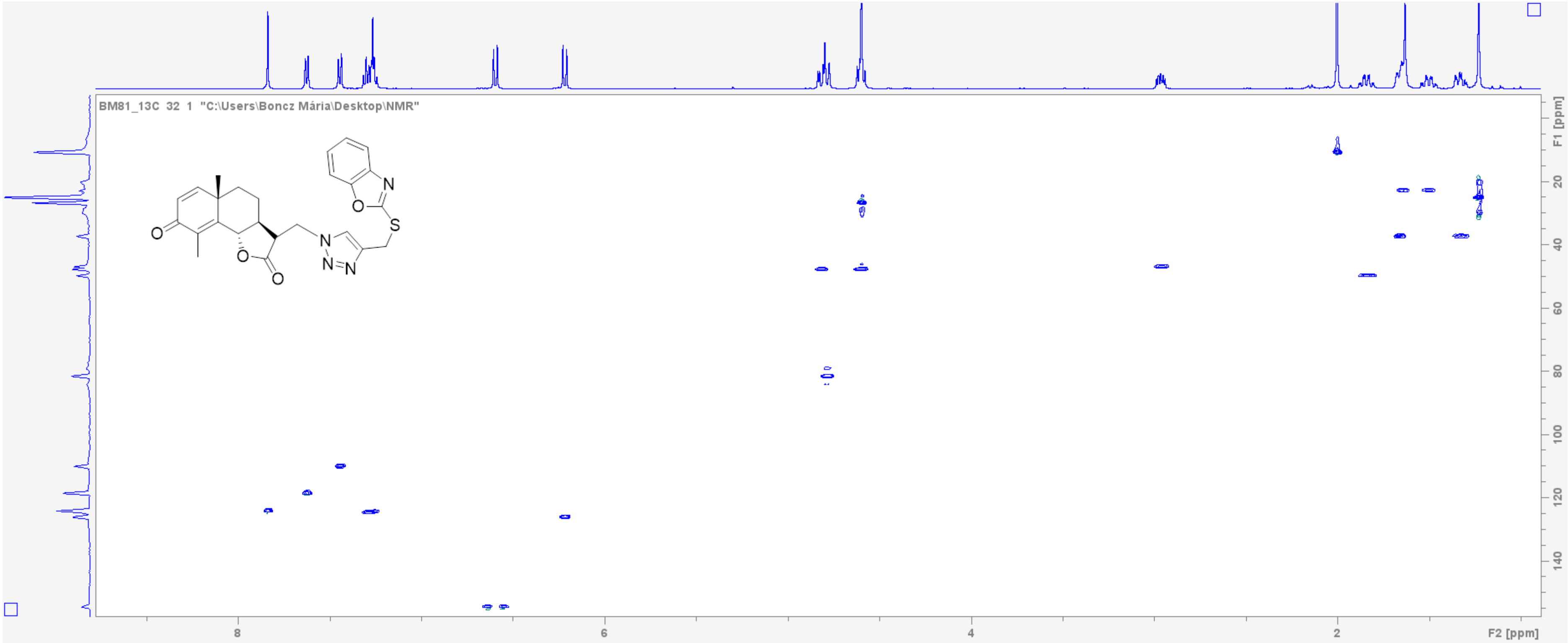

Figure S64 HMBC of compound **50**

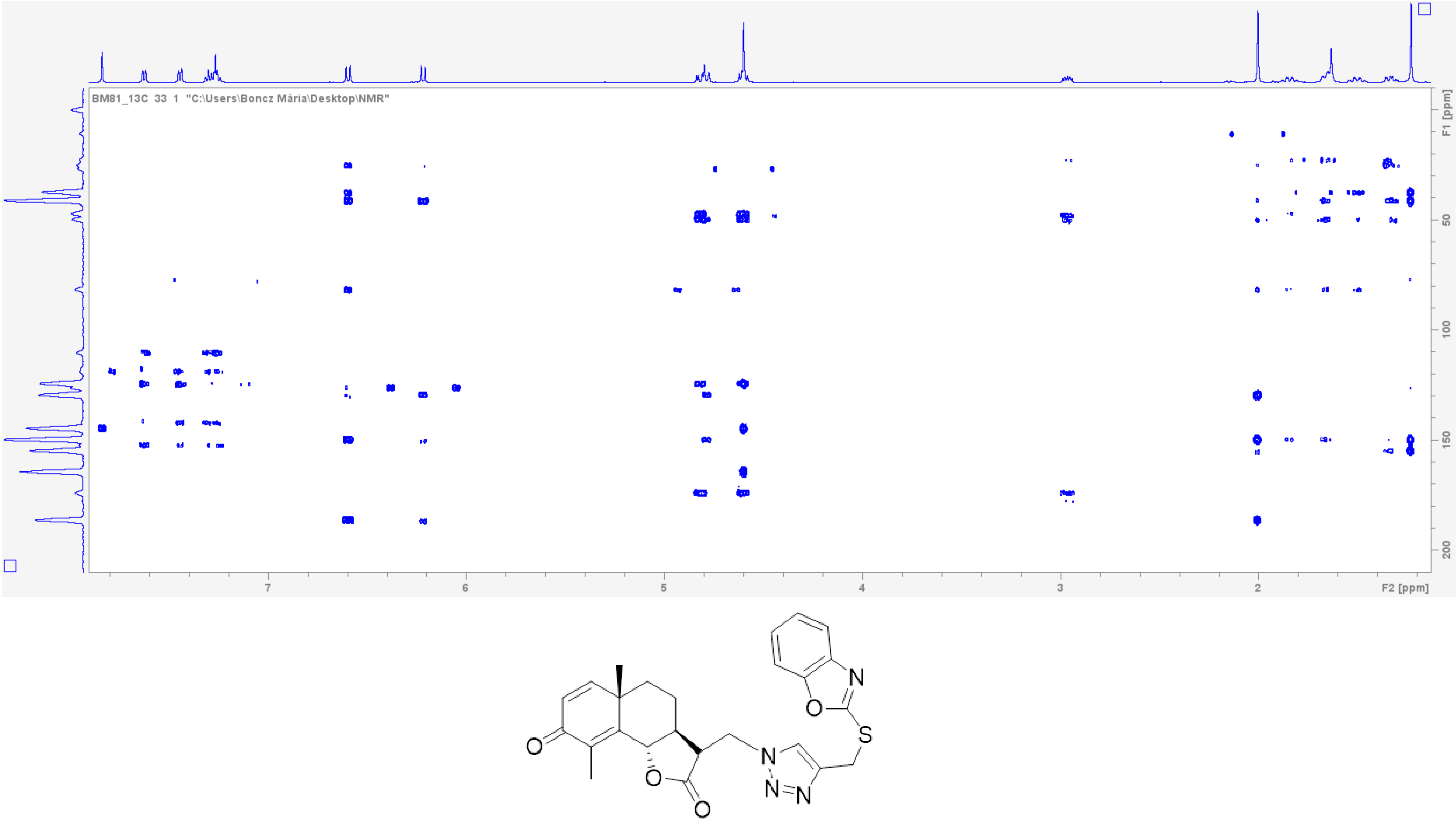

(5a*S*,9b*S*)-3-((4-((benzo[*d*]thiazol-2-ylthio)methyl)-1*H*-1,2,3-triazol-1-yl)methyl)-5a,9-dimethyl-3a,5,5a,9b-tetrahydronaphtho[1,2-*b*]furan-2,8(3*H*,4*H*)-dione, (**51**)

Figure S65 ¹H of compound **51**

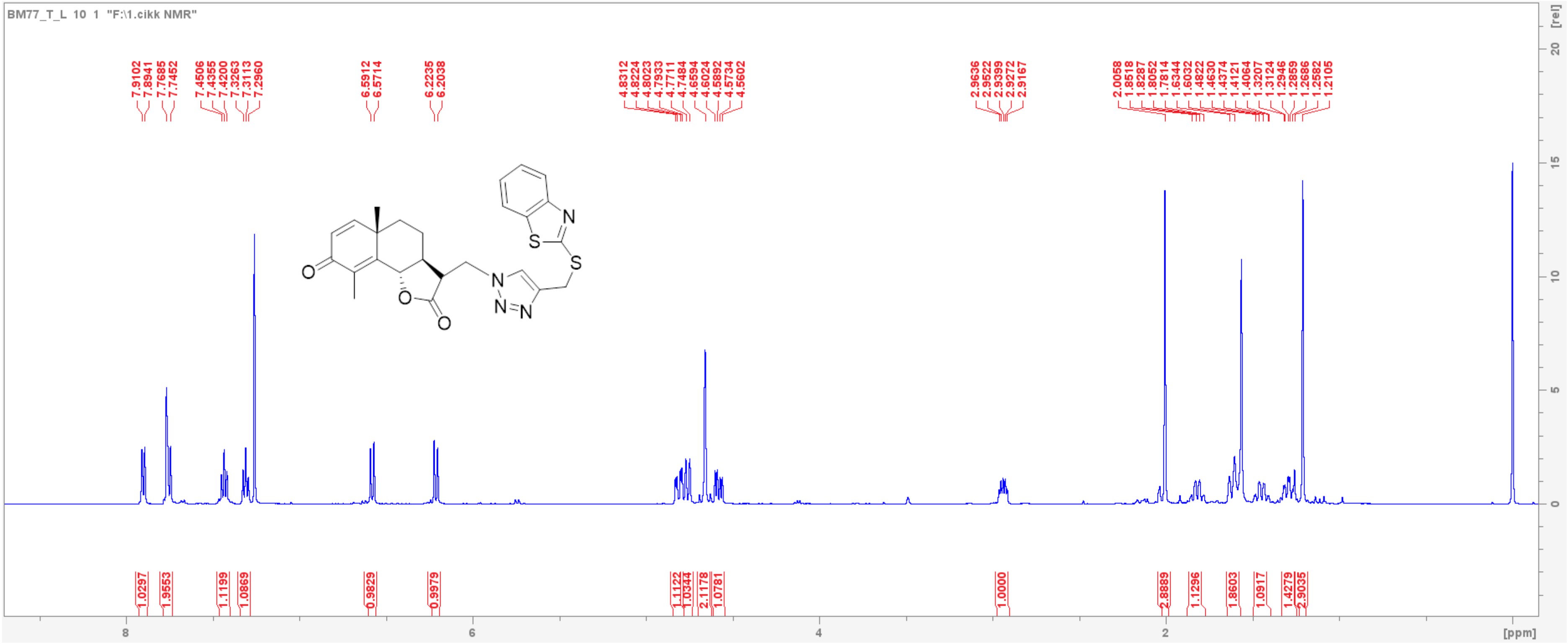

Figure S66 ^{13}C of compound **51**

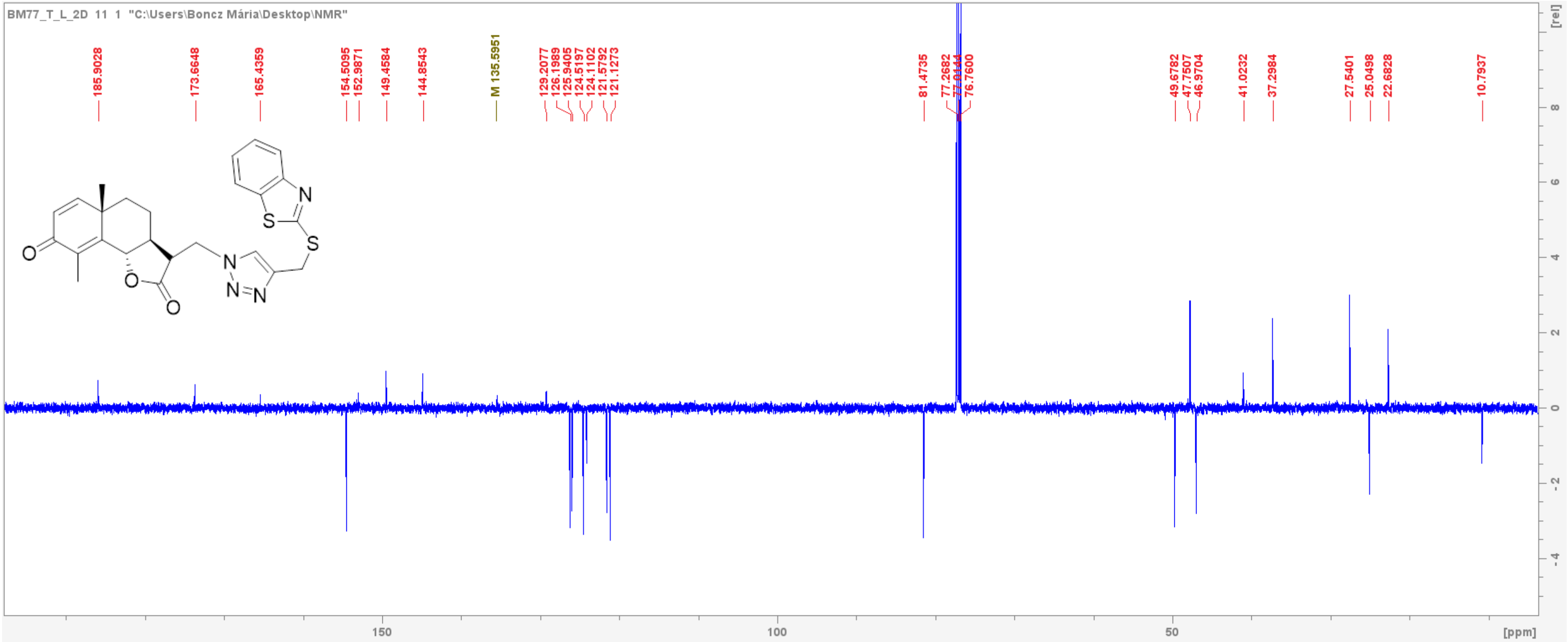

Figure S67 HSQC of compound **51**

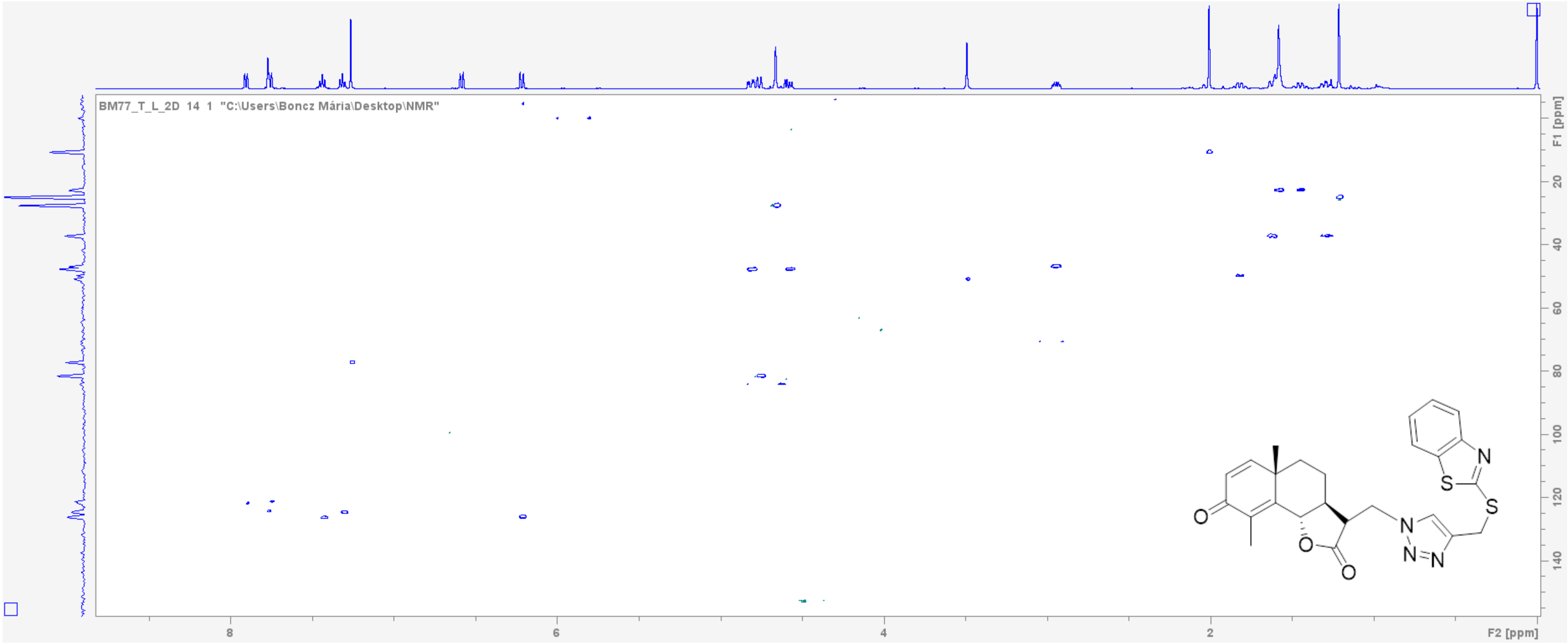

Figure S68 HMBC of compound **51**

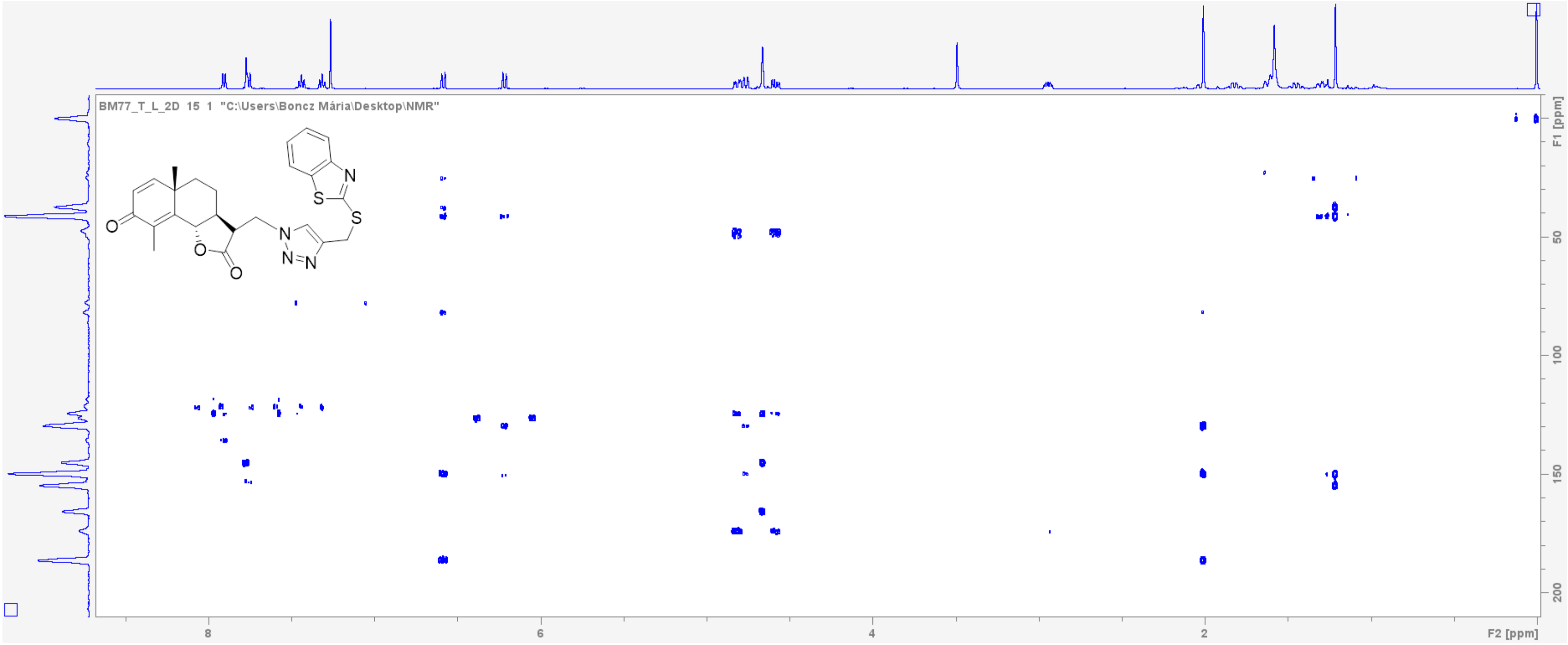

(5a*S*,9b*S*)-5a,9-dimethyl-3-((4-((quinolin-8-yloxy)methyl)-1*H*-1,2,3-triazol-1-yl)methyl)-3a,5,5a,9b-tetrahydronaphtho[1,2-*b*]furan-2,8(3*H*,4*H*)-dione (**52**)

Figure S69 ^{1}H of compound **52**

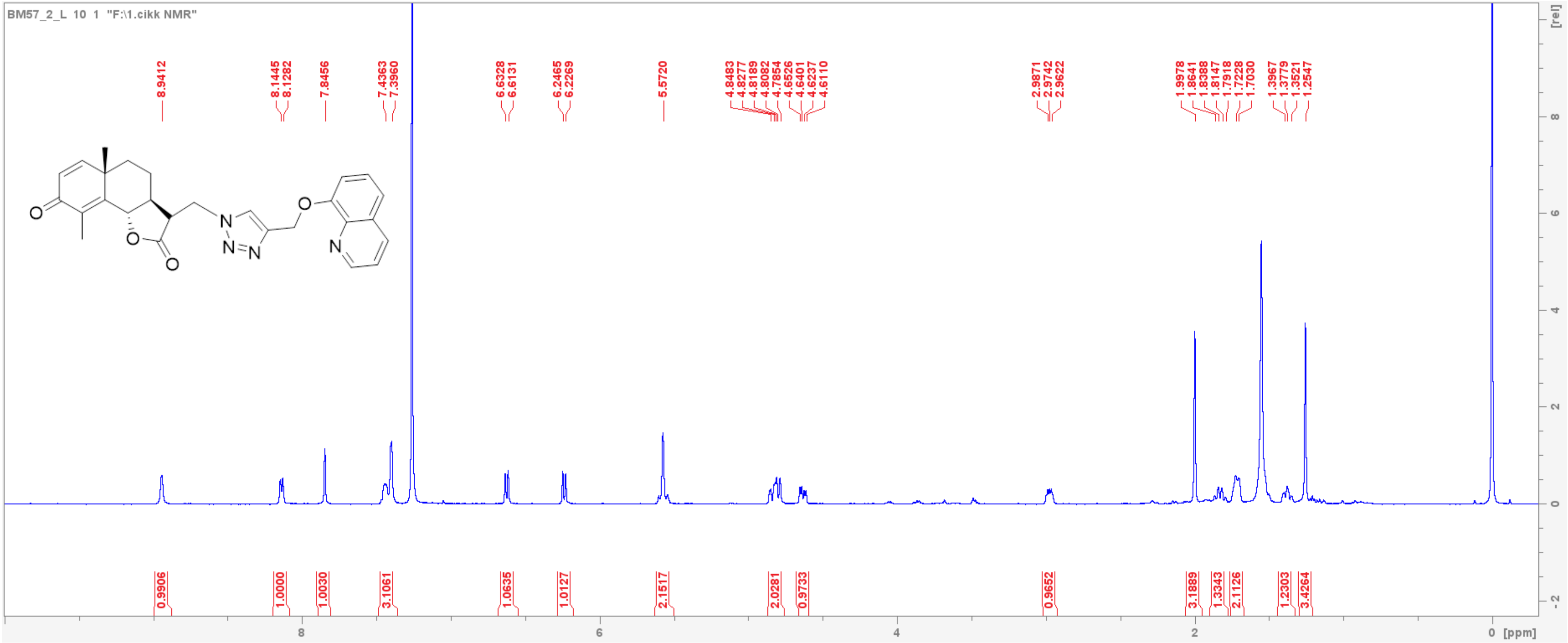

Figure S70 ^{13}C of compound **52**

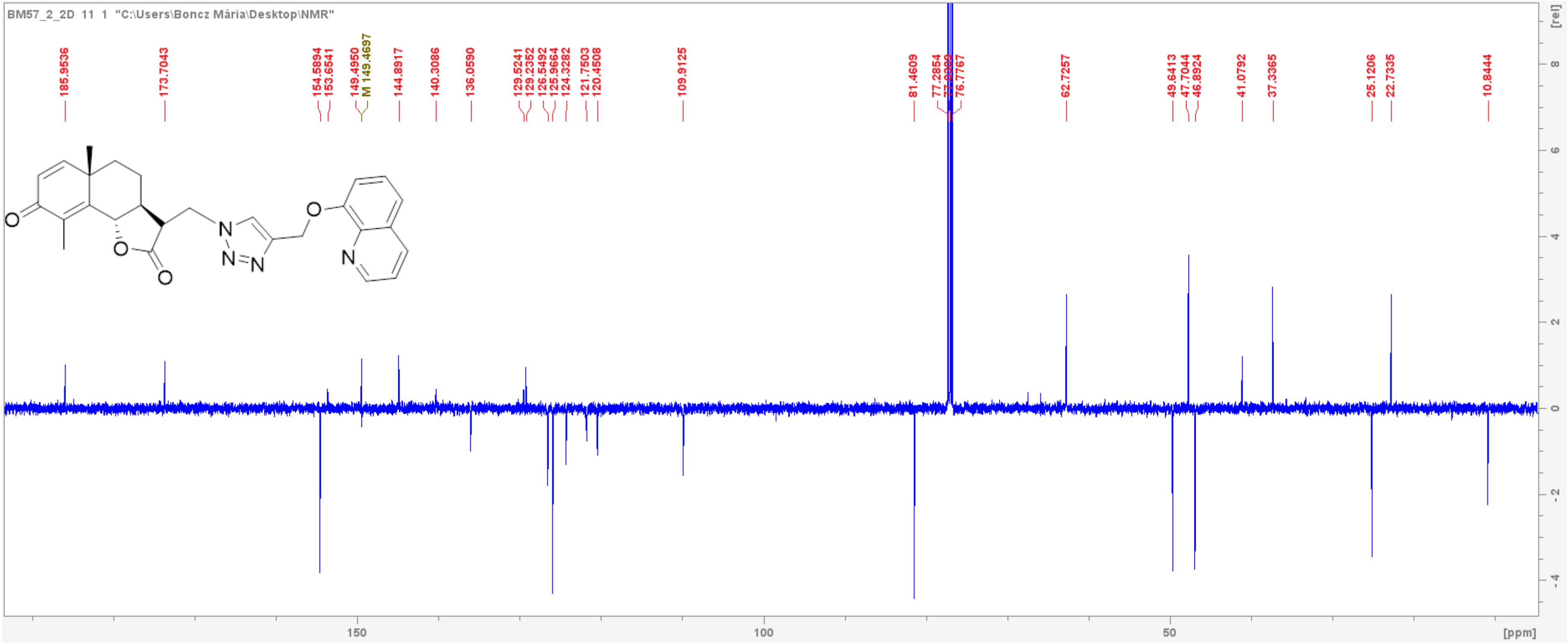

Figure S71 HSQC of compound **52**

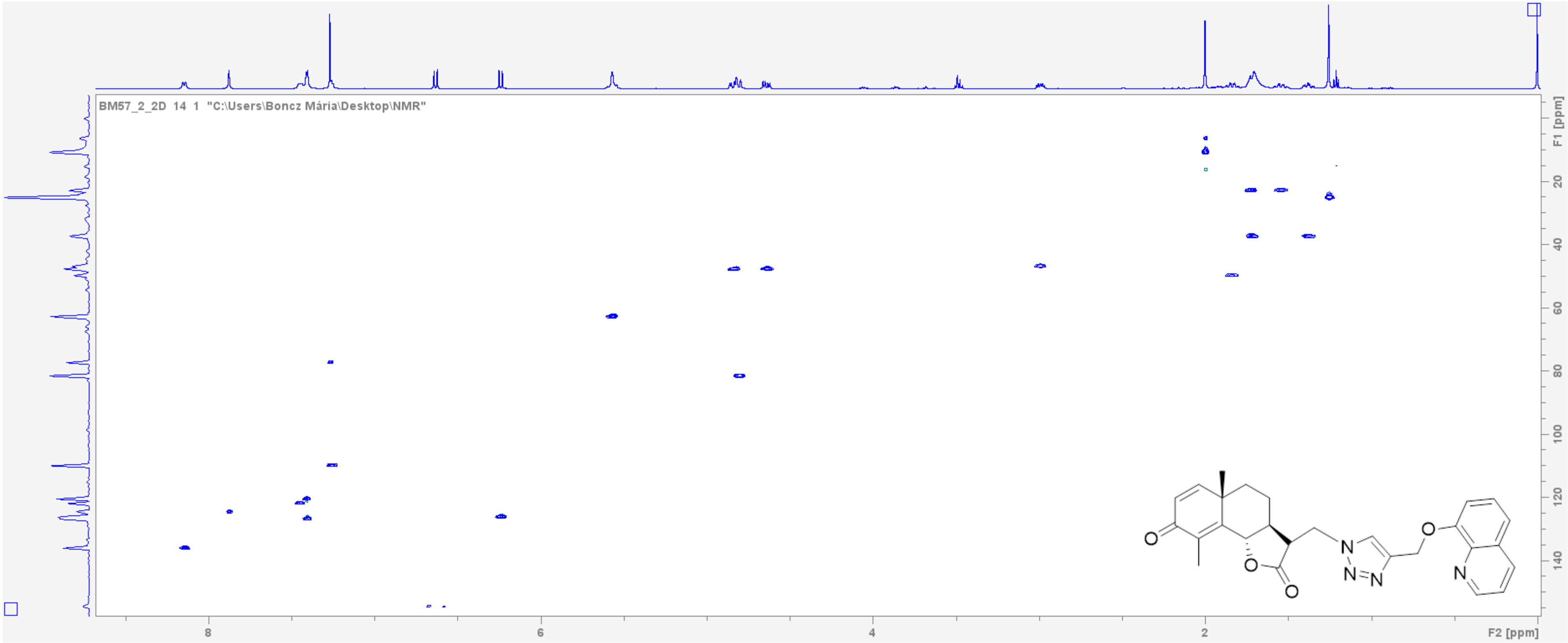

Figure S72 HMBC of compound **52**

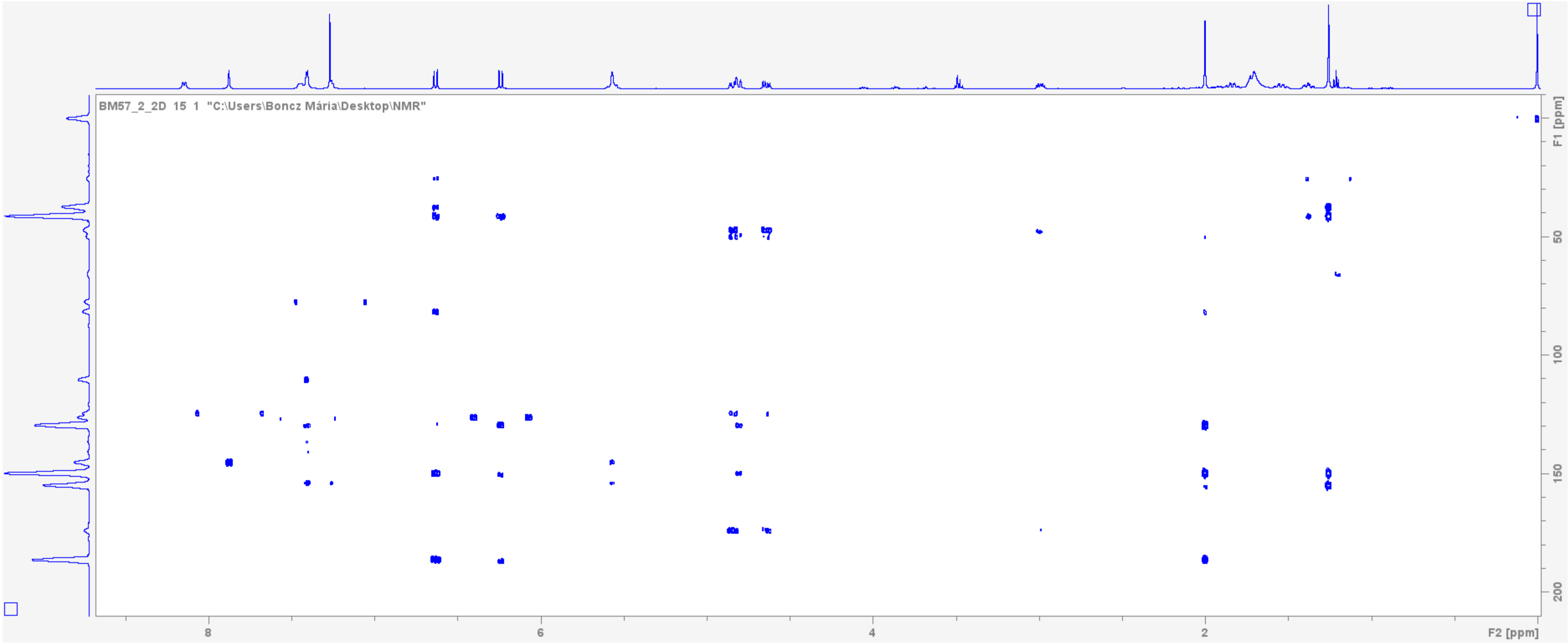

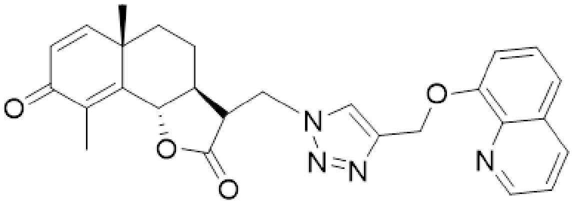

(5a*S*,9b*S*)-5a,9-dimethyl-3-((4-(((2-oxo-2*H*-chromen-6-yl)oxy)methyl)-1*H*-1,2,3-triazol-1-yl)methyl)-3a,5,5a,9b-tetrahydronaphtho[1,2-*b*]furan-2,8(3*H*,4*H*)-dione (**53**)

Figure S73 ^{1}H of compound **53**

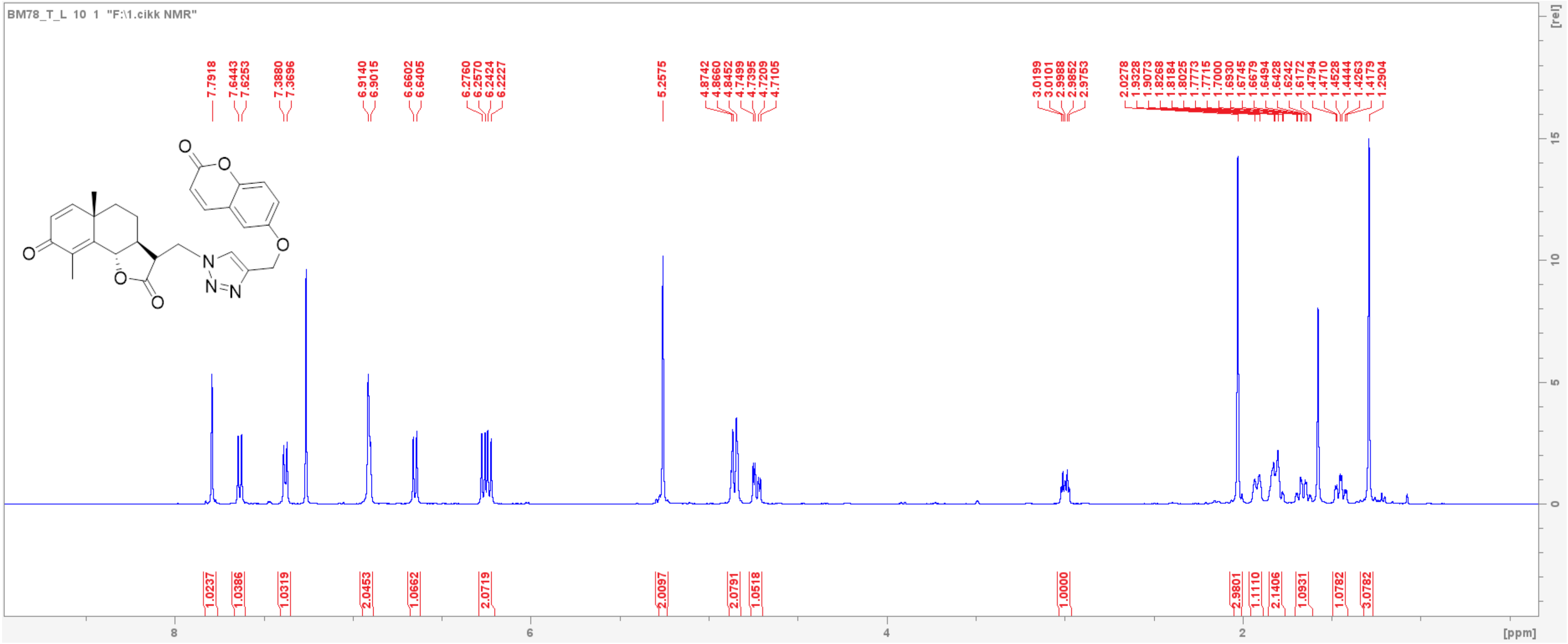

Figure S74 ^{13}C-NMR of compound **53**

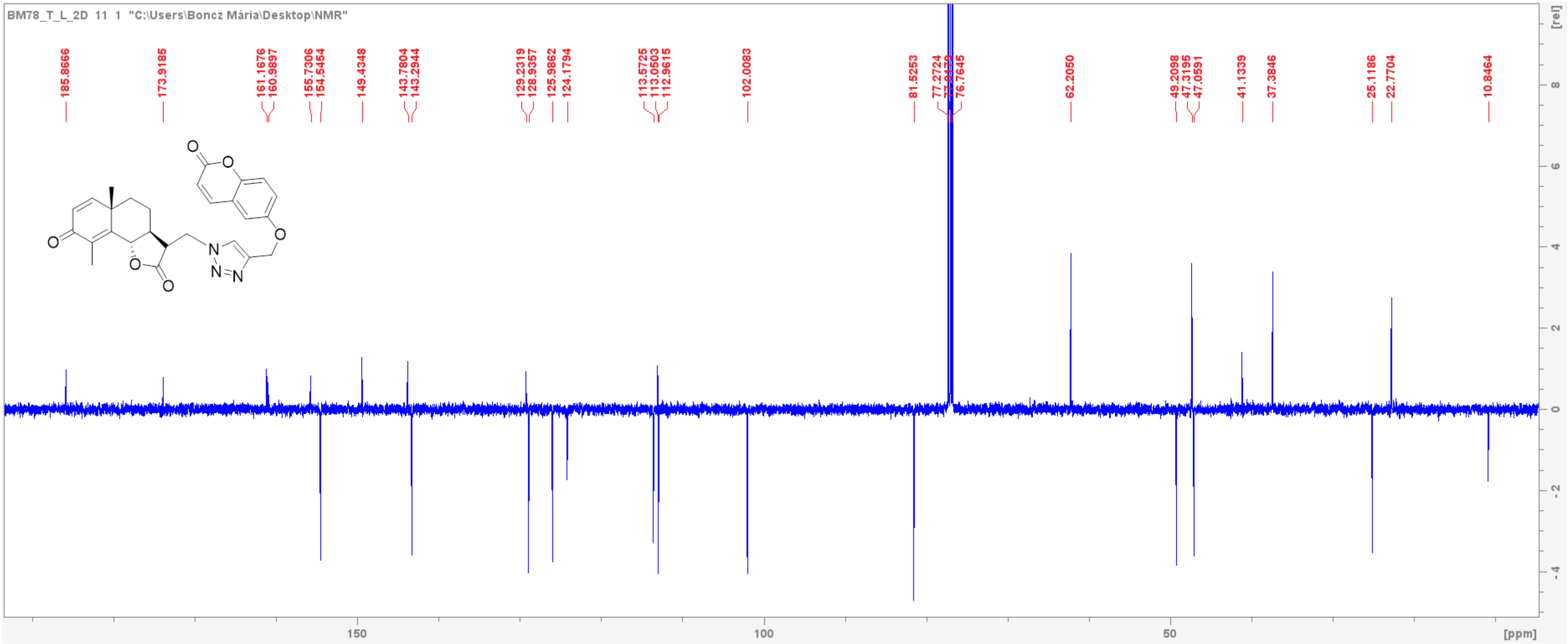

(5aS,9bS)-5a,9-dimethyl-3-((4-(((2,5-dichloropyrimidin-4-yl)amino)methyl)-1H-1,2,3-triazol-1-yl)methyl)-3a,5,5a,9b-tetrahydronaphtho[1,2-b]furan-2,8(3H,4H)-dione (**54**)

Figure S75 ^{1}H of compound **54**

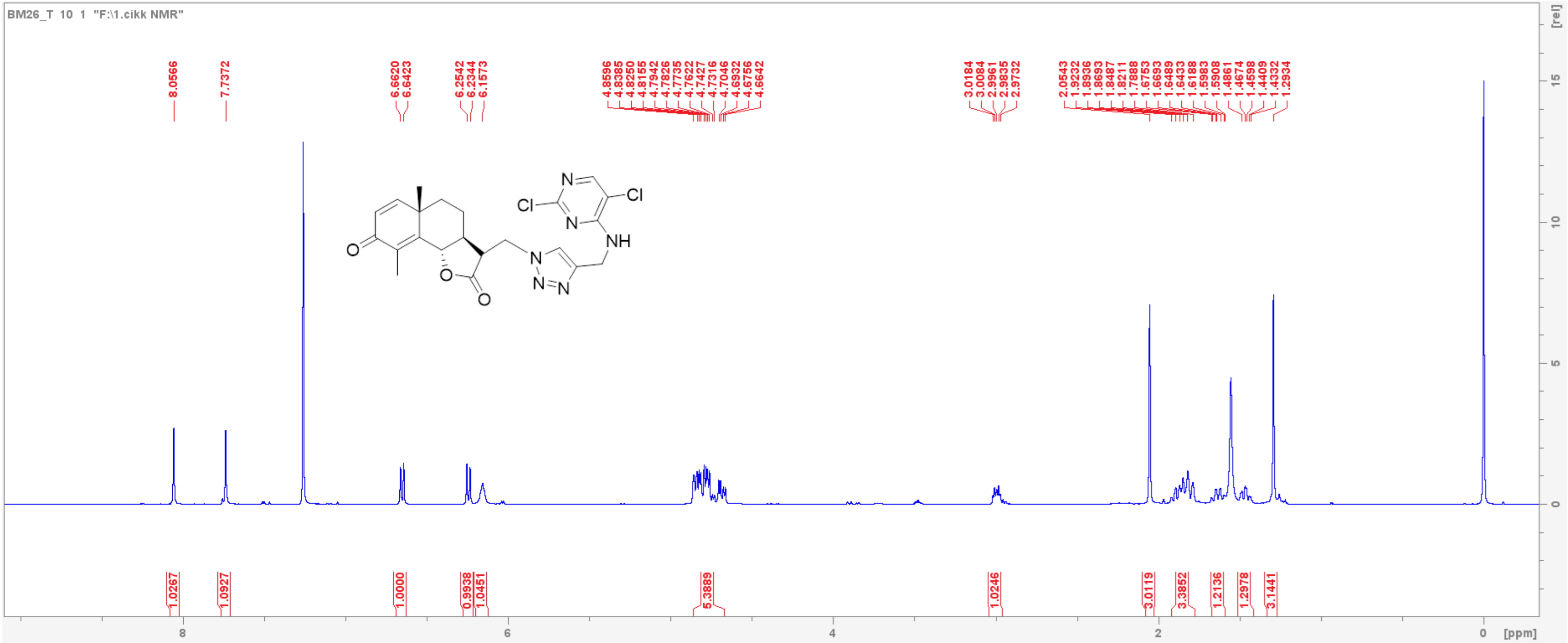

Figure S76 ^{13}C-NMR of compound **54**

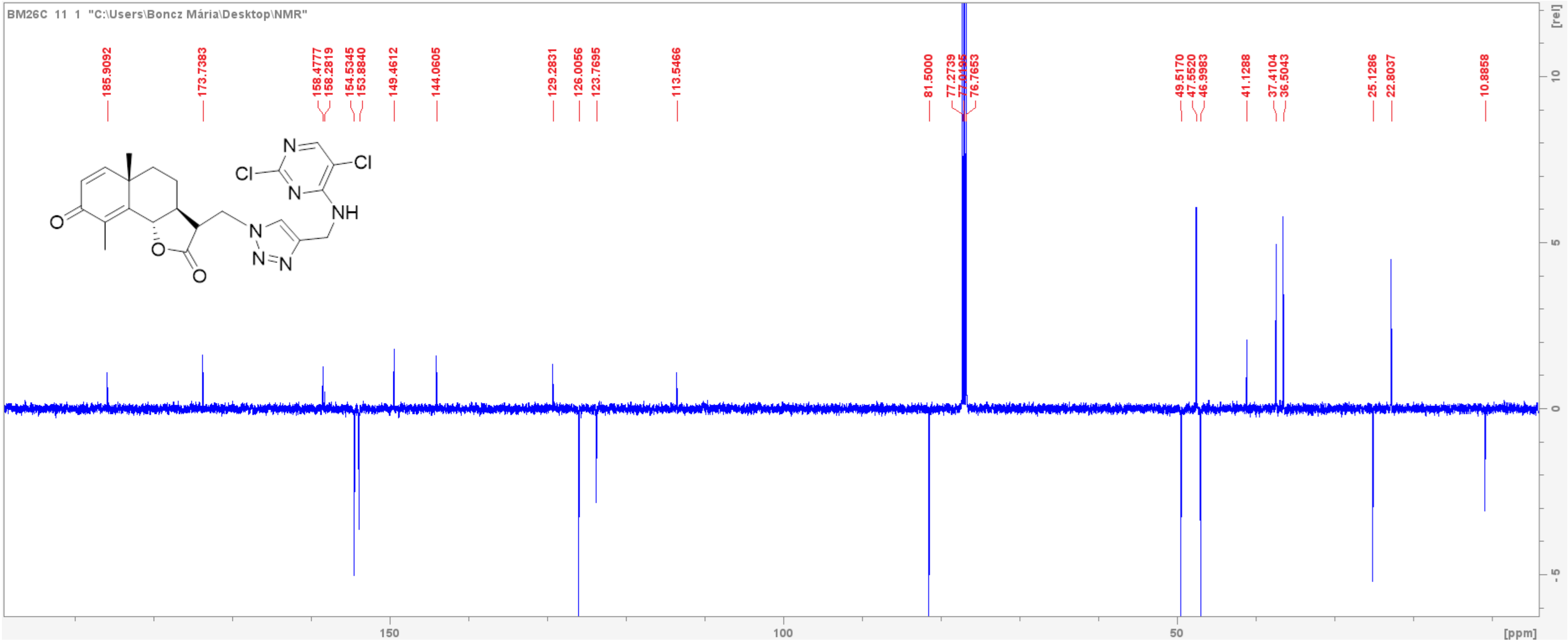

(5a*S*,9b*S*)-5a,9-dimethyl-3-((4-(((2-chloro-5-fluoropyrimidin-4-yl)amino)methyl)-1*H*-1,2,3-triazol-1-yl)methyl)-3a,5,5a,9b-tetrahydronaphtho[1,2-*b*]furan-2,8(3*H*,4*H*)-dione (**55**)

Figure S77 ^{1}H of compound **55**

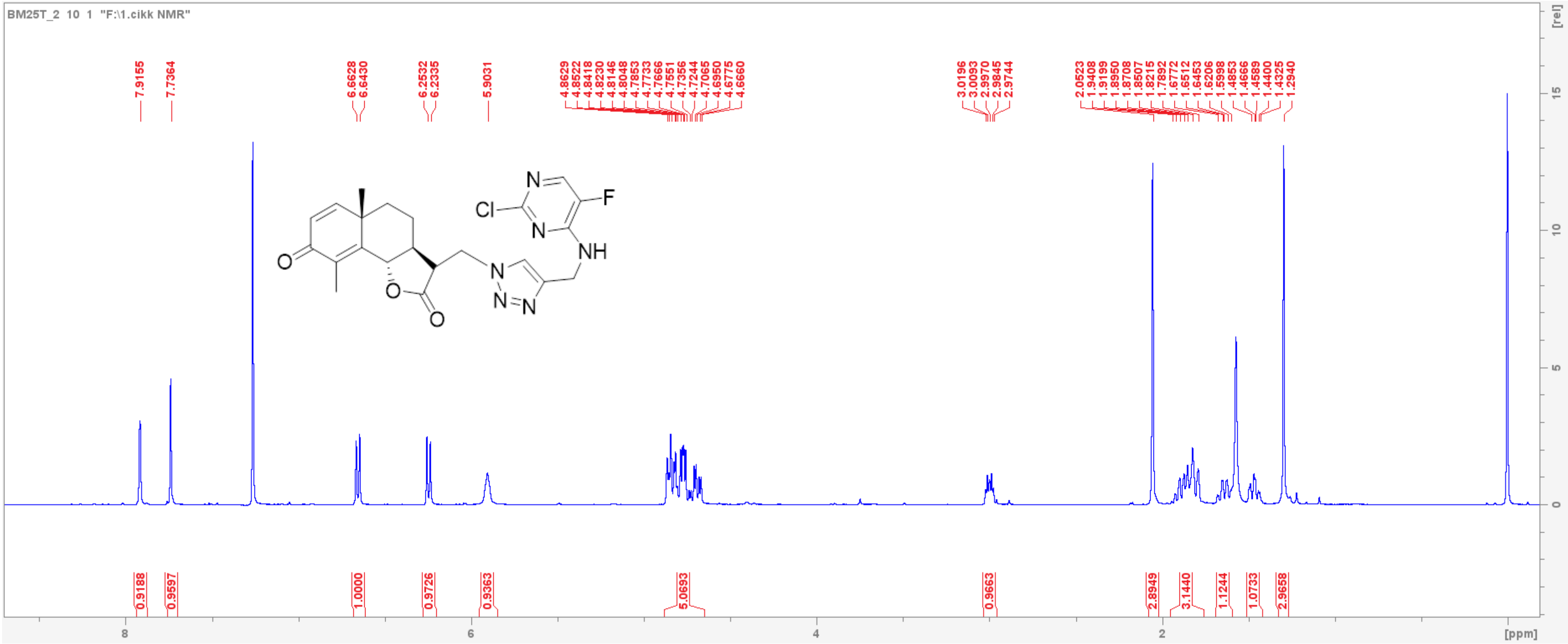

Figure S78 ^{13}C-NMR of compound **55**

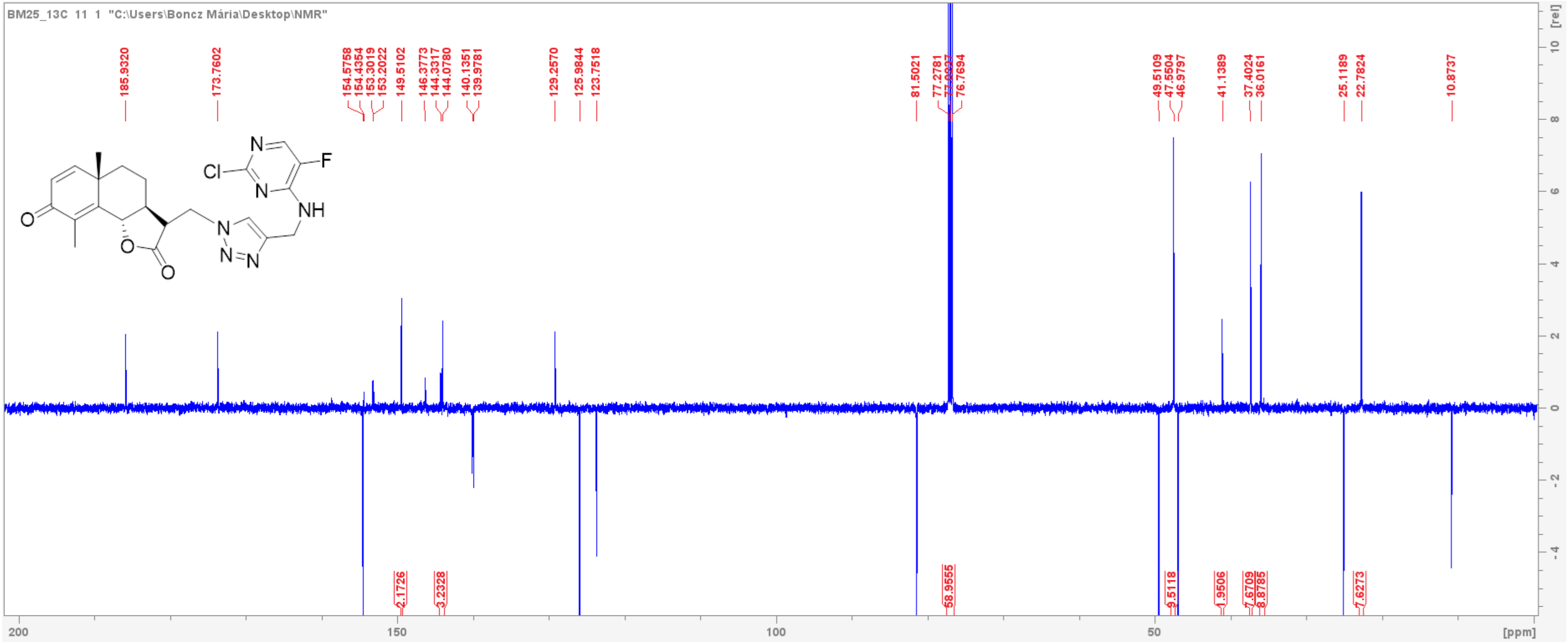

Figure S79 HMBC of compound **55**

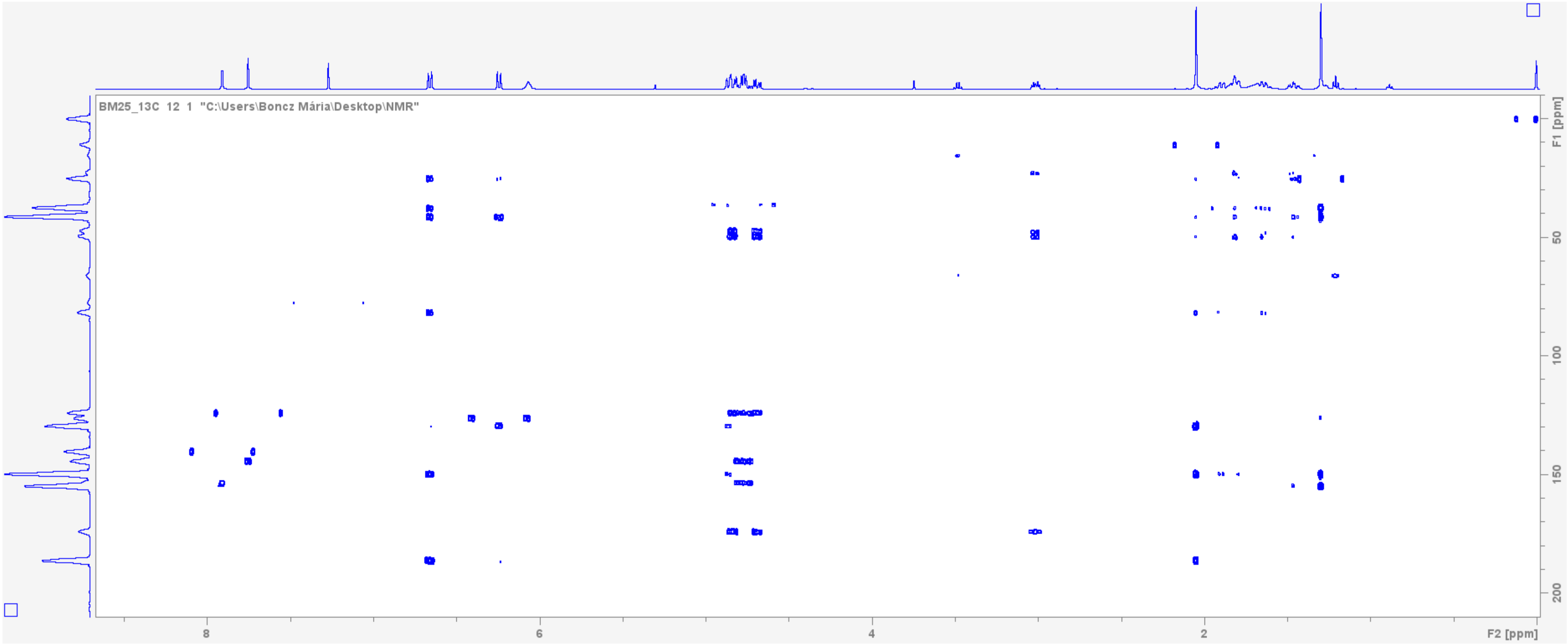

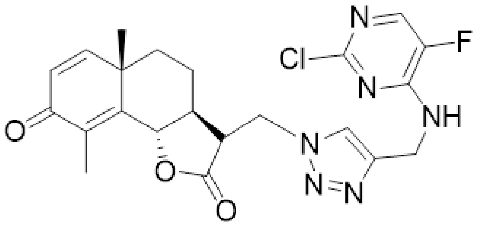

Figure S80 ^{19}F-NMR of compound **55**

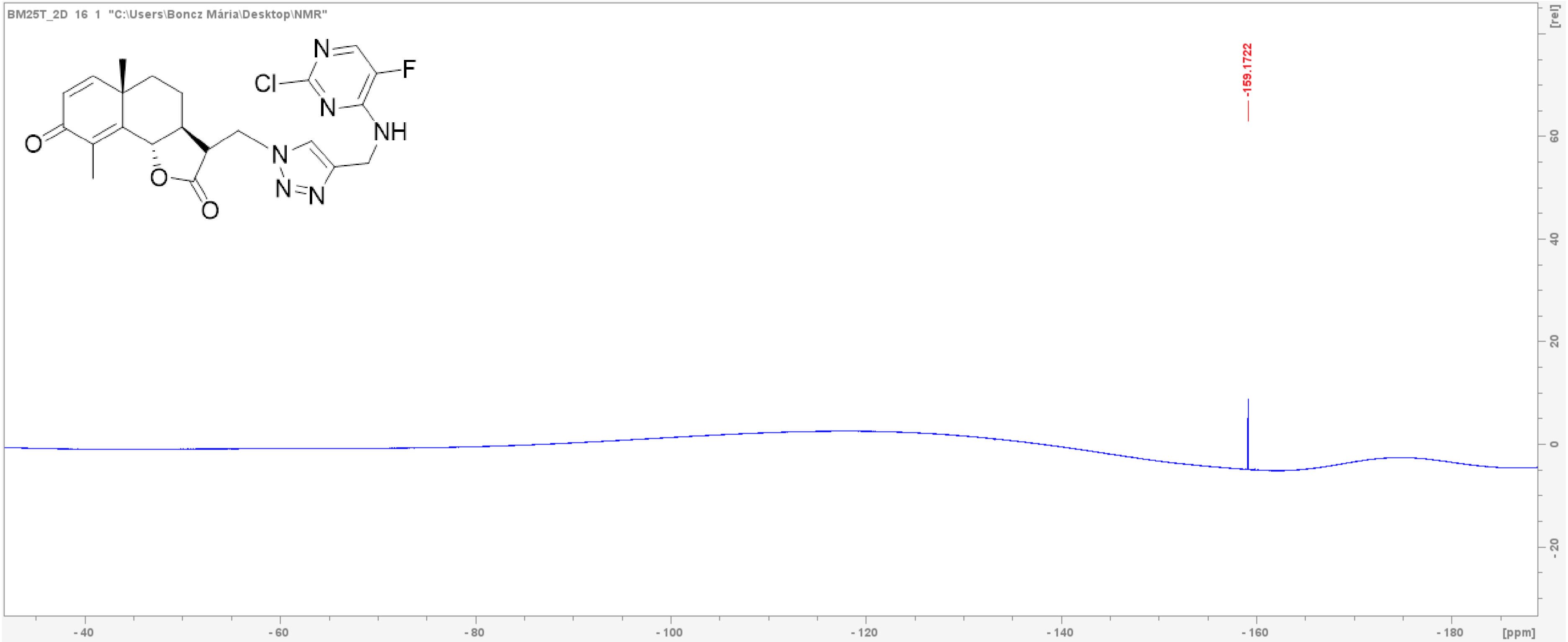

(5a*S*,9b*S*)-5a,9-dimethyl-3-((4-(((2-trifluoromethyl-5-chloropyrimidin-4-yl)amino)methyl)-1*H*-1,2,3-triazol-1-yl)methyl)-3a,5,5a,9b-tetrahydronaphtho[1,2-*b*]furan-2,8(3*H*,4*H*)-dione (**56**)

Figure S81 ^{1}H of compound **56**

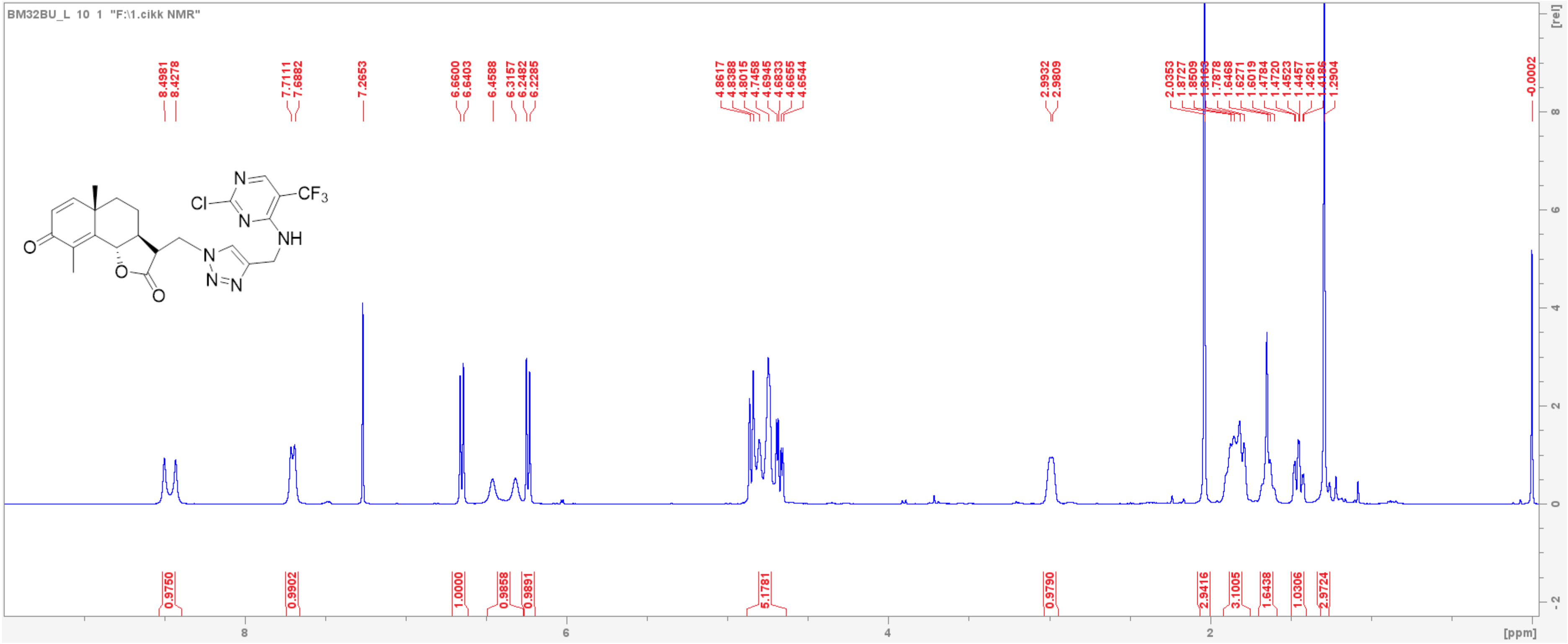

Figure S82 ^{13}C-NMR of compound **56**

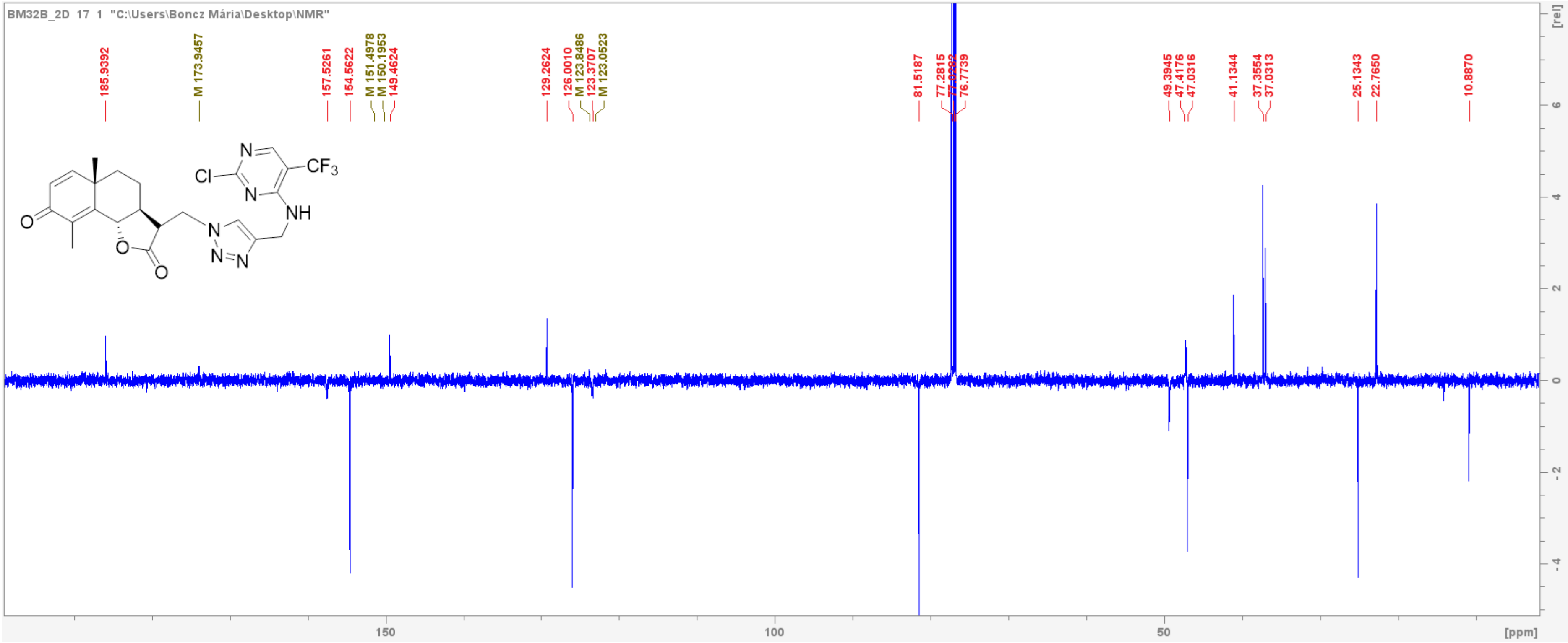

Figure S83 HSQC of compound **56**

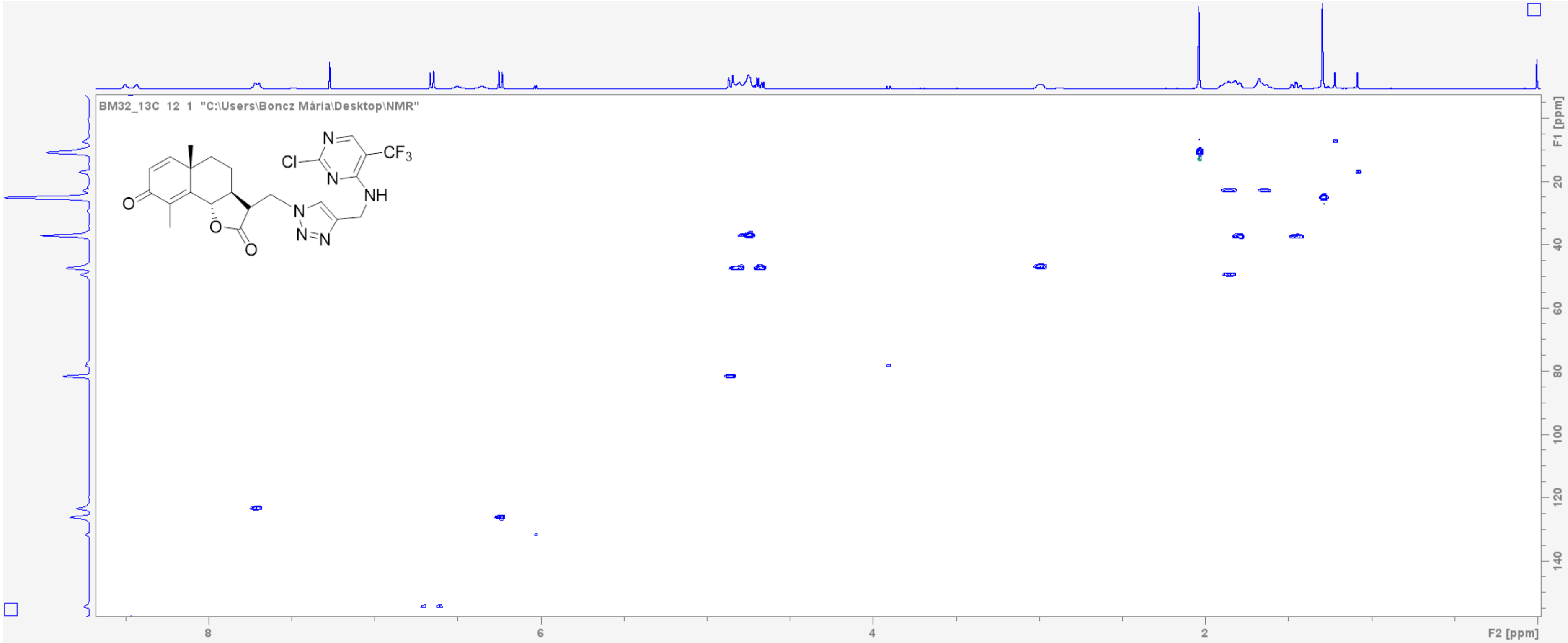

Figure S84 HMBC of compound **56**

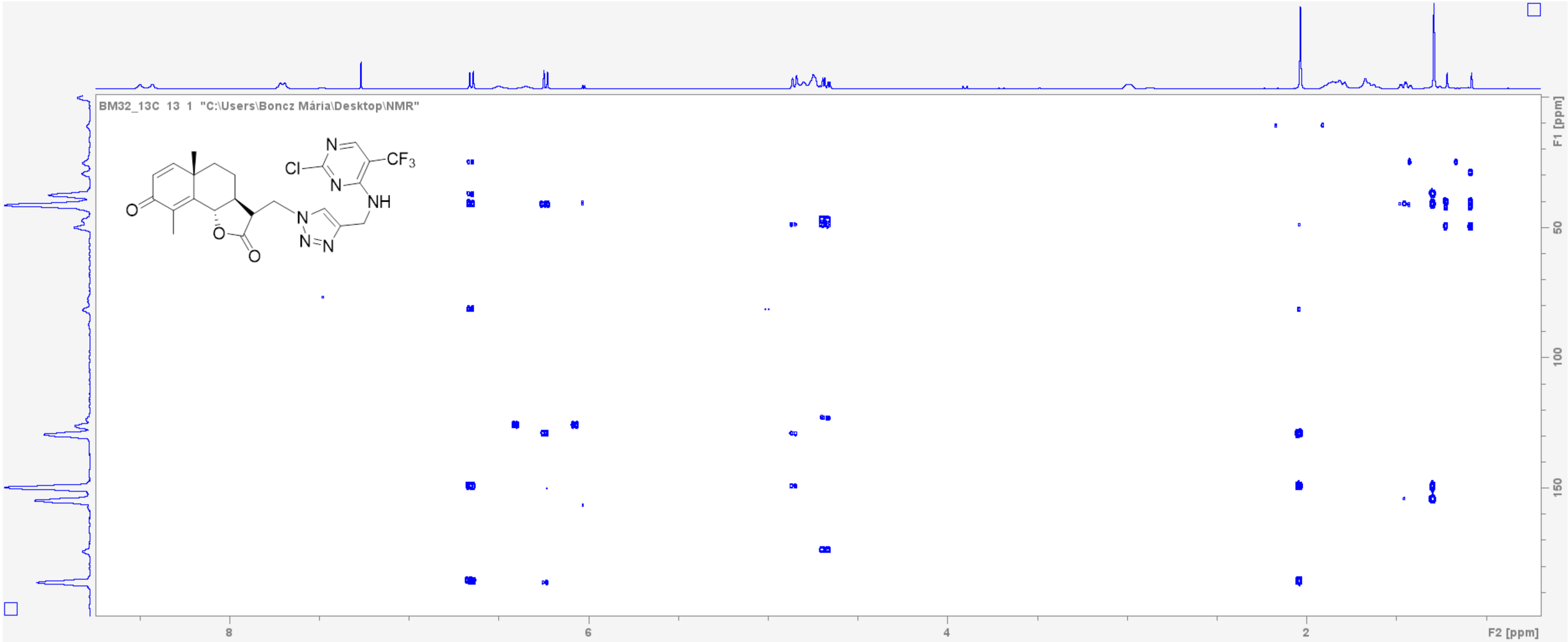

Figure S85 ^{19}F-NMR of compound **56**

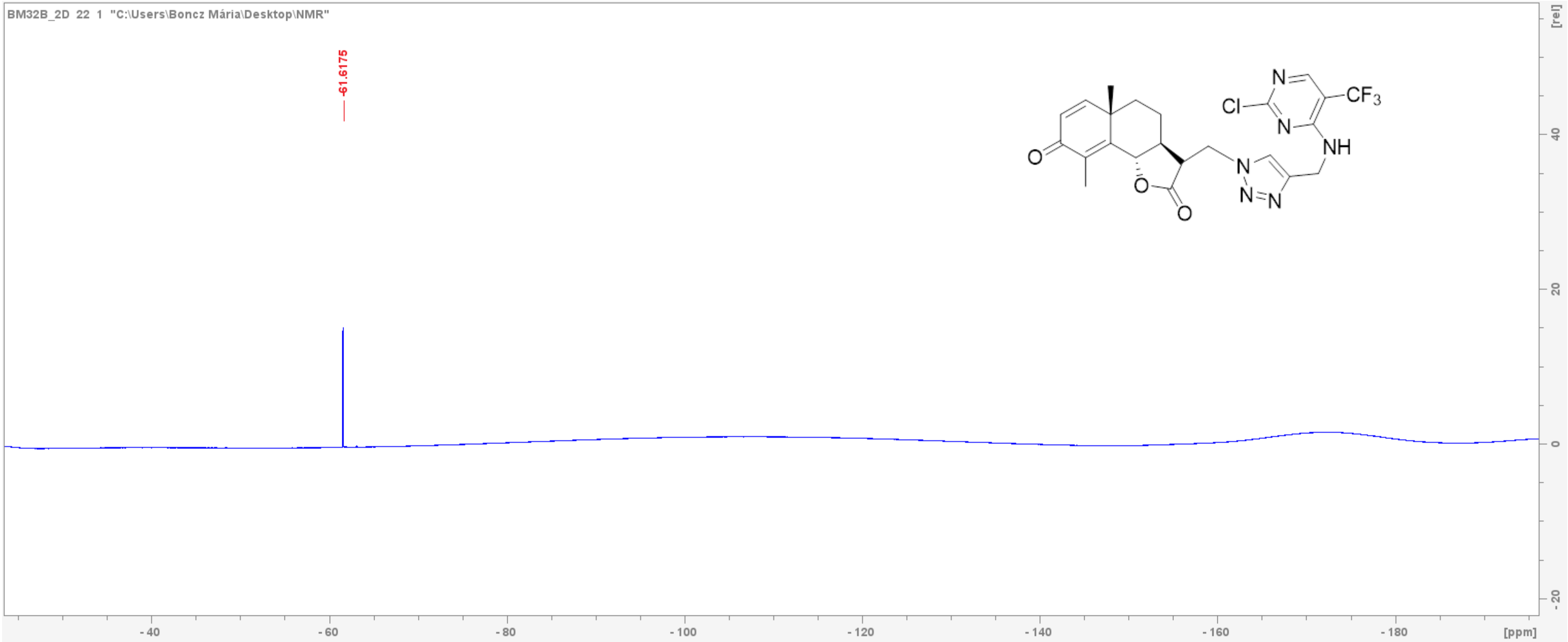

(5a*S*,9b*S*)-5a,9-dimethyl-3-((4-(((5-chloro-2-((4-(trifluoromethyl)phenyl)amino)pyrimidin-4-yl)amino)methyl)-1*H*-1,2,3-triazol-1-yl)methyl)-3a,5,5a,9b-tetrahydronaphtho[1,2-*b*]furan-2,8(3*H*,4*H*)-dione (**57**)

Figure S86 ^{1}H of compound **57**

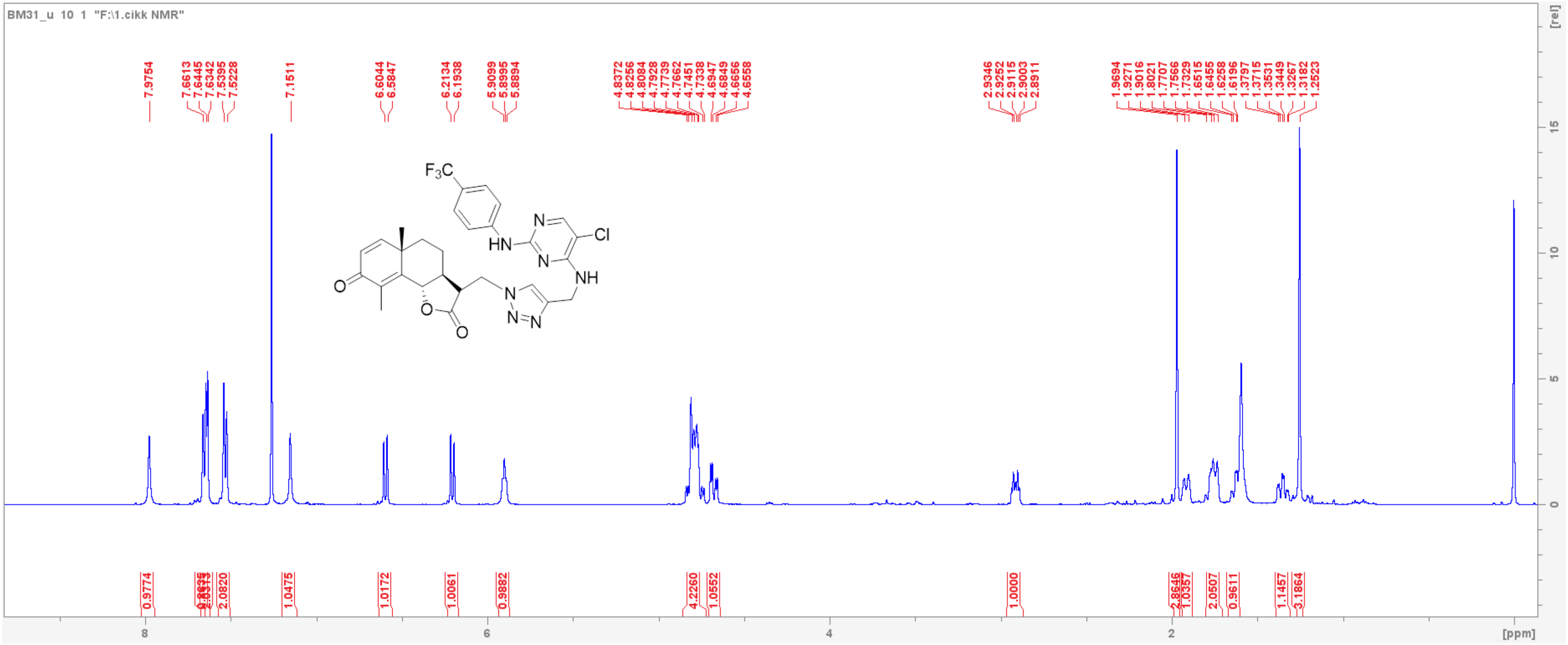

Figure S87 ^{13}C-NMR of compound **57**

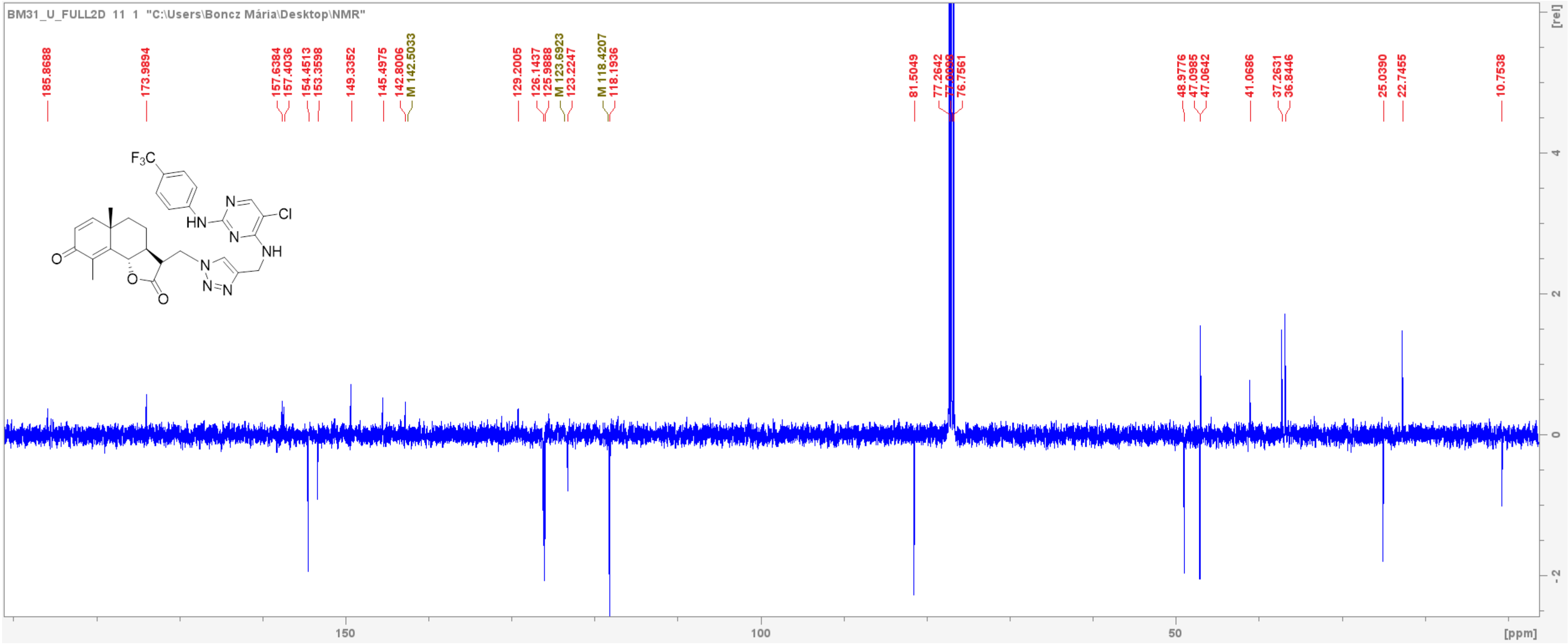

Figure S88 HSQC of compound **57**

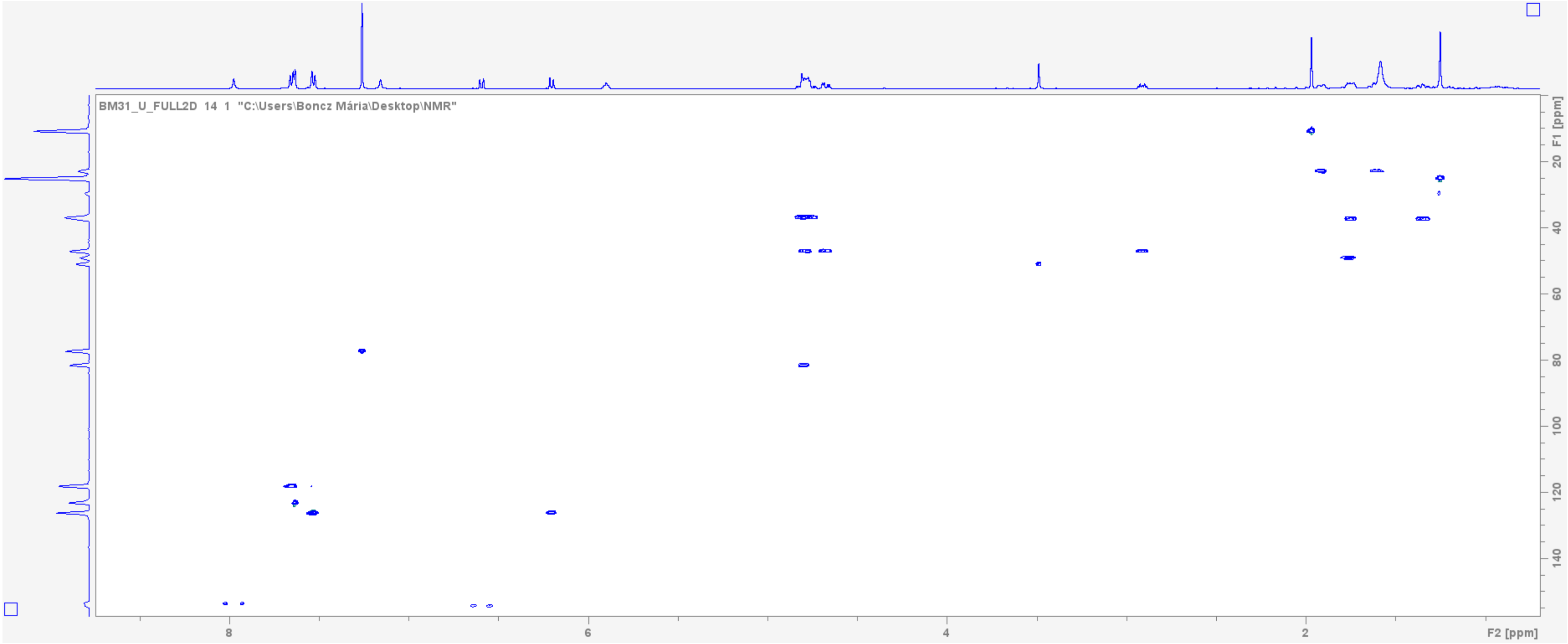

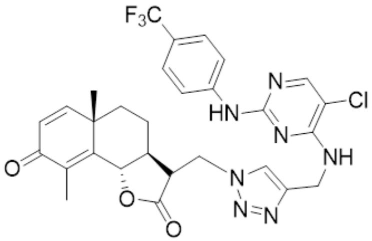

Figure S89 HMBC of compound **57**

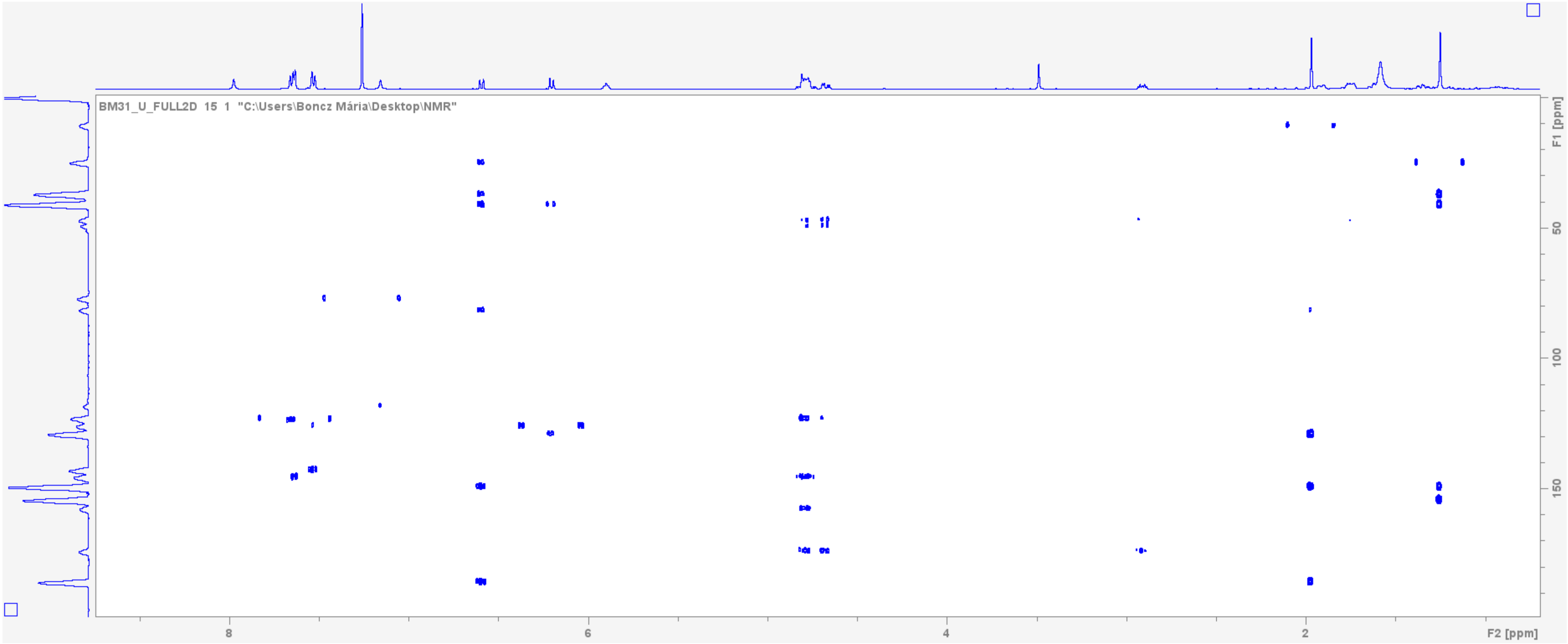

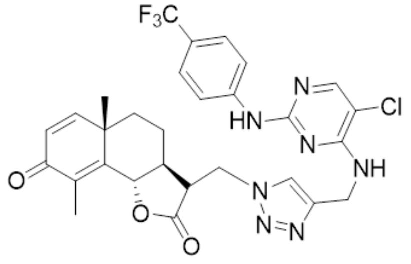

Figure S90 ^{19}F-NMR of compound **57**

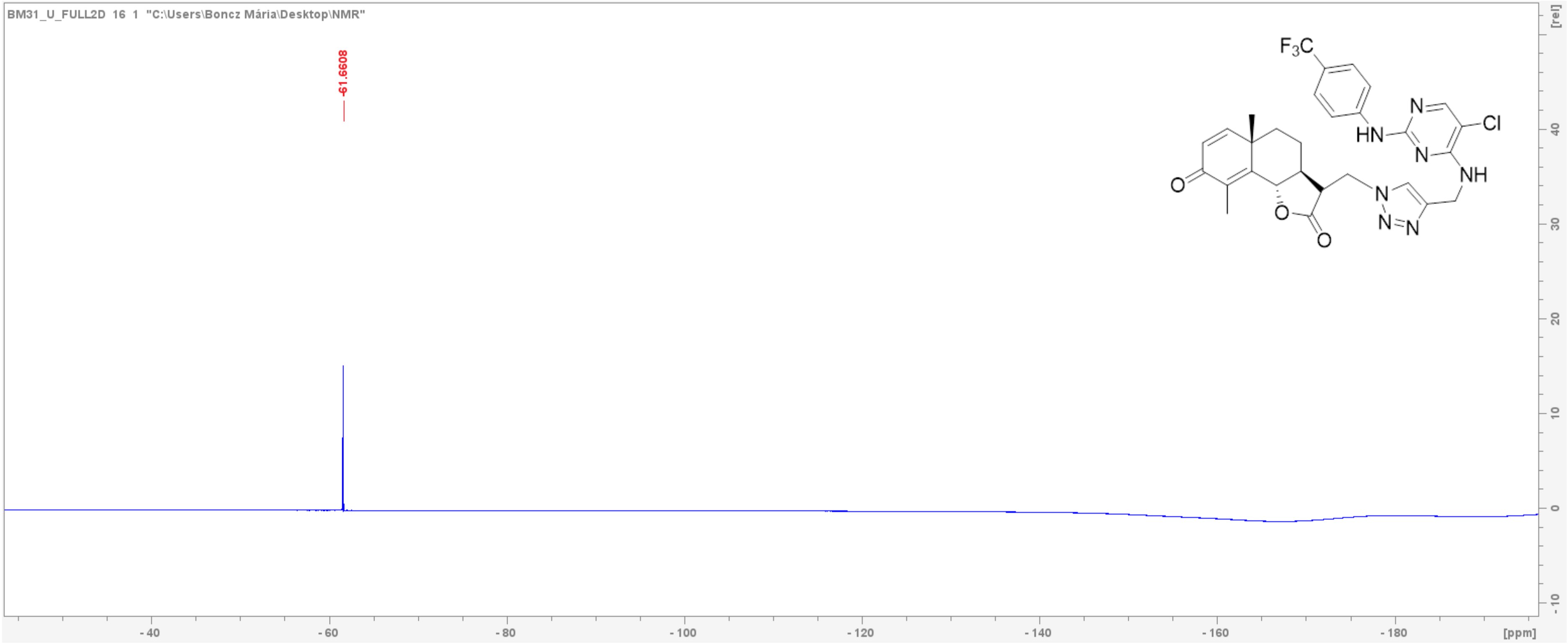

(5a*S*,9b*S*)-5a,9-dimethyl-3-((4-(((5-fluoro-2-((4-(trifluoromethyl)phenyl)amino)pyrimidin-4-yl)amino)methyl)-1*H*-1,2,3-triazol-1-yl)methyl)-3a,5,5a,9b-tetrahydronaphtho[1,2-*b*]furan-2,8(3*H*,4*H*)-dione (**58**)

Figure S91 ^{1}H-NMR of compound **58**

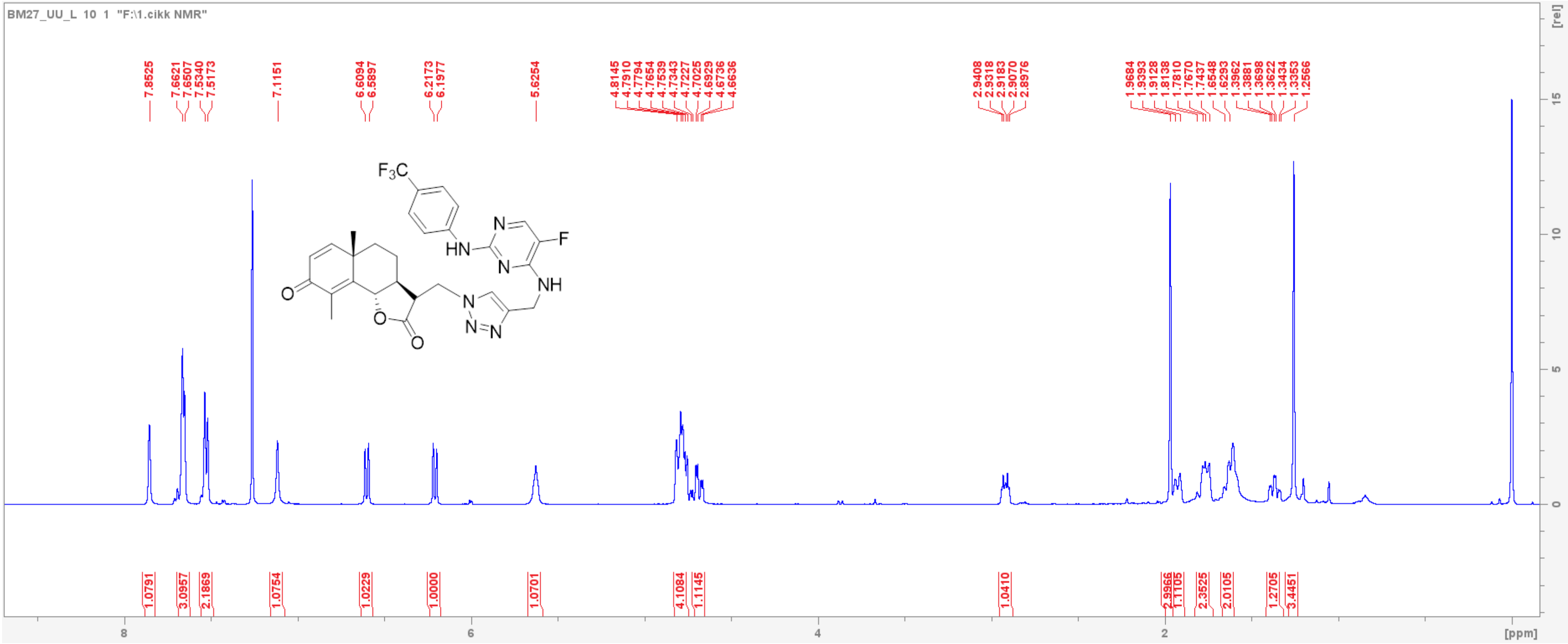

Figure S92 ^{13}C-NMR of compound **58**

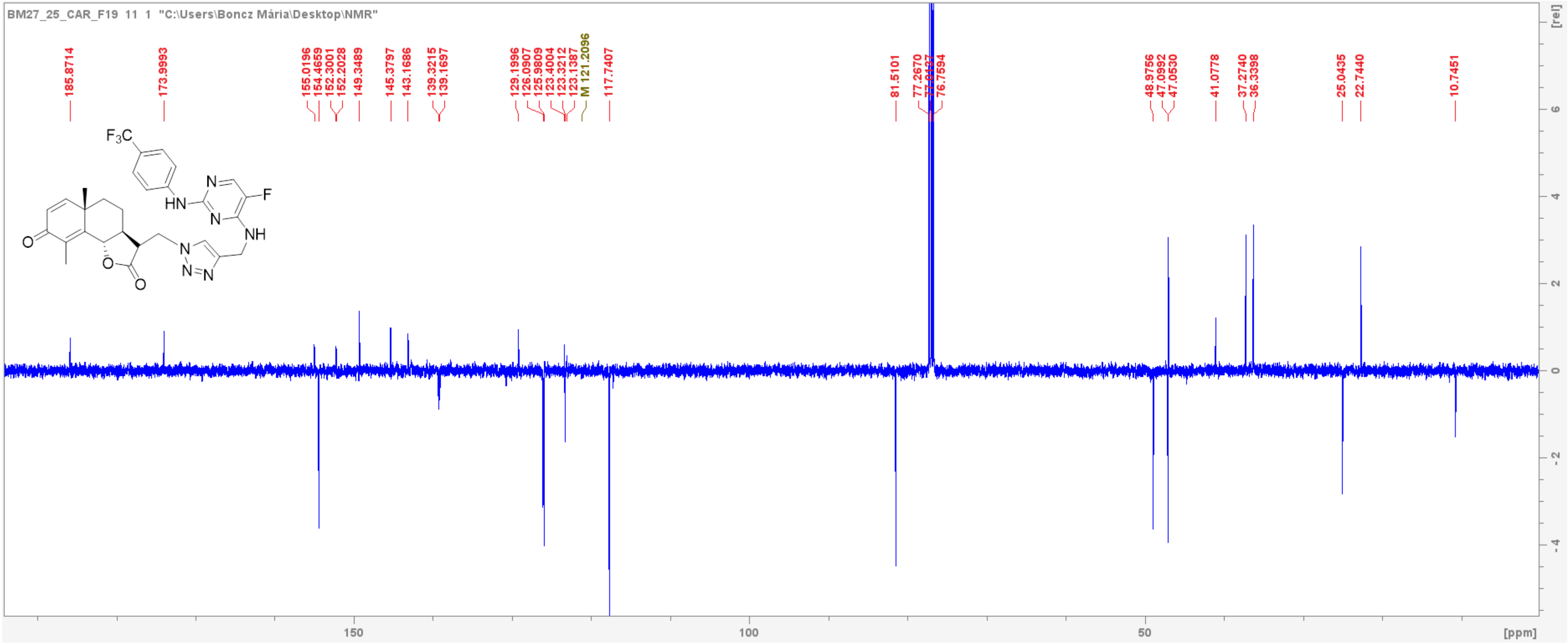

Figure S93 HMBC of compound **58**

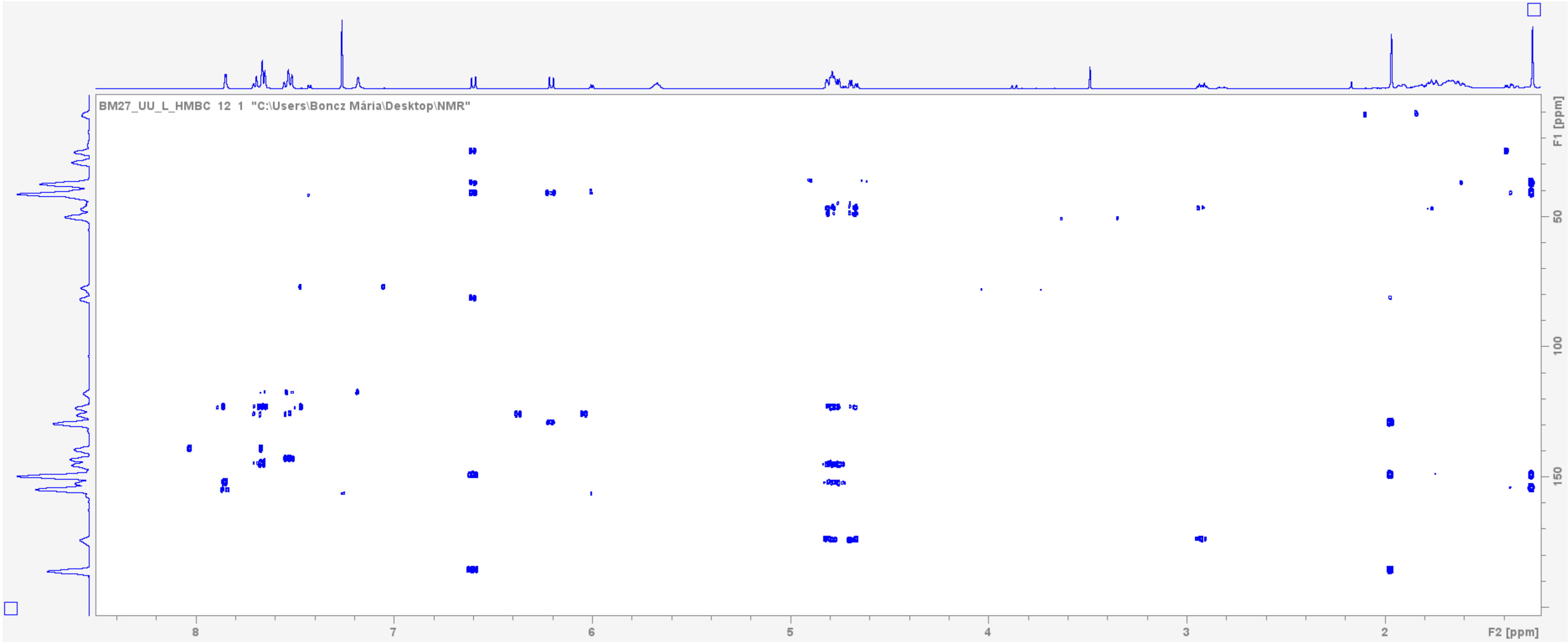

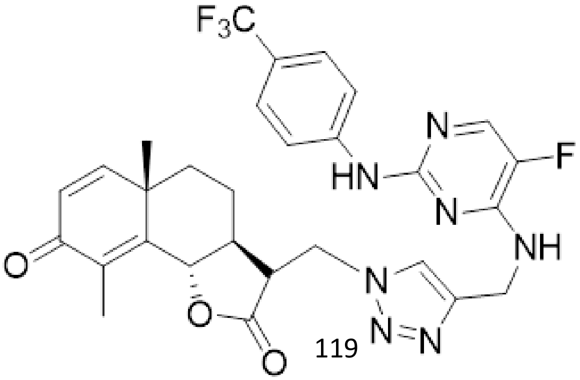

Figure S94 ^{19}F-NMR of compound **58**

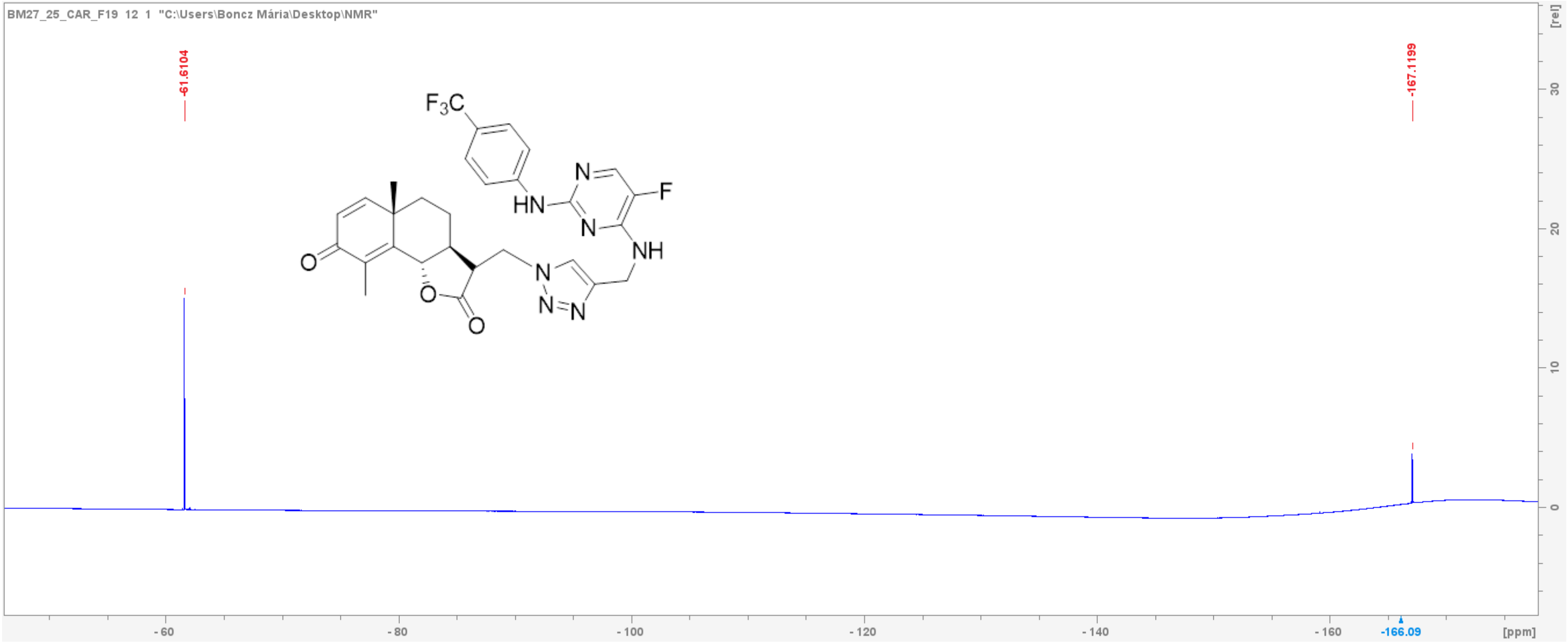

(5a*S*,9b*S*)-5a,9-dimethyl-3-((4-(((5-fluoro-4-((4-(trifluoromethyl)phenyl)amino)pyrimidin-2-yl)amino)methyl)-1*H*-1,2,3-triazol-1-yl)methyl)-3a,5,5a,9b-tetrahydronaphtho[1,2-*b*]furan-2,8(3*H*,4*H*)-dione (**59**)

Figure S95 ^{1}H-NMR of compound **59**

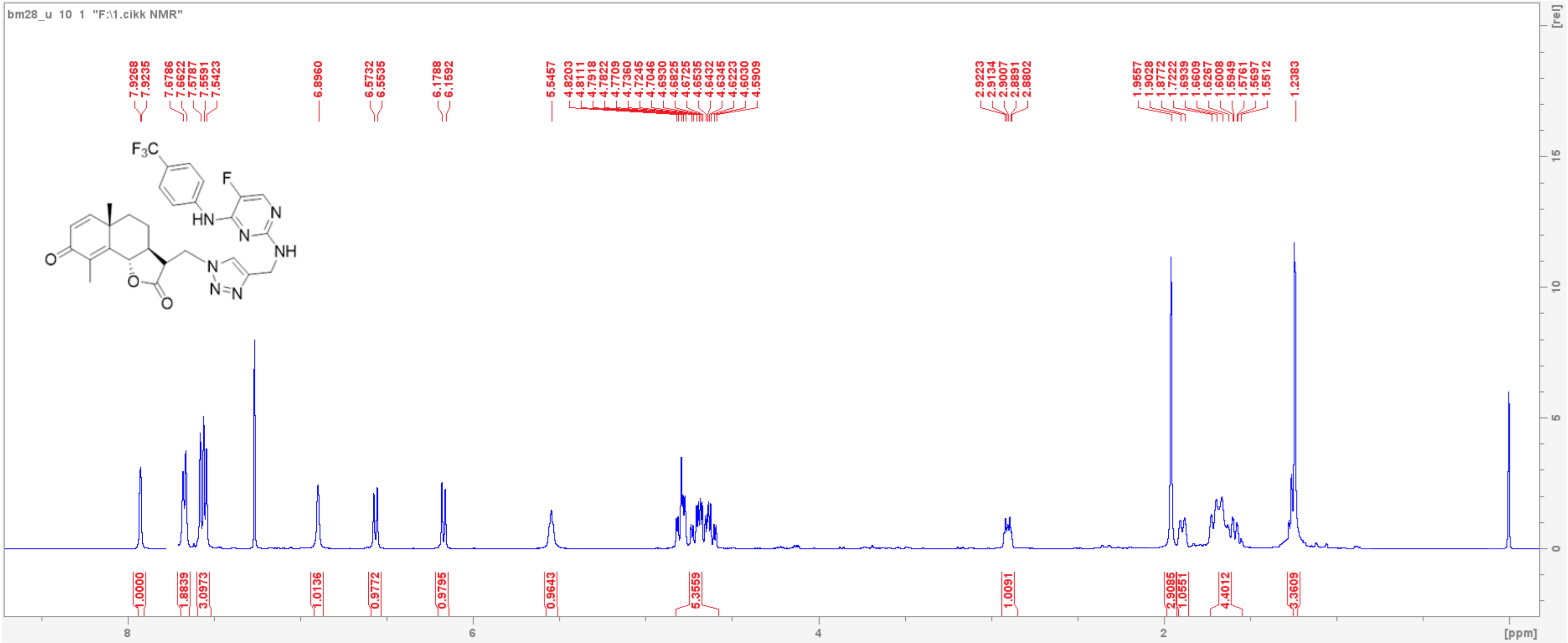

Figure S96 ^{13}C-NMR of compound **59**

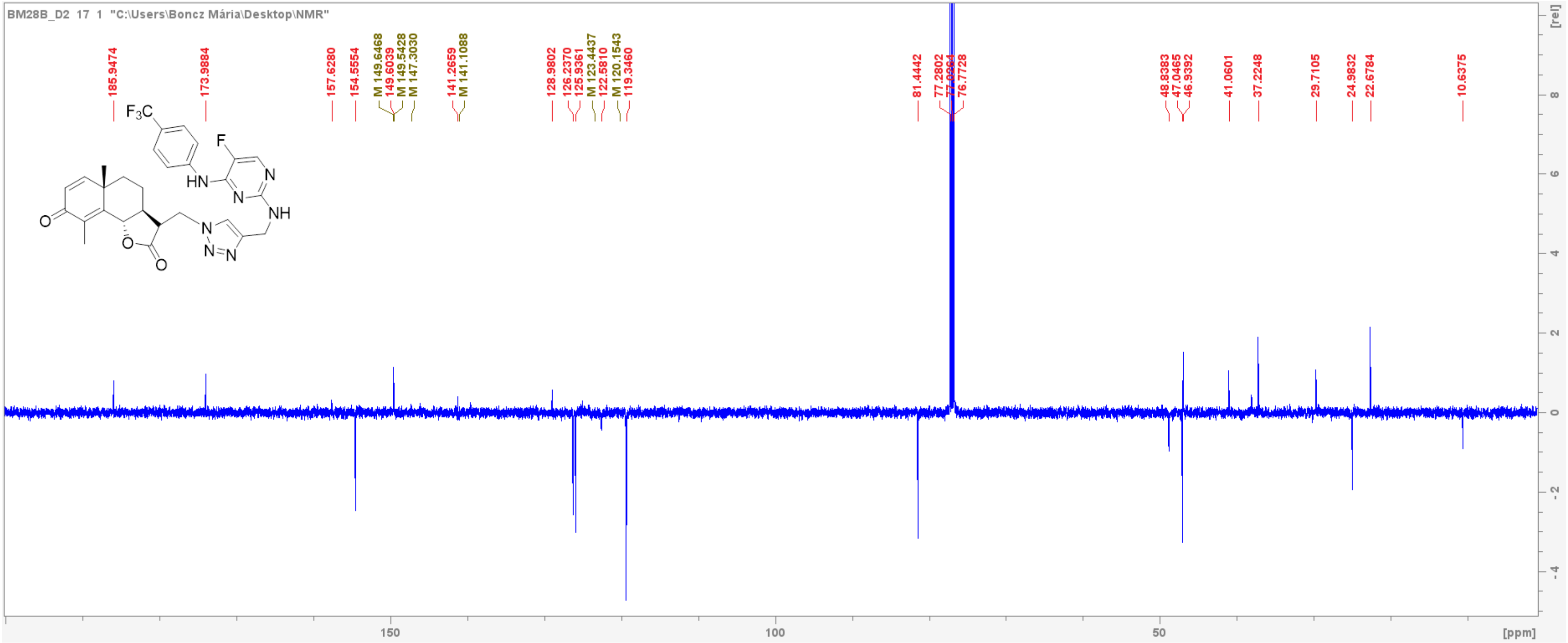

Figure S97 HSQC of compound **59**

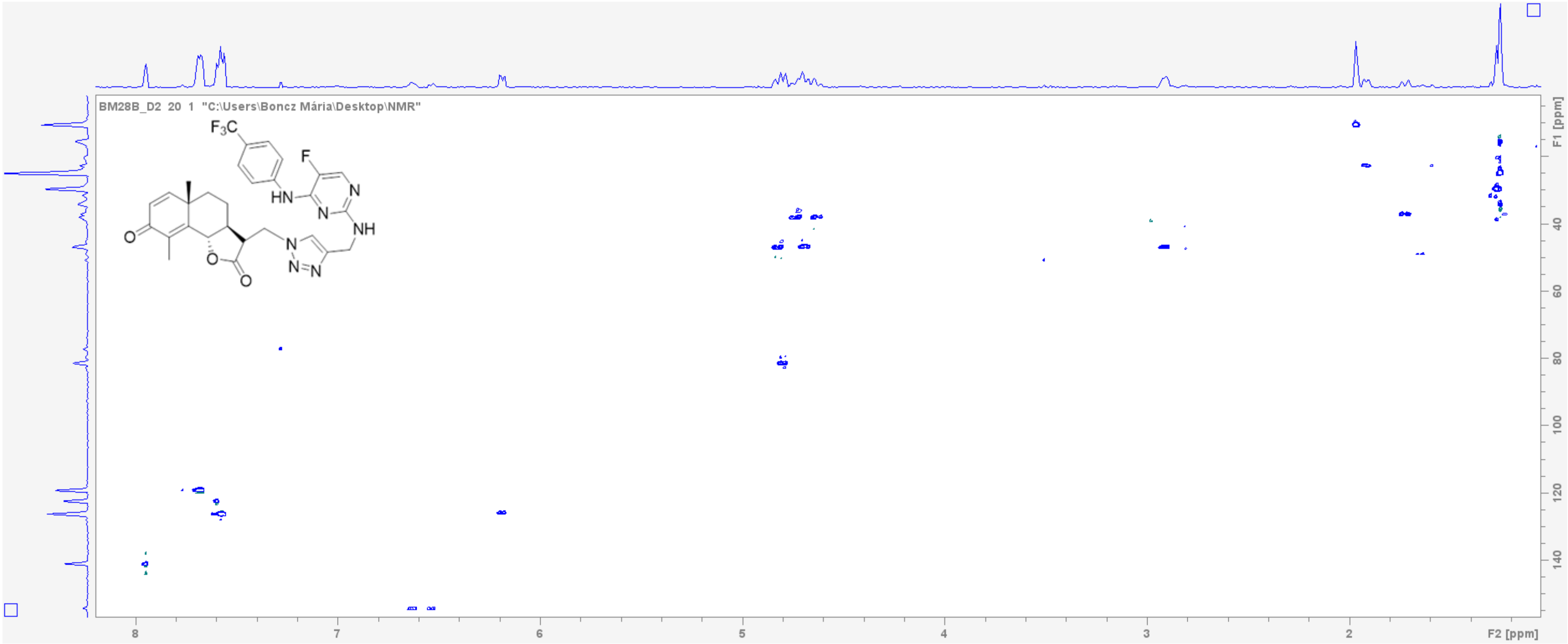

Figure S98 HMBC of compound **59**

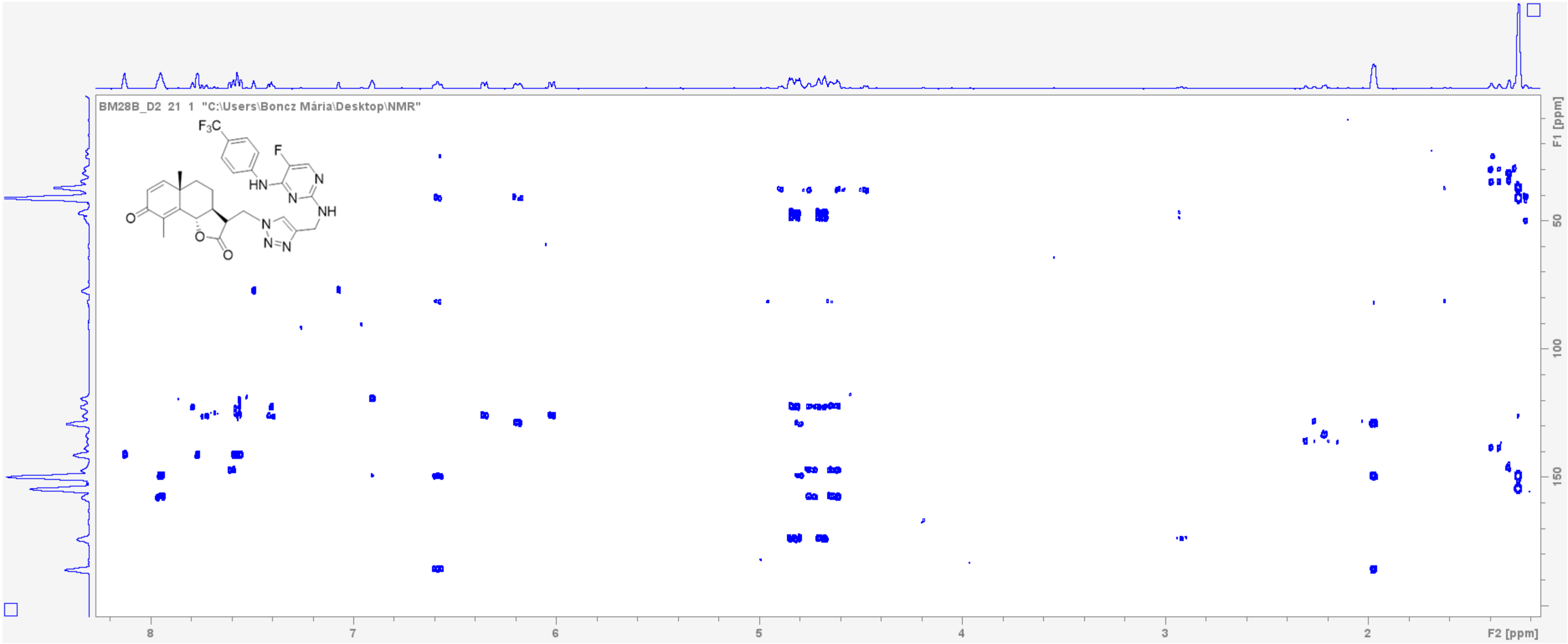

Figure S99 HMBC of compound **59**

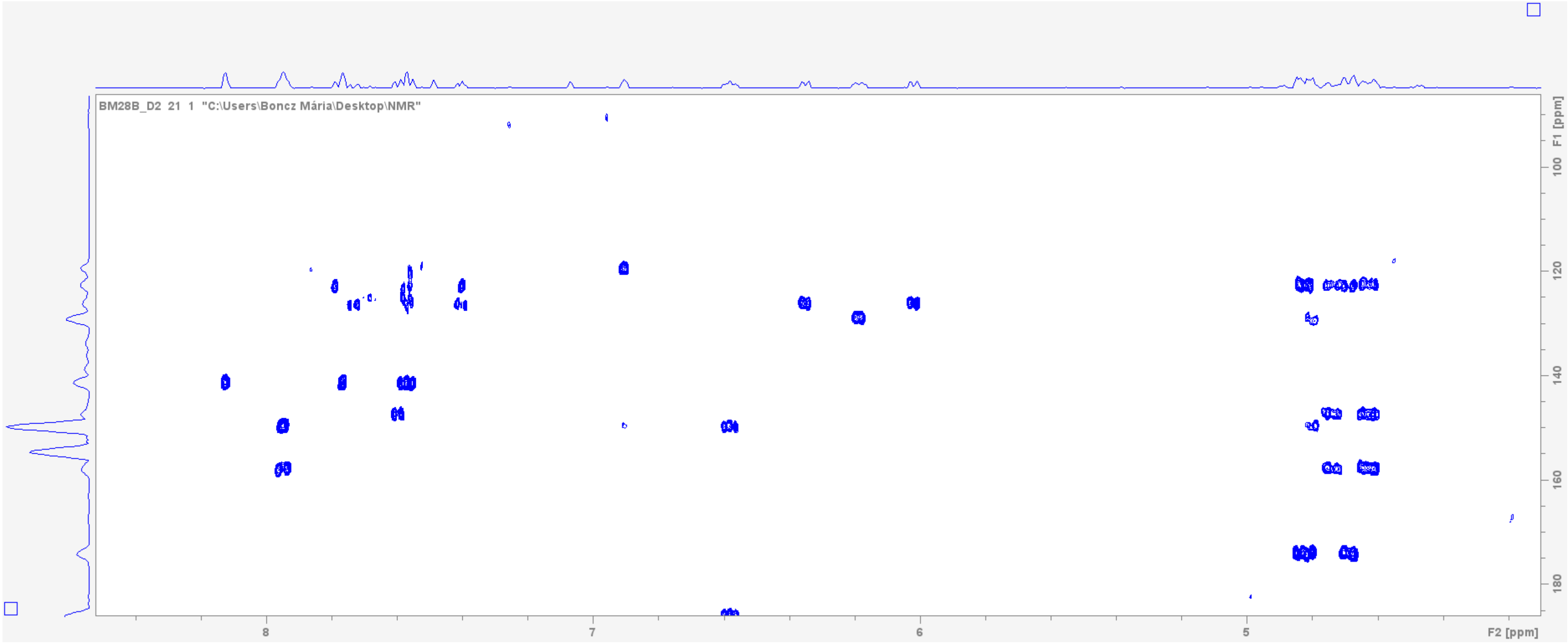

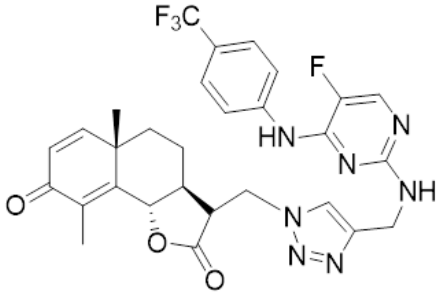

Figure S100 ^{19}F-NMR of compound **59**

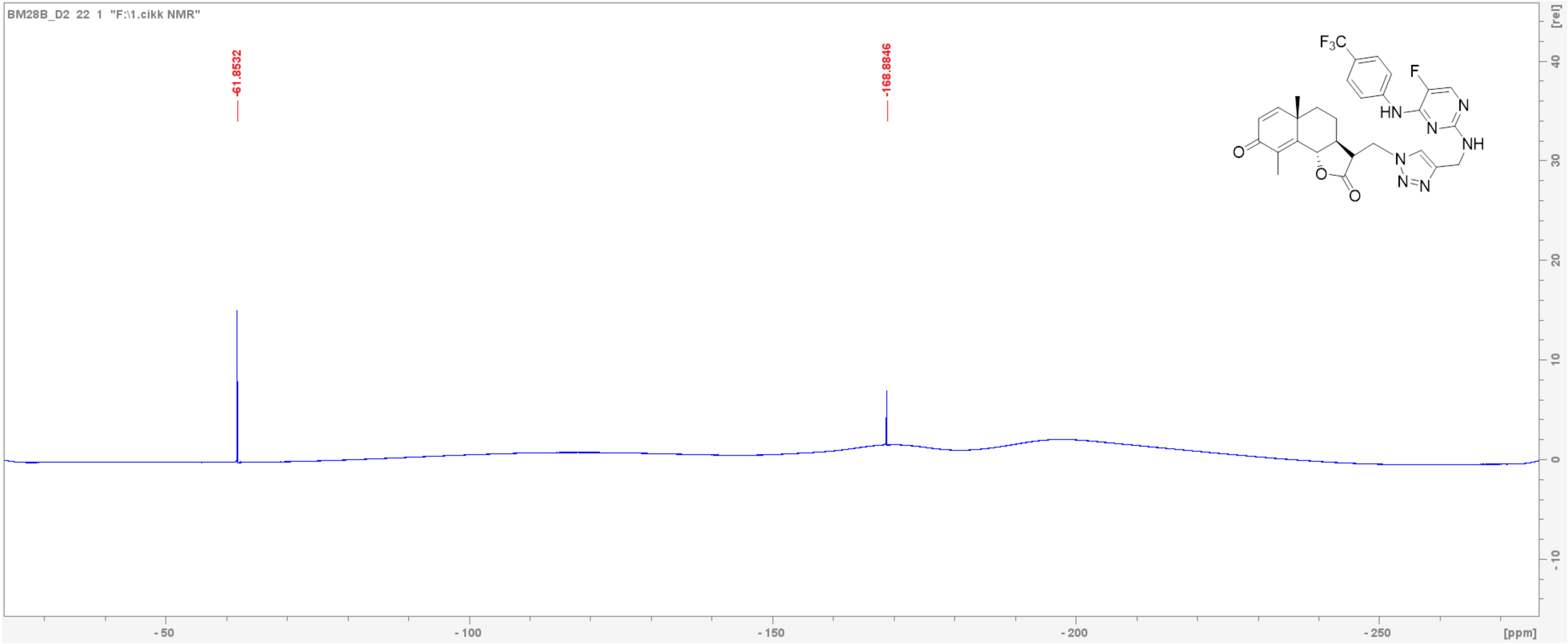

Supplement: Supplementary file 1 [file antibiotics-15-00611-s001.zip › antibiotics-4322438-supplementary.pdf]
